# Palladium-Catalyzed Direct C=H Functionalization of Benzoquinone**

**DOI:** 10.1002/anie.201408054

**Published:** 2014-10-10

**Authors:** Sarah E Walker, James A Jordan-Hore, David G Johnson, Stuart A Macgregor, Ai-Lan Lee

**Affiliations:** Institute of Chemical Sciences, Heriot-Watt UniversityEdinburgh EH14 4AS (UK)

**Keywords:** benzoquinone, C=H functionalization, palladium, synthetic methods, water

## Abstract

A direct Pd-catalyzed C=H functionalization of benzoquinone (BQ) can be controlled to give either mono- or disubstituted BQ, including the installation of two different groups in a one-pot procedure. BQ can now be directly functionalized with aryl, heteroaryl, cycloalkyl, and cycloalkene groups and, moreover, the reaction is conducted in environmentally benign water or acetone as solvents.

Benzoquinone (BQ, **1**)[2] and its derivatives are ubiquitous in organic chemistry as they are useful in many fields, such as oxidation chemistry,[3] molecular electronics,[4] medicinal chemistry,[5] natural products,[6] dyes,[7] and as ligands.[8] Despite the prevalence of Pd^0^-catalyzed cross-couplings for the formation of C=C bonds, a method for the direct Pd-catalyzed Heck coupling with BQ has so far eluded synthetic chemists. This reflects its electronic properties: BQ and its derivatives will often act as an oxidant[3] or ligand[8a–c] rather than a substrate in Pd catalysis.[9,10] As a result, for decades, the controlled Pd-catalyzed cross-coupling of BQ has relied on first installing a Br, I, or OTf group (substrate **2**), followed by a Stille or Suzuki coupling (**2**→**3**, Scheme 1).[11,12] This procedure involves additional steps but may also suffer from chemo- and regioselectivity issues during halogenations.[13] The direct C=H functionalization of BQ would clearly expedite the synthesis of BQ-containing targets, but to date, an efficient Pd-catalyzed monofunctionalization has proven elusive.[10,14]

**Scheme 1:**
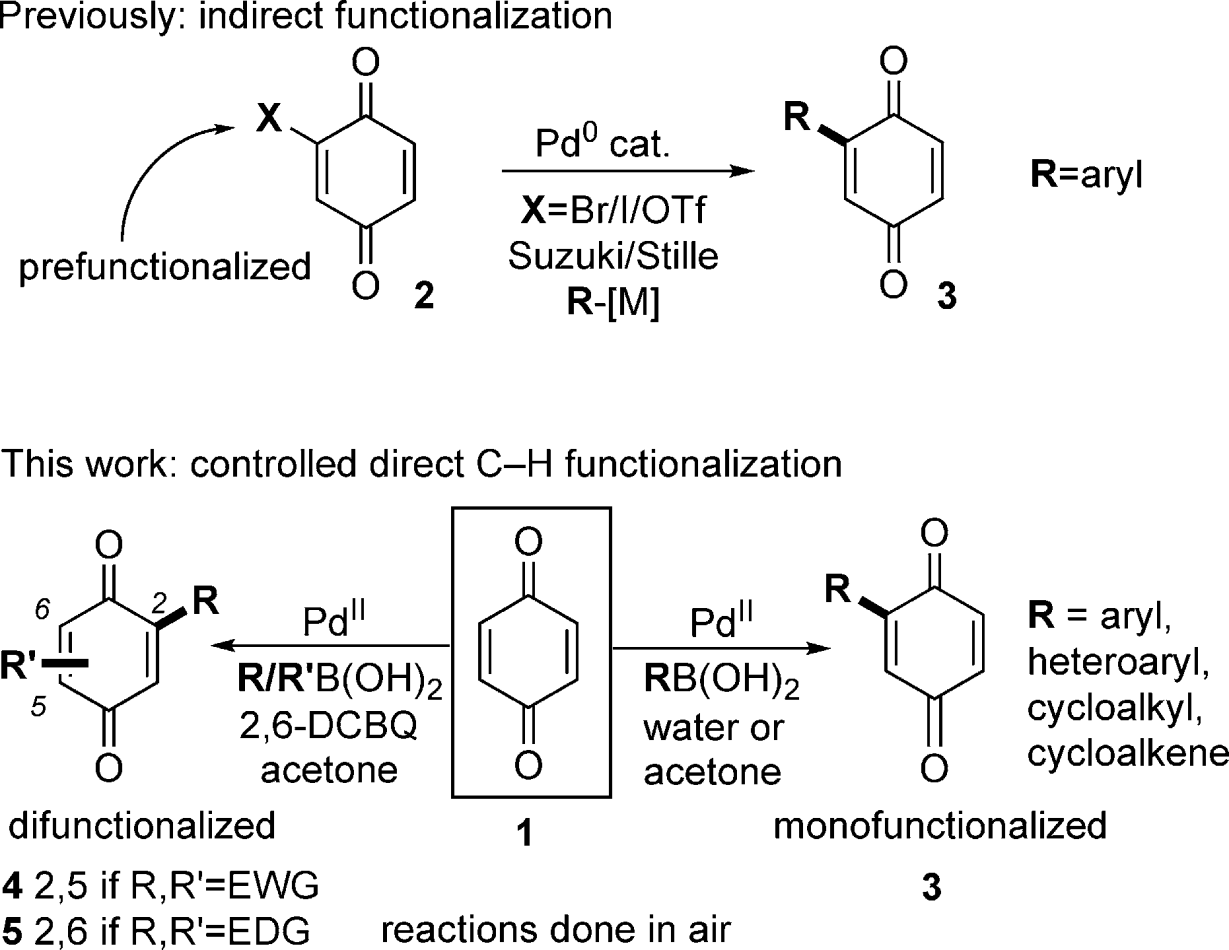
Pd-catalyzed methods for the functionalization of BQ.

Current methods for the direct functionalization of BQ are based on the Meerwein arylation.[15] This approach, however, utilizes potentially explosive diazonium salt precursors, proceeds through a radical mechanism, and is limited to arylations. Baran et al. have recently reported a Ag-catalyzed C=H monofunctionalization of BQs using boronic acids[16] and a strong co-oxidant (K_2_S_2_O_8_), which is also thought to proceed through a radical mechanism.[17] However, strong oxidants preclude the use of attractive cross-coupling partners with readily oxidizable (e.g. benzylic) positions.[16b] Furthermore, no examples of functionalizations with heterocycles or alkenes are known and the radical methods are so far mainly useful only for monofunctionalizations. A mild and practical Pd-catalyzed method, capable of either mono- or difunctionalization, is therefore highly desirable.[18]

Initial attempts at Pd-catalyzed C=H arylation of BQ with the Pd(OTf)_2_ system used in our previous work[19] gave either irreproducible results or complex mixtures of mono- and various diarylated products. After extensive optimization, we found that the less active catalyst Pd(OCOCF_3_)_2_ allowed for controlled monofunctionalization in either acetone or water as solvent[20] (Table 1). This required BQ to be used in excess (optimally 3 equiv, see the Supporting Information); one equivalent acting as an oxidant in the reaction and a further equivalent ensuring the reaction stops after monofunctionalization. As BQ is far cheaper than organoboronic acids, it makes sense to use the latter as the limiting reagent.

**Table 1:** C=H monofunctionalization of BQ.[a] 
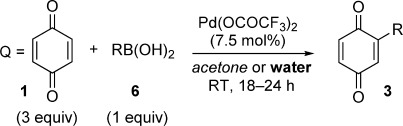

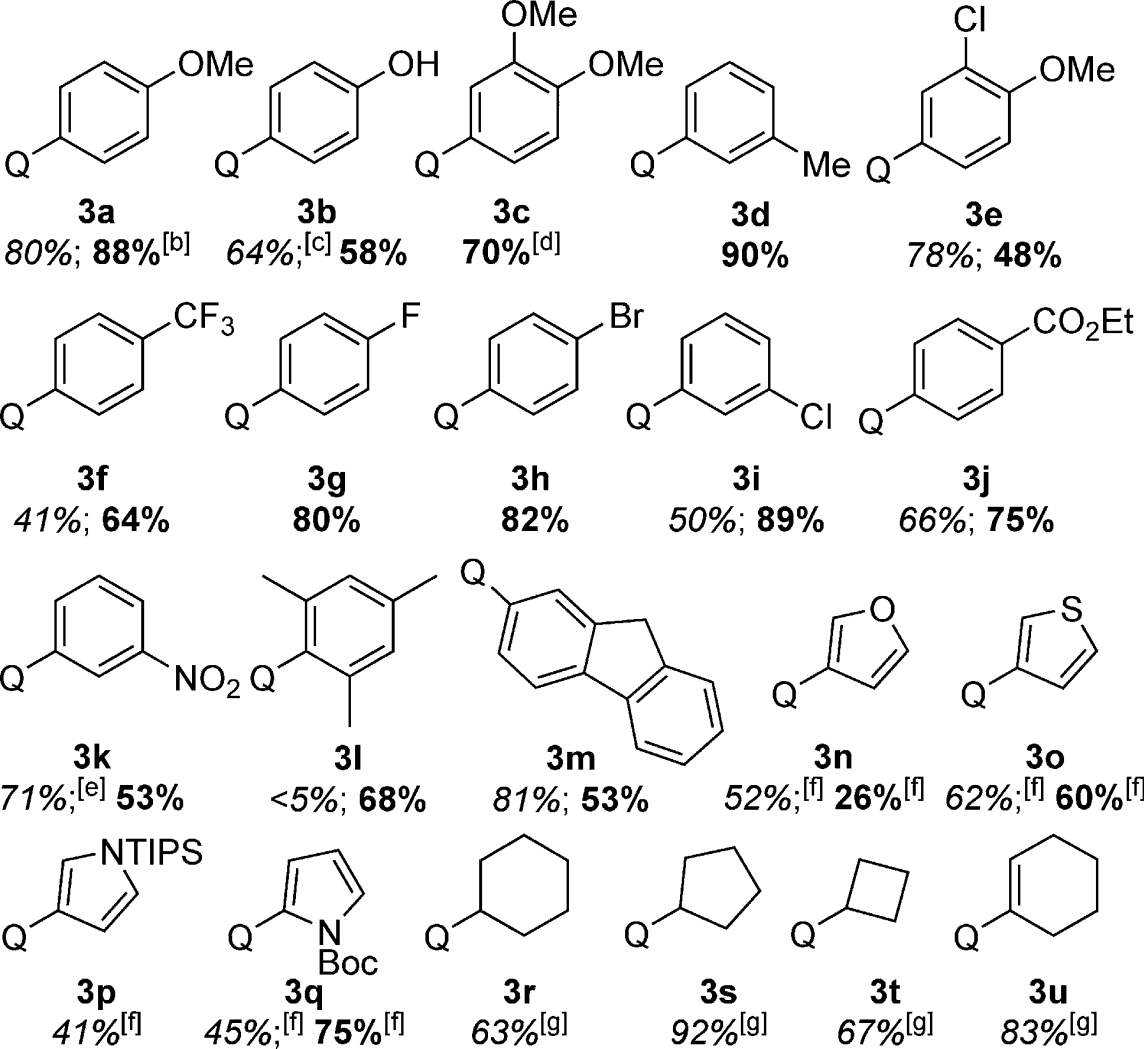

[a] Yields of isolated products are given. For yields in italics, acetone was used as solvent; for yields in bold, water was used as solvent. If only one yield is given, the reaction in the other solvent proceeded with poor conversion and the product was not isolated.

[b] 87 % with 1 mol % catalyst.

[c] 6 equiv BQ used.

[d] Gram-scale reaction resulted also in 70 % yield.

[e] 50 °C, 48 h.

[f] 40 h.

[g] 40 °C, 48 h.

Using our optimized conditions, the scope of organoboronic acids was investigated (Table 1). Aryl boronic acids with electron-rich, electron-poor, and *ortho*, *meta*, and *para* substituents all performed well (products **3 a**–**3 k**). Even hindered mesityl- (**3 l**) and readily oxidizable fluorene boronic acids (**3 m**) underwent the reaction smoothly (the latter being incompatible with existing Ag-catalyzed radical methods[16b]). Some boronic acids provided better yields in acetone, while others fared better in water; the two solvents seeming to complement each other. Heterocyclic boronic acids are also suitable substrates (**3 n**–**3 q**), as are cycloalkyl- and cycloalkene boronic acids (**3 r**–**3 u**).[21] Some of the more active aryl boronic acids reacted well with lower catalyst loadings, for example, **3 a** was produced in 87 % yield with only 1 mol % catalyst.[22] This air- and water-tolerant reaction could also be carried out on a gram scale: **3 c** was produced in 70 % yield on both 1 mmol and 10.5 mmol scale.

Having developed an efficient Pd-catalyzed direct C=H monofunctionalization of BQ, we sought to extend this to a controlled C=H difunctionalization. Diarylated BQs have found diverse uses as ligands,[8] in molecular electronics,[4b] natural products,[23] and biologically active compounds,[24] despite their multi-step syntheses, which are often restricted to homo-disubstituted BQs.[2,25] We first explored a one-pot homo-difunctionalization (R=R′ in Scheme 1). In a one-pot procedure, an excess of BQ cannot be used, but without this excess of BQ, the monoarylated intermediate **3** acts as an oxidant. Success therefore relied on finding a suitable sacrificial oxidant, and extensive screening showed 2,6-dichloro-1,4-benzoquinone (2,6-DCBQ) to be ideal and allowed a range of homo-diarylations to be investigated (Table 2).

**Table 2:** C=H homo-difunctionalization of BQ: dependence of the selectivity on the electronics of the substituent. 
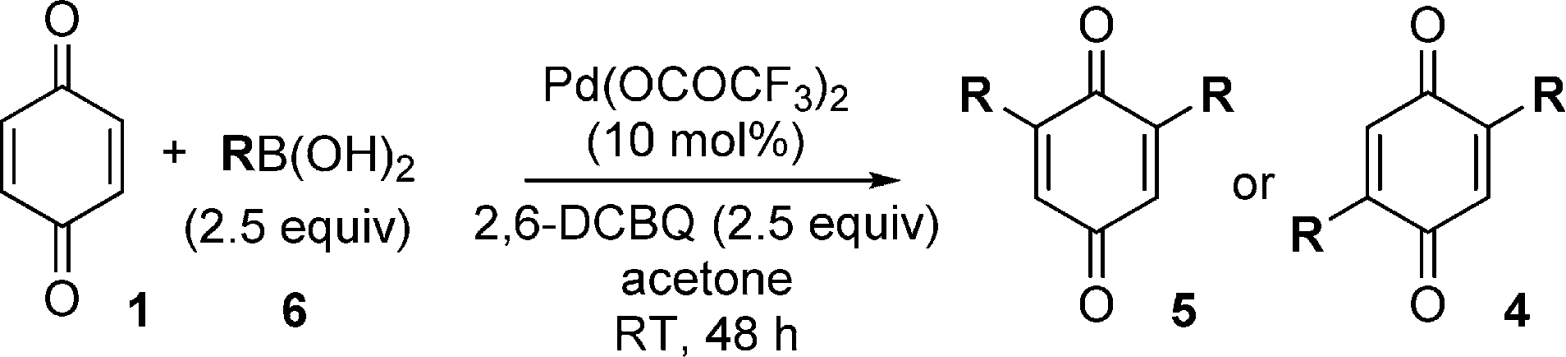

Entry	R	Yield5 [%][a]	Yield4 [%][a]
1	*p*-HO-C_6_H_4_	71 (**5 a**)	<5 (**4 a**)
2	*m*-MeO-*p*-HO-C_6_H_3_	73 (**5 b**)	trace
3	*m*,*p*-(MeO)_2_-C_6_H_3_	58 (**5 c**)	n.d.
4	*m*-tolyl	53 (**5 d**)	28 (**4 d**)
5	Ph	29 (**5 e**)	44 (**4 e**)
6[b]	*p*-F_3_C-C_6_H_4_	–	51 (**4 f**)
7[b–d]	*p*-EtO_2_C-C_6_H_4_	–	25 (**4 g**)
8	*o*-Me-*p*-HO-C_6_H_3_	41[e] (**5 h**/**4 h**=1:1)	
9	*o*-MeO-C_6_H_4_	25 (**5 i**)	28 (**4 i**)

[a] Yields of isolated products are given.

[b] At 35 °C.

[c] Additional 2,6-DCBQ, catalyst, and boronic acid added, treated with FeCl_3_ at the end of reaction.

[d] Product only moderately stable.

[e] Isomers not fully separable. n.d.=not determined.

Under our optimized conditions, the selectivity for 2,6 disubstitution (**5**) or 2,5 disubstitution (**4**) appears to be controlled by the electronic nature of the substituent that is introduced (R). For example, strongly electron-donating substituents provide the 2,6 isomers **5 a**–**c**, selectively (Table 2, entries 1–3). A weakly electron-donating substituent (*meta*-tolyl) reduces the selectivity, but **5 d** is still the major product (Table 2, entry 4), whereas an electron-neutral substituent (phenyl) gives a poor 1:1.5 ratio of **5 e**/**4 e** (entry 5).[26] Electron-withdrawing substituents reverse the preference, with **4 f** and **4 g** formed selectively (Table 2, entries 6 and 7). Such products seem relatively unstable compared to their counterparts with electron-donating groups and this may contribute to the lower yields of isolated products in these cases.[27] Finally, *ortho* substituents on the aryl ring are detrimental for selectivity (Table 2, compare entry 8 with entry 1), presumably because of steric factors (entries 8 and 9).

With the selectivity and trends for the homo-difunctionalizations in hand, we addressed the more challenging issue of C=H hetero-difunctionalization, in which two different R groups are introduced. Controlled and selective hetero-difunctionalizations are not feasible with traditional methods (see before). Initially, a stepwise procedure utilizing the monofunctionalized BQs **3** (Table 1) was investigated, with the second substituent (R′) being introduced using modified conditions from our homo-difunctionalization reactions.

The same selectivity trends seen for 2,5 or 2,6 homo-difunctionalizations also apply to hetero-difunctionalizations (Table 3). With two electron-donating substituents, the 2,6 isomers **5 j**–**l** are the major products, with higher selectivities observed for more electron-donating substituents (Table 3, entries 1–3). An *ortho* substituent causes a drop in selectivity (**5 m**/**4 m**=5:4, Table 3, entry 4). A combination of electron-donating and electron-poor groups leads, unsurprisingly, to a lower selectivity, independent of the installation order (Table 3, entries 5 and 6). Two different electron-poor substituents cause a switch in selectivity to the 2,5 isomer **4** (Table 3, entry 7), and even a mixed-heterocyclic difunctionalized BQ can be produced selectively in good yield as the 2,5 isomer (**4 p**, 74 %, entry 8). The employment of a mixture of electron-withdrawing aryl and heterocyclic groups also favors the formation of isomer **4** (Table 3, entries 9 and 10), with lower yields reflecting the apparently poorer stability of these products.[27] A current limitation is that alkyl boronic acids, though efficient in monofunctionalizations, are not suitable substrates for difunctionalizations (Table 3, entry 11).

**Table 3:** C=H hetero-difunctionalization of BQ. 
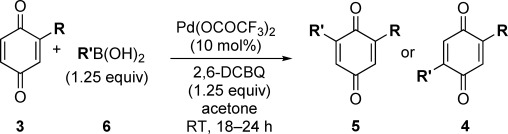

Entry	R	R′	Yield5[%][a]	Yield4[%][a]
1	*p*-MeO-C_6_H_4_	*p*-HO-C_6_H_4_	73 (**5 j**)	<5 (**4 j**)
2	*m*,*p*-(MeO)_2_-C_6_H_3_	*p*-HO-C_6_H_4_	71 (**5 k**)	10 (**4 k**)
3	*m*,*p*-(MeO)_2_-C_6_H_3_	*p*-MeO-C_6_H_4_	65 (**5 l**)	21 (**4 l**)
4	*p*-HO-C_6_H_4_	*o*-MeO-C_6_H_4_	50 (**5 m**)	41 (**4 m**)
5	*m*,*p*-(MeO)_2_-C_6_H_3_	*p*-EtO_2_C-C_6_H_4_	44 (**5 n**)	26 (**4 n**)
6	*p*-EtO_2_C-C_6_H_4_	*m*,*p*-(MeO)_2_-C_6_H_3_	48 (**5 n**)	16 (**4 n**)
7[d]	*p*-EtO_2_C-C_6_H_4_	*p*-F_3_C-C_6_H_4_	<5 (**5 o**)	47 (**4 o**)
8[b]	*N*-Boc-pyrrole-2	3-thiophene	trace	74 (**4 p**)
9[b–d]	*m*-O_2_N-C_6_H_4_	3-thiophene	trace	42 (**4 q**)
10[d]	*p*-EtO_2_C-C_6_H_4_	3*-*thiophene	trace	34 (**4 r**)
11	*m*,*p*-(MeO)_2_-C_6_H_3_	cyclohexyl	–[e]	–

[a] Yields of isolated products.

[b] 2.5 equiv of boronic acid **6** used.

[c] Treated with FeCl_3_ at the end of reaction.

[d] Product only moderately stable.

[e] Complex mixture of products.

A final target was the establishment of a simple one-pot C=H hetero-difunctionalization procedure. Again this required a reduction in the amount of BQ used in the monofunctionalization (3 equiv). Following optimization (see the Supporting Information), use of 1.5 equivalents of BQ with 1.5 equivalents of 2,6-DCBQ was adopted as optimal to produce **3 a** in situ, followed by the addition of a second different aryl boronic acid to successfully give the hetero-difunctionalized product **5 j** in 47 % yield over two steps (Scheme 2), equivalent to a good average of 69 % for each step.

**Scheme 2:**
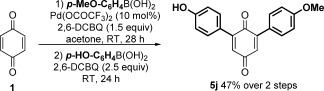
One-pot C=H hetero-difunctionalization of BQ.

Several mechanisms for the C=H functionalization of BQ are possible. Previously, we showed that the reaction with cyclohexenones under ligand-free conditions can be switched between oxidative Heck and conjugate addition in the final step of the cycle.[19b] Similarly, the C=H functionalization of BQ could proceed through 1) a direct Pd^II^-catalyzed oxidative Heck reaction, with the oxidant (BQ or 2,6-DCBQ) needed to reoxidize Pd^0^ to Pd^II^, or 2) conjugate addition to form functionalized hydroquinone, which is then oxidized in situ to the functionalized BQ product. Initial DFT calculations suggest that the regioselectivity of difunctionalization originates from the BQ insertion step rather than from charge or frontier orbital control.[28] The mechanisms of these processes will be the subject of future work.

In conclusion, we have developed the first efficient Pd-catalyzed direct C=H monofunctionalization of benzoquinone. Furthermore, an additional C=H functionalization to give difunctionalized products has been achieved, including the controlled installation of two different groups in a one-pot procedure, which is a major advancement in the field. Regioselectivities were found to be dependent on electronics on the aryl ring, and good selectivities were obtained for electron-rich and electron-deficient substrates. We believe that this new Pd-catalyzed method will allow rapid access to functionalized BQs that were previously difficult to synthesize.
