# Supplementary material for: Palladium-Catalyzed Direct C=H Functionalization of Benzoquinone
Source: Angew Chem Int Ed Engl. 2014 Oct 10;53(50):13876–9. doi: 10.1002/anie.201408054 (PMC4502976; doi:10.1002/anie.201408054)
Supplement: Supplementary file 1 [file anie0053-13876-sd1.pdf]

Angewandte Chemie
Eine Zeitschrift der Gesellschaft Deutscher Chemiker

Supporting Information
© Wiley-VCH 2014
69451 Weinheim, Germany

Palladium-Catalyzed Direct C–H Functionalization of Benzoquinone**

Sarah E. Walker, James A. Jordan-Hore, David G. Johnson, Stuart A. Macgregor, and Ai-Lan Lee**

anie_201408054_sm_miscellaneous_information.pdf

Supporting Information

Table of Contents

Experimental Supporting Information – Pages S3-S90

1. General Experimental Considerations S3

2. Preparation of Palladium Trifluoroacetate S4

3. Purification of Benzoquinone S4

4. General procedures for the palladium(II)-catalyzed C-H functionalization of benzoquinone S4

5. Diacetylation of 2,5- and 2,6-diaryl-1,4-hydroquinones S27

6. Reducing catalyst loading S29

7. One pot hetero-difunctionalization procedure S29

8. Control reactions S30

9. Crystal structures of difunctionalized products S31

10. Reducing the equivalents of BQ S36

11. Oxidant Screens S37

12. ^{1}H NMR and ^{13}C NMR Spectra S38

13. References S90

Computational Supporting Information – Pages S91-S166

1. Computational Details and References. S92

2. Computed Charge Distributions in **3** (R = *p*-$C_6H_4CF_3$ and *p*-C_6H_4OMe) S93

3. Orbital Distributions in the LUMOs of **3** (R = *p*-$C_6H_4CF_3$ and *p*-C_6H_4OMe) S94

4. Mechanistic Studies S95

    4.1 Product Stabilities S95

    4.2 Reaction profiles for migratory insertion. S96

5. Computed Cartesian Coordinates (Å) and Energies (au) for all species S102

Palladium(II)-Catalysed Direct CH-functionalization of Benzoquinone

Supplementary Information

1. General Experimental Considerations

^{1}H NMR spectra were recorded on Bruker AV 300, DPX 400 and AV 400 spectrometers at 300 and 400 MHz respectively and referenced to residual solvent. ^{13}C NMR spectra were recorded using the same spectrometers at 75 and 100 MHz respectively. Chemical shifts (δ in ppm) were referenced to tetramethylsilane (TMS) or to residual solvent peaks ($CDCl_3$ at δ_H 7.26, δ_C at 77.00 ppm, $(CD_3)_2CO$ at δ_H 2.05 ppm, δ_C at 29.84 ppm or $(CD_3)_2SO$ at δ_H 2.50 ppm, δ_C at 39.52 ppm). *J* values are given in Hz and s, d, dd, t, q, hept., m and app. abbreviations correspond to singlet, doublet, doublet of doublet, triplet, quartet, heptet, multiplet, apparent and combinations thereof. Mass spectra were obtained at the EPSRC National Mass Spectrometry Service Centre in Swansea. Infrared spectra were obtained on Perkin-Elmer Spectrum 100 FT-IR Universal ATR Sampling Accessory or Thermo Scientific Nicolet iS5 FT-IR spectrometer, deposited neat or as a chloroform solution to a diamond/ZnSe plate. Where necessary, sublimation of benzoquinone was carried out using a Kugelrohr distillation apparatus (Büchi B-585 or Büchi GKR-50).

Flash column chromatography was carried out using Matrix silica gel 60 from Fisher Chemicals and thin layer chromatography was performed using Merck silica gel 60 F254 precoated sheets and visualised by UV (254 nm) or stained by the use of aqueous acidic $KMnO_4$, aqueous acidic ceric ammonium molybdate, acidic dinitrophenyl hydrazine or molecular iodine as appropriate. Petroleum ether refers to petroleum ether (40–60 %) and EtOAc refers to ethyl acetate. Acetone was purchased from Fisher Scientific, all boronic acids were purchased from either Sigma Aldrich, Alfa Aesar or Fluorochem, palladium acetate trimer was provided by Johnson-Matthey and all other chemicals were provided by Sigma Aldrich. All chemicals were used without further purification unless otherwise stated. The reaction was performed without the need for dry solvents or inert atmosphere and all reactions were carried out in air.

2. Preparation of Palladium Trifluoroacetate[1]:

$Pd(OTFA)_2$ was prepared using the method specified in *Acta Cryst.* **1989**, *C45*, 1289 with minor modifications.

To a 100 mL round bottomed flask was added $Pd(OAc)_2$ (1.00 g, 4.45 mmol) and trifluoroacetic acid (25 mL). The resultant slurry was stirred for 10 min at 35 ^{o}C and the slurry was concentrated to dryness under reduced pressure. Trace TFA was removed *in vacuo*, to give $Pd(OTFA)_2$ as a brown dust (1.47 g, 99 %).

3. Purification of Benzoquinone:

Commercially available benzoquinone often appears either as an off yellow or green colour. This colouration is due to the presence of impurities, the most common being hydroquinone. Benzoquinone was purified prior to use by either simple flash column chromatography or recrystallisation from isopropyl alcohol. After purification benzoquinone was stored at room temperature without the exclusion of air. Pure benzoquinone should appear as a bright yellow, light and flocculent solid.

4. General procedures for the palladium(II)-catalysed C-H functionalization of benzoquinone

General procedure 1 - Palladium(II)-catalysed C-H monofunctionalization of benzoquinone in acetone:

Benzoquinone (3 equiv., 3 mmol), the boronic acid (1 equiv., 1 mmol) and palladium trifluoroacetate (7.5 mol%) were added to a round-bottomed flask equipped with a magnetic stir bar, acetone (12 mL) was then added and the reaction was stirred at room temperature for 18-24 h. Upon completion, as determined by thin layer chromatography, the mixture was evaporated to dryness, toluene (5 mL) and enough acetone to dissolve the heterogeneous mixture (0.5–1 mL) was added. The slurry was then purified directly by flash column chromatography to afford the monofunctionalized product. Where benzoquinone coeluted with the monofunctionalized product, it was removed by sublimation using a Kugelrohr distillation apparatus.

General procedure 2 - Palladium(II)-catalysed C-H monofunctionalization of benzoquinone in water:

Benzoquinone (3 equiv., 3 mmol), the boronic acid (1 equiv., 1 mmol) and palladium trifluoroacetate (7.5 mol%) were added to a round-bottomed flask equipped with a magnetic

stir bar, distilled water (12 mL) was then added, the solution briefly sonicated to disperse the reagents where necessary and the reaction was stirred at room temperature for 18-24 h. Upon completion, as determined by thin layer chromatography, EtOAc (20 mL) and water (10 mL) were added. The layers were separated and the aqueous layer was washed with a EtOAc (3 × 20 mL). The organic layer was then washed with brine (20 mL), dried over $MgSO_4$ and concentrated under reduced pressure. To the resultant solid, toluene (5 mL) and enough acetone to dissolve the heterogeneous mixture (0.5–1 mL) was added. The slurry was then purified directly by flash column chromatography to afford the monofunctionalized product. Where benzoquinone coeluted with the monofunctionalized product, it was removed by sublimation using a Kugelrohr distillation apparatus.

Where monofunctionalized products have been synthesised using both procedures 1 and 2, the procedure which gives the highest yield is that which is mentioned with the characterisation data for each compound.

General procedure 3 - Palladium(II)-catalysed C-H monofunctionalization of benzoquinone using cycloalkyl boronic acids:

Benzoquinone (3 equiv., 1.5 mmol), the corresponding boronic acid (1 equiv., 0.5 mmol) and $Pd(OTFA)_2$ (7.5 mol%) were added to a 5 mL round bottomed flask with stirrer bar. Acetone (3.3 mL) was added and the reaction was stirred at 40 °C for 24 h after which a further portion of $Pd(OTFA)_2$ (7.5 mol%) was added and the reaction stirred for another 24 h. Upon completion, as determined by thin layer chromatography, the mixture was evaporated to dryness, toluene (5 mL) and enough acetone to dissolve the heterogeneous mixture (0.5–1 mL) was added. The slurry was then purified directly by flash column chromatography to afford the monofunctionalized product.

Cyclobutyl and cyclopentyl boronic acid were found to be unstable in air (but stable once in solution) and were therefore weighed out under a nitrogen atmosphere in a glove box.

The products formed using cyclobutyl and cyclopentyl boronic acids (compounds **3t** and **3s**) were found to be unstable when subjected to very low pressure. In these cases, after column chromatography, the majority of solvent was removed using the rotary evaporator to form a concentrated solution and the remaining solvent evaporated by placing the solution under a steady flow of nitrogen.

General procedure 4 - Palladium(II)-catalysed homo-difunctionalization of benzoquinone:

Benzoquinone (1 equiv., 0.1 mmol), the boronic acid (2.5 equiv., 0.25 mmol), 2,6-dichlorobenzoquinone (2.5 equiv., 0.25 mmol) and palladium trifluoroacetate (10 mol%) were added to a round bottomed flask equipped with a magnetic stir bar. Acetone (0.340 mL) was then added and the reaction was stirred at room temperature for 48 h. Upon completion, as determined by thin layer chromatography, the mixture was evaporated to dryness, toluene (5 mL) and enough acetone to dissolve the heterogeneous mixture (0.5–1 mL) was added. The slurry was then purified directly by flash column chromatography to afford the difunctionalized product.

General procedure 5 - Palladium(II)-catalysed Hetero-difunctionalization of monofunctionalized benzoquinone derivatives:

The monofunctionalized benzoquinone derivative (1 equiv., 0.05 mmol), the boronic acid (1.25 equiv., 0.0625 mmol), 2,6-dichloro-1,4-benzoquinone (1.25 equiv., 0.0625 mmol) and palladium trifluoroacetate (10 mol%) were added to a round bottomed flask equipped with a magnetic stir bar. Acetone (0.170 mL) was then added and the reaction was stirred at 20 °C for 18 h. Upon completion, as determined by thin layer chromatography, the mixture was evaporated to dryness, toluene (5 mL) and enough acetone to dissolve the heterogeneous mixture (0.5–1 mL) was added. The slurry was then purified directly by flash column chromatography to afford the hetero-difunctionalized product.

2-(4-Methoxyphenyl)-1,4-benzoquinone (3a)[2]

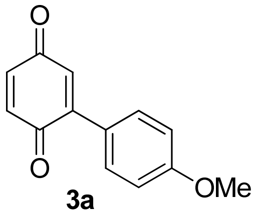

3a

General procedure 2 was followed to give the product **3a** in 88% yield as a brown solid; M. p. 108-110 °C; R_f 0.68 (3:1 hexane:EtOAc); ^{1}H NMR (300 MHz, $CDCl_3$): δ = 7.41 (d, J = 8.8 Hz, 2H, Ar-H), 6.90 (d, J = 8.8 Hz, 2H, Ar-H), 6.82 – 6.69 (m, 3H, 3 × O=C-C<u>H</u>), 3.79 (s, 3H, OC<u>H₃</u>); ^{13}C NMR (75 MHz, $CDCl_3$): δ = 187.7 (C), 187.1 (C), 161.4 (C), 145.2 (C), 137.0 (CH), 136.3 (CH), 131.1 (CH), 130.9 (CH), 125.0 (C), 114.1 (CH), 55.4 (CH_3); HRMS (APCI) calculated for $[M+H]^+$ 215.0703, $C_{13}H_{11}O_3$ found: 215.0709.

2-(4-Hydroxyphenyl)-1,4-benzoquinone (3b)[3]

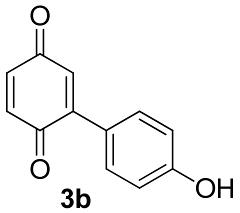

3b

General procedure 1 was followed, using 6 equiv. benzoquinone to give the product **3b** in 64% yield as a bright red solid; M. p. 173-176 °C; R_f 0.56 (2:1 petroleum ether:EtOAc); ^{1}H NMR (300 MHz, Acetone-d_6): δ = 8.84 (s, 1H, OH), 7.48 (d, J = 8.7 Hz, 2H, Ar-H), 6.92 (d, J = 8.7 Hz, 2H, Ar-H), 6.87 – 6.79 (m, 3H, 3 × O=C-CH); ^{13}C NMR (75 MHz, Acetone-d_6): δ = 188.3 (C), 187.9 (C), 160.2 (C), 146.0 (C), 138.0 (CH), 136.9 (CH), 132.0 (CH), 131.9 (CH), 125.2 (C), 116.2 (CH); IR: ῡ = 3317 br str, 1646 v str, 1607 str, 1514 m, 1434 w, 1343 w, 1252 m, 1098 w, 978 w, 900 v str, 840 v str; HRMS (APCI) calculated for $[M+H]^+$ 201.0546, $C_{12}H_9O_3$ found: 201.0546.

2-(3,4-Dimethoxyphenyl)-1,4-benzoquinone (3c)[2]

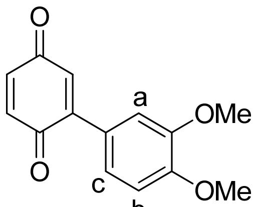

3c

General procedure 2 was followed on gram scale (10.5 mmol, 1.136 g benzoquinone) to give the product **3c** in 70% yield as a black solid; M. p. 137-140 °C; R_f 0.43 (2:1 hexane:EtOAc); ^{1}H NMR (300 MHz, $CDCl_3$): δ = 7.14 (dd, J = 8.4, 2.1 Hz, 1H, H_c), 7.05 (d, J = 2.1 Hz, 1H, H_a), 6.94 (d, J = 8.4 Hz, 1H, H_b), 6.86 – 6.82 (m, 3H, 3 × O=C-CH), 3.93 (s, 3H, OCH_3), 3.92 (s, 3H, OCH_3); ^{13}C NMR (101 MHz, $CDCl_3$): δ = 187.6 (C), 187.0 (C), 151.1 (C), 148.9 (C), 145.2 (C), 137.1 (CH), 1362 (CH), 131.3 (CH), 125.2 (C), 122.7 (CH), 112.18 (CH), 111.1 (CH), 56.01 (CH_3), 55.99 (CH_3); IR: ῡ = 1652 v str, 1515 str, 1263 str, 1146 m, 1093 m, 1023 m, 901 w, 860 w.

2-(3-Methylphenyl)-1,4-benzoquinone (3d)[2]

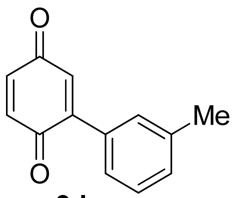

3d

General procedure 2 was followed to give the product **3d** in 90% yield as a brown solid; M. p. 84-87 °C; R_f 0.45 (10:1 petroleum ether:EtOAc); ^{1}H NMR (300 MHz, $CDCl_3$): δ = 7.49 – 7.00 (m, 4H, Ar-H), 6.92 – 6.61 (m, 3H, 3 × O=C-CH), 2.34 (s, 3H, CH_3); ^{13}C NMR (101 MHz, $CDCl_3$): δ = 187.6 (C), 186.7 (C), 146.2 (C), 138.2 (C), 137.1 (CH), 136.2 (CH), 132.62 (C), 132.56 (CH), 130.9 (CH), 129.8 (CH), 128.4 (CH), 126.4 (CH), 21.4 (CH_3); IR: ῡ = 2922 w, 2856 str, 1643 v str, 1602 m, 1590 str, 189 w, 1296 str, 1098 str, 900 str, 888 str, 781 v str, 697 v str; HRMS (APCI) calculated for $[M+H]^+$ 199.0754, $C_{13}H_{11}O_2$ found: 199.0753.

2-(3-Chloro-4-methoxyphenyl)-1,4-benzoquinone (3e)

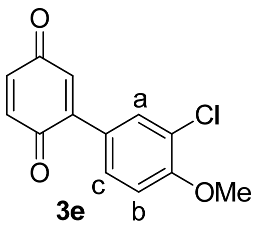

General procedure 1 was followed to give the product **3e** in 78% yield as a red solid; M. p. 160-162 °C; R_f 0.20 (5:1 hexane:EtOAc); ^{1}H NMR (400 MHz, $CDCl_3$): δ = 7.57 (d, J = 2.2 Hz, 1H, H_a), 7.43 (dd, J = 8.6, 2.2 Hz, 1H, H_c), 6.99 (d, J = 8.6 Hz, 1H, H_b), 6.88 – 6.78 (m, 3H, 3 × O=C-C$\underline{H}$), 3.96 (s, 3H, OC$\underline{H_3}$); ^{13}C NMR (101 MHz, $CDCl_3$): δ = 187.4 (C), 186.6 (C), 156.6 (C), 144.0 (C), 137.0 (CH), 136.3 (CH), 131.7 (CH), 131.0 (CH), 129.1 (CH), 125.7 (C), 122.9 (C), 111.8 (CH), 56.3 (CH_3); IR: ṽ = 3062 w, 2954 w, 1653 v str, 1599 str, 1588 str, 1505 str, 1303 str, 1266 str, 1064 str; HRMS (APCI) calculated for $[M+H]^+$ 249.0313, $C_{13}H_{10}O_3Cl$ found: 249.0312.

2-(4-Trifluoromethylphenyl)-1,4-benzoquinone (3f)[2]

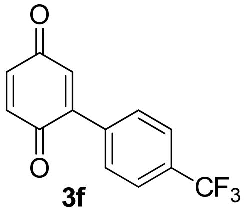

General procedure 2 was followed to give the product **3f** in 64% yield as a black solid; M. p. 109-113 °C; R_f 0.17 (5:1 hexane:EtOAc); ^{1}H NMR (400 MHz, $CDCl_3$): δ = 7.71 (d, J = 8.1 Hz, 2H, Ar-H), 7.59 (d, J = 8.1 Hz, 2H, Ar-H), 6.93 – 6.85 (m, 3H, 3 × O=C-C$\underline{H}$); ^{13}C NMR (101 MHz, $CDCl_3$): δ = 187.1 (C), 186.0 (C), 144.7 (C), 137.0 (CH), 136.4 (CH), 136.1 (C), 133.6 (CH), 131.9 (C, q, J = 32.8 Hz), 129.6 (CH), 125.5 (CH, q, J = 3.7 Hz), 123.77 (C, q, J = 272.4 Hz); IR: ṽ = 2930 w, 1649 v str, 1597 m , 1406 m, 1329 str, 1120 v str, 1107 v str, 1069 v str, 908 v str, 860 str, 848 str.

2-(4-Fluorophenyl)-1,4-benzoquinone (3g)[2]

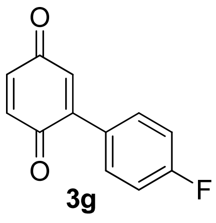

General procedure 2 was followed to give the product **3g** in 80% yield (with ~5% benzoquinone impurity) as a green solid; M. p. 152-155 °C; R_f 0.38 (5:1 hexane:EtOAc); ^{1}H NMR (300 MHz, $CDCl_3$): δ = 7.56 – 7.43 (m, 2H, Ar-H), 7.21 – 7.08 (m, 2H, Ar-H), 6.93 – 6.73 (m, 3H, 3 × O=C-C$\underline{H}$); ^{13}C NMR (101 MHz, $CDCl_3$): δ = 187.4 (C), 186.5 (C), 164.0 (d, J = 250.8 Hz, C-F), 144.8 (C), 137.0 (CH), 136.3 (CH), 132.4 (CH), 131.3 (d, J = 8.5 Hz, CH), 128.64 (d, J = 3.5 Hz, C), 115.76 (d, J = 21.8 Hz, CH); HRMS (APCI) calculated for $[M+H]^+$ 203.0503, $C_{12}H_8O_2F$ found: 203.0502.

2-(4-Bromophenyl)-1,4-benzoquinone (3h)[4]

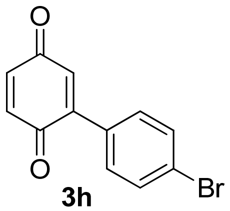

3h

General procedure 2 was followed to give the product **3h** in 82% yield as a bright yellow solid; M. p. 112-115 °C; R_f 0.38 (5:1 hexane:EtOAc); ^{1}H NMR (300 MHz, $CDCl_3$): δ = 7.58 (d, J = 8.5 Hz, 2H, Ar-H), 7.36 (d, J = 8.5 Hz, 2H, Ar-H), 6.92 – 6.79 (m, 3H, 3 × O=C-C<u>H</u>); ^{13}C NMR (101 MHz, $CDCl_3$): δ = 187.3 (C), 186.2 (C), 144.8 (C), 137.0 (CH), 136.4 (CH), 132.6 (CH), 131.8 (CH), 131.4 (C), 130.8 (CH), 124.9 (C); HRMS (APCI) calculated for $[M+H]^+$ 262.9702, $C_{12}H_8O_2Br$ found: 262.9700.

2-(3-Chlorophenyl)-1,4-benzoquinone (3i)[5]

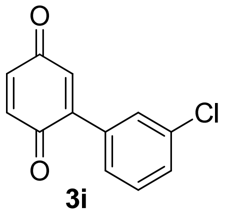

3i

General procedure 2 was followed to give the product **3i** in 89% yield as a dark yellow solid; M. p. 142-144 °C; R_f 0.38 (5:1 hexane:EtOAc); ^{1}H NMR (300 MHz, $CDCl_3$): δ = 7.51 – 7.47 (m, 1H, Ar-H), 7.47 – 7.41 (m, 1H, Ar-H), 7.41 – 7.33 (m, 2H, Ar-H), 6.95 – 6.73 (m, 3H, 3 × O=C-C<u>H</u>); ^{13}C NMR (101 MHz, $CDCl_3$): δ = 187.2 (C), 186.1 (C), 144.6 (C), 137.0 (CH), 136.4 (CH), 134.5 (C), 134.3 (C), 133.1 (CH), 130.1 (CH), 129.8 (CH), 129.3 (CH), 127.4 (CH); IR: ῦ = 3077 w, 1654 v str, 1590 str, 1562 str, 1344 str, 1299 str, 1099 str, 1085 str, 903 v str, 883 v str, 833 v str, 690 v str; HRMS (APCI) calculated for $[M+H]^+$ 219.0207, $C_{12}H_8O_2Cl$ found: 219.0213.

2-(4-Ethoxycarbonylphenyl)-1,4-benzoquinone (3j)

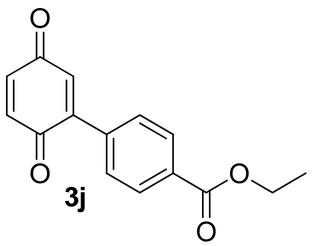

3j

General procedure 2 was followed to give the product **3j** in 75% yield as a dark green solid; M. p. 119-123 °C; R_f 0.57 (2:1 hexane:EtOAc); ^{1}H NMR (300 MHz, $CDCl_3$): δ = 8.12 (d, J = 8.7 Hz, 2H, Ar-H), 7.55 (d, J = 8.7 Hz, 2H, Ar-H), 6.94 – 6.85 (m, 3H, 3 × O=C-C<u>H</u>), 4.41 (q, J = 7.1 Hz, 2H, C<u>H$_2$</u>CH_3), 1.41 (t, J = 7.1 Hz, 3H, CH_2C<u>H$_3$</u>); ^{13}C NMR (101 MHz, $CDCl_3$): δ = 187.2 (C), 186.1 (C), 165.9 (C), 145.1 (C), 137.0 (CH), 136.8 (C), 136.4 (CH), 133.4 (CH), 131.8 (C), 129.6 (CH), 129.2 (CH), 61.3 (CH_2), 14.3 (CH_3); IR: ῦ = 2929 w, 1716 w, 1651 v str, 1596 str, 1407 str, 1326 v str, 1278 str, 1168 str, 1120 v str, 1068 v str, 908 v str, 732 v str; HRMS (APCI) calculated for $[M+H]^+$ 257.0808, $C_{15}H_{13}O_4$ found: 257.0811.

2-(3-Nitrophenyl)-1,4-benzoquinone (3k)[6]

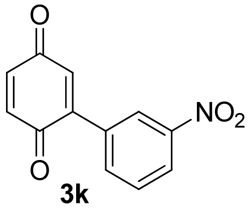

General procedure 1 was followed, but the reaction required 48 h at 50 °C to give the product **3k** in 71% yield as a grey solid; M. p. 118-125 °C; R_f 0.14 (5:1 hexane:EtOAc); ^{1}H NMR (300 MHz, $CDCl_3$): δ = 8.37 (t, *J* = 1.9 Hz, 1H, Ar-H), 8.36 – 8.30 (m, 1H, Ar-H), 7.85 – 7.79 (m, 1H, Ar-H), 7.69 – 7.62 (m, 1H, Ar-H), 6.98 – 6.90 (m, 3H, 3 × O=C-C$\underline{H}$); ^{13}C NMR (75 MHz, $CDCl_3$): δ = 186.8 (C), 185.7 (C), 148.3 (C), 143.7 (C), 136.9 (CH), 136.6 (CH), 135.1 (CH), 134.1 (C), 133.8 (CH), 129.6 (CH), 124.7 (CH), 124.3 (CH); IR: ῦ = 3084 w, 2925 w, 1658 v str, 1614 str, 1592 str, 1528 v str, 1345 v str, 1295 str, 1275 str, 1094 v str, 902 v str, 733 v str; HRMS (APCI) calculated for $[M+H]^+$ 230.0448, $C_{12}H_8O_4N$ found: 230.0450.

2-(2,4,6-Trimethylphenyl)-1,4-benzoquinone (3l)

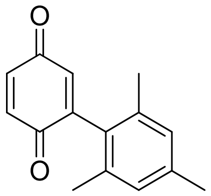

General procedure 2 was followed to give the product **3l** in 68% yield as a dark red thick oil; R_f 0.43 (10:1 hexane:EtOAc); ^{1}H NMR (300 MHz, $CDCl_3$): δ = 6.93 (s, 2H, Ar-H), 6.90 – 6.64 (m, 3H, 3 × O=C-C$\underline{H}$), 2.31 (s, 3H, C$\underline{H_3}$), 2.08 (s, 6H, 2 × C$\underline{H_3}$); ^{13}C NMR (75 MHz, $CDCl_3$): δ = 187.4 (C), 186.2 (C), 148.1 (C), 138.4 (C), 136.7 (CH), 136.4 (CH), 135.4 (CH), 135.3 (C), 129.7 (C), 128.3 (CH), 21.0 (CH_3), 20.2 (CH_3); IR: ῦ = 2921 w, 1656 v str, 1611 w, 1597 w, 1282 str, 1090 m, 912 m, 835 m; HRMS (APCI) calculated for $[M+H]^+$ 227.1067, $C_{15}H_{15}O_2$ found: 227.1067.

2-(2-Fluorenyl)-1,4-benzoquinone (3m)

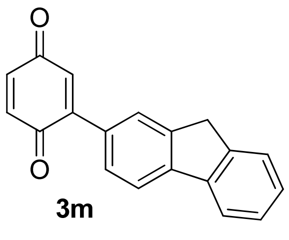

General procedure 1 was followed to give the product **3m** in 81% yield as a brown solid; M. p. 195-198 °C; R_f 0.59 (3:1 petroleum ether:EtOAc); ^{1}H NMR (300 MHz, $CDCl_3$): δ = 7.92 – 7.77 (m, 2H, Ar-H), 7.74 – 7.65 (m, 1H, Ar-H), 7.63 – 7.46 (m, 2H, Ar-H), 7.46 – 7.31 (m, 2H, Ar-H), 6.98 – 6.80 (m, 3H, 3 × O=C-C$\underline{H}$), 3.96 (s, 2H, C$\underline{H_2}$); ^{13}C NMR (75 MHz, $CDCl_3$): δ = 187.6 (C), 187.0 (C), 146.1 (C), 143.8 (C), 143.8 (C), 143.4 (C), 140.7 (C), 137.1 (CH), 136.3 (CH), 132.1 (CH), 130.9 (C), 128.2 (CH), 127.6 (CH), 127.0 (CH), 126.0 (CH), 125.2 (CH), 120.5 (CH), 119.9 (CH), 36.9 (CH_2); IR: ῦ = 3053 w, 2924 w, 1645 v str, 1589 str, 1456 w, 767 str, 732 v str; HRMS (APCI) calculated for $[M+H]^+$ 273.0910, $C_{19}H_{13}O_2$ found: 273.0907.

C-H Monofunctionalization of benzoquinone with heterocyclic boronic acids: General procedures 1 and 2 used. Reactions carried out on a 0.5 mmol scale with a reaction time of 40-43 h.

2-(3-Furanyl)-1,4-benzoquinone (3n)

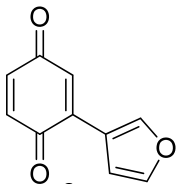

3n

General procedure 1 was followed to give the product **3n** in 52% yield as a dark brown solid; M. p. decomposes at 110 °C; R_f 0.59 (2:1 hexane:EtOAc); ^{1}H NMR (300 MHz, $CDCl_3$): δ = 8.35 (s, 1H, HetAr-H), 7.48 (dd, J = 2.0, 1.5 Hz, 1H, HetAr-H), 6.87 – 6.72 (m, 3H, 3 × O=C-C$\underline{H}$), 6.67 – 6.61 (m, 1H, HetAr-H); ^{13}C NMR (101 MHz, $CDCl_3$): δ = 187.4 (C), 186.4 (C), 146.1 (CH), 143.6 (CH), 138.0 (C), 137.1 (CH), 136.2 (CH), 128.5 (CH), 118.0 (C), 107.7 (CH); IR: ῦ = 2973 w, 1651 v str, 1597 str, 1296 str, 1164 m, 1033 str, 913 str, 806 str; HRMS (APCI) calculated for $[M+H]^+$ 175.0390, $C_{10}H_7O_3$ found: 175.0390.

2-(3-Thienyl)-1,4-benzoquinone (3o)

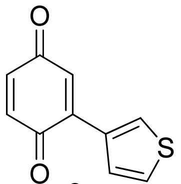

3o

General procedure 1 was followed to give the product **3o** in 62% yield as a brown solid; M. p. 137-139 °C; R_f 0.50 (2:1 hexane:EtOAc); ^{1}H NMR (300 MHz, $CDCl_3$): δ = 8.12 (dd, J = 2.6, 1.6 Hz, 1H, HetAr-H), 7.43 – 7.35 (m, 2H, HetAr-H), 6.96 – 6.76 (m, 3H, 3 × O=C-C$\underline{H}$); ^{13}C NMR (101 MHz, $CDCl_3$): δ = 187.9 (C), 186.8 (C), 139.2 (C), 137.1 (CH), 136.1 (CH), 132.8 (C), 129.9 (CH), 129.8 (CH), 126.7 (CH), 126.2 (CH); IR: ῦ = 3054 w, 1659 v str, 1646 v str, 1578 v str, 1510 m, 1419 m, 1371 m, 1286 v str, 1096 v str, 907 v str, 789 v str; HRMS (APCI) calculated for $[M+H]^+$ 191.0161, $C_{10}H_7O_2S$ found: 191.0166.

2-(1-(Triisopropylsilyl)-3-pyrrolyl)-1,4-benzoquinone (3p)

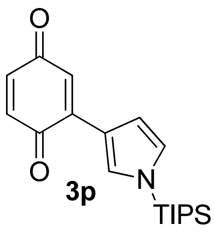

3p

General procedure 1 was followed to give the product **3p** in 41% yield as a dark red solid; M. p. 75-78 °C; R_f 0.60 (5:1 hexane:EtOAc); ^{1}H NMR (300 MHz, $CDCl_3$): δ = 7.73 (dd, J = 2.0, 1.4 Hz, 1H, HetAr-H), 6.84 – 6.68 (m, 4H, 3 × O=C-C$\underline{H}$ and HetAr-H), 6.61 (dd, J = 3.0, 1.4 Hz, 1H, HetAr-H), 1.49 (hept., J = 7.5 Hz, 3H, C$\underline{H}$(CH_3)$_2$), 1.12 (d, J = 7.5 Hz, 18H, Si(CH(C$\underline{H}_3$)$_2$)$_3$); ^{13}C NMR (75 MHz, $CDCl_3$): δ = 188.1 (C), 188.0 (C), 140.1 (C), 136.9 (CH), 136.2 (CH), 129.9 (CH), 125.7 (CH), 125.3 (CH), 118.3 (C), 109.3 (CH), 17.7 (CH_3), 11.6 (CH); IR: ῦ = 2950 str, 2866 str, 1663 v str, 1650 v str, 1573 v str, 1495 str, 1285 v str,

1221 v str, 1082 v str, 884 v str, 658 v str; HRMS (NSI) calculated for $[M+H]^+$ 330.1884, $C_{19}H_{28}O_2NSi$ found: 330.1886.

2-(1-(*tert*-Butoxycarbonyl)-2-pyrrolyl)-1,4-benzoquinone (3q)

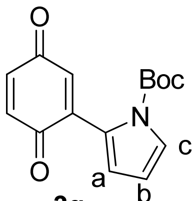

3q

General procedure 2 was followed to give the product **3q** in 75% yield as a dark red thick oil; R_f 0.66 (2:1 hexane:EtOAc); ^{1}H NMR (300 MHz, $CDCl_3$): δ = 7.41 (dd, J = 3.4, 1.7 Hz, 1H, H_c), 6.86 – 6.70 (m, 3H, 3 × O=C-C<u>H</u>), 6.37 (dd, J = 3.4, 1.7 Hz, 1H, H_a), 6.25 (t, J = 3.4 Hz, 1H, H_b), 1.51 (s, 9H, O^t<u>Bu</u>); ^{13}C NMR (75 MHz, $CDCl_3$): δ = 187.5 (C), 185.8 (C), 148.6 (C), 141.7 (C), 136.7 (CH), 136.6 (CH), 130.1 (CH), 126.6 (C), 124.9 (CH), 117.0 (CH), 111.2 (CH), 84.7 (C), 27.8 (CH_3); IR: ῦ = 2981 w, 1744 v str, 1661 v str, 1595 w, 1468 w, 1404 w, 1314 v str, 1139 v str; HRMS (APCI) calculated for $[M+H]^+$ 274.1074, $C_{15}H_{16}O_4N$ found: 274.1070.

2-(Cyclohexyl)-1,4-benzoquinone (3r)[2]

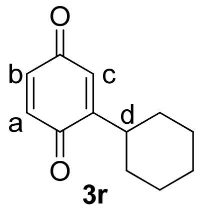

3r

General procedure 3 for cycloalkyl boronic acids was followed to give the product **3r** in 63% yield as a yellow amorphous solid; R_f 0.27 (5:1 hexane:EtOAc); ^{1}H NMR (300 MHz, $CDCl_3$): δ = 6.75 (d, J = 10.2 Hz, 1H, H_a), 6.68 (dd, J = 10.2, 2.4 Hz, 1H, H_b), 6.49 (dd, J = 2.4, 1.1 Hz, 1H, H_c), 2.77 – 2.56 (m, 1H, H_d), 1.89 – 1.64 (m, 5H, C<u>H2</u>), 1.51 – 1.28 (m, 2H, C<u>H2</u>), 1.28 – 1.03 (m, 3H, C<u>H2</u>); ^{13}C NMR (101 MHz, $CDCl_3$): δ = 188.2 (C), 187.1 (C), 154.0 (C), 137.0 (CH), 135.9 (CH), 130.7 (CH), 36.3 (CH), 32.0 (CH_2), 26.3 (CH_2), 25.9 (CH_2); IR: ῦ = 2927 str, 2854 str, 1657 v str, 1598 str, 1449 w; HRMS (APCI) calculated for $[M+H]^+$ 191.1067, $C_{12}H_{15}O_2$ found: 191.1063.

2-(Cyclopentyl)-1,4-benzoquinone (3s)[2]

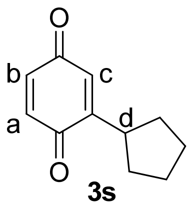

3s

General procedure 3 for cycloalkyl boronic acids was followed to give the product **3s** in 92% yield as a dark brown thick oil; R_f 0.27 (5:1 hexane:EtOAc); ^{1}H NMR (300 MHz, $CDCl_3$): δ = 6.75 (d, J = 10.0 Hz, 1H, H_a), 6.70 (dd, J = 10.0, 2.3 Hz, 1H, H_b), 6.56 (dd, J = 2.3, 1.3 Hz, 1H, H_c), 3.14 – 2.97 (m, 1H, H_d), 2.08 – 1.86 (m, 2H, C<u>H2</u>), 1.85 – 1.61 (m, 4H, C<u>H2</u>), 1.53 – 1.32 (m, 2H, C<u>H2</u>); ^{13}C NMR (75 MHz, $CDCl_3$): δ = 188.1 (C), 187.6 (C), 153.2 (C), 137.0 (CH), 136.0 (CH), 130.1 (CH), 38.7 (CH), 32.0 (CH_2), 25.2 (CH_2); HRMS (APCI) calculated for $[M+H]^+$ 177.0910, $C_{11}H_{13}O_2$ found: 177.0906.

2-(Cyclobutyl)-1,4-benzoquinone (3t)

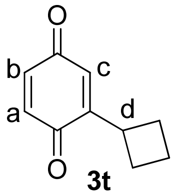

3t

General procedure 3 for cycloalkyl boronic acids was followed, except that the second portion of catalyst was added after 19 h, to give the product **3t** in 67% yield as a yellow amorphous solid; R_f 0.22 (10:1 petroleum ether:diethyl ether); ^{1}H NMR (300 MHz, $CDCl_3$): δ = 6.72 – 6.70 (m, 2H, H_a and H_b), 6.56 – 6.53 (m, 1H, H_c), 3.61 – 3.42 (m, 1H, H_d), 2.37 – 2.21 (m, 2H, C$\underline{H_2}$), 2.13 – 1.90 (m, 3H, C$\underline{H_2}$), 1.90 – 1.78 (m, 1H, C$\underline{H_2}$); ^{13}C NMR (101 MHz, $CDCl_3$): δ = 188.1 (C), 187.4 (C), 152.2 (C), 136.8 (CH), 136.2 (CH), 130.3 (CH), 34.6 (CH), 27.9 (CH_2), 18.5 (CH_2); IR: $\tilde{\upsilon}$ = 2923 str, 2849 w, 1644 v str, 1592 m, 1306 m, 1085 m, 891 str, 575 str; HRMS (APCI) calculated for $[M+H]^+$ 163.0754, $C_{10}H_{11}O_2$ found: 163.0753.

2-(Cyclohexen-1-yl)-1,4-benzoquinone (3u)

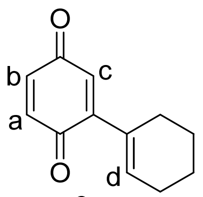

3u

General procedure 1 was followed on a 1.50 mmol scale to give the product **3u** in 84% yield as a red oil. The product decomposes readily and was therefore isolated as quickly as possible. R_f 0.47 (5:1 hexane:EtOAc); ^{1}H NMR (400 MHz, $CDCl_3$): δ = 6.72 (dd, J = 7.8, 0.3 Hz, 1H, H_a), 6.69 (dd, J = 7.8, 1.5 Hz, 1H, H_b), 6.54 (dd, J = 1.5, 0.3 Hz, 1H, H_c), 6.50 – 6.45 (m, 1H, H_d), 2.32 – 2.16 (m, 4H, C$\underline{H_2}$), 1.79 – 1.69 (m, 2H, C$\underline{H_2}$), 1.69 – 1.60 (m, 2H, C$\underline{H_2}$); ^{13}C NMR (101 MHz, $CDCl_3$): δ = 188.2 (C), 187.4 (C), 146.9 (C), 137.7 (CH), 136.6 (CH), 135.6 (CH), 131.2 (C), 129.1 (CH), 27.0 (CH_2), 26.4 (CH_2), 22.4 (CH_2), 21.5 (CH_2); IR: $\tilde{\upsilon}$ = 2930 str, 2860 str, 1649 v str, 1608 str, 1566 str, 1449 w, 1280 str, 1090 str, 901 str, 861 m, 731 w; HRMS (APCI) calculated for $[M+H]^+$ 189.0910, $C_{12}H_{13}O_2$ found: 189.0906.

Palladium(II)-catalysed homo-difunctionalization of benzoquinone

2,6-Bis-(4-hydroxyphenyl)-1,4-benzoquinone (5a) and **2,5-Bis-(4-hydroxyphenyl)-1,4-benzoquinone (4a)**

General procedure 4 was followed to yield **5a** and **4a** in an approximate >10:1 ratio.

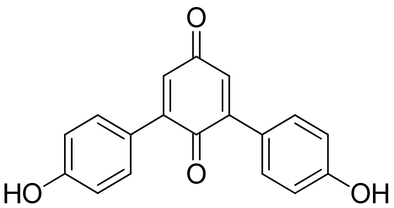

Major product
5a

Dark red solid, 71% yield; M. p. 211-215 °C; R_f 0.39 (1:1 hexane:EtOAc); ^{1}H NMR (300 MHz, Acetone-d_6): δ = 8.82 (s, 2H, O$\underline{H}$), 7.52 (d, J = 8.8 Hz, 4H, Ar-H), 6.93 (d, J = 8.8 Hz, 4H, Ar-H), 6.82 (s, 2H, O=C-C$\underline{H}$); ^{13}C NMR (75 MHz, Acetone-d_6): δ = 188.01 (C), 187.8 (C), 160.1 (C), 147.1 (C),

132.1 (CH), 131.0 (CH), 125.9 (C), 116.1 (CH); IR: ῡ = 3318 br str, 1637 v str, 1605 v str, 1579 v str, 1506 v str, 1438 str, 1232 v str, 1176 v str, 1105 v str, 910 str, 840 v str, 730 str; HRMS (APCI) calculated for $[M+H]^+$ 293.0808, $C_{18}H_{13}O_4$ found: 293.0810.
A crystal structure was also obtained of this product. This can be found on page S33.

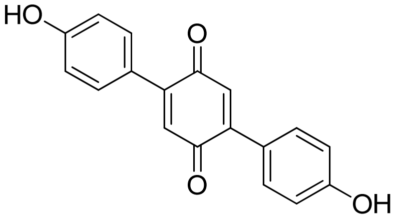

Minor product
4a

Black solid, <5% yield; M. p. >300 °C; R_f 0.39 (1:1 hexane:EtOAc); ^{1}H NMR (300 MHz, DMSO-d_6): δ = 10.00 (s, 2H, O<u>H</u>), 7.47 (d, J = 8.8 Hz, 4H, Ar-H), 6.90 (s, 2H, O=C-C<u>H</u>), 6.84 (d, J = 8.8 Hz, 4H, Ar-H); ^{13}C NMR (75 MHz, DMSO-d_6): δ = 187.3 (C), 159.4 (C), 144.2 (C), 131.2 (CH), 130.7 (CH), 123.2 (C), 115.4 (CH); IR: ῡ = 3379 br, 2567 m, 2506 m, 1637 v str, 1594 v str, 1507 v str, 1347 m, 1250 v str, 1171 str, 992 m, 899 str, 805 str; HRMS (APCI) calculated for $[M-H]^-$ 291.0663, $C_{18}H_{11}O_4$ found: 291.0658.

2,6-Bis-(4-hydroxy-3-methoxyphenyl)-1,4-benzoquinone (5b)

General procedure 4 was followed to yield **5b** as the major product. Evidence of a minor product, believed to be the 2,5 isomer, was observed. However, it was impossible to isolate pure for characterisation.

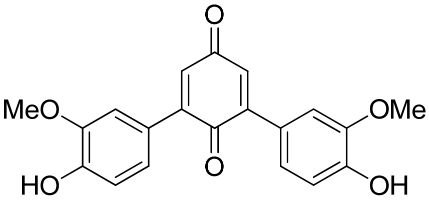

5b

Dark red solid, 73% yield; M. p. 178-179 °C; R_f 0.66 (1:1.5 hexane:EtOAc); ^{1}H NMR (300 MHz, $CDCl_3$): δ = 7.16 – 7.05 (m, 4H, Ar-H), 6.99 (d, J = 8.4 Hz, 2H, Ar-H), 6.86 (s, 2H, O=C-C<u>H</u>), 5.87 (s, 2H, OH), 3.94 (s, 6H, OC<u>H$_3$</u>); ^{13}C NMR (75 MHz, $CDCl_3$): δ = 187.6 (C), 187.0 (C), 147.7 (C), 146.4 (C), 145.9 (C), 131.3 (CH), 125.3 (C), 123.3 (CH), 114.6 (CH), 111.9 (CH), 56.1 (CH_3); IR: ῡ = 3287 br, 2938 w, 1631 m, 1586 m, 1563 m, 1509 str, 1426 str, 1259 str, 899 str, 855 m; HRMS (APCI) calculated for $[M+H]^+$ 353.1020, $C_{20}H_{17}O_6$ found: 353.1016.

2,6-Bis-(3,4-dimethoxyphenyl)-1,4-benzoquinone (5c)[7]

General procedure 4 was followed to yield **5c** as the major product. Evidence of a minor product, believed to be the 2,5 isomer was observed. However, it was impossible to isolate pure for characterisation.

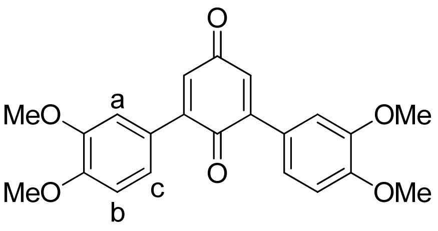

Major product
5c

Dark red solid, 58% yield; M. p. 160-164 °C; R_f 0.47 (95:5 dichloromethane:EtOAc); ^{1}H NMR (300 MHz, $CDCl_3$): δ = 7.15 (dd, J = 8.4, 2.1 Hz, 2H, H_c), 7.09 (d, J = 2.1 Hz, 2H, H_a), 6.95 (d, J = 8.4 Hz, 2H, H_b), 6.87 (s, 2H, O=C-CH), 3.93 (s, 6H, OCH_3); 3.92 (s, 6H, OCH_3); ^{13}C NMR (75 MHz, $CDCl_3$): δ = 187.6 (C), 186.8 (C), 150.9 (C), 148.8 (C), 145.8 (C), 131.4 (CH), 125.8 (C), 122.8 (CH), 112.3 (CH), 111.0 (CH), 56.00 (CH_3), 55.97 (CH_3); IR: ῡ = 3018 w, 2936 w, 2838 w, 1640 v str, 1596 str, 1510 v str, 1463 w, 1255 v str, 1145 m, 1021 w, 746 v str; HRMS (APCI) calculated for $[M+H]^+$ 381.1333, $C_{22}H_{21}O_6$ found: 381.1334.

2,6-Bis-(3-methylphenyl)-1,4-benzoquinone (5d)[8] and **2,5-Bis-(3-methylphenyl)-1,4-benzoquinone (4d)[8]**

General procedure 4 was followed to yield **5d** and **4d** in an approximate 2:1 ratio.

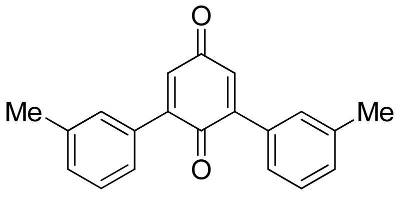

Major product
5d

Dark brown thick oil, 53% yield; R_f 0.83 (4:1 petroleum ether:EtOAc); ^{1}H NMR (300 MHz, $CDCl_3$): δ = 7.34 – 7.14 (m, 8H, Ar-H), 6.83 (s, 2H, O=C-CH), 2.34 (s, 6H, CH_3); ^{13}C NMR (75 MHz, $CDCl_3$): δ = 187.7 (C), 186.3 (C), 146.7 (C), 138.2 (C), 133.1 (C), 132.5 (CH), 130.8 (CH), 130.0 (CH), 128.4 (CH), 126.5 (CH), 21.4 (CH_3); IR: ῡ = 2923 w, 1647 v str, 1591 w, 1485 m, 1303 w, 771 w; HRMS (APCI) calculated for $[M+H]^+$ 289.1223, $C_{20}H_{17}O_2$ found: 289.1220.

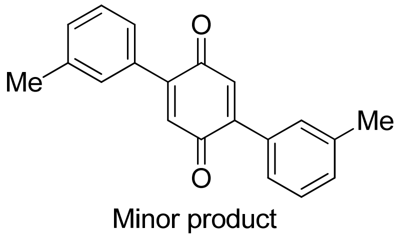

Minor product
4d

Dark brown thick oil, 28% yield; R_f 0.90 (4:1 petroleum ether:EtOAc); ^{1}H NMR (300 MHz, $CDCl_3$): δ = 7.40 – 7.27 (m, 8H, Ar-H), 6.95 (s, 2H, O=C-CH), 2.42 (s, 6H, CH_3); ^{13}C NMR (75 MHz, $CDCl_3$): δ = 187.1 (C), 145.7 (C), 138.3 (C), 133.1 (CH), 132.5 (C), 130.9 (CH), 129.9 (CH), 128.4 (CH), 126.4 (CH), 21.5 (CH_3); IR: ῡ = 2923 w, 1646 v str, 1580 w, 1349 w, 785 str; HRMS (APCI) calculated for $[M+H]^+$ 289.1223, $C_{20}H_{17}O_2$ found: 289.1224.

2,5-Diphenyl-1,4-benzoquinone (4e)[9] and **2,6-Diphenyl-1,4-benzoquinone (5e)**[9]

General procedure 4 was followed to yield **4e** and **5e** in an approximate 3:2 ratio.

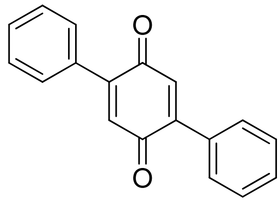

Major product
4e

Yellow solid, 44% yield; M. p. 214-218 °C; R_f 0.62 (80:20 dichloromethane:hexane); ^{1}H NMR (300 MHz, $CDCl_3$): δ = 7.60 – 7.51 (m, 4H, Ar-H), 7.51 – 7.43 (m, 6H, Ar-H), 6.97 (s, 2H, O=C-C<u>H</u>); ^{13}C NMR (75 MHz, $CDCl_3$): δ = 187.0 (C), 145.6 (C), 133.1 (CH), 132.5 (C), 130.1 (CH), 129.3 (CH), 128.6 (CH); IR: ῦ = 3053 w, 1640 v str, 1604 w, 1488 w, 1444 w, 904 v str, 769 v str, 696 v str.

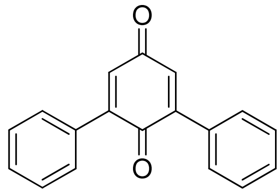

Minor product
5e

Red solid, 29% yield; M. p. 126-132 °C; R_f 0.28 (80:20 dichloromethane:hexane); ^{1}H NMR (300 MHz, $CDCl_3$): δ = 7.57 – 7.49 (m, 4H, Ar-H), 7.49 – 7.43 (m, 6H, Ar-H), 6.93 (s, 2H, O=C-C<u>H</u>); ^{13}C NMR (75 MHz, $CDCl_3$): δ = 187.6 (C), 186.1 (C), 146.5 (C), 133.2 (C), 132.6 (CH), 130.0 (CH), 129.4 (CH), 128.5 (CH); IR: ῦ = 3037 w, 1644 v str, 1601 w, 1591 w, 1494 w, 1447 str, 743 v str, 687 v str.

2,5-Bis-(4-trifluoromethylphenyl)-1,4-benzoquinone (4f)[10]

General procedure 4 was followed using an elevated temperature of 35 °C to give **4f**[10] as the major isomer. Some evidence of trace amounts of 2,6 isomer were observed however it was found to be unstable and impossible to isolate pure for characterisation.

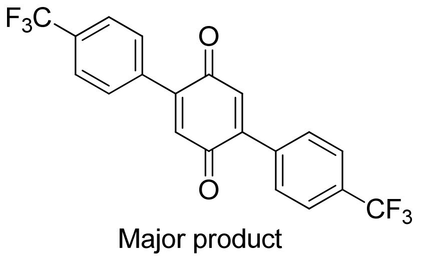

Major product
4f

Bright yellow solid, 51% yield; M. p. 211-216 °C; R_f 0.61 (4:1 petroleum ether:EtOAc); ^{1}H NMR (300 MHz, $CDCl_3$): δ = 7.75 (d, J = 8.3 Hz, 4H, Ar-H), 7.66 (d, J = 8.3 Hz, 4H, Ar-H), 7.03 (s, 2H, O=C-C<u>H</u>); ^{13}C NMR (75 MHz, $CDCl_3$): δ = 186.0 (C), 144.6 (C), 135.6 (C), 133.9 (CH), 132.1 (q, J = 33.0 Hz, C), 129.7 (CH), 125.6 (q, J = 3.7 Hz, CH), 123.8 (q, J = 272.6 Hz, C); IR: ῦ = 2925 w, 1660 w, 1645 v str, 1609 w, 1410 w, 1328 v str, 1113 v str, 1069 v str, 910 str, 854 v str, 817 m, 701 m; HRMS (APCI) calculated for $[M+H]^+$ 397.0658, $C_{20}H_{11}O_2F_6$ found: 397.0651.

A crystal structure was also obtained of this product. This can be found on page S34.

2,5-Bis-(4-ethoxycarbonylphenyl)-1,4-benzoquinone (4g)

General procedure 4 was followed using an elevated temperature of 35 °C and 3 equivalents (0.300 mmol) of 2,6-dichloro-1,4-benzoquinone. After 24 h, a second portion of boronic acid (0.051 mmol, 0.5 equiv.) and $Pd(OTFA)_2$ (0.005 mmol, 5 mol%) were added and after a further 7 h, $FeCl_3$ (0.272 mmol, 2.7 equiv.) was added as an additional oxidant. Upon purification by column chromatography, evidence of reduced product was observed so 2,6-dichloro-1,4-benzoquinone (0.250 mol, 2.5 equiv.) was added to the fraction tubes and the resulting solution stirred for 72 h. After evaporation under reduced pressure, the residue was purified by column chromatography to give **4g** in 25 % yield.

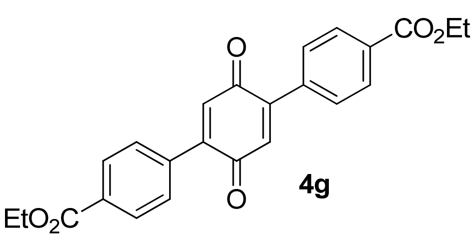

4g

Yellow amorphous solid, 25% yield; R_f 0.31 (2:1 hexane: EtOAc); ^{1}H NMR (300 MHz, $CDCl_3$): δ = 8.13 (d, J = 8.6 Hz, 4H, Ar-H), 7.61 (d, J = 8.6 Hz, 4H, Ar-H), 6.85 (s, 2H, O=C-C$\underline{H}$), 4.40 (q, J = 7.1 Hz, 4H, C$\underline{H_2}$CH$_3$), 1.41 (t, J = 7.1 Hz, 6H, CH$_2$C$\underline{H_3}$); ^{13}C NMR (75 MHz, $CDCl_3$): δ = 166.5 (C), 149.5 (C), 142.0 (C), 130.1 (CH), 129.7 (C), 129.3 (CH), 129.0 (C), 117.0 (CH), 61.2 (CH_2), 14.3 (CH_3); IR: ῦ = 3431 br m, 2978 m, 1709 str, 1693 str, 1605 m, 1447 m, 1432 w, 1398 m, 1367 m, 1272 v str, 1102 str, 858 str, 774 str, 711 str; HRMS (APCI) calculated for $[M+H]^+$ 405.1333, $C_{24}H_{21}O_6$ found: 405.1333.

2,5-Bis-(4-hydroxy-2-methylphenyl)-1,4-benzoquinone (4h) and **2,6-Bis-(4-hydroxy-2-methylphenyl)-1,4-benzoquinone (5h)**

General procedure 4 was followed to give **4h** and **5h** in 41% combined yield and an approximate 1:1 ratio. The products were not easily separable but a small amount of material was isolated of each isomer just for characterisation purposes.

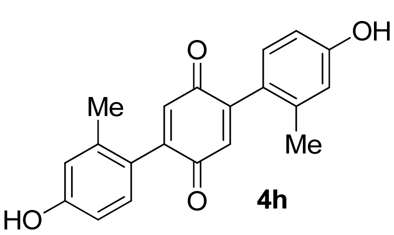

4h

Red solid, 15% yield; M. p. 65-67 °C; R_f 0.21 (1:1 hexane:EtOAc); ^{1}H NMR (300 MHz, Acetone-d$_6$): δ = 8.51 (s, 2H, O$\underline{H}$), 7.28 – 6.92 (m, 2H, Ar-H), 6.90 – 6.54 (m, 6H, Ar-H and 2 × O=C-C$\underline{H}$), 1.20 (s, 6H, C$\underline{H_3}$); ^{13}C NMR (101 MHz, Acetone-d$_6$): δ = 187.6 (C), 159.2 (C), 148.7 (C), 139.0 (C), 135.3 (CH), 132.0 (CH), 125.9 (C), 117.9 (CH), 113.4 (CH), 20.8 (CH_3); IR: ῦ = 3305 br str, 2923 w, 1694 m, 1647 str, 1602 v str, 1498 m, 1454 m, 1295 m, 1230 str, 1188 v

str, 990 m, 860 m, 752 m; HRMS (NSI) calculated for $[M+H]^+$ 321.1121, $C_{20}H_{17}O_4$ found: 321.1120.

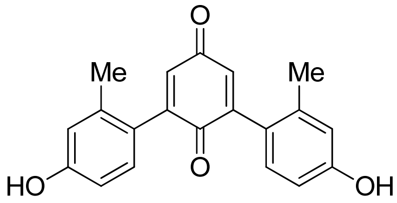

5h

Red solid, 5% yield; M. p. 50-52 °C; R_f 0.18 (1:1 hexane:EtOAc); ^{1}H NMR (300 MHz, Acetone-d_6): δ = 8.49 (s, 2H, O<u>H</u>), 7.05 (d, J = 8.3 Hz, 2H, O=C-C<u>H</u>), 6.76 (d, J = 2.2 Hz, 2H, Ar-H), 6.75 – 6.68 (m, 4H, Ar-H), 1.20 (s, 6H, C<u>H$_3$</u>); ^{13}C NMR (101 MHz, Acetone-d_6): δ = 188.5 (C), 186.6 (C), 159.2 (C), 149.5 (C), 138.8 (C), 134.8 (CH), 131.9 (CH), 126.6 (C), 117.9 (CH), 113.5 (CH), 20.9 (CH_3); IR: ῡ = 3305 br str, 2923 m, 1698 w, 1645 v str, 1602 v str, 1498 m, 1454 m, 1289 v str, 1225 str, 1086 str, 822 m, 791 m; HRMS (APCI) calculated for $[M+H]^+$ 321.1121, $C_{20}H_{17}O_4$ found: 321.1128.

2,6-Bis-(2-methoxyphenyl)-1,4-benzoquinone (5i) and **2,5-Bis-(2-methoxyphenyl)-1,4-benzoquinone (4i)**

General procedure 4 was followed to yield **5i** and **4i** in an approximate 1:1 ratio.

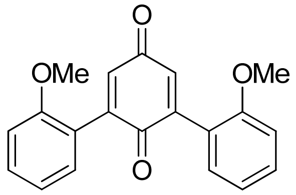

5i

Bright orange solid, 25% yield; M. p. 114-116 °C; R_f 0.32 (1:1 hexane:EtOAc); ^{1}H NMR (300 MHz, $CDCl_3$): δ = 7.41 (ddd, J = 8.3, 7.5, 1.8 Hz, 2H, Ar-H), 7.21 (dd, J = 7.5, 1.8 Hz, 2H, Ar-H), 7.07 – 6.92 (m, 4H, Ar-H), 6.87 (s, 2H, O=C-C<u>H</u>), 3.81 (s, 6H, OC<u>H$_3$</u>); ^{13}C NMR (75 MHz, $CDCl_3$): δ = 188.0 (C), 184.3 (C), 157.2 (C), 146.5 (C), 133.9 (CH), 131.0 (CH), 130.7 (CH), 123.3 (C), 120.6 (CH), 111.3 (CH), 55.8 (CH_3); IR: ῡ = 2936 w, 1647 str, 1598 str, 1576 m, 1489 str, 1453 m, 1249 str, 1128 m, 911 str, 823 w, 736 v str; HRMS (APCI) calculated for $[M+H]^+$ 321.1121, $C_{20}H_{17}O_4$ found: 321.1124.

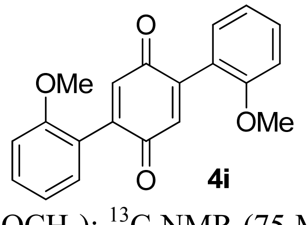

4i

Bright orange solid, 28% yield; M. p. 208-210 °C; R_f 0.36 (1:1 hexane:EtOAc); ^{1}H NMR (300 MHz, $CDCl_3$): δ = 7.42 (ddd, J = 8.4, 7.5, 1.8 Hz, 2H, Ar-H), 7.23 (dd, J = 7.5, 1.8 Hz, 2H, Ar-H), 7.09 – 6.95 (m, 4H, Ar-H), 6.91 (s, 2H, O=C-C<u>H</u>), 3.82 (s, 6H, OC<u>H$_3$</u>); ^{13}C NMR (75 MHz, $CDCl_3$): δ = 186.1 (C), 157.3 (C), 145.3 (C), 134.9 (CH), 131.1 (CH), 130.6 (CH), 122.6 (C), 120.6 (CH), 111.3 (CH), 55.7 (CH_3); IR: ῡ = 2963 w, 1651 str,

1607 m, 1591 m, 1488 m, 1466 m, 1280 m, 913 str, 822 m, 791 m, 760 v str; HRMS (APCI) calculated for $[M+H]^+$ 321.1121, $C_{20}H_{17}O_4$ found: 321.1121.

Palladium(II)-catalysed functionalization of monofunctionalized benzoquinone derivatives

2-(4-Hydroxyphenyl)-6-(4-methoxyphenyl)-1,4,benzoquinone (5j) and **2-(4-Hydroxyphenyl)-5-(4-methoxyphenyl)-1,4,benzoquinone (4j)**

General procedure 5 was followed to give the products **5j** and **4j** in >10:1 ratio.

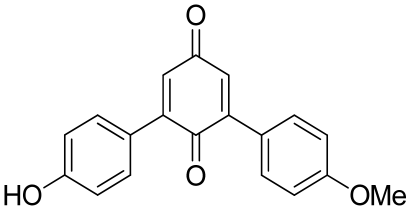

Major product
5j

Dark brown solid, 73% yield; M. p. 143-145 °C; R_f 0.50 (1:1 hexane:EtOAc); ^{1}H NMR (300 MHz, Acetone-d$_6$): δ = 8.83 (s, 1H, OH), 7.59 (d, J = 9.0 Hz, 2H, Ar-H), 7.52 (d, J = 8.9 Hz, 2H, Ar-H), 7.03 (d, J = 9.0 Hz, 2H, Ar-H), 6.94 (d, J = 8.9 Hz, 2H, Ar-H), 6.84 (d, J = 2.7 Hz, 1H, O=C-CH), 6.82 (d, J = 2.7 Hz, 1H, O=C-CH), 3.87 (s, 3H, OCH$_3$); ^{13}C NMR (101 MHz, Acetone-d$_6$): δ = 188.1 (C), 187.8 (C), 162.2 (C), 160.3 (C), 147.3 (C), 147.1 (C), 132.1 (CH), 132.0 (CH), 131.6 (CH), 131.2 (CH), 127.1 (C), 125.9 (C), 116.2 (CH), 114.7 (CH), 55.8 (CH$_3$); IR: ῦ = 3115 br str, 2928 str, 2840 m, 1635 v str, 1602 v str, 1583 v str, 1511 v str, 1444 str, 1236 v str, 1172 v str, 1028 str, 912 str, 827 str, 784 v str; HRMS (APCI) calculated for $[M+H]^+$ 307.0965, $C_{19}H_{15}O_4$ found: 307.0963.

A crystal structure was also obtained of this product. This can be found on page S31.

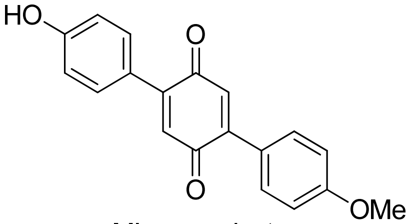

Minor product
4j

Dark brown solid, <5% yield; M. p. 190-192 °C; R_f 0.56 (1:1 hexane:EtOAc); ^{1}H NMR (300 MHz, Acetone-d$_6$): δ = 8.85 (s, 1H, OH), 7.63 (d, J = 9.0 Hz, 2H, Ar-H), 7.55 (d, J = 8.9 Hz, 2H, Ar-H), 7.04 (d, J = 9.0 Hz, 2H, Ar-H), 6.94 (d, J = 8.9 Hz, 2H, Ar-H), 6.91 (s, 1H, O=C-CH), 6.89 (s, 1H, O=C-CH), 3.87 (s, 3H, OCH$_3$); ^{13}C NMR (101 MHz, Acetone-d$_6$): δ = 188.0 (C), 187.9 (C), 162.2 (C), 160.3 (C), 145.6 (C), 145.4 (C), 132.4 (2 × CH), 132.1 (CH), 131.9 (CH), 126.1 (C), 125.0 (C), 116.2 (CH), 114.7 (CH), 55.8 (CH$_3$); IR: ῦ = 3387 br str, 2925 str, 2853 m, 1638 v str, 1600 v str, 1510 v str, 1441 m, 1247 v str, 1175 v str, 1028 str, 904 str, 835 str, 786 w; HRMS (APCI) calculated for $[M+H]^+$ 307.0965, $C_{19}H_{15}O_4$ found: 307.0966.

2-(4-Hydroxyphenyl)-6-(3,4-dimethoxyphenyl)-1,4,benzoquinone (5k) and **2-(4-Hydroxyphenyl)-5-(3,4-dimethoxyphenyl)-1,4,benzoquinone (4k)**

General procedure 5 was followed to give the products **5k** and **4k** in an approximate 7:1 ratio.

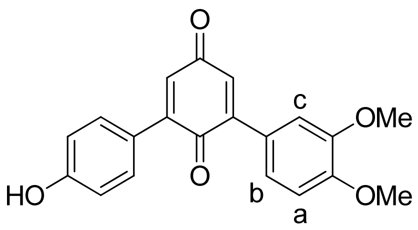

Major product
5k

Dark red solid, 71% yield; M. p. 169-170 °C; R_f 0.43 (1:1 hexane:EtOAc); ^{1}H NMR (400 MHz, Acetone-d$_6$): δ = 8.84 (s, 1H, OH), 7.52 (d, J = 8.9 Hz, 2H, Ar-H), 7.25 (d, J = 2.1 Hz, 1H, H$_c$), 7.22 (dd, J = 8.4, 2.1 Hz, 1H, H$_b$), 7.04 (d, J = 8.4 Hz, 1H, H$_a$), 6.94 (d, J = 8.9 Hz, 2H, Ar-H), 6.87 (d, J = 2.7 Hz, 1H, O=C-CH), 6.82 (d, J = 2.7 Hz, 1H, O=C-CH), 3.87 (s, 3H, OCH$_3$), 3.87 (s, 3H, OCH$_3$); ^{13}C NMR (101 MHz, Acetone-d$_6$): δ = 188.1 (C), 187.7 (C), 160.1 (C), 152.1 (C), 150.3 (C), 147.2 (C), 147.1 (C), 132.2 (CH), 131.7 (CH), 131.1 (CH), 127.2 (C), 125.9 (C), 123.8 (CH), 116.1 (CH), 114.2 (CH), 112.3 (CH), 56.3 (CH$_3$), 56.2 (CH$_3$); IR: $\tilde{\upsilon}$ = 3395 br, 2935 w, 1637 str, 1583 str, 1510 v str, 1440 w, 1415 w, 1251 v str, 1214 str, 1171 str, 1019 str, 817 w, 767 w; HRMS (NSI) calculated for $[M+H]^+$ 337.1071, $C_{20}H_{17}O_5$ found: 337.1073.

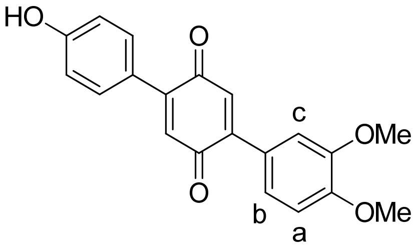

Minor product
4k

Dark red solid, 10% yield; M. p. 198-200 °C; R_f 0.40 (1:1 hexane:EtOAc); ^{1}H NMR (400 MHz, Acetone-d$_6$): δ = 8.86 (s, 1H, OH), 7.55 (d, J = 8.7 Hz, 2H, Ar-H), 7.29 – 7.24 (m, 2H, H$_b$ and H$_c$), 7.05 (d, J = 9.0 Hz, 1H, H$_a$), 6.94 (d, J = 8.7 Hz, 2H, Ar-H), 6.94 (s, 1H, O=C-CH), 6.89 (s, 1H, O=C-CH), 3.88 (s, 6H, 2 × OCH$_3$); ^{13}C NMR (101 MHz, Acetone-d$_6$): δ = 188.1 (C), 187.9 (C), 160.3 (C), 152.2 (C), 150.1 (C), 145.5 (C), 145.4 (C), 132.5 (CH), 132.1 (CH), 132.0 (CH), 126.3 (C), 125.1 (C), 123.9 (CH), 116.2 (CH), 113.9 (CH), 112.3 (CH), 56.3 (CH$_3$), 56.2 (CH$_3$); IR: $\tilde{\upsilon}$ = 3449 br m, 2922 str, 1637 v str, 1588 str, 1512 v str, 1466 w, 1430 w, 1271 m, 1140 str, 1019 str, 818 m, 766 m; HRMS (NSI) calculated for $[M+H]^+$ 337.1071, $C_{20}H_{17}O_5$ found: 337.1074.

2-(3,4-Dimethoxyphenyl)-6-(4-methoxyphenyl)-1,4,benzoquinone (5l) and 2-(3,4-Dimethoxyphenyl)-5-(4-methoxyphenyl)-1,4,benzoquinone (4l)

General procedure 5 was followed to give the products **5l** and **4l** in an approximate 3:1 ratio.

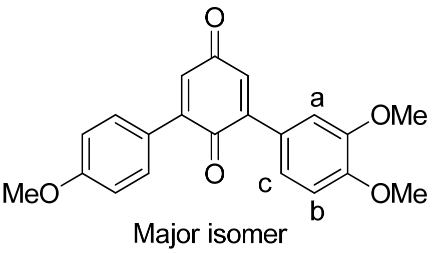

Major isomer
5l

Dark red solid, 65% yield; M. p. 140-142 °C; R_f 0.27 (1:1 hexane:EtOAc); ^{1}H NMR (300 MHz, $CDCl_3$): δ = 7.50 (d, J = 8.8 Hz, 2H, Ar-H), 7.16 (dd, J = 8.4, 2.1 Hz, 1H, H_c), 7.09 (d, J = 2.1 Hz, 1H, H_a), 6.98 (d, J = 8.8 Hz, 2H, Ar-H), 6.94 (d, J = 8.4 Hz, 1H, H_b), 6.87 (d, J = 2.7 Hz, 1H, O=C-C<u>H</u>), 6.86 (d, J = 2.7 Hz, 1H, O=C-C<u>H</u>), 3.93 (s, 3H, OC<u>H</u>$_3$), 3.92 (s, 3H, OC<u>H</u>$_3$), 3.86 (s, 3H, OC<u>H</u>$_3$); ^{13}C NMR (75 MHz, $CDCl_3$): δ = 187.6 (C), 186.9 (C), 161.2 (C), 150.8 (C), 148.8 (C), 145.9 (C), 145.7 (C), 131.4 (CH), 131.1 (CH), 130.9 (CH), 125.8 (C), 125.6 (C), 122.7 (CH), 114.0 (CH), 112.4 (CH), 110.9 (CH), 55.99 (CH_3), 55.97 (CH_3), 55.4 (CH_3); IR: ῦ = 3009 w, 2935 w, 2837 w, 1640 v str, 1601 str, 1510 v str, 1463 w, 1249 str, 1175 str, 1146 m, 1025 m; HRMS (APCI) calculated for $[M+H]^+$ 351.1227, $C_{21}H_{19}O_5$ found: 351.1226.

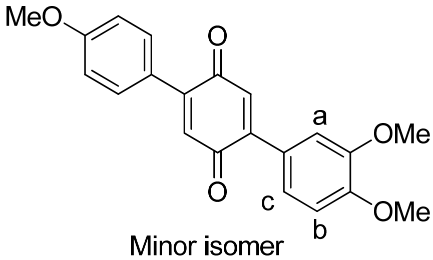

Minor isomer
4l

Bright red solid, 21% yield; M. p. 156-158 °C; R_f 0.30 (1:1 hexane:EtOAc); ^{1}H NMR (300 MHz, $CDCl_3$): δ = 7.55 (d, J = 8.8 Hz, 2H, Ar-H), 7.21 (dd, J = 8.4, 2.1 Hz, 1H, H_c), 7.13 (d, J = 2.1 Hz, 1H, H_a), 6.99 (d, J = 8.8 Hz, 2H, Ar-H), 6.95 (d, J = 8.4 Hz, 1H, H_b), 6.92 (s, 1H, O=C-C<u>H</u>), 6.90 (s, 1H, O=C-C<u>H</u>), 3.94 (app. s, 6H, 2 × OC<u>H</u>$_3$), 3.87 (s, 3H, OC<u>H</u>$_3$); ^{13}C NMR (75 MHz, $CDCl_3$): δ = 187.4 (C × 2), 161.4 (C), 151.0 (C), 148.9 (C), 144.8 (C × 2), 131.8 (CH), 131.6 (CH), 130.9 (CH), 125.1 (C), 124.8 (C), 122.8 (CH), 114.1 (CH), 112.2 (CH), 111.0 (CH), 56.00 (CH_3), 55.98 (CH_3), 55.0 (CH_3); IR: ῦ = 3003 w, 2935 w, 2837 w, 1645 v str, 1601 str, 1588 str, 1510 v str, 1463 w, 1259 str, 1175 str, 1145 m, 1025 m; HRMS (APCI) calculated for $[M+H]^+$ 351.1227, $C_{21}H_{19}O_5$ found: 351.1226.

2-(4-Hydroxyphenyl)-6-(2-methoxyphenyl)-1,4,benzoquinone (5m) and 2-(4-Hydroxyphenyl)-5-(2-methoxyphenyl)-1,4,benzoquinone (4m)

General procedure 5 was followed to give the products **5m** and **4m** in an approximate 5:4 ratio.

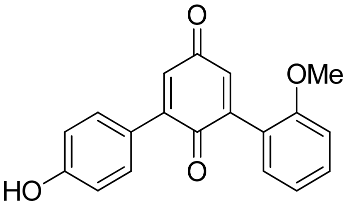

Major product
5m

Dark red solid, 50% yield; M. p. 178-181 °C; R_f 0.19 (1:1 hexane:EtOAc); ^{1}H NMR (300 MHz, Acetone-d_6): δ = 8.86 (s, 1H, OH), 7.51 (d, J = 8.7 Hz, 2H, Ar-H), 7.45 (ddd, J = 8.3, 7.5, 1.8 Hz, 1H, Ar-H), 7.32 (dd, J = 7.5, 1.8 Hz, 1H, Ar-H), 7.12 (dd, J = 8.3, 1.0 Hz, 1H, Ar-H), 7.04 (td, J = 7.5, 1.0 Hz, 1H, Ar-H), 6.94 (d, J = 8.7 Hz, 2H, Ar-H), 6.86 (d, J = 2.7 Hz, 1H, O=C-CH), 6.77 (d, J = 2.7 Hz, 1H, O=C-CH), 3.83 (s, 3H, OCH$_3$); ^{13}C NMR (75 MHz, Acetone-d_6): δ = 187.9 (C), 186.6 (C), 160.3 (C), 158.2 (C), 148.1 (C), 147.3 (C), 134.1 (CH), 132.0 (CH), 131.9 (CH), 131.2 (CH), 130.4 (CH), 125.7 (C), 124.9 (C), 121.4 (CH), 116.2 (CH), 112.2 (CH), 56.2 (CH$_3$); IR: ῡ = 3255 br, 2952 w, 1664 m, 1637 str, 1571 str, 1511 m, 1275 str, 1229 v str, 1174 m, 908 str, 743 str; HRMS (NSI) calculated for $[M+H]^+$ 307.0965, $C_{19}H_{15}O_4$ found: 307.0968.

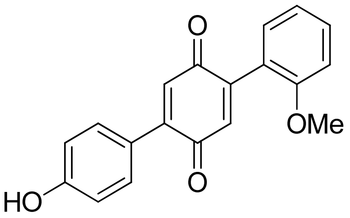

Minor product
4m

Red solid, 41% yield; M. p. 207-209 °C; R_f 0.23 (1:1 hexane:EtOAc); ^{1}H NMR (300 MHz, Acetone-d_6): δ = 8.85 (s, 1H, OH), 7.55 (d, J = 8.3 Hz, 2H, Ar-H), 7.49 – 7.38 (m, 1H, Ar-H), 7.27 (dd, J = 7.5, 1.7 Hz, 1H, Ar-H), 7.11 (d, J = 8.3 Hz, 1H, Ar-H), 7.06 – 7.00 (m, 1H, Ar-H), 6.95 (d, J = 8.3 Hz, 2H, Ar-H), 6.90 (d, J = 0.7 Hz, 1H, O=C-CH), 6.84 (d, J = 0.7 Hz, 1H, O=C-CH), 3.80 (d, J = 0.7 Hz, 3H, OCH$_3$); ^{13}C NMR (75 MHz, Acetone-d_6): δ = 188.1 (C), 186.4 (C), 158.4 (C), 146.4 (C), 145.6 (C), 135.5 (CH), 134.2 (C), 132.1 (CH), 131.8 (CH), 131.7 (CH), 131.4 (CH), 125.1 (C), 123.9 (C), 121.2 (CH), 116.2 (CH), 112.3 (CH), 56.1 (CH$_3$); IR: ῡ = 3423 br, 2922 w, 1635 v str, 1588 v str, 1514 v str, 1437 m, 1248 v str, 1173 v str, 763 v str, 753 str; HRMS (NSI) calculated for $[M+H]^+$ 307.0965, $C_{19}H_{15}O_4$ found: 307.0968.

2-(4-Ethoxycarbonylphenyl)-6-(3,4-dimethoxyphenyl)-1,4,benzoquinone (5n) and **2-(4-Ethoxycarbonylphenyl)-5-(3,4-dimethoxyphenyl)-1,4,benzoquinone (4n)**

General procedure 5 was followed to give the products **5n** and **4n** in an approximate 3:1 ratio. See page S27 for the procedure used to differentiate between the 2,5 and 2,6 isomers (**4n** and **5n**).

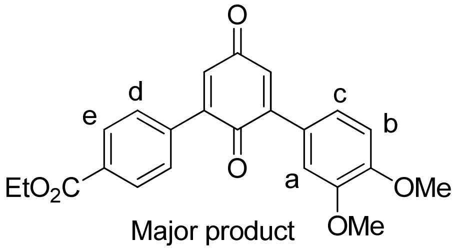

Major product
5n

Black solid, 48% yield; M. p. 148-150 °C; R_f 0.30 (2:1 hexane:EtOAc); ^{1}H NMR (300 MHz, $CDCl_3$): δ = 8.13 (d, J = 8.7 Hz, 2H, H_e), 7.58 (d, J = 8.7 Hz, 2H, H_d), 7.17 (dd, J = 8.4, 2.1 Hz, 1H, H_c), 7.09 (d, J = 2.1 Hz, 1H, H_a), 7.00 – 6.90 (m, 3H, 2 × O=C-C$\underline{H}$, H_b), 4.41 (q, J = 7.1 Hz, 2H, $C\underline{H}_2CH_3$), 3.94 (s, 3H, $OC\underline{H}_3$), 3.93 (s, 3H, $OC\underline{H}_3$), 1.42 (t, J = 7.1 Hz, 3H, $CH_2C\underline{H}_3$); ^{13}C NMR (75 MHz, $CDCl_3$): δ = 187.2 (C), 186.0 (C), 166.0 (C), 151.1 (C), 148.9 (C), 145.8 (C), 145.7 (C), 137.5 (C), 133.4 (CH), 131.6 (C), 131.4 (CH), 129.5 (CH), 129.3 (CH), 125.5 (C), 122.9 (CH), 112.3 (CH), 111.0 (CH), 61.3 (CH_2), 56.02 (CH_3), 55.99 (CH_3), 14.3 (CH_3); IR: ῡ = 2935 w, 1715 str, 1643 v str, 1513 str, 1464 m, 1257 v str, 1100 v str, 1022 v str, 917 w, 748 v str, 706 m; HRMS (APCI) calculated for $[M+H]^+$ 393.1333, $C_{23}H_{21}O_6$ found: 393.1328.

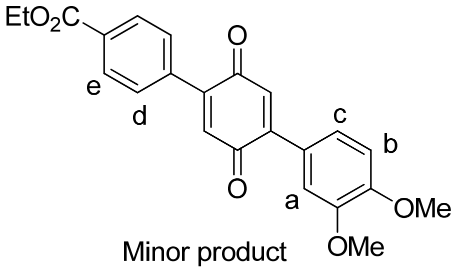

Minor product
4n

Black solid, 16% yield; M. p. 186-188 °C; R_f 0.33 (2:1 hexane:EtOAc); ^{1}H NMR (300 MHz, $CDCl_3$): δ = 8.13 (d, J = 8.4 Hz, 2H, H_e), 7.61 (d, J = 8.4 Hz, 2H, H_d), 7.22 (dd, J = 8.4, 2.1 Hz, 1H, H_c), 7.13 (d, J = 2.1 Hz, 1H, H_a), 7.01 – 6.93 (m, 3H, 2 × O=C-C$\underline{H}$, H_b), 4.41 (q, J = 7.1 Hz, 2H, $C\underline{H}_2CH_3$), 3.95 (s, 3H, $OC\underline{H}_3$), 3.94 (s, 3H, $OC\underline{H}_3$), 1.42 (t, J = 7.1 Hz, 3H, $CH_2C\underline{H}_3$); ^{13}C NMR (75 MHz, $CDCl_3$): δ = 187.2 (C), 186.4 (C), 166.0 (C), 151.2 (C), 149.0 (C), 145.0 (C), 144.7 (C), 136.7 (C), 133.9 (CH), 131.7 (C), 131.6 (CH), 129.6 (CH), 129.3 (CH), 124.8 (C), 122.9 (CH), 112.6 (CH), 111.1 (CH), 61.3 (CH_2), 56.03 (CH_3), 56.01 (CH_3), 14.3 (CH_3); IR: ῡ = 2934 w, 1706 v str, 1644 v str, 1587 m, 1516 str, 1443 m, 1287 v str, 1268 v str, 1105 v str, 1022 v str, 910 str, 762 m, 701 m; HRMS (APCI) calculated for $[M+H]^+$ 393.1333, $C_{23}H_{21}O_6$ found: 393.1328.

2-(4-Ethoxycarbonylphenyl)-5-(4-trifluoromethylphenyl)-1,4,benzoquinone (4o) and **2-(4-Ethoxycarbonylphenyl)-6-(4-trifluoromethylphenyl)-1,4,benzoquinone (5o)**

General procedure 5 was followed to give the products **4o** and **5o** in an approximate 10:1 ratio.

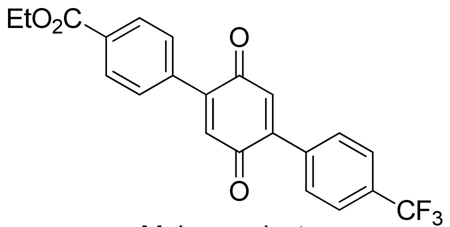

Major product
4o

Bright yellow amorphous solid, 47% yield; R_f 0.43 (5:1 hexane:EtOAc); ^{1}H NMR (300 MHz, $CDCl_3$): δ = 8.14 (d, J = 8.6 Hz, 2H, Ar-H), 7.74 (d, J = 8.3 Hz, 2H, Ar-H), 7.66 (d, J = 8.3 Hz, 2H, Ar-H), 7.62 (d, J = 8.6 Hz, 2H, Ar-H), 7.04 (s, 1H, O=C-CH), 7.02 (s, 1H, O=C-CH), 4.42 (q, J = 7.1 Hz, 2H, CH_2CH_3), 1.42 (t, J = 7.1 Hz, 3H, CH_2CH_3); ^{13}C NMR (101 MHz, $CDCl_3$): δ = 186.10 (C), 186.06 (C), 165.9 (C), 145.0 (C), 144.5 (C), 136.4 (C), 135.7 (C), 134.0 (CH), 133.7 (CH), 132.0 (C), 131.9 (C), 129.7 (2 × CH), 129.3 (CH), 125.5 (q, J = 3.9 Hz, CH), 123.8 (q, J = 271.6 Hz, C), 61.3 (CH_2), 14.3 (CH_3); IR: ῦ = 2924 w, 1723 v str, 1642 v str, 1326 str, 1278 str, 1125 v str, 1107 v str, 1069 v str, 909 v str, 713 str; HRMS (APCI) calculated for $[M+H]^+$ 401.0995, $C_{22}H_{16}F_3O_4$ found: 401.0995.

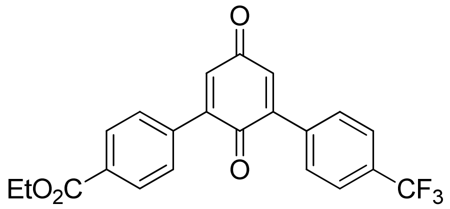

Minor product
5o

Pale yellow amorphous solid, <5% yield; R_f 0.36 (5:1 hexane:EtOAc); ^{1}H NMR (300 MHz, $CDCl_3$): δ = 8.13 (d, J = 8.5 Hz, 2H, Ar-H), 7.73 (d, J = 8.2 Hz, 2H, Ar-H), 7.63 (d, J = 8.2 Hz, 2H, Ar-H), 7.58 (d, J = 8.5 Hz, 2H, Ar-H), 6.99 (d, J = 2.6 Hz, 1H, O=C-CH), 6.97 (d, J = 2.6 Hz, 1H, O=C-CH), 4.41 (q, J = 7.1 Hz, 2H, CH_2CH_3), 1.42 (t, J = 7.1 Hz, 3H, CH_2CH_3); ^{13}C NMR (75 MHz, $CDCl_3$): δ = 186.8 (C), 185.1 (C), 165.9 (C), 145.6 (C), 145.3 (C), 137.0 (C), 136.4 (C), 133.6 (CH), 133.5 (CH), 132.2 (C), 131.9 (C), 129.8 (CH), 129.6 (CH), 129.4 (CH), 125.46 (q, J = 3.9 Hz, CH), 124.8 (q, J = 271.6 Hz, C), 61.3 (CH_2), 14.3 (CH_3); IR: ῦ = 2924 w, 2855 str, 1715 str, 1609 w, 1457 w, 1325 v str, 1278 str, 1124 v str, 1068 v str, 843 w, 758 m; HRMS (APCI) calculated for $[M+H]^+$ 401.0995, $C_{22}H_{16}F_3O_4$ found: 401.0995.

2-(1-(*tert*-Butoxycarbonyl)-2-pyrrolyl)-5-(3-thienyl)-1,4,benzoquinone (4p)

General procedure 5 was followed, using 2.5 equivalents of 3-thienyl boronic acid to give the 2,5 isomer **4p**. Another product, believed to be the 2,6 isomer was observed but only in trace amounts.

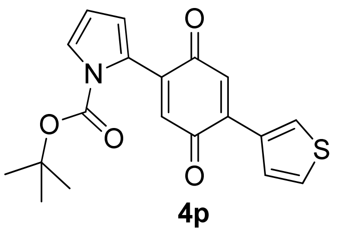

4p

Red solid, 74% yield; M. p. 125-127 °C; R_f 0.50 (2:1 hexane:EtOAc); ^{1}H NMR (400 MHz, $CDCl_3$): δ = 8.15 (dd, J = 2.8, 1.5 Hz, 1H, HetAr-H), 7.42 (dd, J = 3.4, 1.7 Hz, 1H, HetAr-H), 7.41 – 7.37 (m, 2H, HetAr-H), 6.99 (s, 1H, O=C-C<u>H</u>), 6.78 (s, 1H, O=C-C<u>H</u>), 6.41 (dd, J = 3.4, 1.7 Hz, 1H, HetAr-H), 6.26 (t, J = 3.4 Hz, 1H, HetAr-H), 1.52 (s, 9H, (C<u>H</u>$_3$)$_3$); ^{13}C NMR (101 MHz, $CDCl_3$): δ = 187.1 (C), 186.3 (C), 148.7 (C), 141.2 (C), 139.3 (C), 133.0 (C), 130.8 (CH), 130.2 (CH), 129.7 (CH), 126.9 (CH), 126.5 (C), 125.9 (CH), 125.0 (CH), 117.0 (CH), 111.1 (CH), 84.7 (C), 27.9 (CH_3); IR: ῦ = 2979 w, 1744 v str, 1655 v str, 1587 str, 1474 w, 1405 w, 1315 v str, 1223 m, 1136 v str, 848 w, 734 w; HRMS (NSI) calculated for $[M+H]^+$ 356.0951, $C_{19}H_{18}O_4NS$ found: 356.0954.

2-(3-Nitrophenyl)-5-(3-thienyl)-1,4,benzoquinone (4q)

General procedure 5 was followed, using 2.5 equivalents of 3-thienyl boronic acid and $FeCl_3$ (1.25 equiv.) was added for 1 h at the end of the reaction. Column chromatography yielded the 2,5 isomer **4q** as the major product but evidence of reduced product was observed following washing of the column with ethyl acetate. $FeCl_3$ (2.5 equiv.), was added as an additional oxidant to the column wash and left to stir at room temperature for 18 h, after which the solution was evaporated under reduced pressure and purified by column chromatography to yield further 2,5 product **4q**. Another product, believed to be the 2,6 isomer was observed but only in trace amounts.

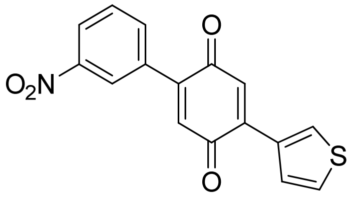

4q

Red solid, 42% yield; M. p. 175-178 °C; R_f 0.27 (2:1 hexane:EtOAc); ^{1}H NMR (400 MHz, $CDCl_3$): δ = 8.45 – 8.41 (m, 1H, Ar-H), 8.36 – 8.31 (m, 1H, Ar-H), 8.23 – 8.21 (m, 1H, HetAr-H), 7.90 – 7.86 (m, 1H, Ar-H), 7.70 – 7.63 (m, 1H, Ar-H), 7.45 – 7.43 (m, 2H, HetAr-H), 7.08 (s, 1H, O=C-C<u>H</u>), 7.01 (s, 1H, O=C-C<u>H</u>); ^{13}C NMR (101 MHz, $CDCl_3$): δ = 186.6 (C), 186.2 (C), 148.3 (C), 143.1 (C), 139.3 (C), 135.2 (CH), 134.3 (CH), 134.0 (C), 132.3 (C), 130.4 (CH), 130.1 (CH), 129.6

(CH), 126.7 (CH), 126.4 (CH), 124.6 (CH), 124.3 (CH); IR: ῦ = 3107 w, 2925 w, 1650 v str, 1583 w, 1528 v str, 1347 str, 1220 w, 1158 w, 906 w, 730 w; HRMS (APCI) calculated for $[M+H]^+$ 312.0325, $C_{16}H_{10}O_4NS$ found: 312.0327.

2-(4-Ethoxycarbonylphenyl)-5-(3-thienyl)-1,4,benzoquinone (4r)

General procedure 5 was followed to give **4r** as the major product. Another product, believed to be the 2,6 isomer was observed but only in trace amounts.

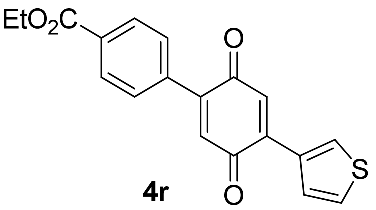

4r

Red solid, 34% yield; M. p. 144-146 °C; R_f 0.26 (3:1 hexane:EtOAc); ^1H NMR (300 MHz, CDCCl$_3$): δ = 8.20 (dd, J = 2.7, 1.6 Hz, 1H, HetAr-H), 8.12 (d, J = 8.6 Hz, 2H, Ar-H), 7.61 (d, J = 8.6 Hz, 2H, Ar-H), 7.44 – 7.41 (m, 2H, HetAr-H), 7.05 (s, 1H, O=C-C<u>H</u>), 6.96 (s, 1H, O=C-C<u>H</u>), 4.41 (q, J = 7.2 Hz, 2H, $C\underline{H_2}CH_3$), 1.41 (t, J = 7.2 Hz, 3H, $CH_2C\underline{H_3}$); ^{13}C NMR (101 MHz, $CDCl_3$): δ = 186.9 (C), 186.7 (C), 166.0 (C), 144.6 (C), 139.2 (C), 136.7 (C), 134.0 (CH), 132.6 (C), 131.9 (C), 130.4 (CH), 130.0 (CH), 129.6 (CH), 129.3 (CH), 126.8 (CH), 126.2 (CH), 61.2 (CH_2), 14.3 (CH_3); IR: ῦ = 3105 w, 2981 w, 1717 v str, 1652 v str, 1640 v str, 1583 w, 1409 w, 1277 v str, 1107 str, 911 w, 792 w, 723 m; HRMS (APCI) calculated for $[M+H_2O-H]^+$ 355.0635, $C_{19}H_{15}O_5S$ found: 355.0629.

5. Diacetylation of 2,5- and 2,6-diaryl-1,4-hydroquinones[11]

Acetylation of **5n** and **4n** was carried out in order to differentiate between 2,5 and 2,6 isomers. ^4J coupling could be observed in the ^{1}H NMR spectrum of the 2,6 isomer once acetylation had been carried out, which was absent in the ^{1}H NMR spectrum for the 2,5 isomer. The procedure for the diacetylation of 2,5- and 2,6-diaryl-1,4-hydroquinones was taken from A. Ortega *et al.*, *Bioorg. Med. Chem. Lett.,* **2011**, *21*, 2183-2187.

Ethyl 4-[2,5-bis(acetyloxy)-4-(3,4-dimethoxyphenyl)phenyl]benzoate (SI-1)

Compound **4n** (0.0133 mmol) was diacetylated using the aforementioned procedure[11] to give **SI-1** in a 78% yield.

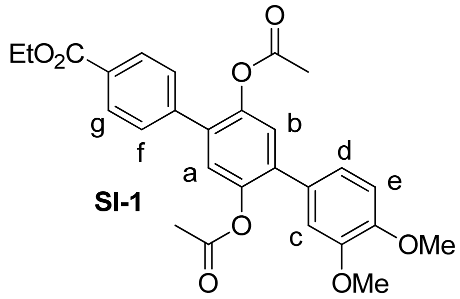

Yellow amorphous solid, 78% yield; R_f 0.23 (2:1 hexane:EtOAc); ^{1}H NMR (400 MHz, $CDCl_3$): δ = 8.10 (d, J = 8.6 Hz, 2H, H_g), 7.53 (d, J = 8.6 Hz, 2H, H_f), 7.20 (s, 1H, $H_{a/b}$), 7.19 (s, 1H, $H_{a/b}$), 7.02 (dd, J = 8.2, 2.0 Hz, 1H, H_d), 6.99 (d, J = 2.0 Hz, 1H, H_c), 6.93 (d, J = 8.2 Hz, 1H, H_e), 4.41 (q, J = 7.1 Hz, 2H, $C\underline{H_2}CH_3$), 3.93 (s, 3H, $OC\underline{H_3}$), 3.91 (s, 3H, $OC\underline{H_3}$), 2.13 (s, 3H, $C\underline{H_3}$), 2.11 (s, 3H, $C\underline{H_3}$), 1.42 (t, J = 7.1 Hz, 3H, $CH_2C\underline{H_3}$); ^{13}C NMR (101 MHz, $CDCl_3$): δ = 169.2 (C), 169.0 (C), 166.3 (C), 148.9 (C), 148.7 (C), 145.6 (C), 145.4 (C), 141.1 (C), 135.5 (C), 133.5 (C), 129.8 (C), 129.6 (CH), 129.0 (C), 128.8 (CH), 125.0 (CH), 124.8 (CH), 121.3 (CH), 111.9 (CH), 111.1 (CH), 61.1 (CH_2), 56.0 (CH_3), 55.9 (CH_3), 20.9 (CH_3), 20.8 (CH_3), 14.4 (CH_3); IR: $\tilde{\upsilon}$ = 2929 w, 1761 str, 1714 str, 1608 w, 1513 w, 1488 w, 1367 str, 1274 str, 1199 v str, 1155 v str, 915 m, 860 w, 730 m; HRMS (APCI) calculated for $[M+H]^+$ 479.1700, $C_{27}H_{27}O_8$ found: 479.1695.

Ethyl 4-[2,5-bis(acetyloxy)-3-(3,4-dimethoxyphenyl)phenyl]benzoate (SI-2)

Compound **5n** (0.0201 mmol) was diacetylated using the aforementioned procedure[11] to give **SI-2** in a 76% yield.

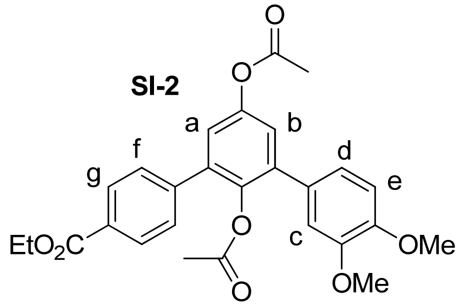

Yellow amorphous solid, 76% yield; R_f 0.22 (2:1 hexane:EtOAc); ^{1}H NMR (400 MHz, $CDCl_3$): δ = 8.08 (d, J = 8.5 Hz, 2H, H_g), 7.52 (d, J = 8.5 Hz, 2H, H_f), 7.16 (d, J = 2.8 Hz, 1H, $H_{a/b}$), 7.12 (d, J = 2.8 Hz, 1H, $H_{a/b}$), 7.00 (dd, J = 8.1, 2.0 Hz, 1H, H_d), 6.97 (d, J = 2.0

Hz, 1H, H_c), 6.91 (d, *J* = 8.1 Hz, 1H, H_e), 4.40 (q, *J* = 7.1 Hz, 2H, $C\underline{H_2}CH_3$), 3.92 (s, 3H, $OC\underline{H_3}$), 3.89 (s, 3H, $OC\underline{H_3}$), 2.32 (s, 3H, $C\underline{H_3}$), 1.82 (s, 3H, $C\underline{H_3}$), 1.41 (t, *J* = 7.1 Hz, 3H, $CH_2C\underline{H_3}$); ^{13}C NMR (101 MHz, $CDCl_3$): δ = 169.3 (C), 168.7 (C), 166.3 (C), 148.8 (C), 148.6 (C), 148.3 (C), 142.5 (C), 141.7 (C), 136.9 (C), 135.9 (C), 129.8 (C), 129.5 (CH), 129.5 (C), 128.9 (CH), 123.4 (CH), 122.3 (CH), 121.4 (CH), 112.0 (CH), 111.0 (CH), 61.1 (CH_2), 55.93 (CH_3), 55.88 (CH_3), 21.1 (CH_3), 20.6 (CH_3), 14.3 (CH_3); IR: ῦ = 2933 w, 1762 str, 1713 str, 1608 w, 1516 str, 1441 w, 1367 str, 1273 str, 1174 v str, 1160 v str, 1022 str, 915 m, 864 w, 731 m; HRMS (APCI) calculated for $[M+H]^+$ 479.1700, $C_{27}H_{27}O_8$ found: 479.1698.

6. Reducing catalyst loading

Generally, 7.5 mol% catalyst loading is necessary to give good yields in the monofunctionalization of benzoquinone, particularly with heterocyclic, alkyl or electron poor aryl boronic acid substrates in order for the reaction to go to completion within 18-48 h. However, more active aryl boronic acids still react well under lower catalyst loadings. The reaction of benzoquinone with arylboronic acid **6a** still furnishes a good 87% yield of product **3a** even with 1 mol% of catalyst.

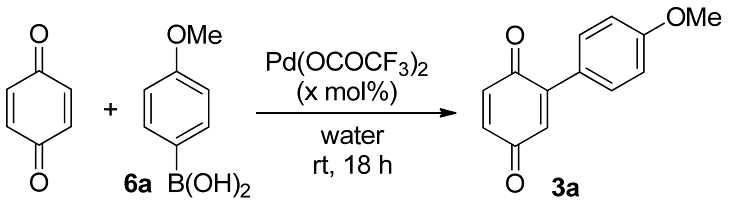

Catalyst loading:		
	7.5 mol%	88%
	1 mol%	87%
	0.1 mol%	37%

7. One pot hetero-difunctionalization procedure

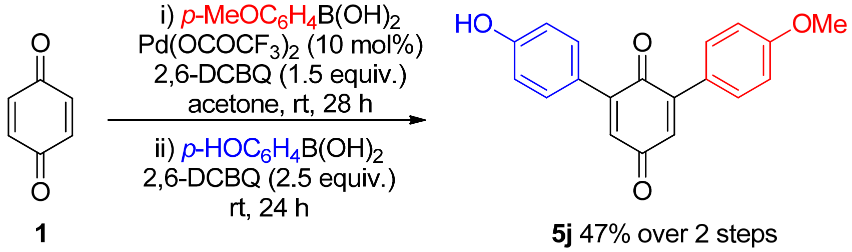

Benzoquinone (16.2 mg, 0.150 mmol), 4-methoxyphenyl boronic acid **6a** (15.3 mg, 0.101 mmol), 2,6-dichloro-1,4-benzoquinone (26.6 mg, 0.150 mmol) and palladium trifluoroacetate (3.3 mg, 9.94×10^{-3} mmol) were added to a round-bottomed flask with magnetic stirrer bar. Acetone (0.340 mL) was then added and the reaction was stirred at room temperature. After 26 hours, 4-hydroxyphenyl boronic acid (34.7 mg, 0.252 mmol) and 2,6-dichloro-1,4-benzoquinone (44.8 mg, 0.253 mmol) were added and the reaction left to stir for a further 18 h. The mixture was then evaporated to dryness, toluene (5 mL) and enough acetone to dissolve the heterogeneous mixture (0.5–1 mL) was added and the slurry was then purified directly by flash column chromatography to afford the hetero-difunctionalized product **5j** in 47% yield (14.5 mg, 0.0473 mol).

8. Control reactions: Reaction to ascertain if isomerization occurs between the 2,5 and 2,6 isomers in the C-H difunctionalization of benzoquinone

In order to ascertain if isomerization occurs during the C-H difunctionalization reactions, 2 control experiments were carried out. Separate samples of the 2,5 and 2,6 isomers of bis-phenyl-1,4-benzoquinone were subjected to the homo-difunctionalization reaction conditions (see scheme below) and the reactions monitored to see if formation of the other isomer was evident. Both reactions did not show any change from starting material to the other isomer, or other side products.

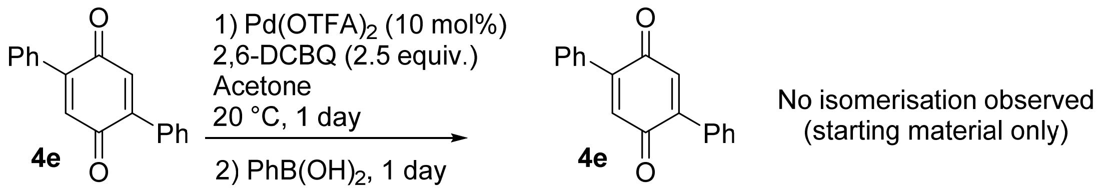

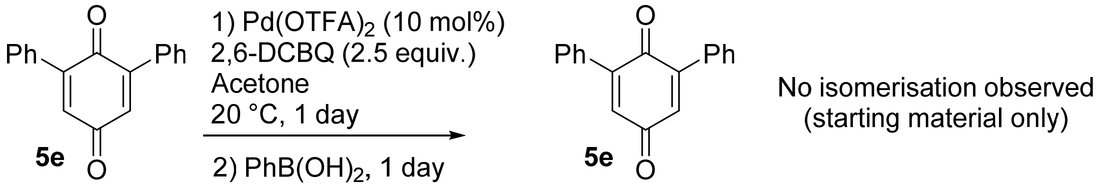

2,5-Bis-phenyl-1,4-benzoquinone **4e** (10.2 mg, 0.0392 mmol), 2,6-dichloro-1,4-benzoquinone (18.0 mg, 0.0980 mmol) and $Pd(OTFA)_2$ (1.8 g, 5.42×10^{-3} mmol), were added to a round bottomed flask equipped with a magnetic stir bar. Acetone (0.180 mL) was then added and the reaction stirred for 24 h. ^{1}H NMR analysis concluded that no formation of the 2,6 isomer had occurred. Phenyl boronic acid (2.4 mg, 0.0197 mmol) and additional acetone (0.5 mL) were then added and the reaction stirred for a further 24 h. Further ^{1}H NMR analysis again confirmed no formation of the 2,6 isomer.

2,6-Bis-phenyl-1,4-benzoquinone **5e** (8.2 mg, 0.0315 mmol), 2,6-dichloro-1,4-benzoquinone (14.8 mg, 0.0836 mmol) and $Pd(OTFA)_2$ (1.5 mg, 4.52×10^{-3} mmol), were added to a round bottomed flask equipped with a magnetic stir bar. Acetone (0.150 mL) was then added and the reaction stirred for 24 h. ^{1}H NMR analysis concluded that no formation of the 2,5 isomer had occurred. Phenyl boronic acid (2.1 mg, 0.0172 mmol) and additional acetone (0.5 mL) were then added and the reaction stirred for a further 24 h. Further ^{1}H NMR analysis again confirmed no formation of the 2,5 isomer.

9. Crystal structures of difunctionalized products

Crystal structures were obtained of a number difunctionalized benzoquinone products in order to confirm their conformation as either 2,5 or 2,6 isomers.

For hetero-difunctionalizations, 2,5 and 2,6 products were distinguished by observation of ^{4}J coupling in the ^{1}H NMR spectrum for the 2,6 isomer, corresponding to the alkenyl protons as shown in the diagram below. To confirm that those compounds with ^{4}J coupling were in fact the 2,6 isomer, a crystal structure was grown of one example which confirmed our hypothesis as correct.

Although ^{4}J coupling is not always observed in ^{1}H NMR for 2,6 homo-difunctionalized products, the 2,5 and 2,6 isomers can be successfully assigned via ^{13}C NMR data. We did however grow crystals of 2 homo-difunctionalized products to confirm their identity.

Crystal structure of 2,6-(4-hydroxyphenyl-4'-methoxyphenyl)-1,4-benzoquinone (5j)

CCDC number: 1016964

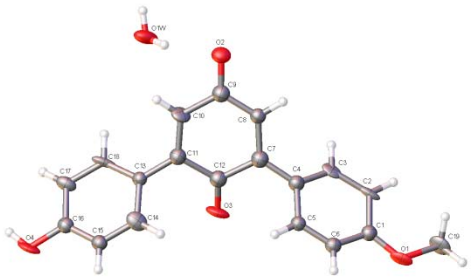

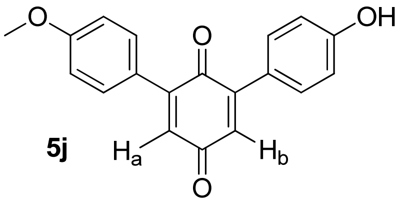

$^{4}J_{ab}$ = 2.7 Hz

Empirical formula	$C_{19}H_{16}O_5$
Formula weight	324.32
Temperature/K	100.15
Crystal system	monoclinic
Space group	$P2_1/n$
a/Å	3.772(3)
b/Å	10.287(9)
c/Å	38.63(4)
α/°	90

β/°	92.52(3)
γ/°	90
Volume/Å^3	1498(2)
Z	4
ρ_{calc}mg/mm^3	1.438
m/mm^{-1}	0.104
F(000)	680.0
Crystal size/mm^3	0.46 × 0.12 × 0.04
Radiation	MoKα (λ = 0.71073)
2Θ range for data collection	2.11 to 45.116°
Index ranges	$-4 \leq h \leq 3$, $-11 \leq k \leq 10$, $-40 \leq l \leq 41$
Reflections collected	10261
Independent reflections	1901 [R_{int} = 0.1754, R_{sigma} = 0.1606]
Data/restraints/parameters	1901/78/222
Goodness-of-fit on F^2	1.837
Final R indexes [I>=2σ (I)]	R_1 = 0.2081, wR_2 = 0.4974
Final R indexes [all data]	R_1 = 0.2697, wR_2 = 0.5244
Largest diff. peak/hole / e Å^{-3}	0.65/-0.69

Crystal structure of 2,6-Bis-(4-hydroxyphenyl)-1,4-benzoquinone (5a)

CCDC number: 1016966

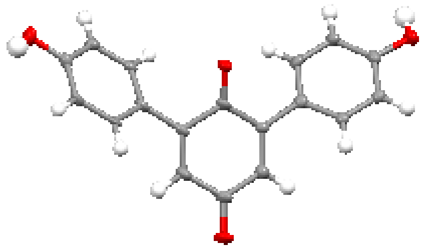

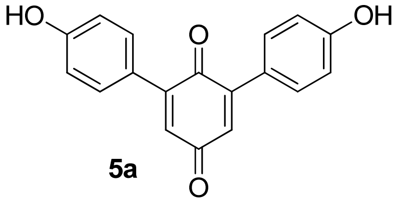

Empirical formula	$C_{18}H_{12}O_4$
Formula weight	292.28
Temperature/K	100.15
Crystal system	monoclinic
Space group	$P2_1$
a/Å	3.7532(6)
b/Å	10.5497(15)
c/Å	17.197(3)
α/°	90.00
β/°	93.643(9)
γ/°	90.00
Volume/Å^3	679.55(19)
Z	2
ρ_{calc}mg/mm^3	1.428
m/mm^{-1}	0.101
F(000)	304.0
Crystal size/mm^3	0.24 × 0.04 × 0.02
2Θ range for data collection	2.38 to 49.7°
Index ranges	$-4 \leq h \leq 4$, $-12 \leq k \leq 12$, $-20 \leq l \leq 19$
Reflections collected	7066
Independent reflections	2188[R(int) = 0.0572]
Data/restraints/parameters	2188/1/201
Goodness-of-fit on F^2	1.164
Final R indexes [I>=2σ (I)]	R_1 = 0.0590, wR_2 = 0.1664
Final R indexes [all data]	R_1 = 0.0901, wR_2 = 0.1826
Largest diff. peak/hole / e Å^{-3}	0.33/-0.37

Crystal structure of 2,5-Bis-(4-trifluoromethylphenyl)-1,4-benzoquinone (4f)

CCDC number: 1016965

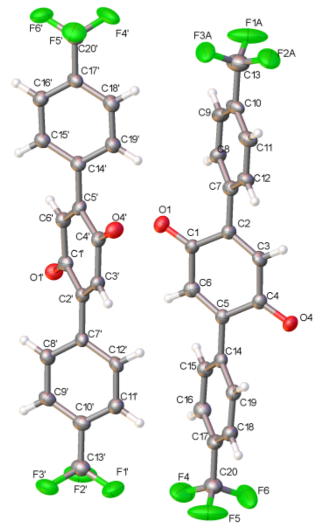

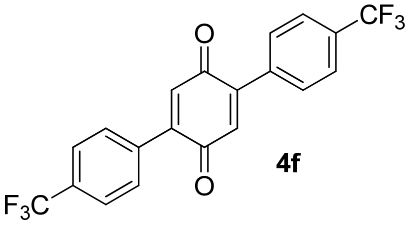

4f

Empirical formula	$C_{20}H_{10}F_6O_2$
Formula weight	396.29
Temperature/K	120
Crystal system	orthorhombic
Space group	$P2_12_12_1$
a/Å	6.179(2)
b/Å	19.363(7)
c/Å	27.410(10)
α/°	90
β/°	90
γ/°	90
Volume/Å^3	3279(18)
Z	8
ρ_{calc}mg/mm^3	1.605
m/mm^{-1}	0.149
F(000)	1600.0
Crystal size/mm^3	0.55 × 0.03 × 0.03

Radiation	MoKα (λ = 0.71075)
2Θ range for data collection	4.208 to 55°
Index ranges	$-7 \leq h \leq 8$, $-24 \leq k \leq 25$, $-23 \leq l \leq 35$
Reflections collected	0
Independent reflections	7471 [R_{int} = 0.066, R_{sigma} = 0.039]
Data/restraints/parameters	7471/51/533
Goodness-of-fit on F^2	1.064
Final R indexes [I>=2σ (I)]	R_1 = 0.0729, wR_2 = 0.1390
Final R indexes [all data]	R_1 = 0.1020, wR_2 = 0.1575
Largest diff. peak/hole / e $Å^{-3}$	0.29/-0.32

10. Reducing the Equivalents of BQ

Table S1. Reducing the Equivalents of Benzoquinone.

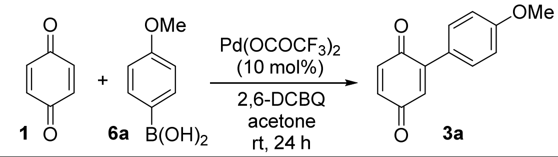

Entry	BQ (Equiv.)	2,6-DCBQ (Equiv.)	Yield **3a** (%)[a]
1	3	0	80
2	2	0	40[b]
3	1	1	54[b]
4	1.5	1.5	71

[a] Isolated yields. [b] Diarylated product **5** also present in crude mixture.

Attempts to reduce the amount of BQ required in the first step are shown in Table 4. Reducing the amount of BQ from 3 to the minimum 2 equiv. (1 equiv. as substrate, 1 equiv. as oxidant) resulted in a much lower 40% yield of **3a** (Entry 2). The lower yield is due to the formation of the undesired difunctionalized product **5**. Using only 1 equiv. of BQ, and adding 1 equiv. of 2,6-DCBQ as oxidant results in a moderate 54% of desired monofunctionalized product **3a**, but also evidence of the undesired homo-difunctionalized product **5** (Entry 3). To our delight, using 1.5 equiv. of BQ and 2,6-DCBQ respectively produces a good 71% yield of the desired **3a** (Entry 4). Adopting these new conditions allows for a successful one-pot C-H hetero-difunctionalization procedure (Scheme 2).

11. Oxidant Screens

Representative initial oxidant screen:

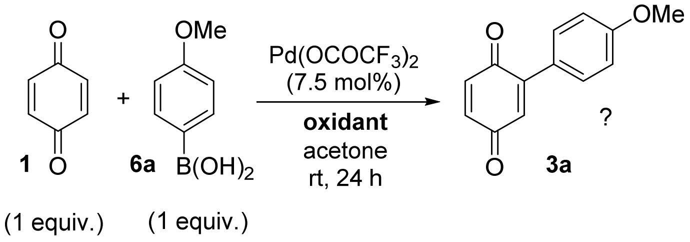

Entry	Oxidant	Result[a]
1	AcOOH	Full conversion to 4-methoxyphenol
2	BnOOBz	<5% conversion, hydroquinone present
3	H_2O_2	~50% conversion, 4-methoxyphenol
4	^{t}BuOOH	Unidentified Products
5	Oxone	<5% conversion, hydroquinone present
6	MnO_2	No reaction
7	DDQ	Hydroquinone present
8	$Ce(SO_4)_2$	20% conversion, hydroquinone present
9	$Cu(OAc)_2$	Unidentified Products – complex mixture.
10	p-Chloranil	Full conversion to mono-(**3a**) and difunctionalised products. However, hydroquinone present and reduced oxidant co-elutes with product upon column chromatography.
11	2,6-DCBQ	Full conversion to mono-(**3a**) and difunctionalised products. No hydroquinone present. Reduced oxidant does not co-elute.

[a]Determined by ^{1}H NMR analysis of the crude mixture.

Presence of hydroquinone in this screen indicates that BQ (**1**) is still acting as an oxidant in the reaction (i.e. added oxidant is not the preferred oxidant over BQ). Of the oxidants screened, 2,6-DCBQ emerged as the best sacrificial oxidant and was thus used in the C-H difunctionalization reactions of BQ. The C-H difunctionalizations were later also evaluated with p-chloranil, p-fluoranil, $FeCl_3$ and 2,6-DCBQ as added oxidants, with 2,6-DCBQ emerging as the optimal sacrificial oxidant.

12. ^{1}H and ^{13}C NMR Spectra

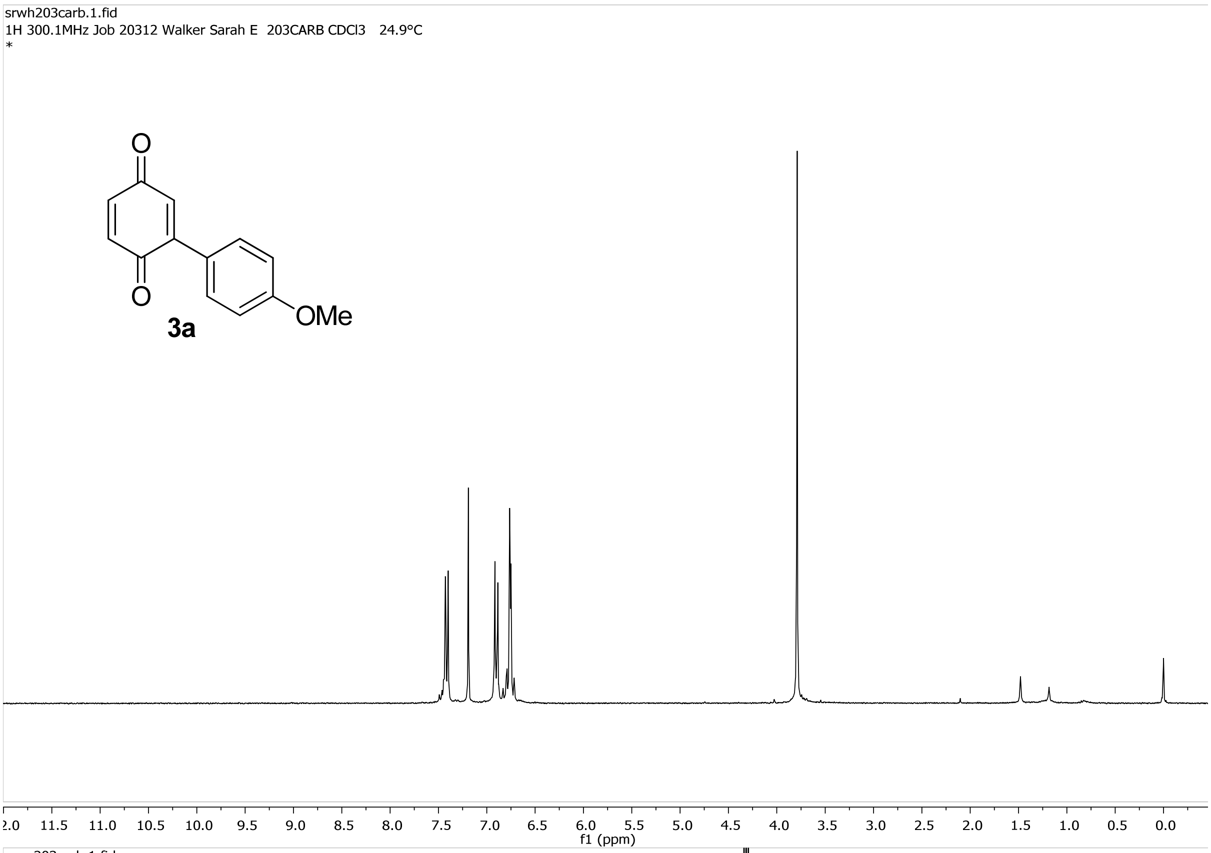

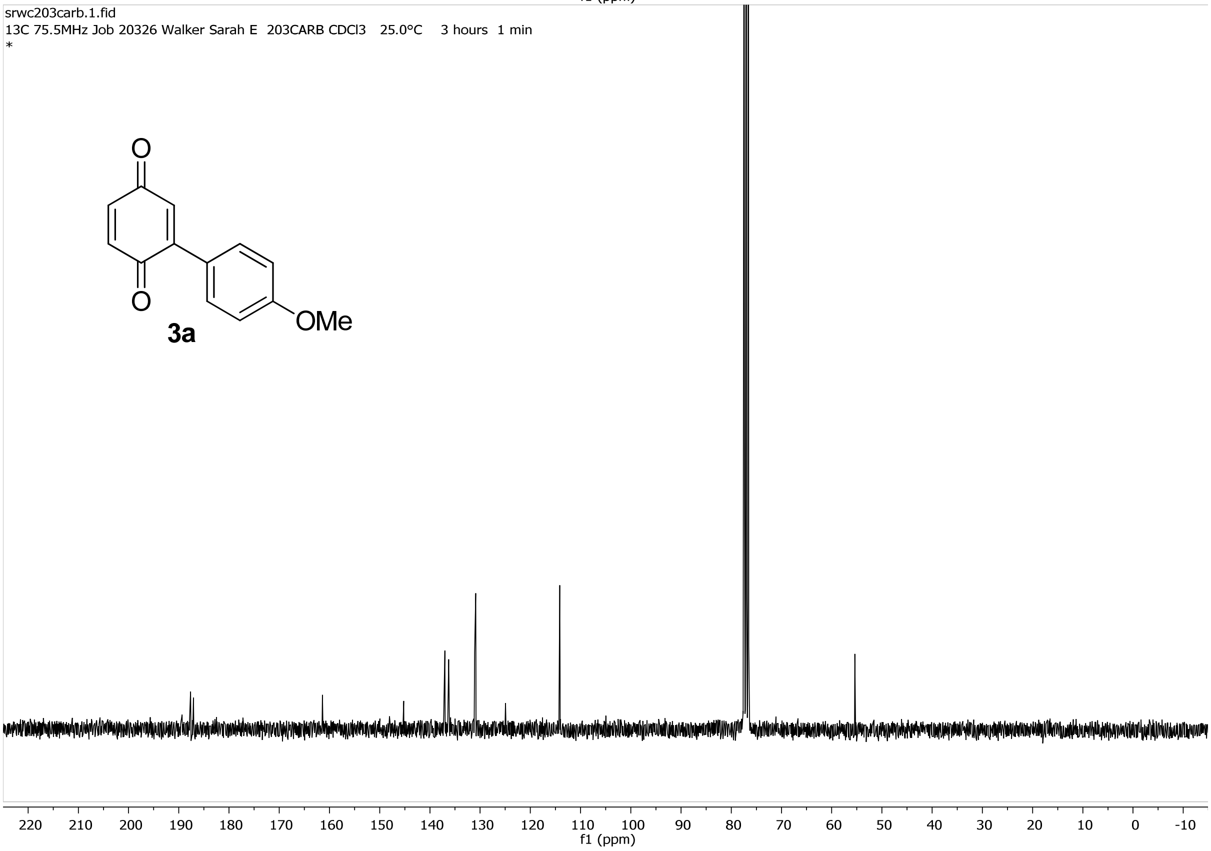

srwh363.1.fid
1H 300.1MHz Job 29555 Walker Sarah E 363 Acetone 25.0°C
*

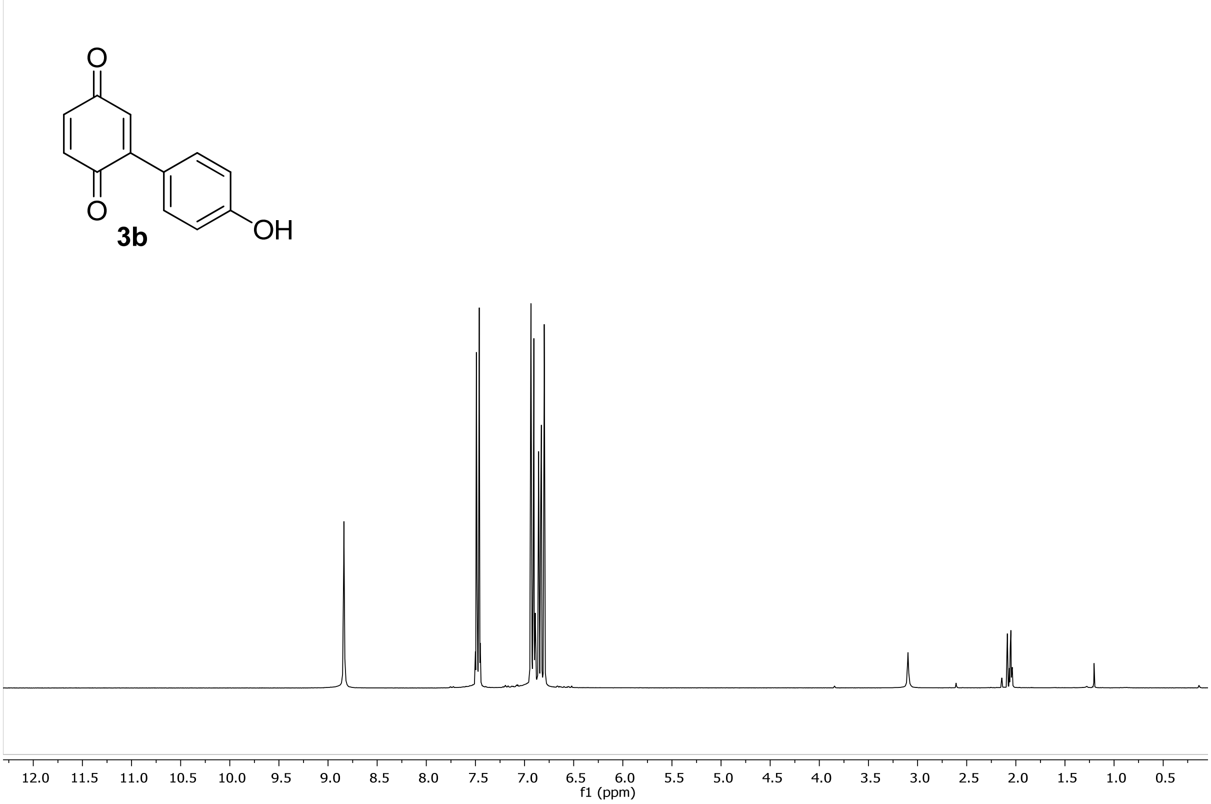

srwc363.1.fid
13C 75.5MHz Job 29559 Walker Sarah E 363 Acetone 25.0°C 0 hour 18 min
*

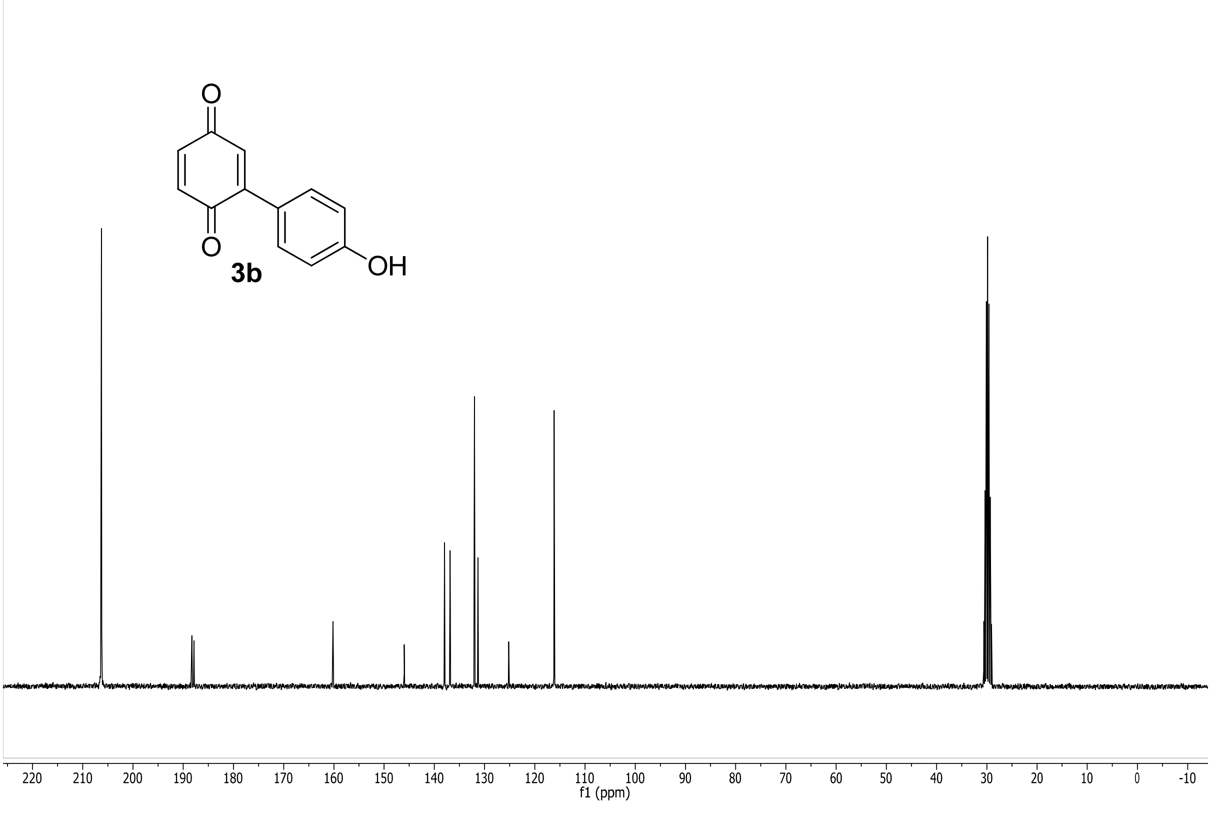

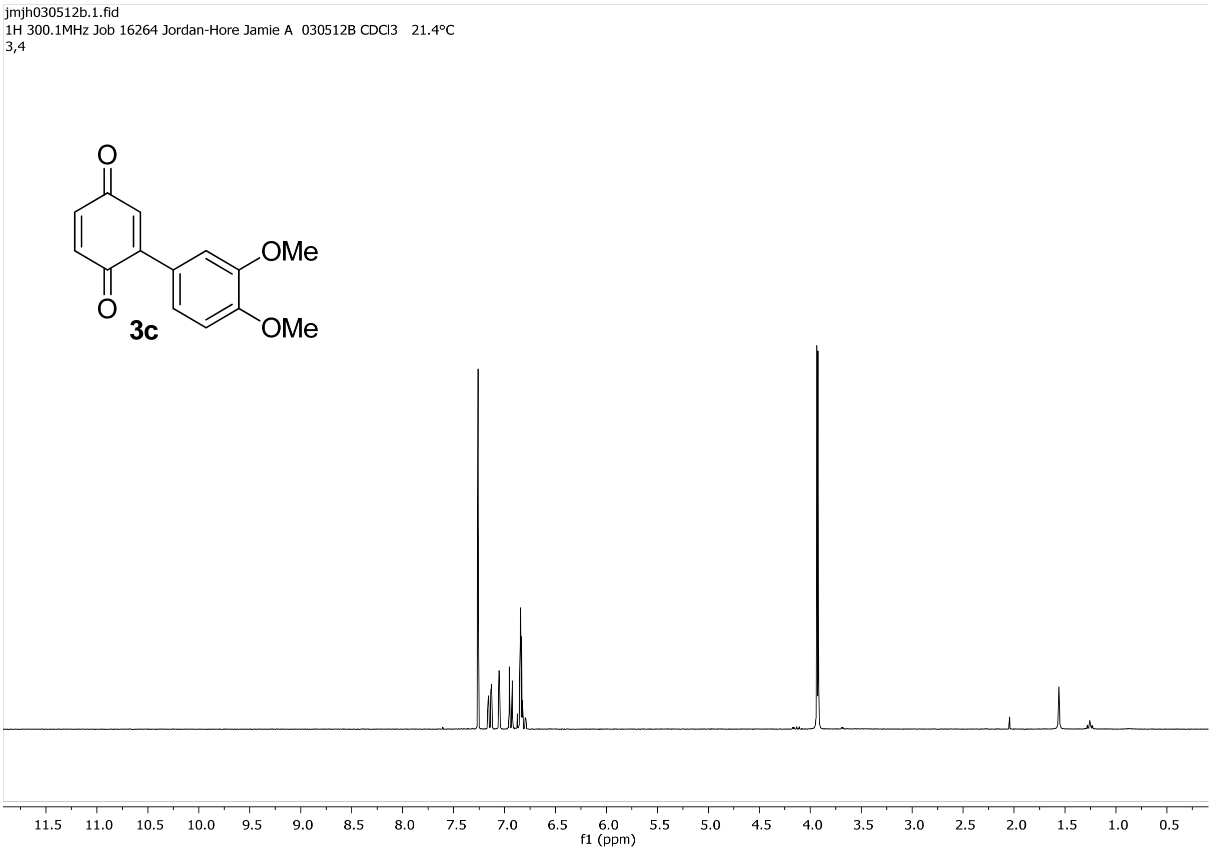
jmjh030512b.1.fid
1H 300.1MHz Job 16264 Jordan-Hore Jamie A 030512B CDCl3 21.4°C
3,4
O
O
OMe
OMe
3c
11.5 11.0 10.5 10.0 9.5 9.0 8.5 8.0 7.5 7.0 6.5 6.0 5.5 5.0 4.5 4.0 3.5 3.0 2.5 2.0 1.5 1.0 0.5
f1 (ppm)

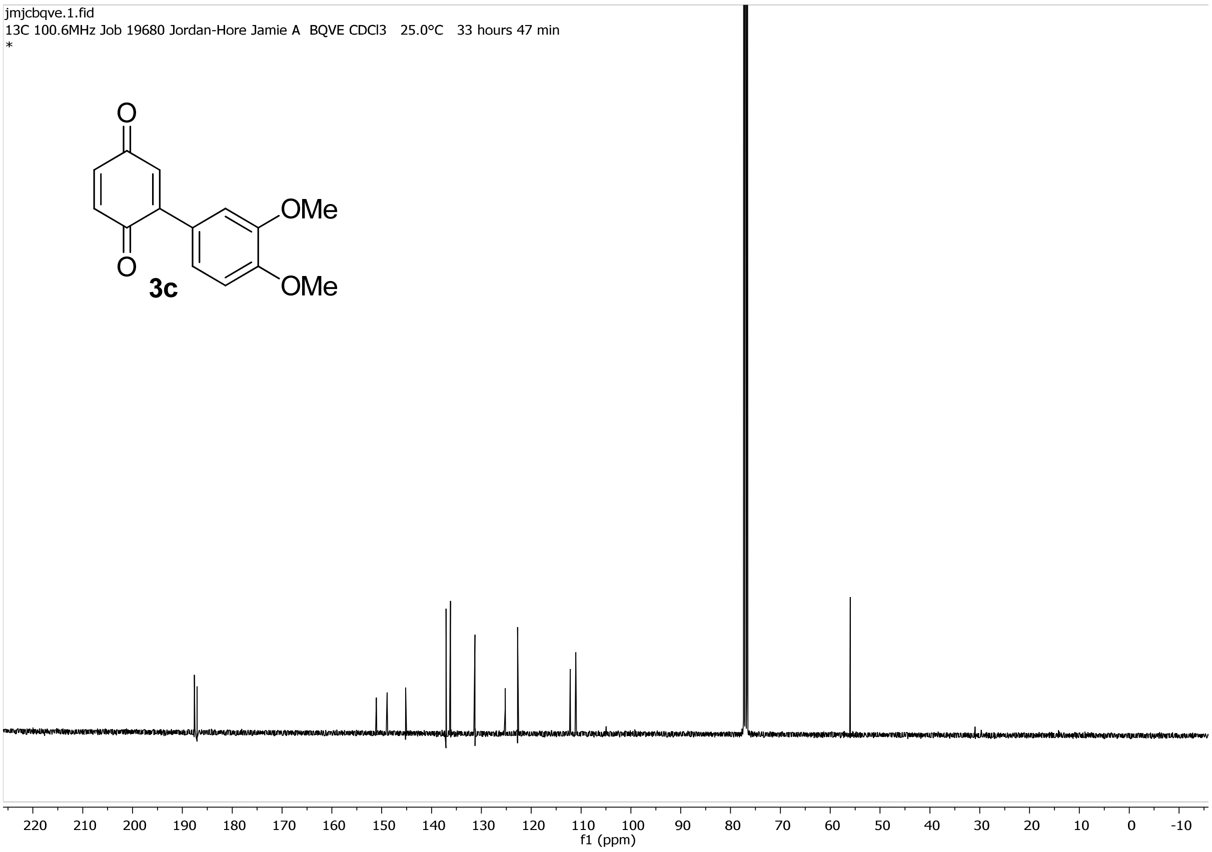
jmjcbqve.1.fid
13C 100.6MHz Job 19680 Jordan-Hore Jamie A BQVE CDCl3 25.0°C 33 hours 47 min
*
O
O
OMe
OMe
3c
220 210 200 190 180 170 160 150 140 130 120 110 100 90 80 70 60 50 40 30 20 10 0 -10
f1 (ppm)

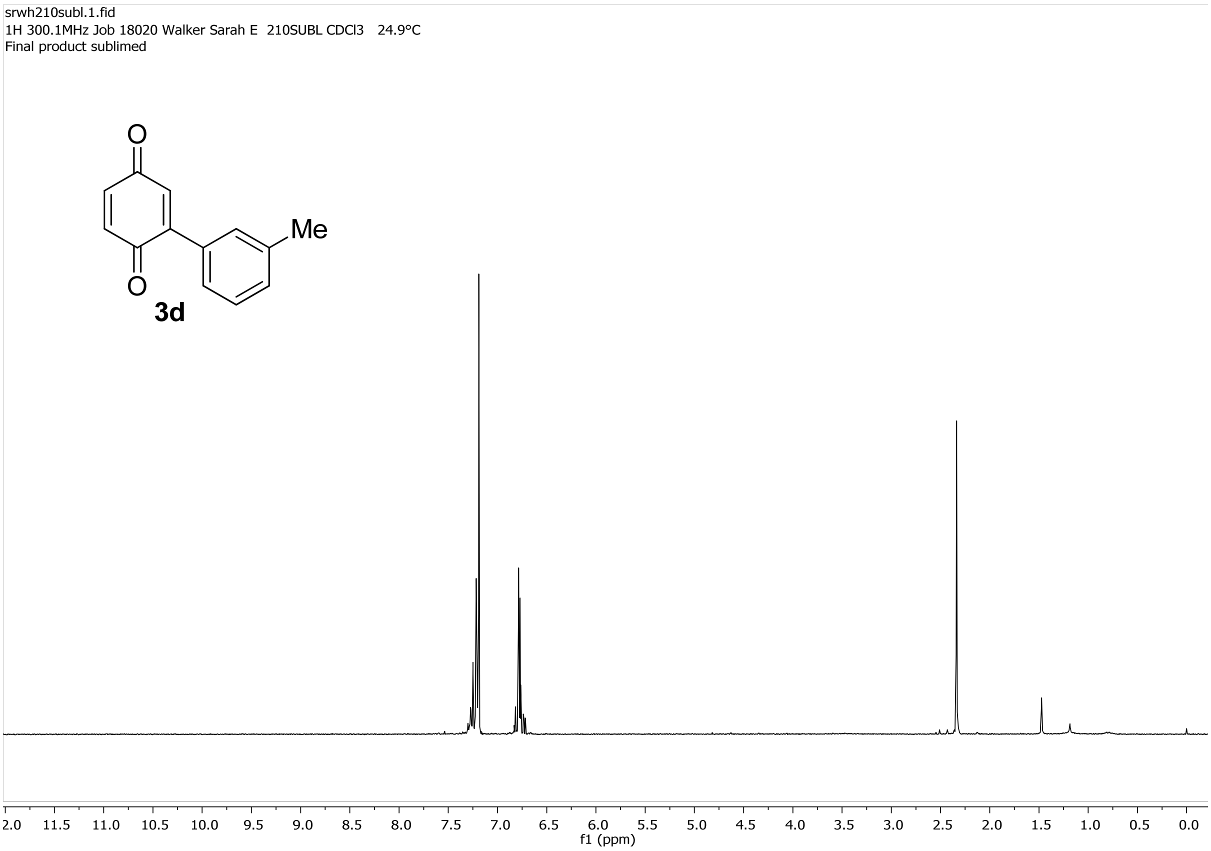
srwh210subl.1.fid
1H 300.1MHz Job 18020 Walker Sarah E 210SUBL CDCl3 24.9°C
Final product sublimed
Me
3d
f1 (ppm)

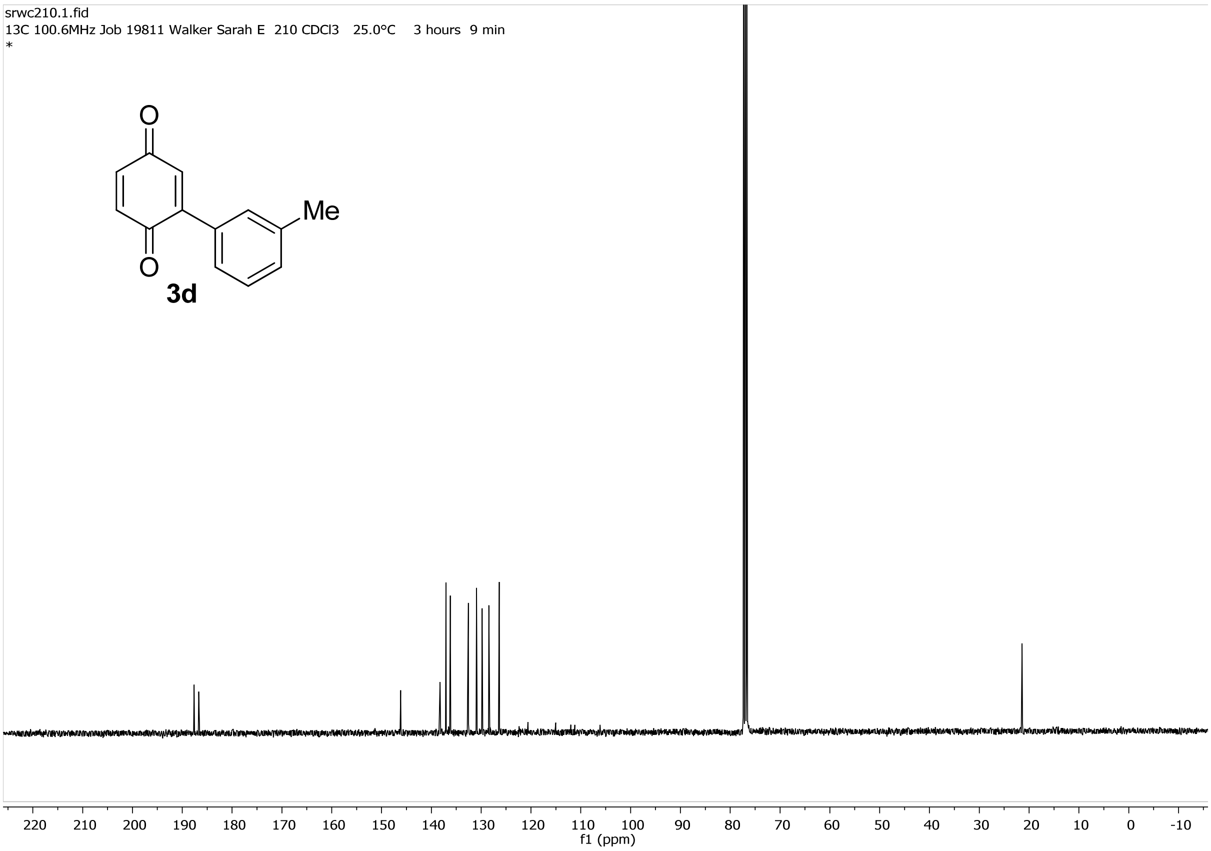
srwc210.1.fid
13C 100.6MHz Job 19811 Walker Sarah E 210 CDCl3 25.0°C 3 hours 9 min
*
Me
3d
f1 (ppm)

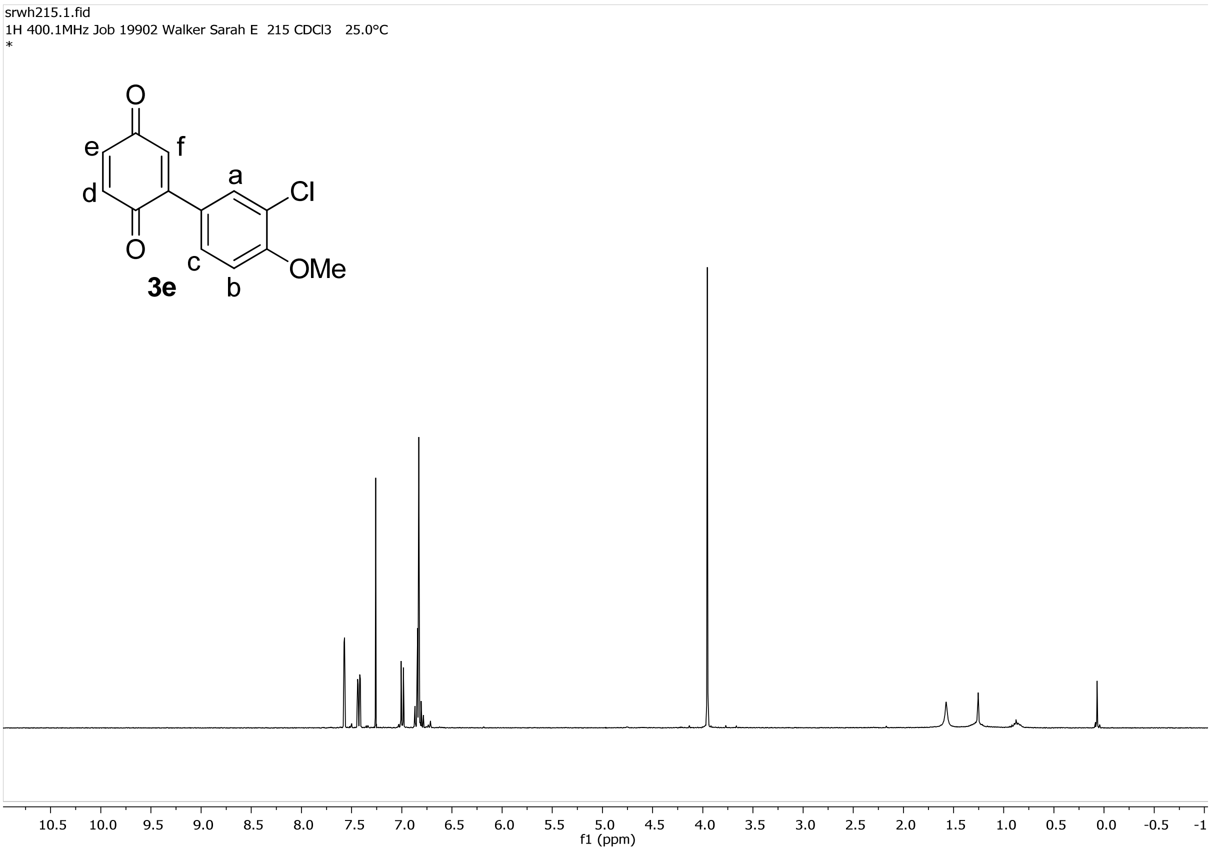
srwh215.1.fid
1H 400.1MHz Job 19902 Walker Sarah E 215 CDCl3 25.0°C
O
e
f
a
Cl
d
O
c
OMe
3e
b
10.5 10.0 9.5 9.0 8.5 8.0 7.5 7.0 6.5 6.0 5.5 5.0 4.5 4.0 3.5 3.0 2.5 2.0 1.5 1.0 0.5 0.0 -0.5 -1
f1 (ppm)

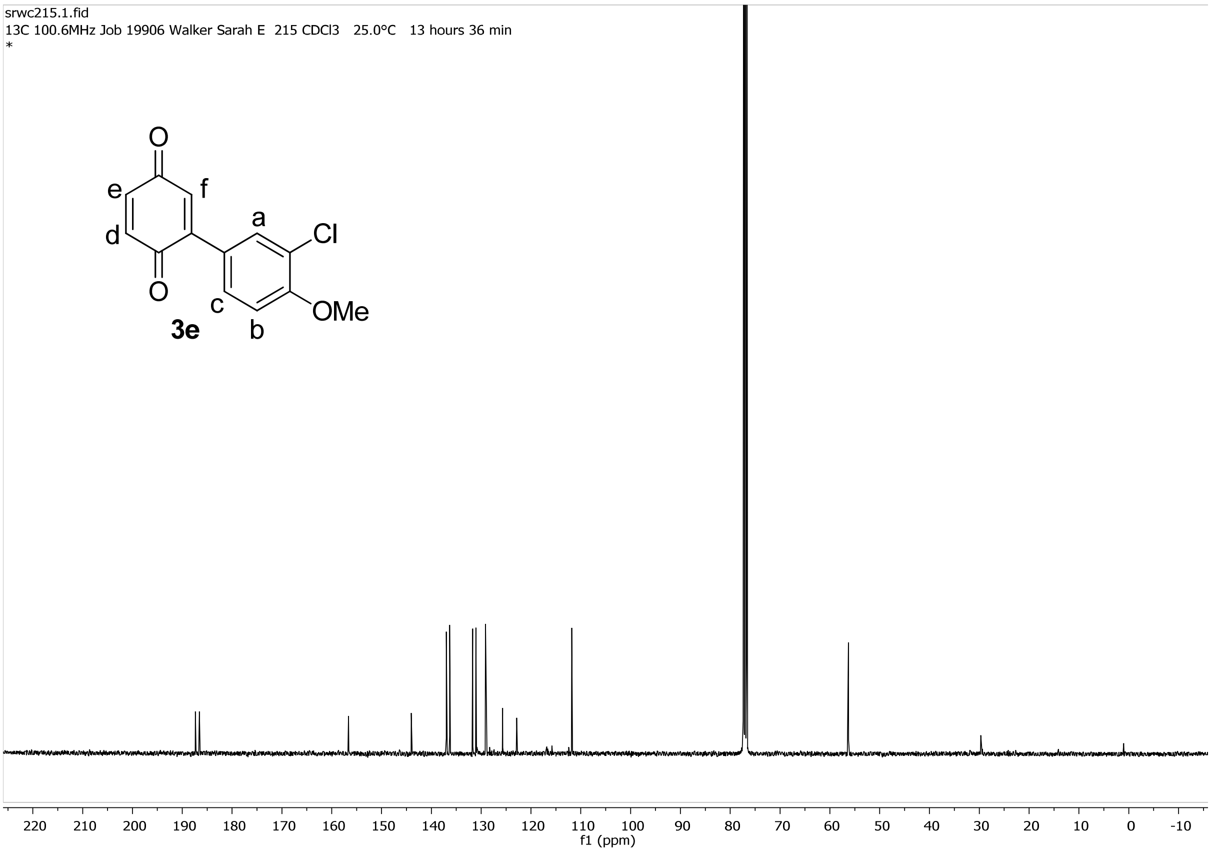
srwc215.1.fid
13C 100.6MHz Job 19906 Walker Sarah E 215 CDCl3 25.0°C 13 hours 36 min
O
e
f
a
Cl
d
O
c
OMe
3e
b
220 210 200 190 180 170 160 150 140 130 120 110 100 90 80 70 60 50 40 30 20 10 0 -10
f1 (ppm)

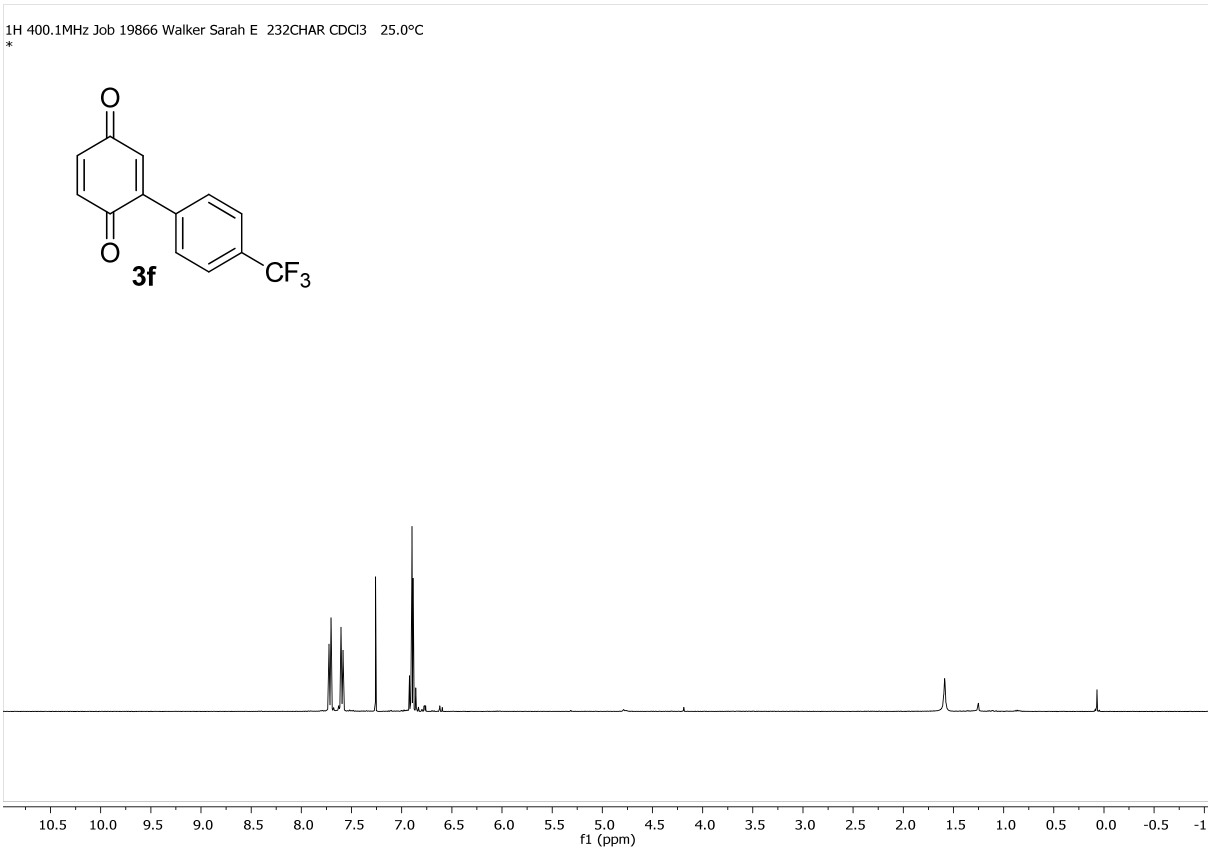
1H 400.1MHz Job 19866 Walker Sarah E 232CHAR CDCl3   25.0°C
*
3f
f1 (ppm)

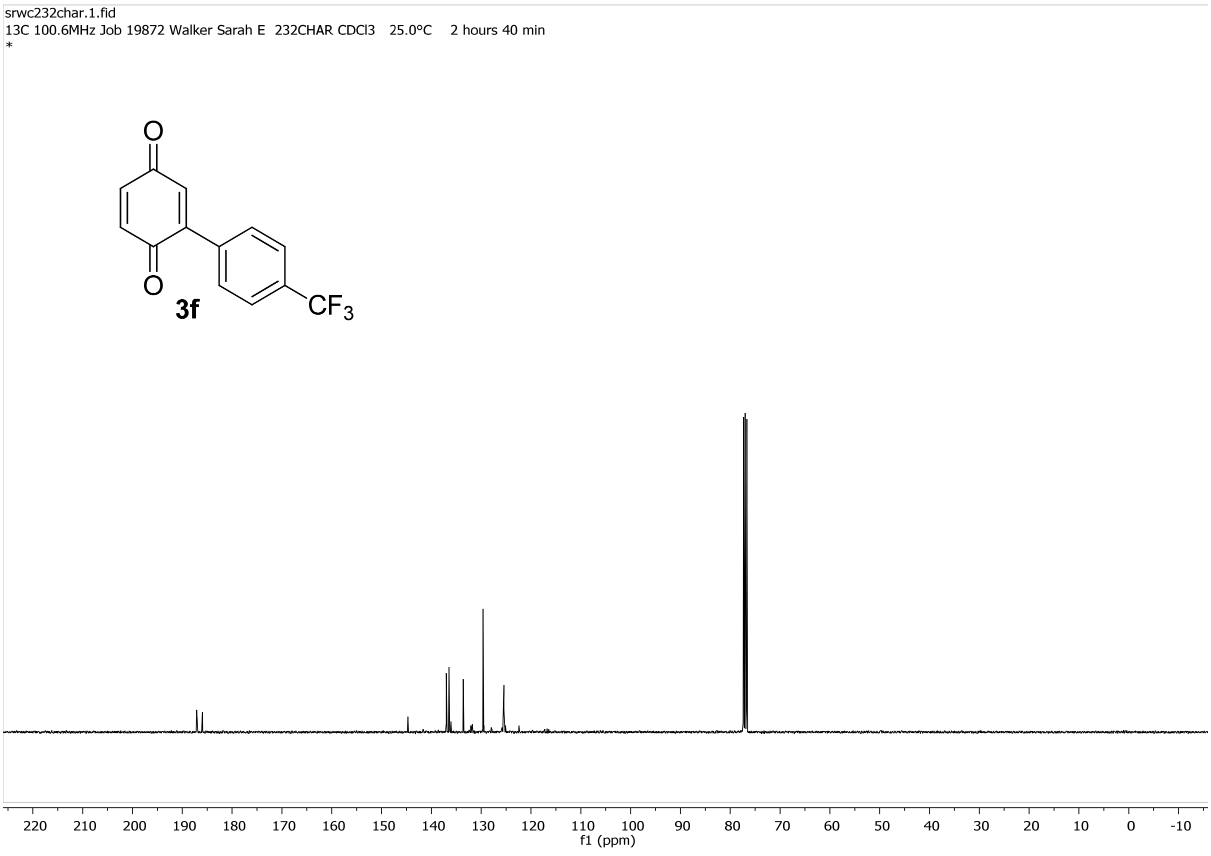
srwc232char.1.fid
13C 100.6MHz Job 19872 Walker Sarah E 232CHAR CDCl3   25.0°C   2 hours 40 min
*
3f
f1 (ppm)

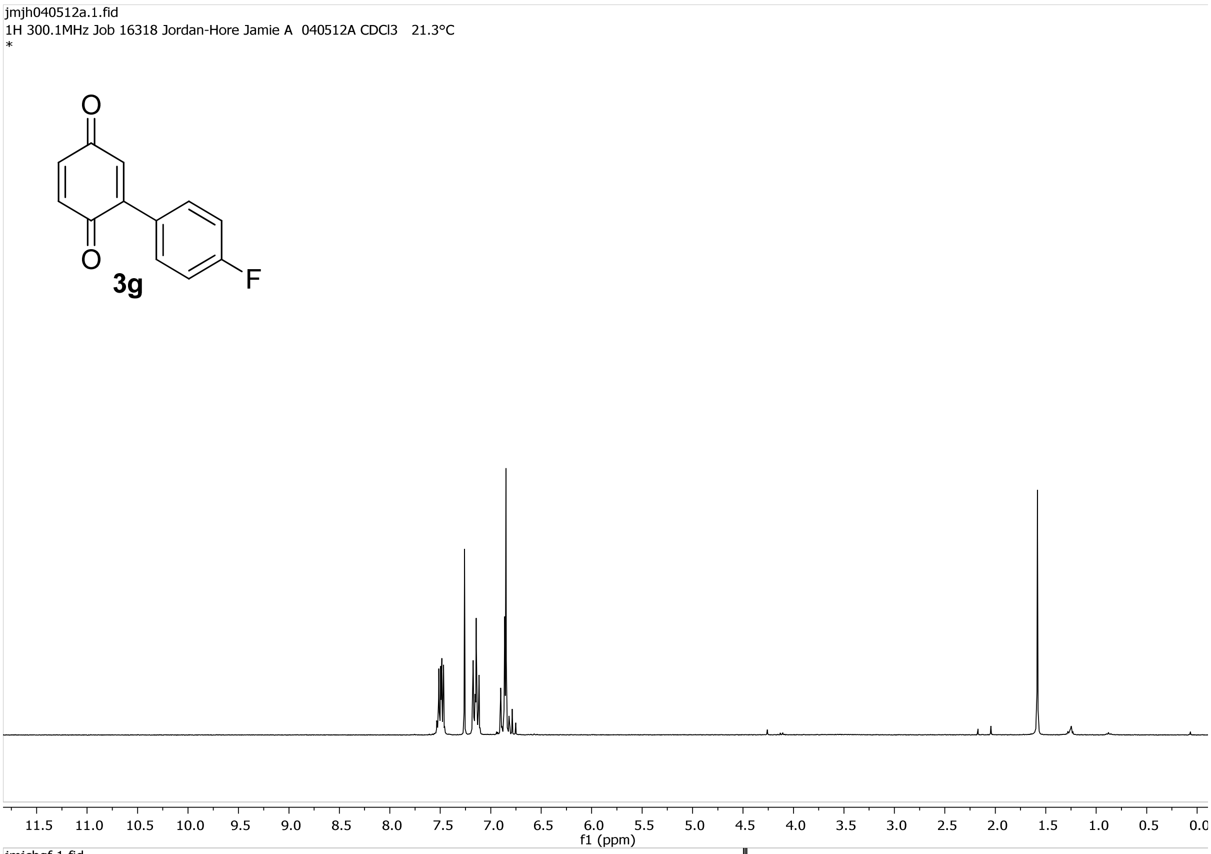
jmjh040512a.1.fid
1H 300.1MHz Job 16318 Jordan-Hore Jamie A 040512A CDCl3 21.3°C
*
O
O
3g
F
11.5 11.0 10.5 10.0 9.5 9.0 8.5 8.0 7.5 7.0 6.5 6.0 5.5 5.0 4.5 4.0 3.5 3.0 2.5 2.0 1.5 1.0 0.5 0.0
f1 (ppm)

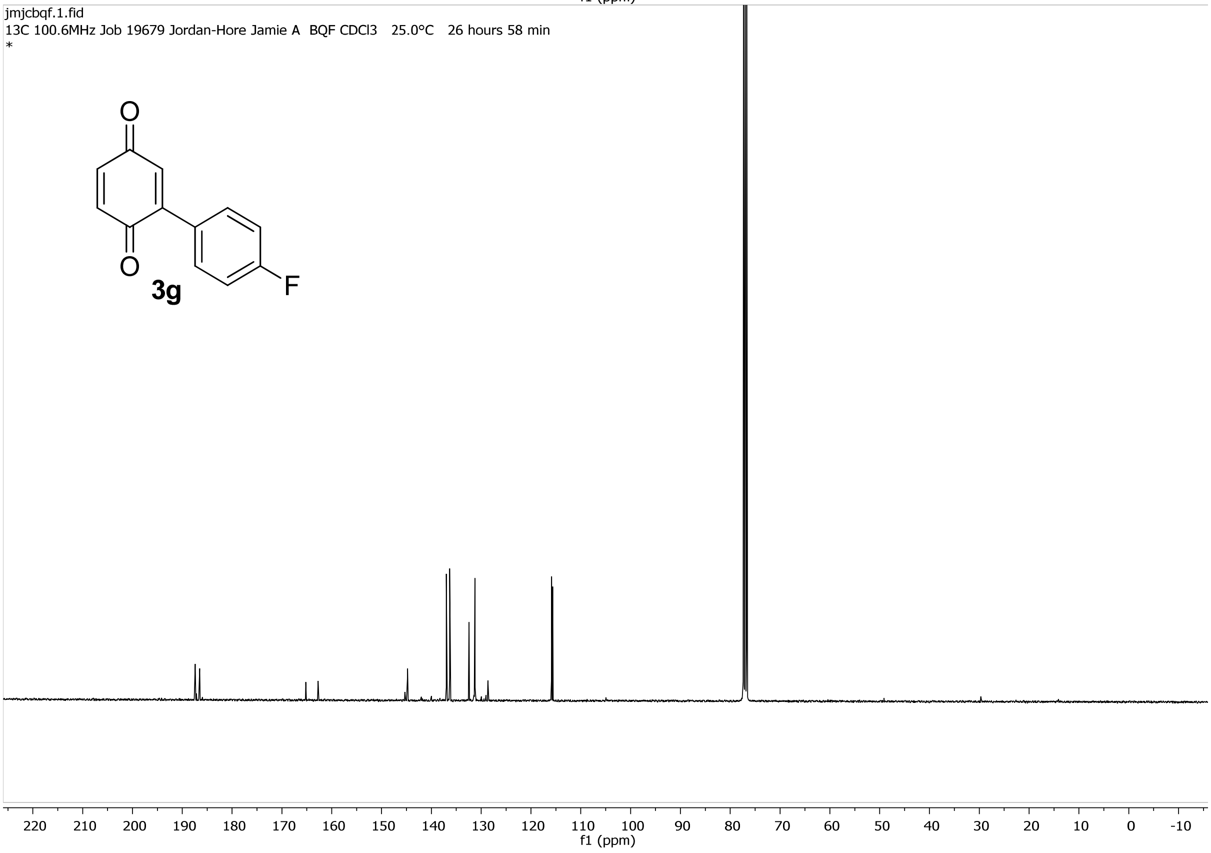
jmjcbqf.1.fid
13C 100.6MHz Job 19679 Jordan-Hore Jamie A BQF CDCl3 25.0°C 26 hours 58 min
*
O
O
3g
F
220 210 200 190 180 170 160 150 140 130 120 110 100 90 80 70 60 50 40 30 20 10 0 -10
f1 (ppm)

srwhbq008col.1.fid
1H 300.1MHz Job 20352 Walker Sarah E BQ008COL CDCl3 24.9°C
Product after column tubes 12-16 yellow spot

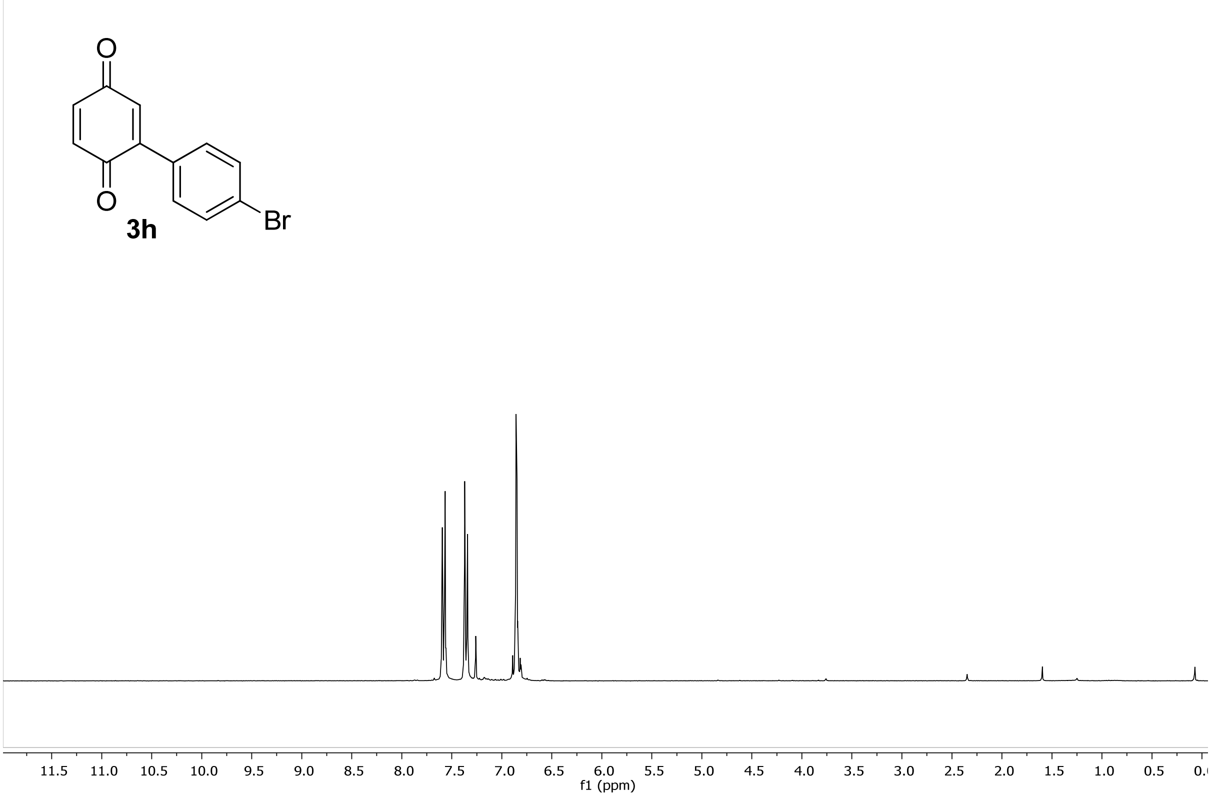

srwc231.1.fid
13C 100.6MHz Job 19816 Walker Sarah E 231 CDCl3 25.0°C 5 hours 6 min
*

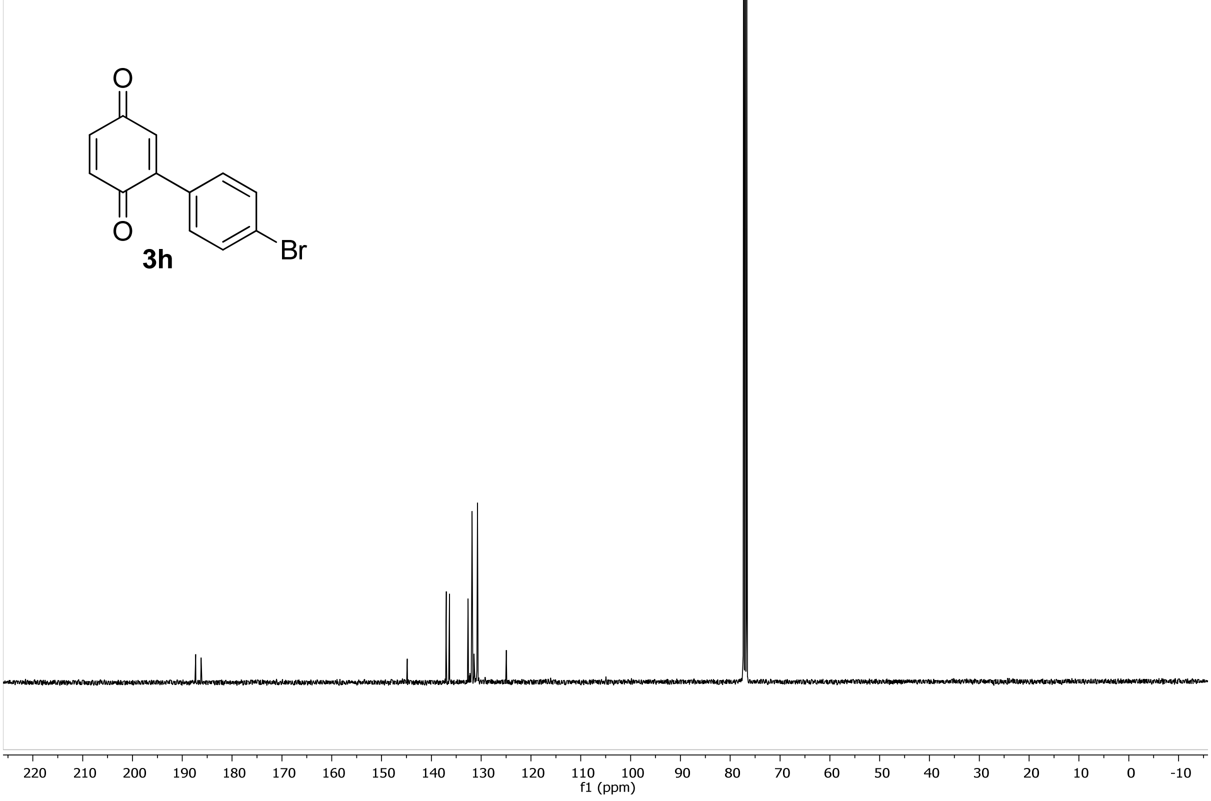

srwhbq005c.1.fid
1H 300.1MHz Job 20168 Walker Sarah E BQ005C CDCl3   25.0°C
Orange rather than green sample

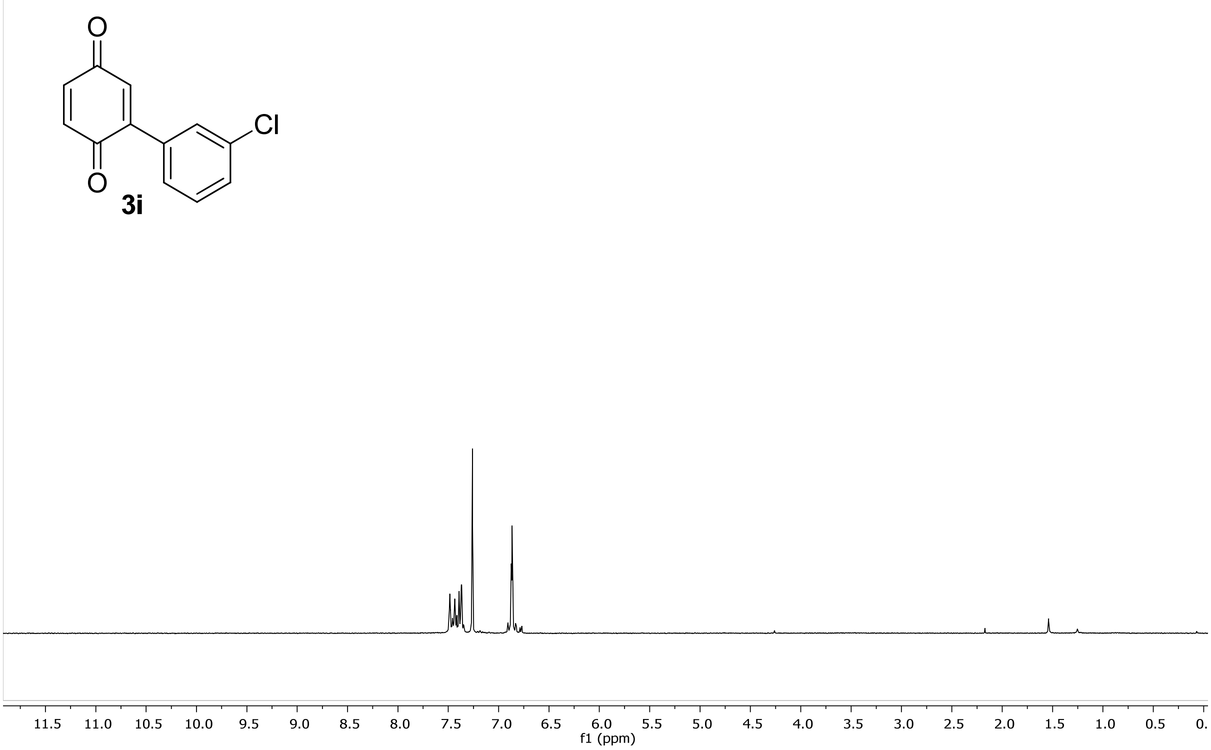

srwc005b.1.fid
13C 100.6MHz Job 19918 Walker Sarah E 005B CDCl3   25.0°C   63 hours 40 min
*

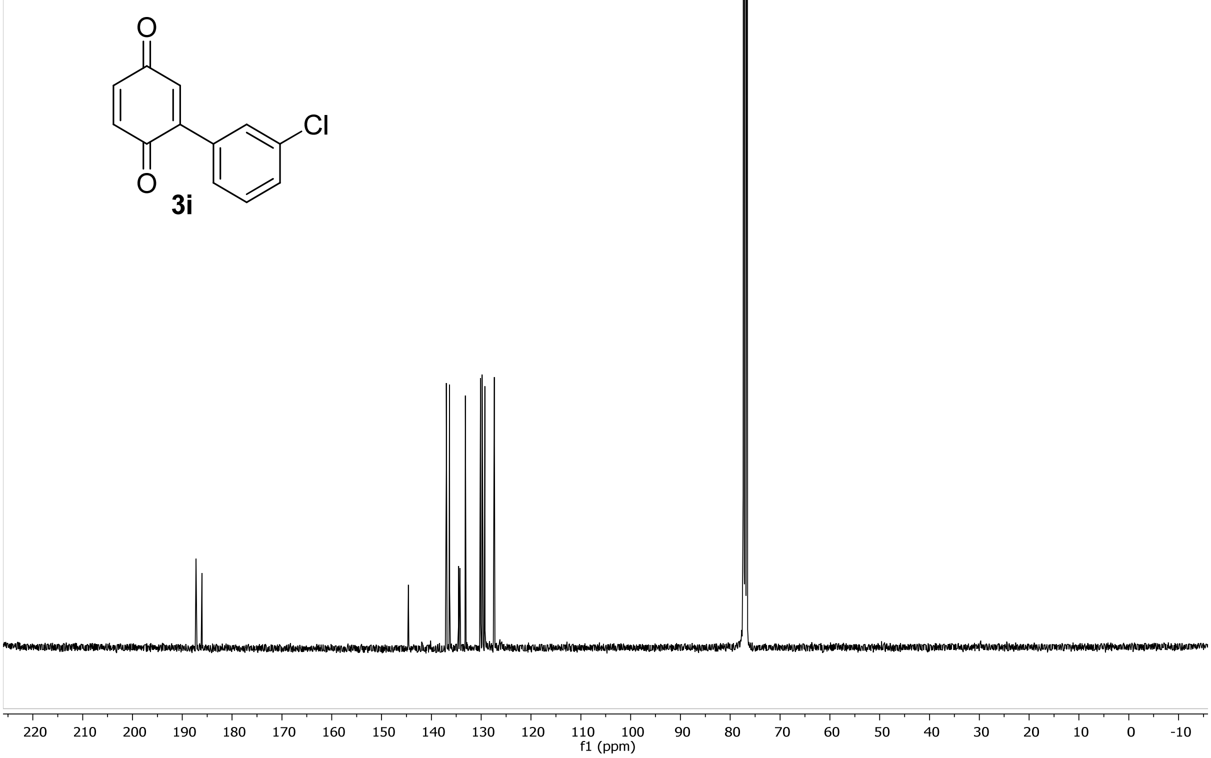

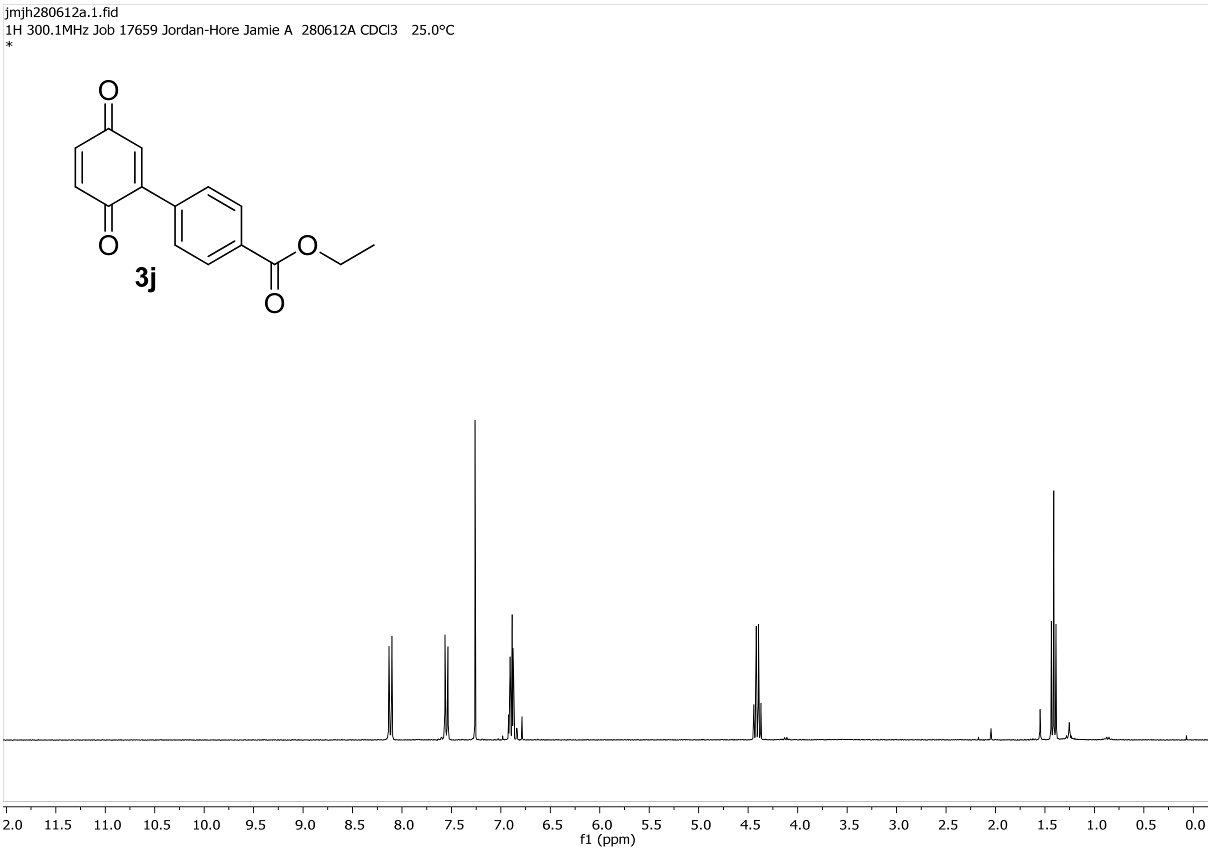
jmjh280612a.1.fid
1H 300.1MHz Job 17659 Jordan-Hore Jamie A 280612A CDCl3 25.0°C
*
3j
2.0 11.5 11.0 10.5 10.0 9.5 9.0 8.5 8.0 7.5 7.0 6.5 6.0 5.5 5.0 4.5 4.0 3.5 3.0 2.5 2.0 1.5 1.0 0.5 0.0
f1 (ppm)

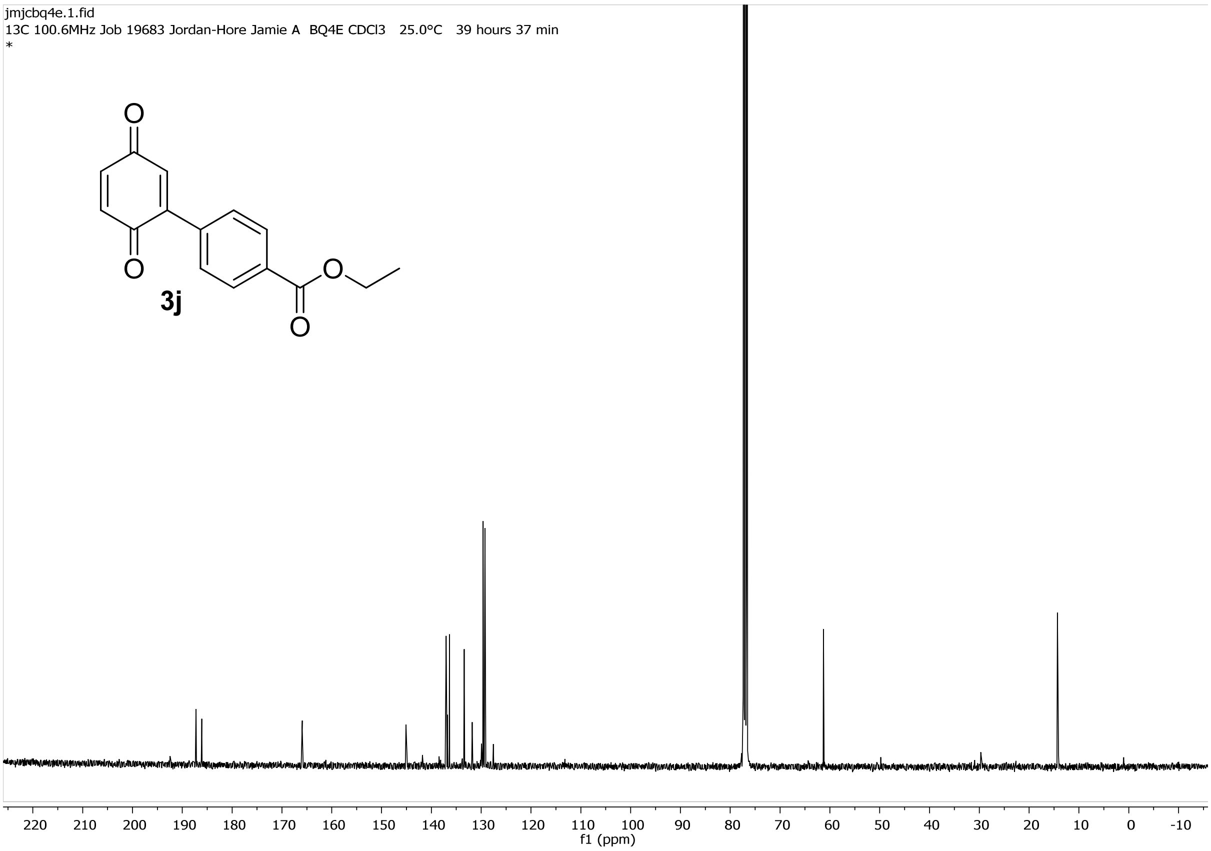
jmjcbq4e.1.fid
13C 100.6MHz Job 19683 Jordan-Hore Jamie A BQ4E CDCl3 25.0°C 39 hours 37 min
*
3j
220 210 200 190 180 170 160 150 140 130 120 110 100 90 80 70 60 50 40 30 20 10 0 -10
f1 (ppm)

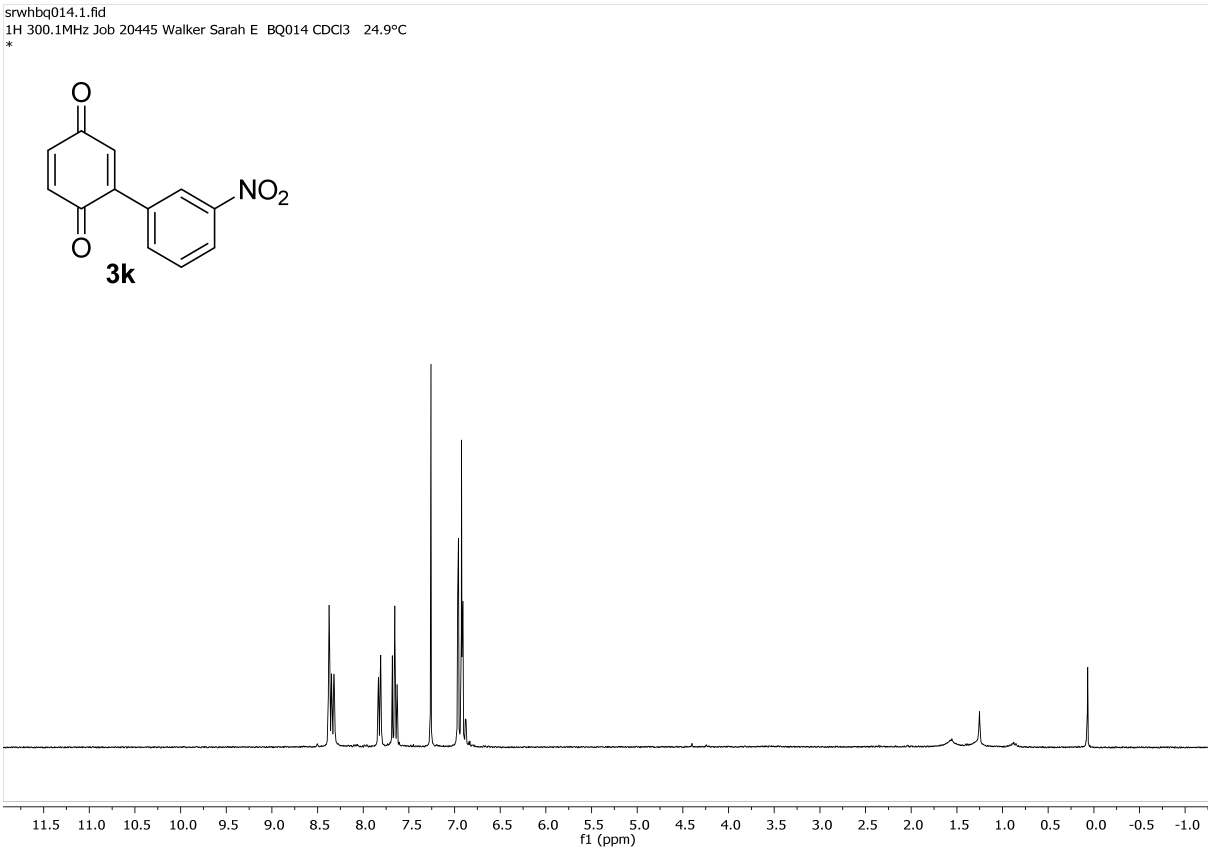
srwhbq014.1.fid
1H 300.1MHz Job 20445 Walker Sarah E BQ014 CDCl3 24.9°C
*
NO2
3k
11.5 11.0 10.5 10.0 9.5 9.0 8.5 8.0 7.5 7.0 6.5 6.0 5.5 5.0 4.5 4.0 3.5 3.0 2.5 2.0 1.5 1.0 0.5 0.0 -0.5 -1.0
f1 (ppm)

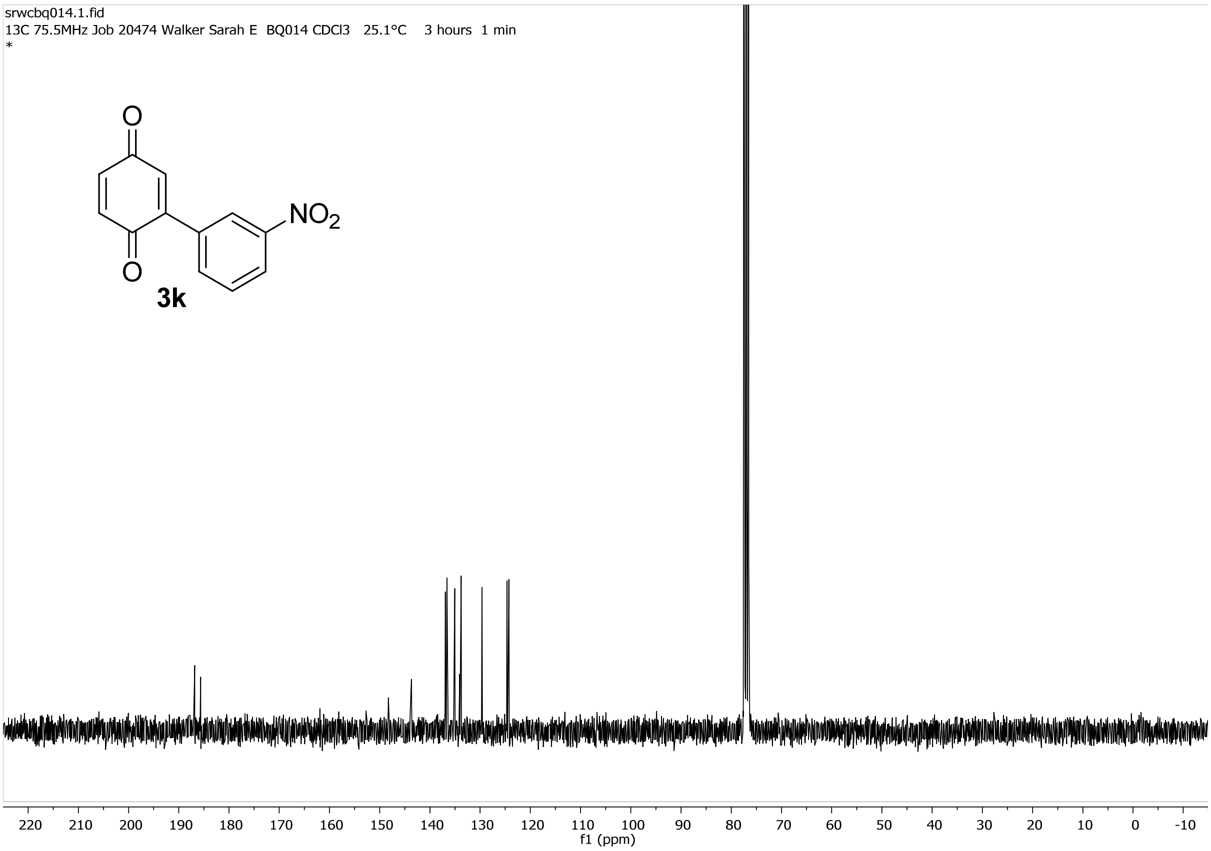
srwcbq014.1.fid
13C 75.5MHz Job 20474 Walker Sarah E BQ014 CDCl3 25.1°C 3 hours 1 min
*
NO2
3k
220 210 200 190 180 170 160 150 140 130 120 110 100 90 80 70 60 50 40 30 20 10 0 -10
f1 (ppm)

jmjh170712a.1.fid
1H 300.1MHz Job 18159 Jordan-Hore Jamie A 170712A CDCl3 25.0°C
mes BQ mono

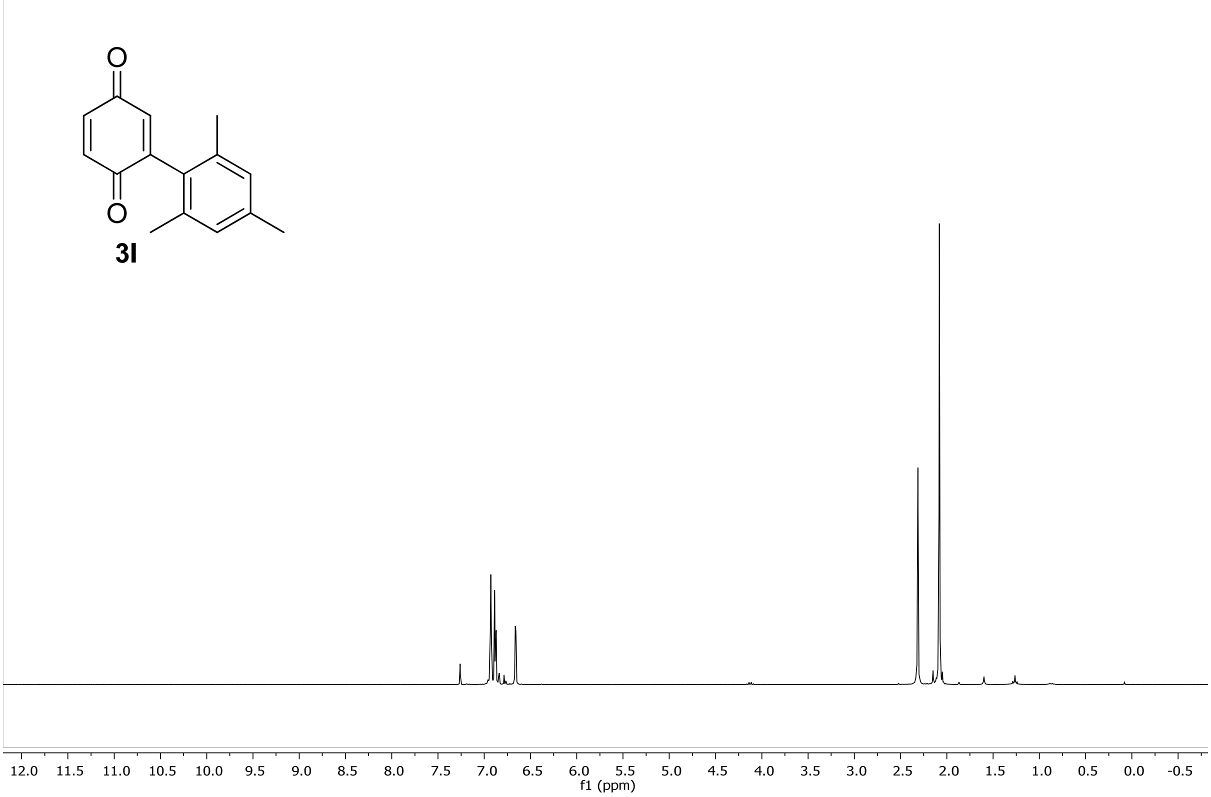

srwcbq011.1.fid
13C 75.5MHz Job 20435 Walker Sarah E BQ011 CDCl3 25.0°C 0 hour 18 min
*

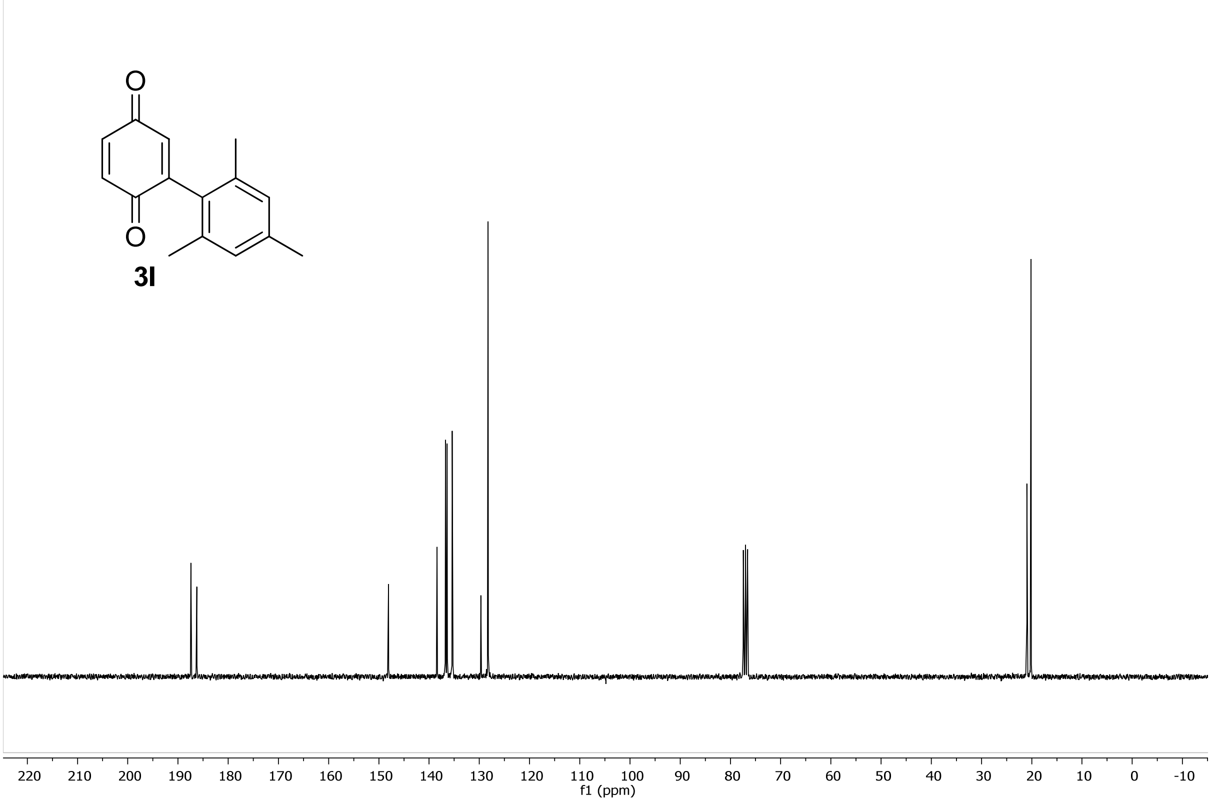

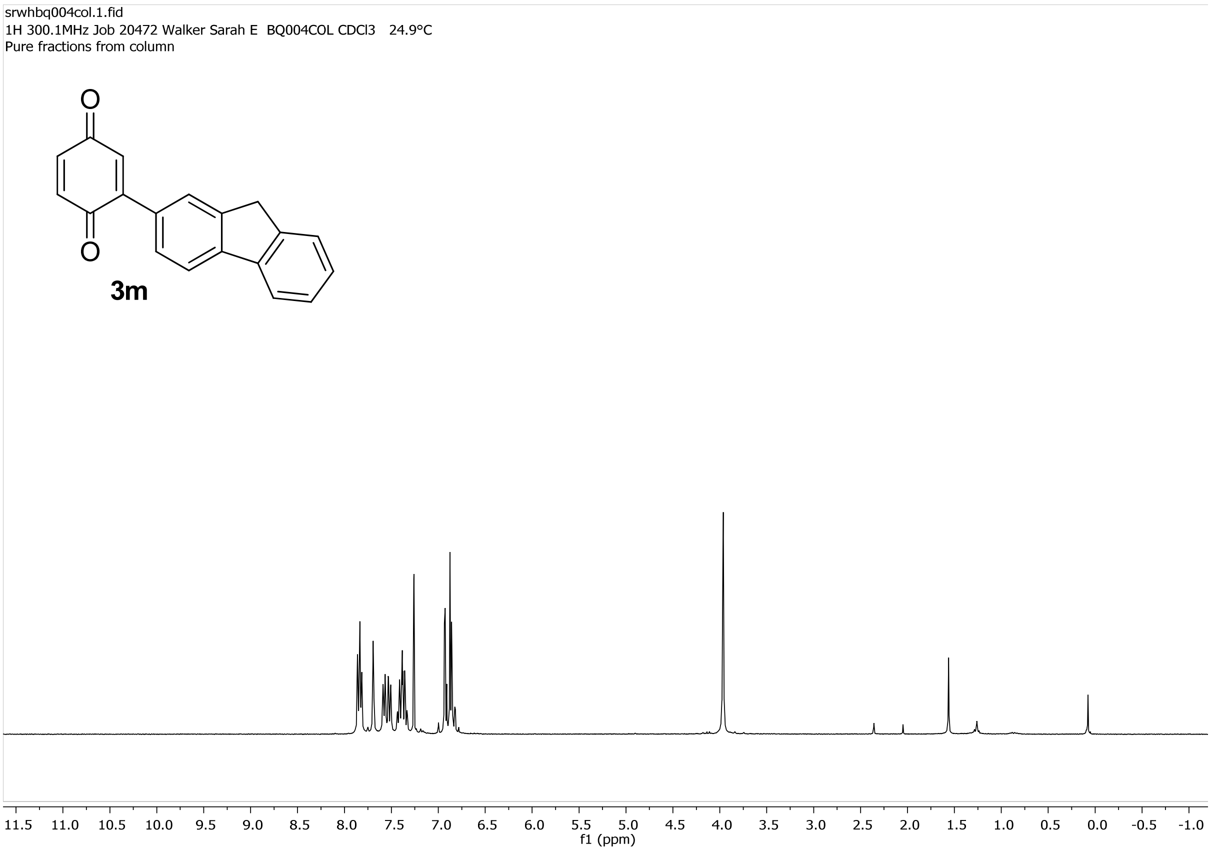
srwhbq004col.1.fid
1H 300.1MHz Job 20472 Walker Sarah E BQ004COL CDCl3 24.9°C
Pure fractions from column
3m
11.5 11.0 10.5 10.0 9.5 9.0 8.5 8.0 7.5 7.0 6.5 6.0 5.5 5.0 4.5 4.0 3.5 3.0 2.5 2.0 1.5 1.0 0.5 0.0 -0.5 -1.0
f1 (ppm)

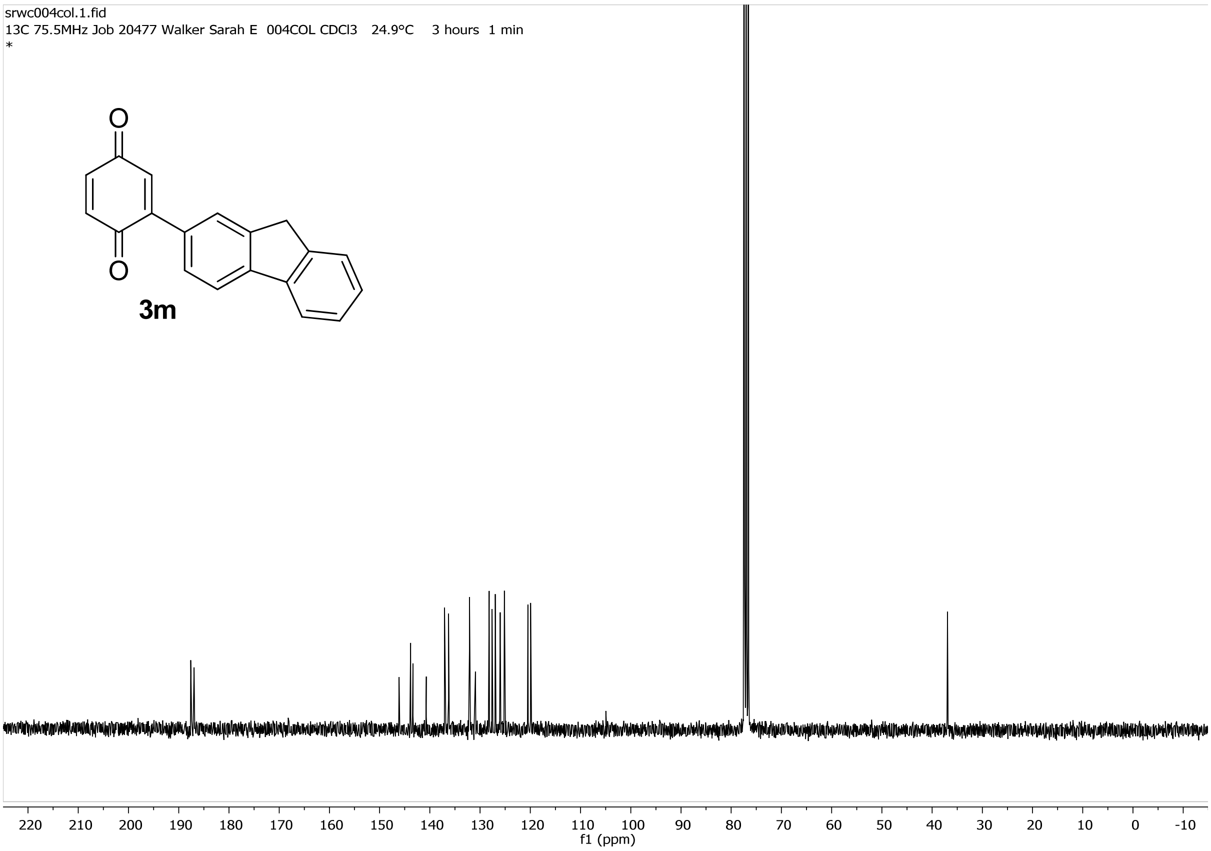
srwc004col.1.fid
13C 75.5MHz Job 20477 Walker Sarah E 004COL CDCl3 24.9°C 3 hours 1 min
*
3m
220 210 200 190 180 170 160 150 140 130 120 110 100 90 80 70 60 50 40 30 20 10 0 -10
f1 (ppm)

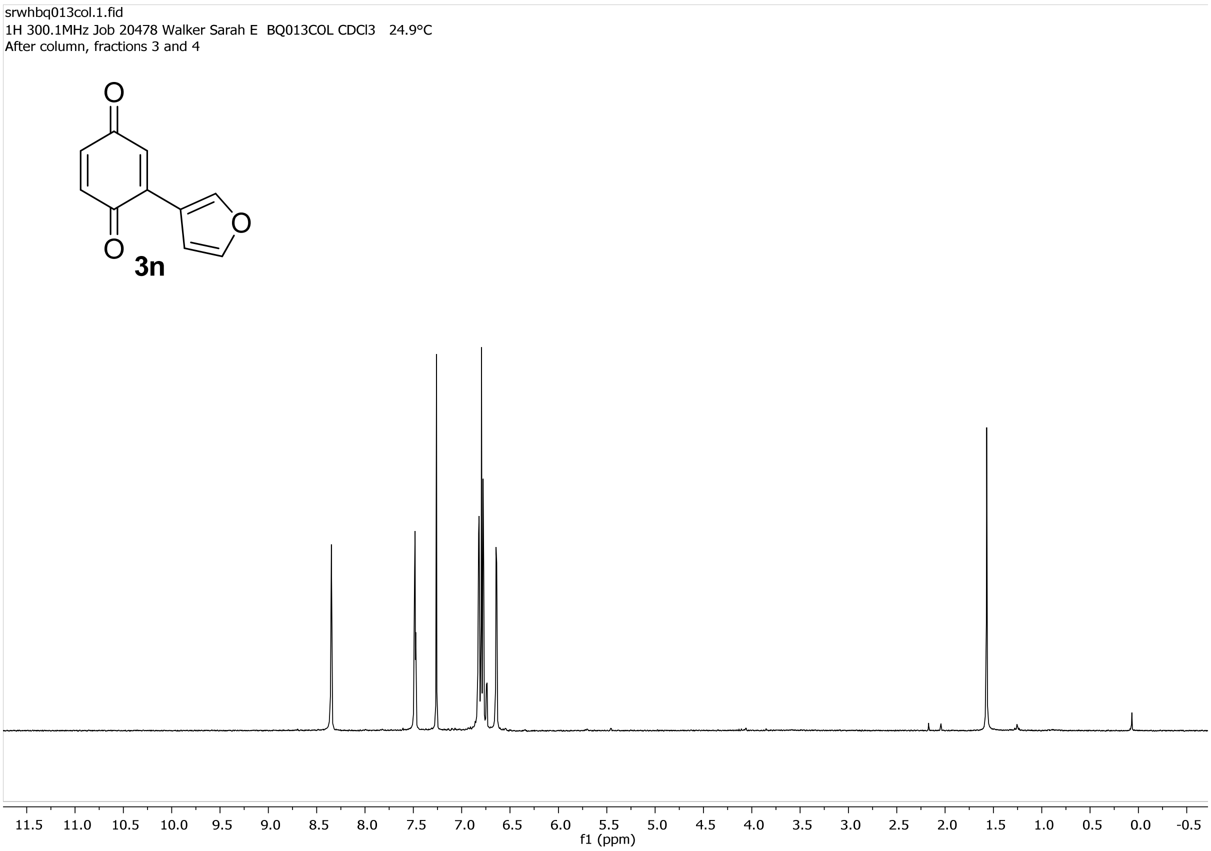
srwhbq013col.1.fid
1H 300.1MHz Job 20478 Walker Sarah E BQ013COL CDCl3 24.9°C
After column, fractions 3 and 4
3n
11.5 11.0 10.5 10.0 9.5 9.0 8.5 8.0 7.5 7.0 6.5 6.0 5.5 5.0 4.5 4.0 3.5 3.0 2.5 2.0 1.5 1.0 0.5 0.0 -0.5
f1 (ppm)

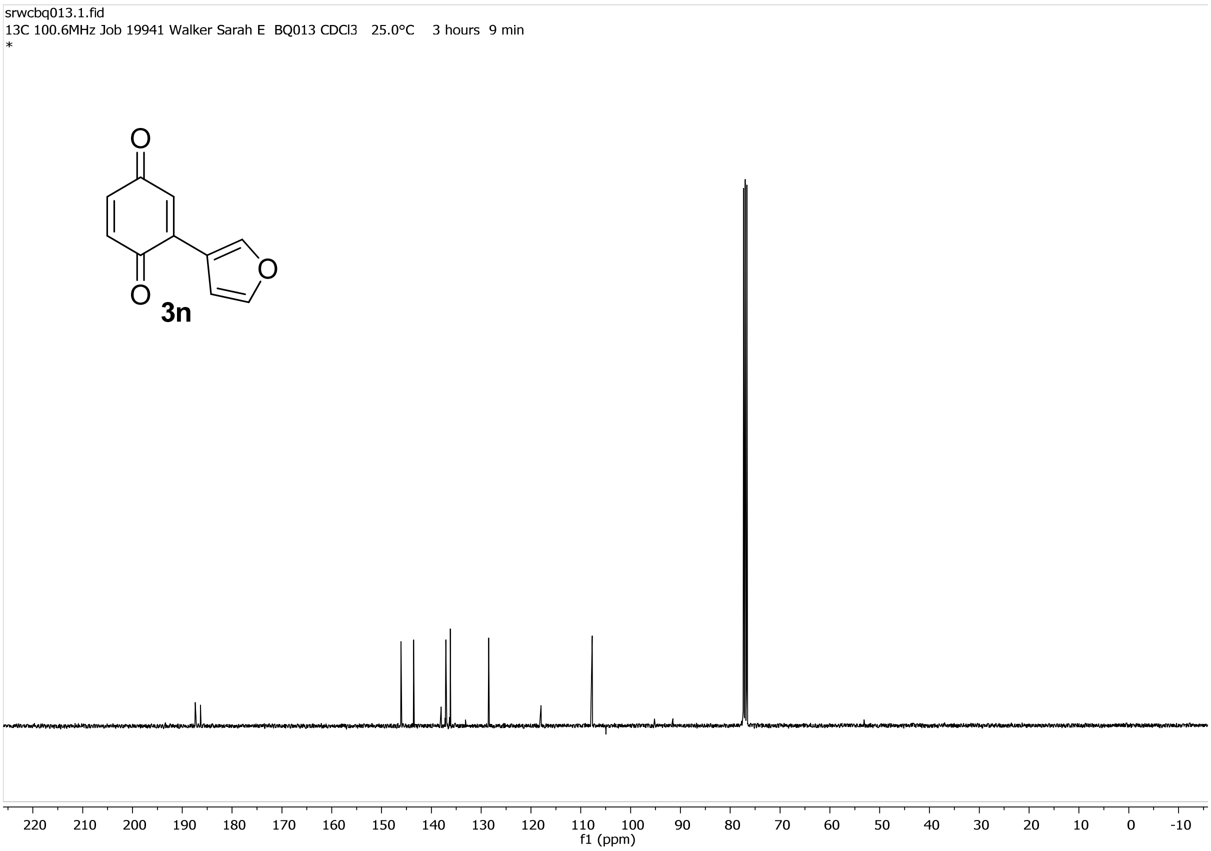
srwcbq013.1.fid
13C 100.6MHz Job 19941 Walker Sarah E BQ013 CDCl3 25.0°C 3 hours 9 min
*
3n
220 210 200 190 180 170 160 150 140 130 120 110 100 90 80 70 60 50 40 30 20 10 0 -10
f1 (ppm)

srwh315a31H 300.1MHz Job 26824 Walker Sarah E 315A3 CDCl3 25.1°C
*

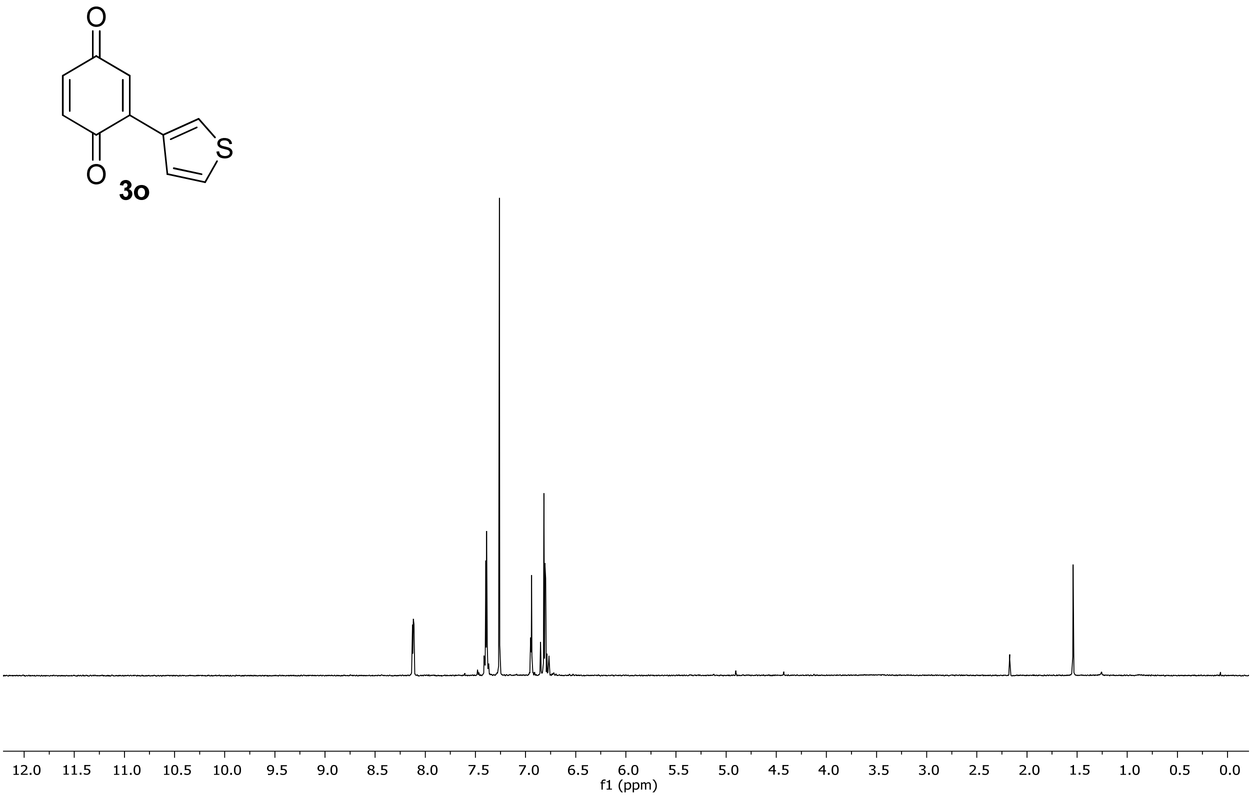

srwc315a313C 75.5MHz Job 27194 Walker Sarah E 315a3 CDCl3 25.0°C 8 hours 17 min

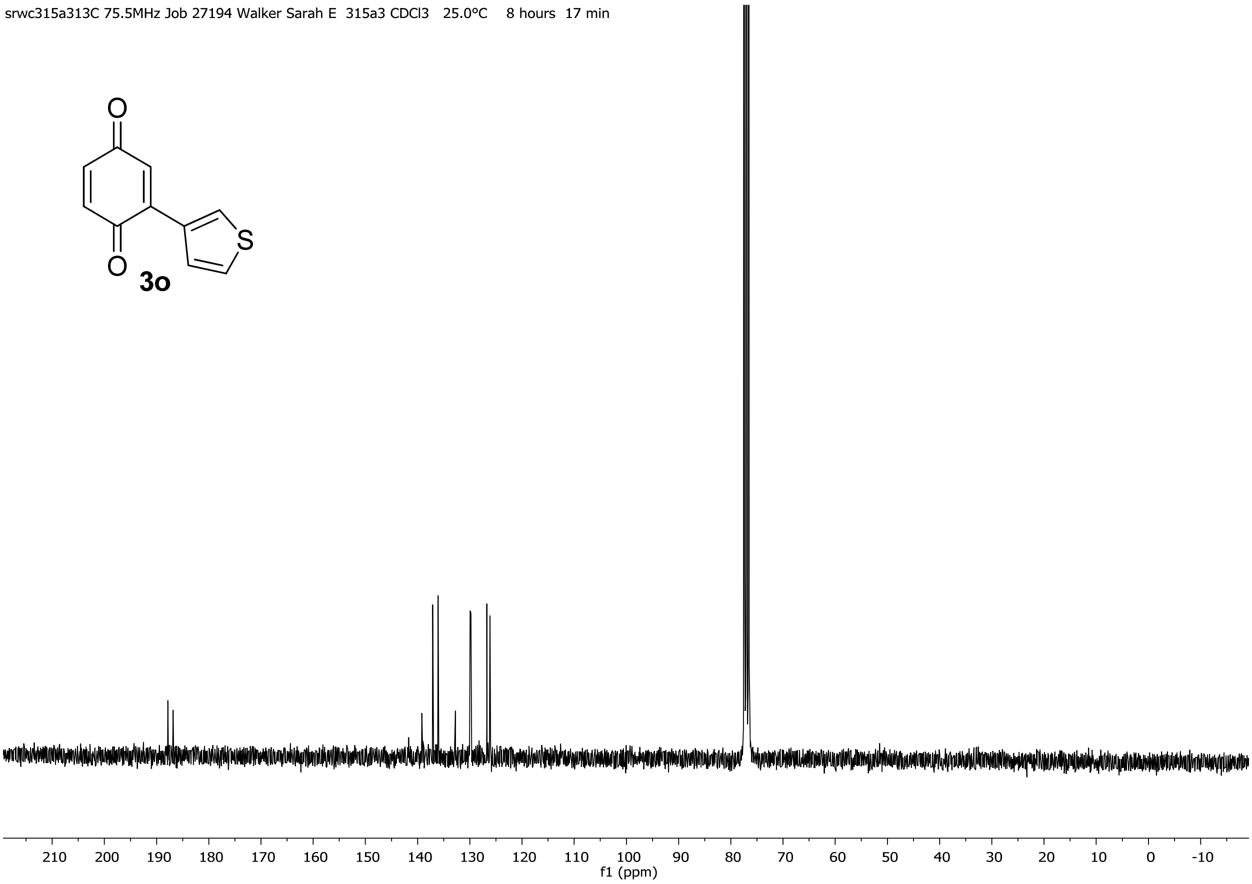

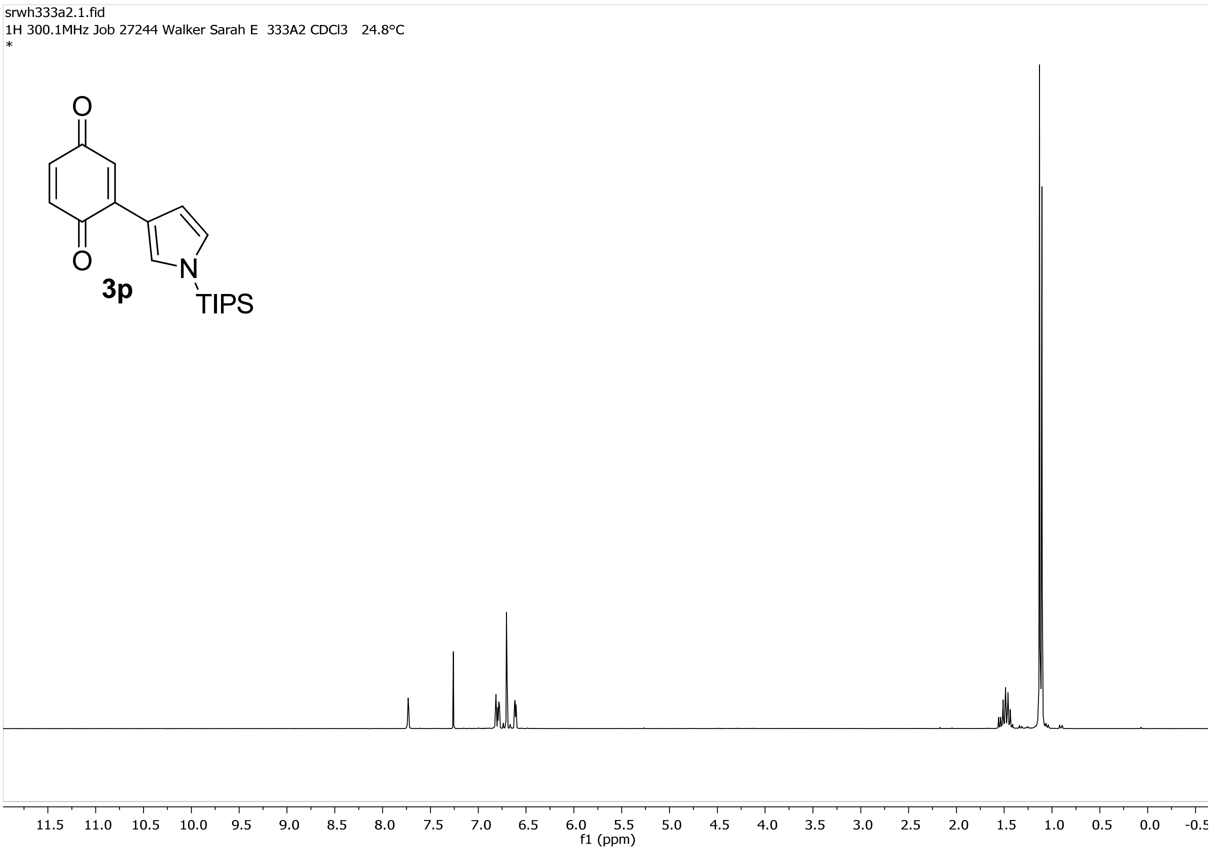
srwh333a2.1.fid
1H 300.1MHz Job 27244 Walker Sarah E 333A2 CDCl3 24.8°C
3p
TIPS
f1 (ppm)

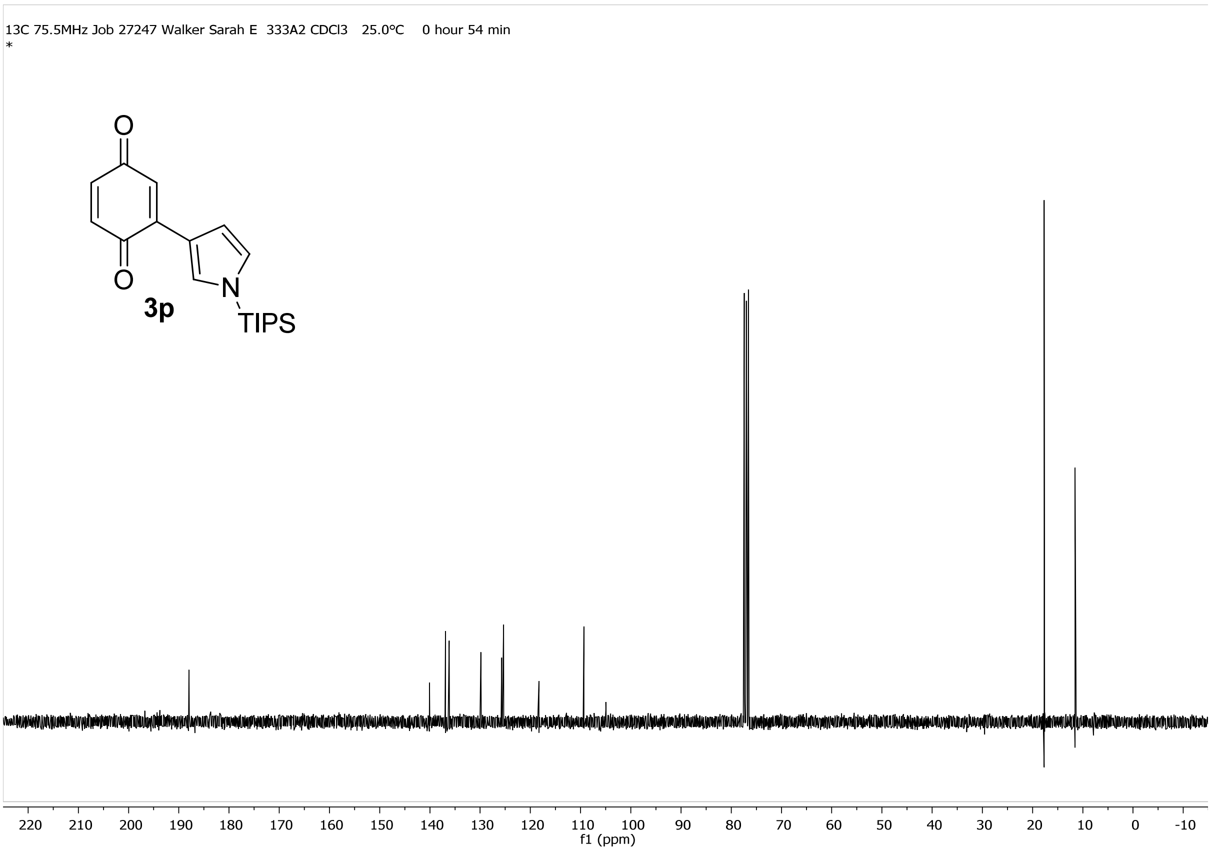
13C 75.5MHz Job 27247 Walker Sarah E 333A2 CDCl3 25.0°C 0 hour 54 min
3p
TIPS
f1 (ppm)

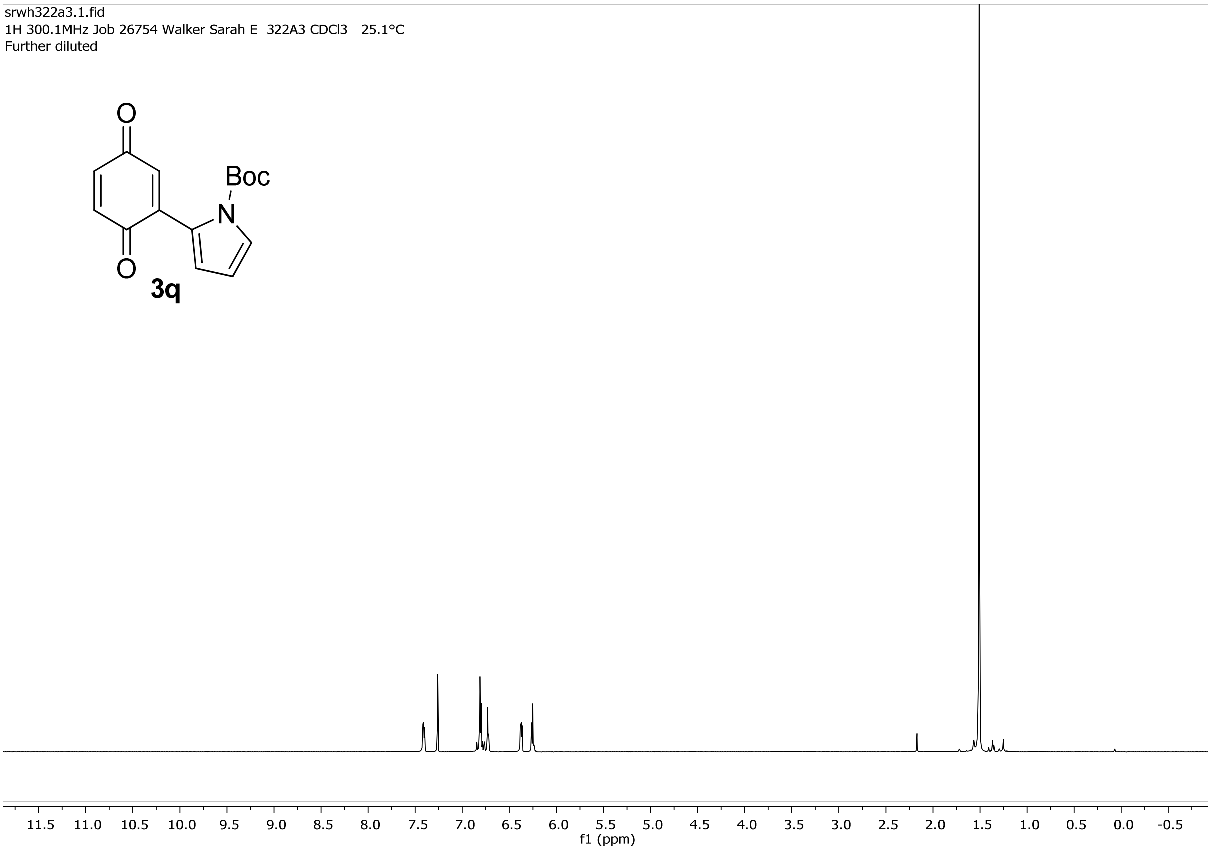
srwh322a3.1.fid
1H 300.1MHz Job 26754 Walker Sarah E 322A3 CDCl3 25.1°C
Further diluted
Boc
N
O
O
3q
11.5 11.0 10.5 10.0 9.5 9.0 8.5 8.0 7.5 7.0 6.5 6.0 5.5 5.0 4.5 4.0 3.5 3.0 2.5 2.0 1.5 1.0 0.5 0.0 -0.5
f1 (ppm)

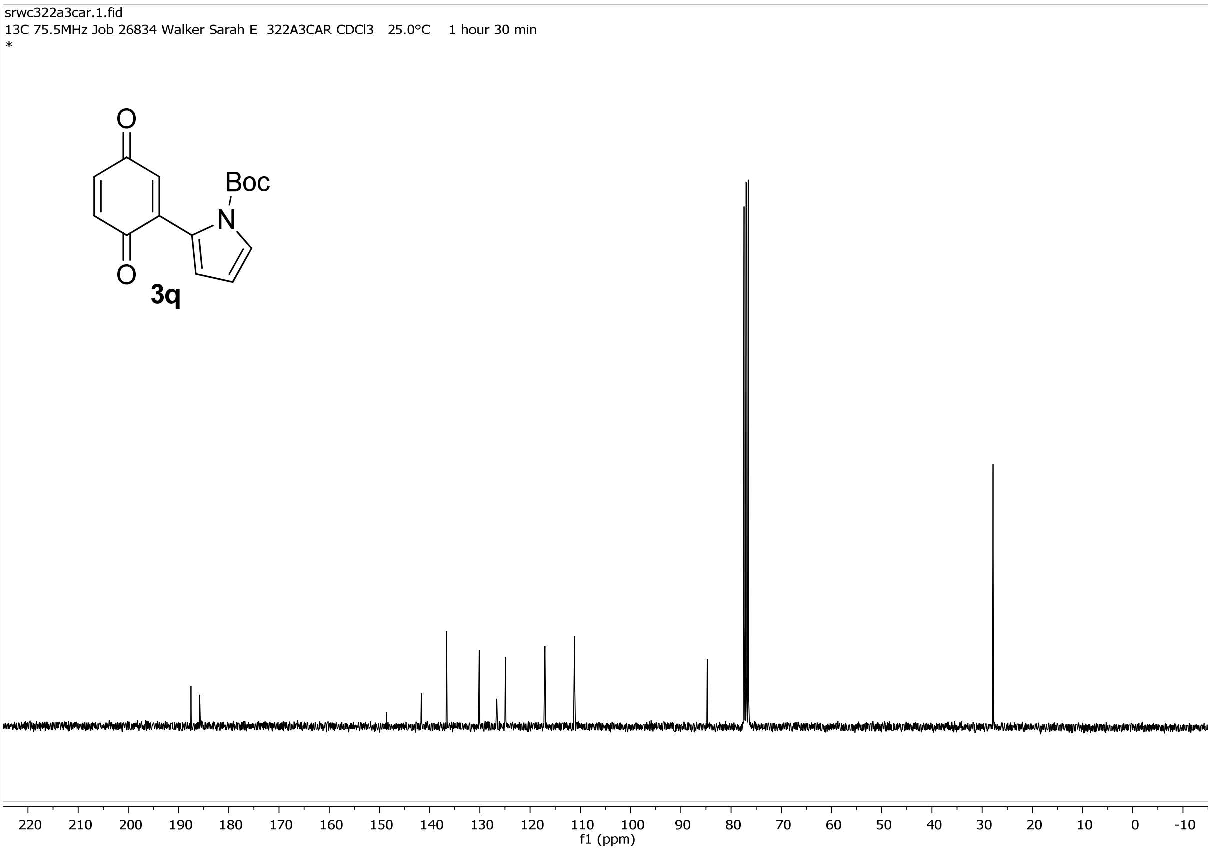
srwc322a3car.1.fid
13C 75.5MHz Job 26834 Walker Sarah E 322A3CAR CDCl3 25.0°C 1 hour 30 min
*
Boc
N
O
O
3q
220 210 200 190 180 170 160 150 140 130 120 110 100 90 80 70 60 50 40 30 20 10 0 -10
f1 (ppm)

srwhbq017c1H 300.1MHz Job 20665 Walker Sarah E BQ017C CDCl3   24.9°C
Mono cyclohexane after being on Buchi 1 hour - no DCM?

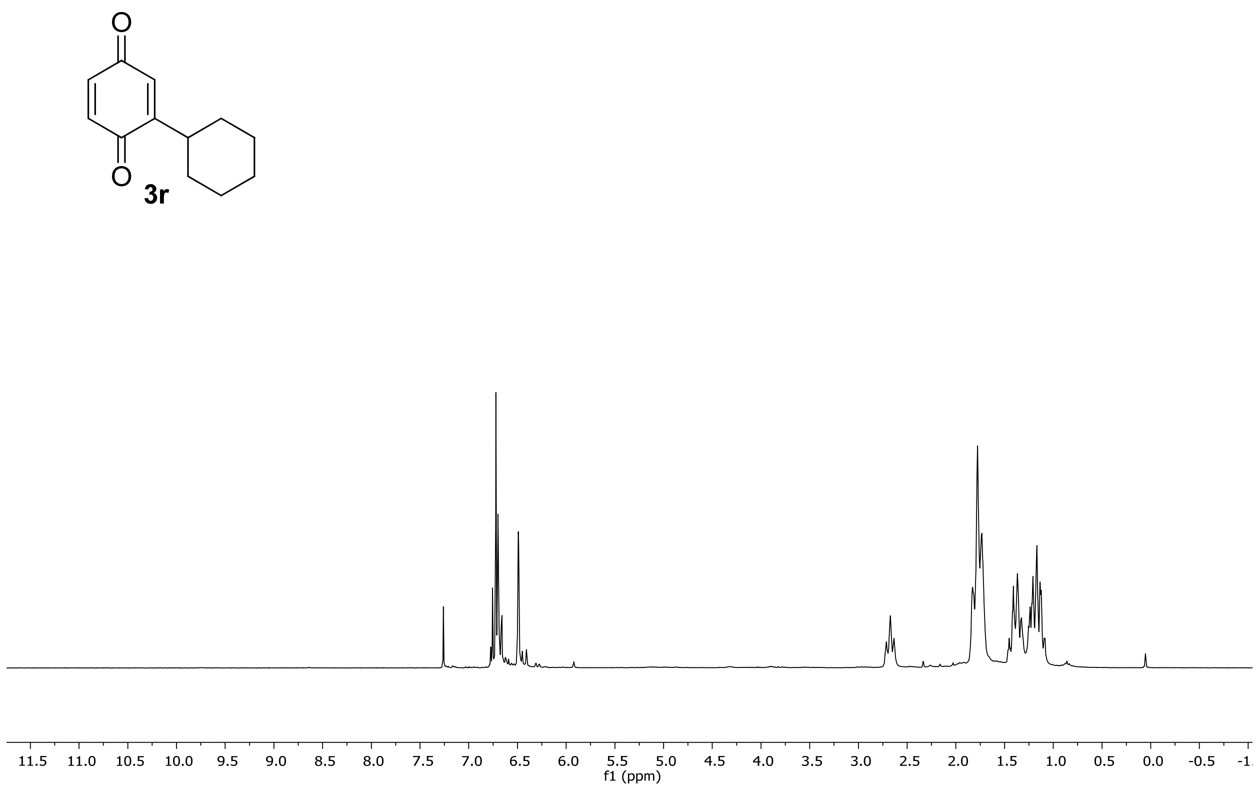

srwcbq017c.1.fid
13C 100.6MHz Job 19955 Walker Sarah E BQ017C CDCl3   25.0°C   0 hour 14 min
*

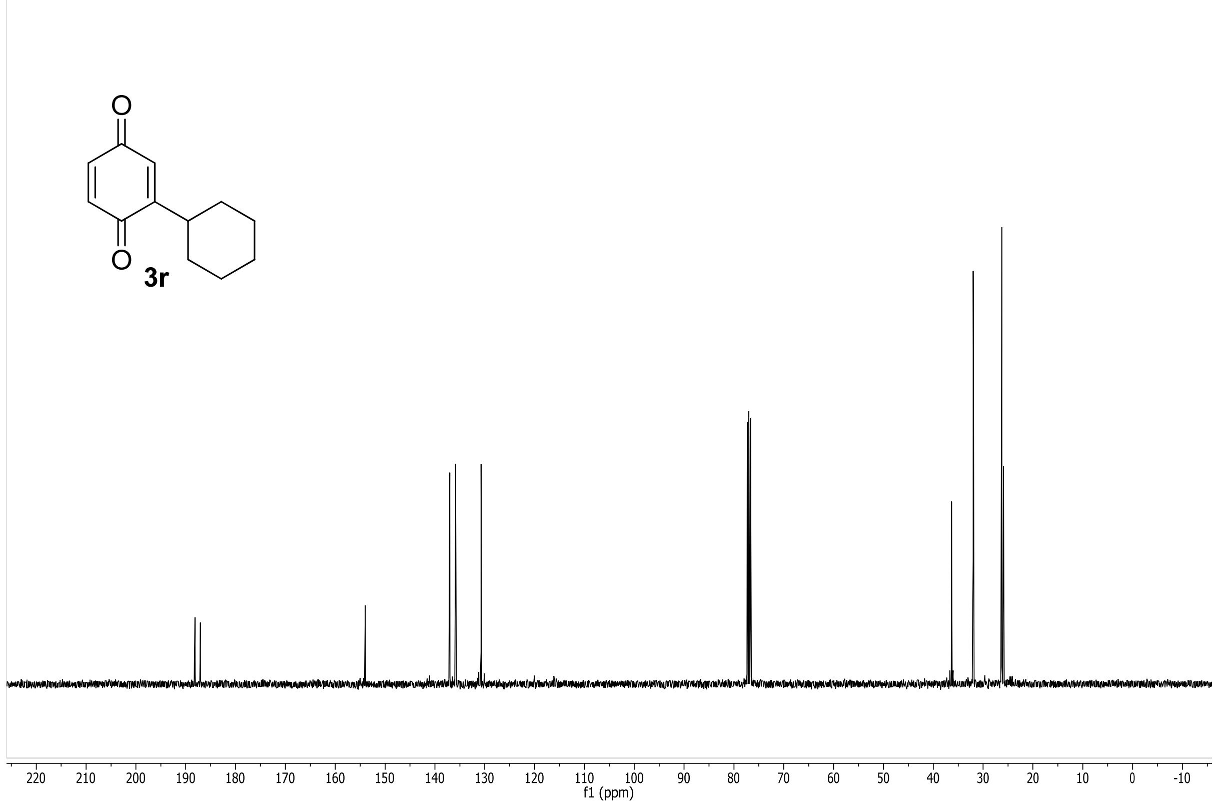

srwhbq018c1H 300.1MHz Job 20699 Walker Sarah E BQ018C CDCl3 25.0°C
*

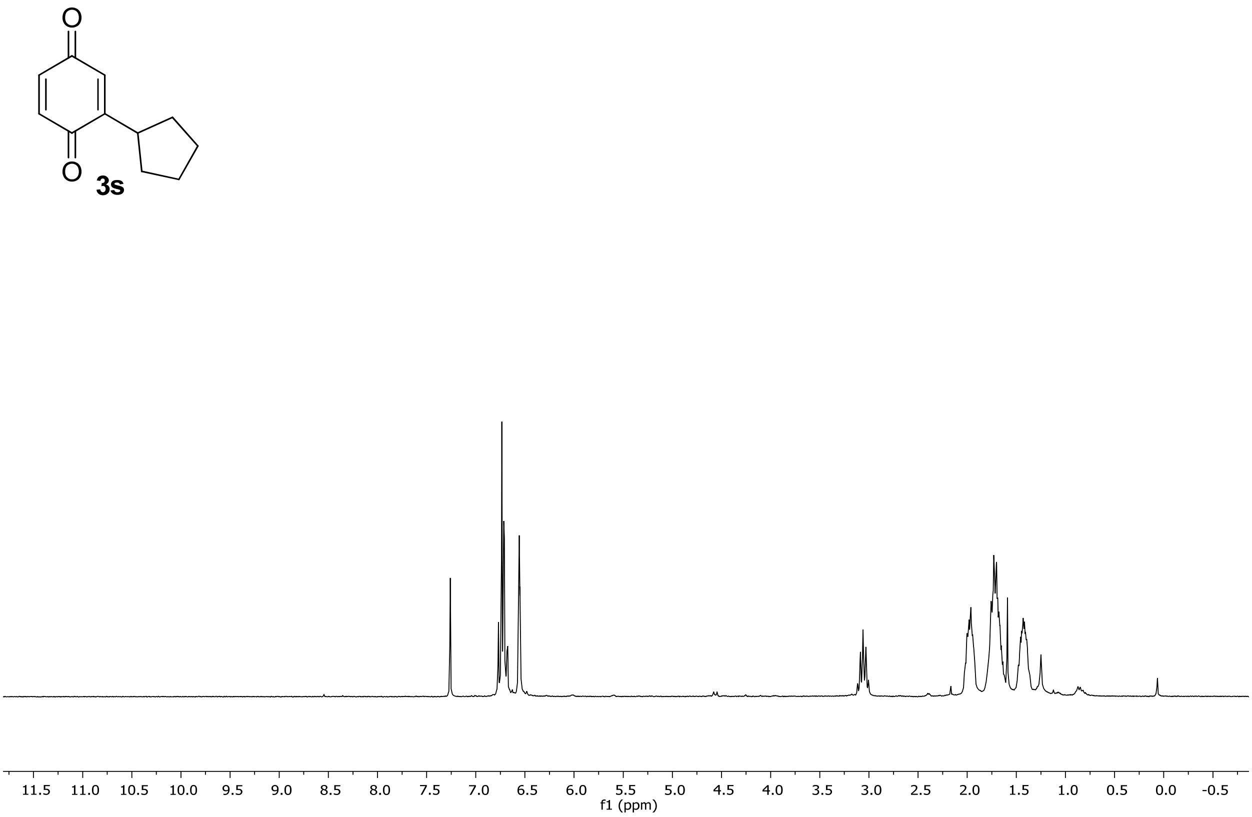

srwcbq018pur.1.fid
13C 75.5MHz Job 20766 Walker Sarah E BQ018PUR CDCl3 25.0°C 0 hour 18 min
Pure, no grease?

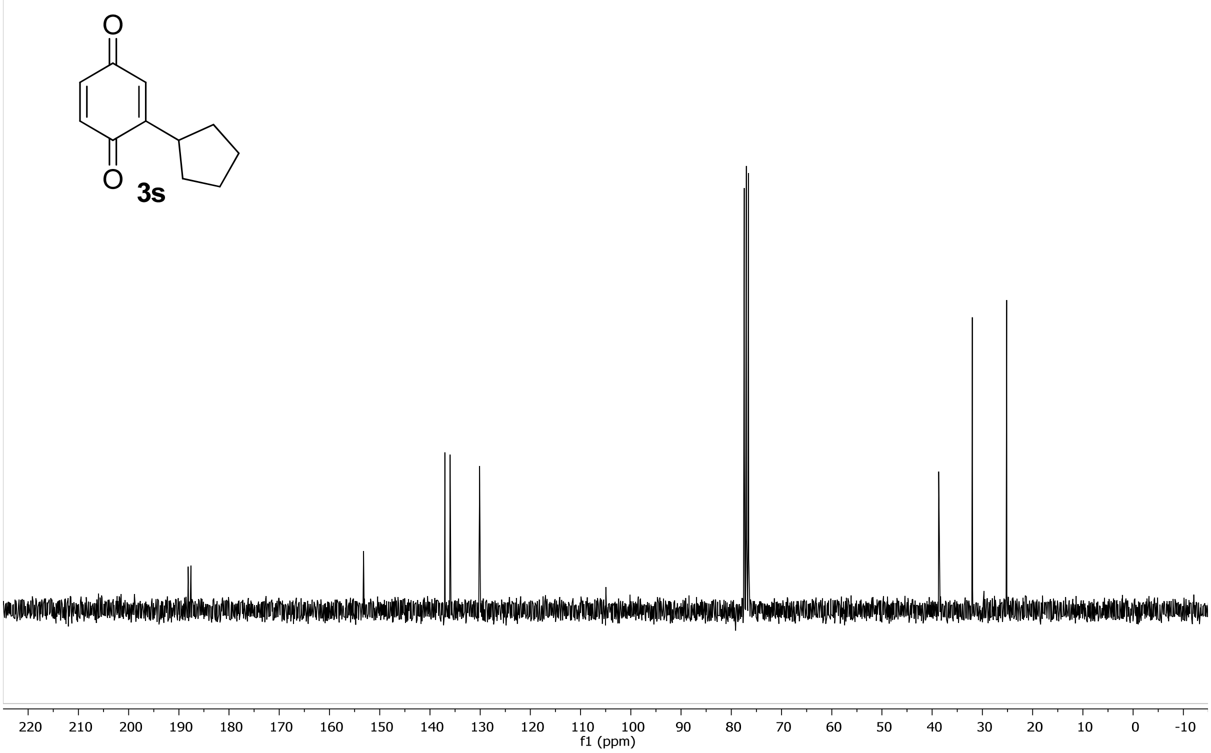

jmjh230512a1H 300.1MHz Job 16824 Jordan-Hore Jamie A 230512A CDCl3 21.2°C
tp

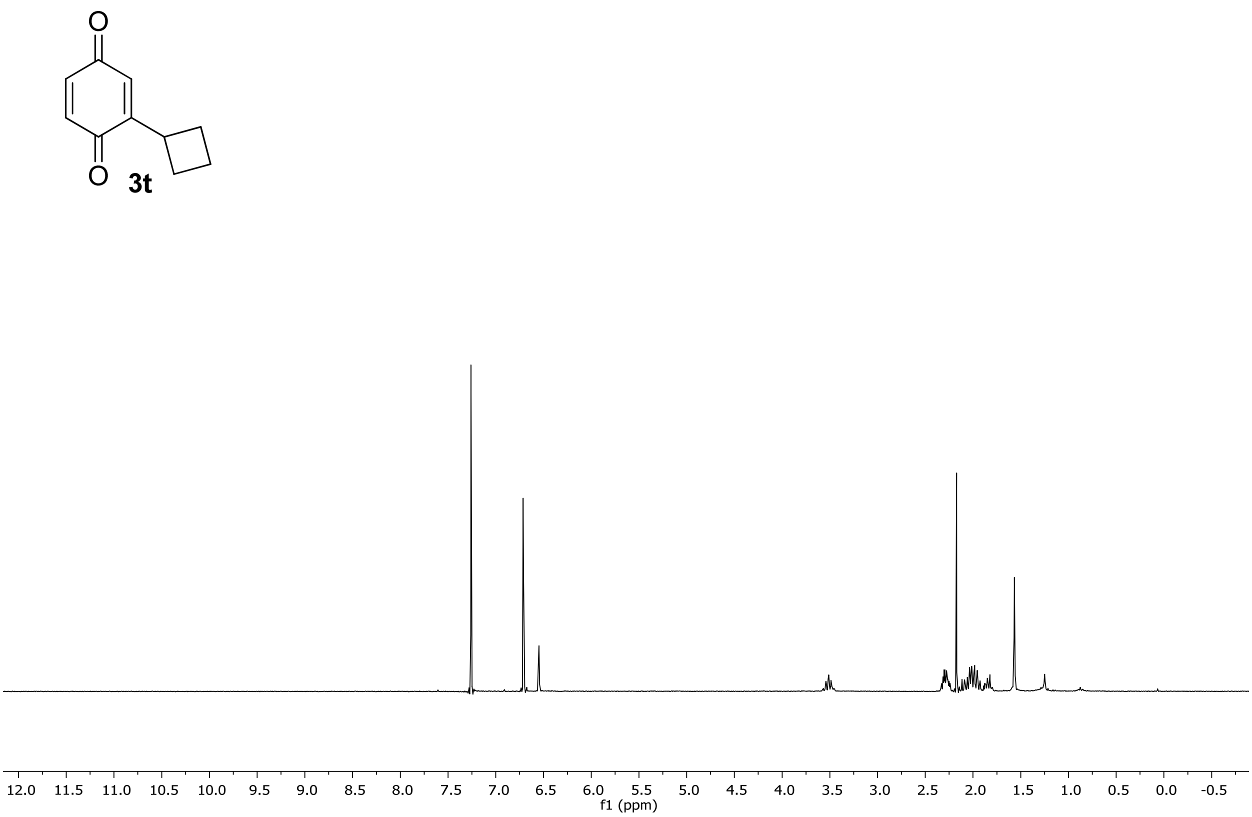

srwc304a.1.fid
13C 100.6MHz Job 20035 Walker Sarah E 304A CDCl3 25.0°C 17 hours 59 min
*

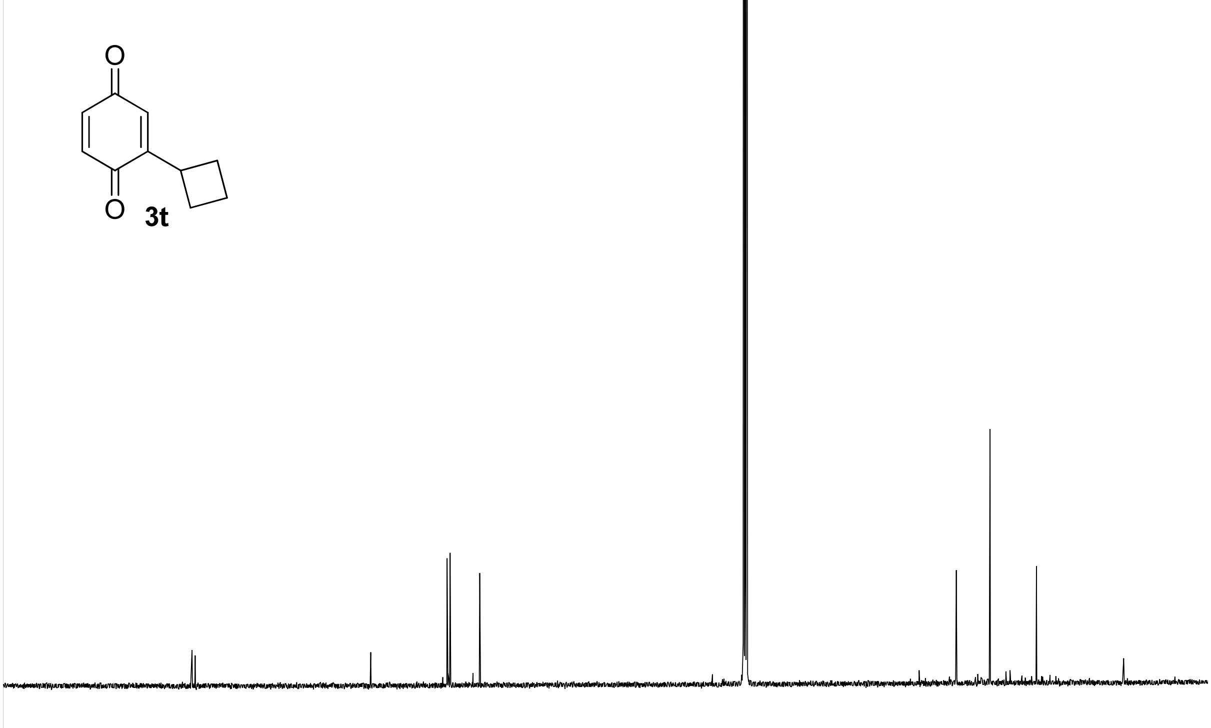

srwh343col2a.1.fid
1H 400.1MHz Job 20076 Walker Sarah E 343COL2A CDCl3 25.0°C
*

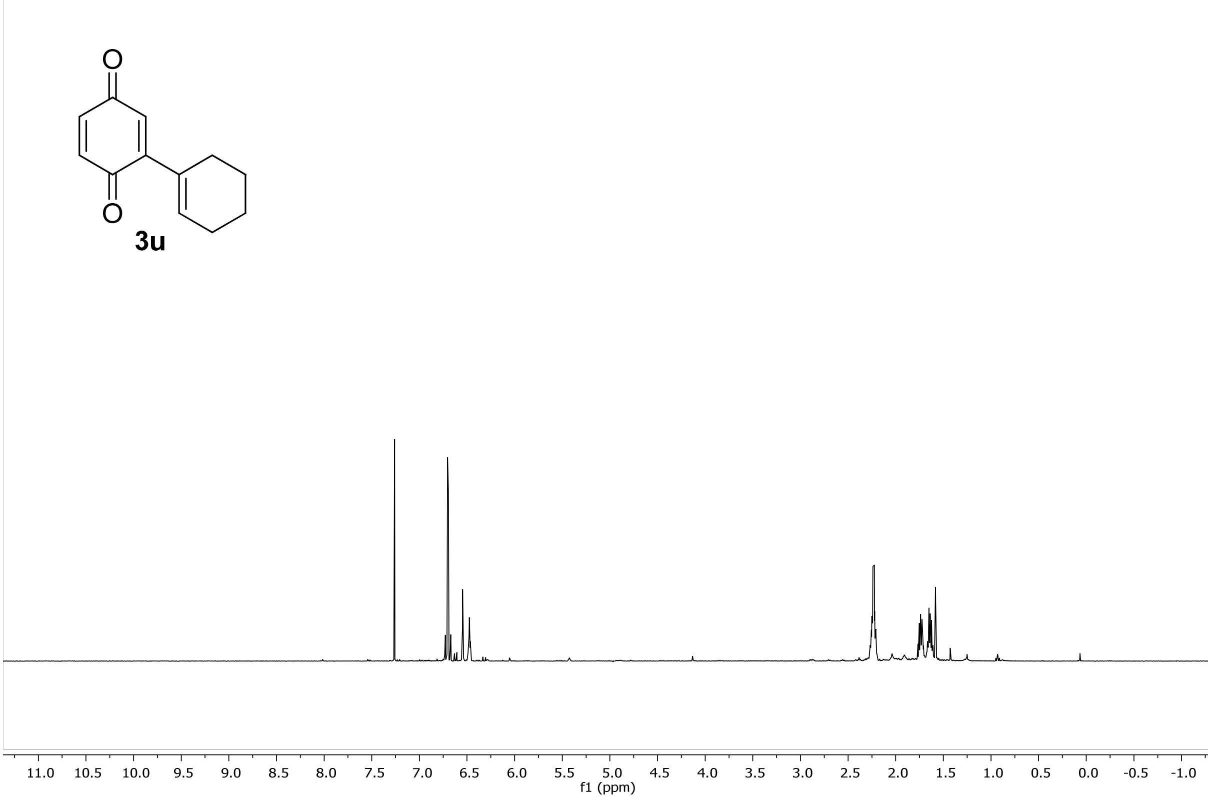

srwc343col2a.1.fid
13C 100.6MHz Job 20077 Walker Sarah E 343COL2A CDCl3 25.0°C 3 hours 53 min
*

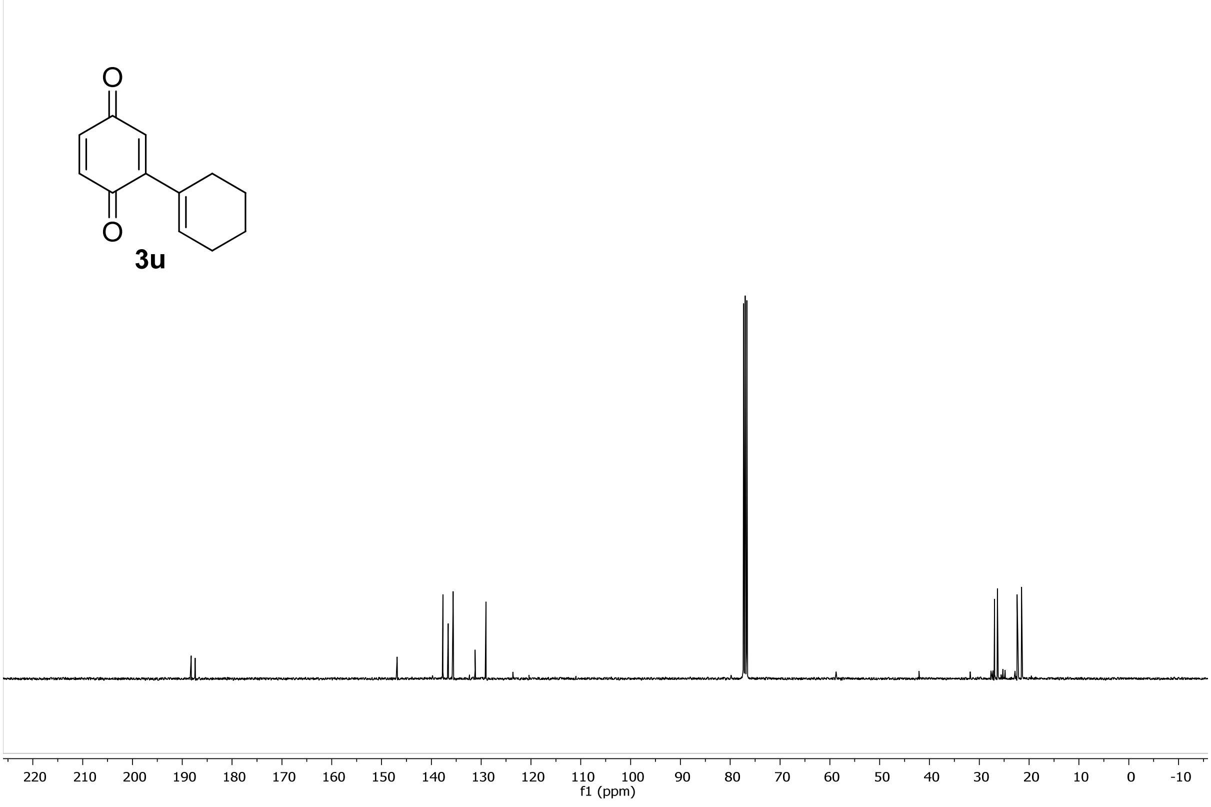

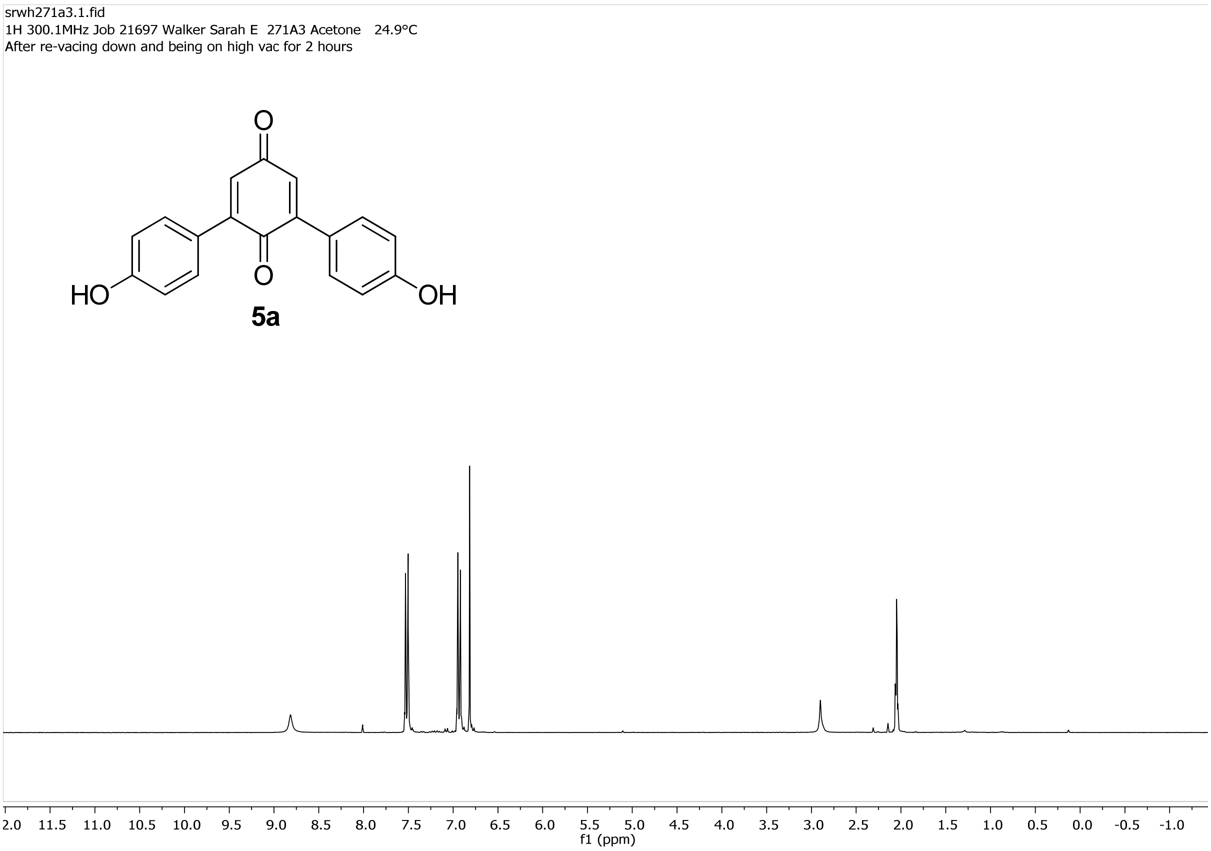
srwh271a3.1.fid
1H 300.1MHz Job 21697 Walker Sarah E 271A3 Acetone 24.9°C
After re-vacing down and being on high vac for 2 hours
5a
f1 (ppm)

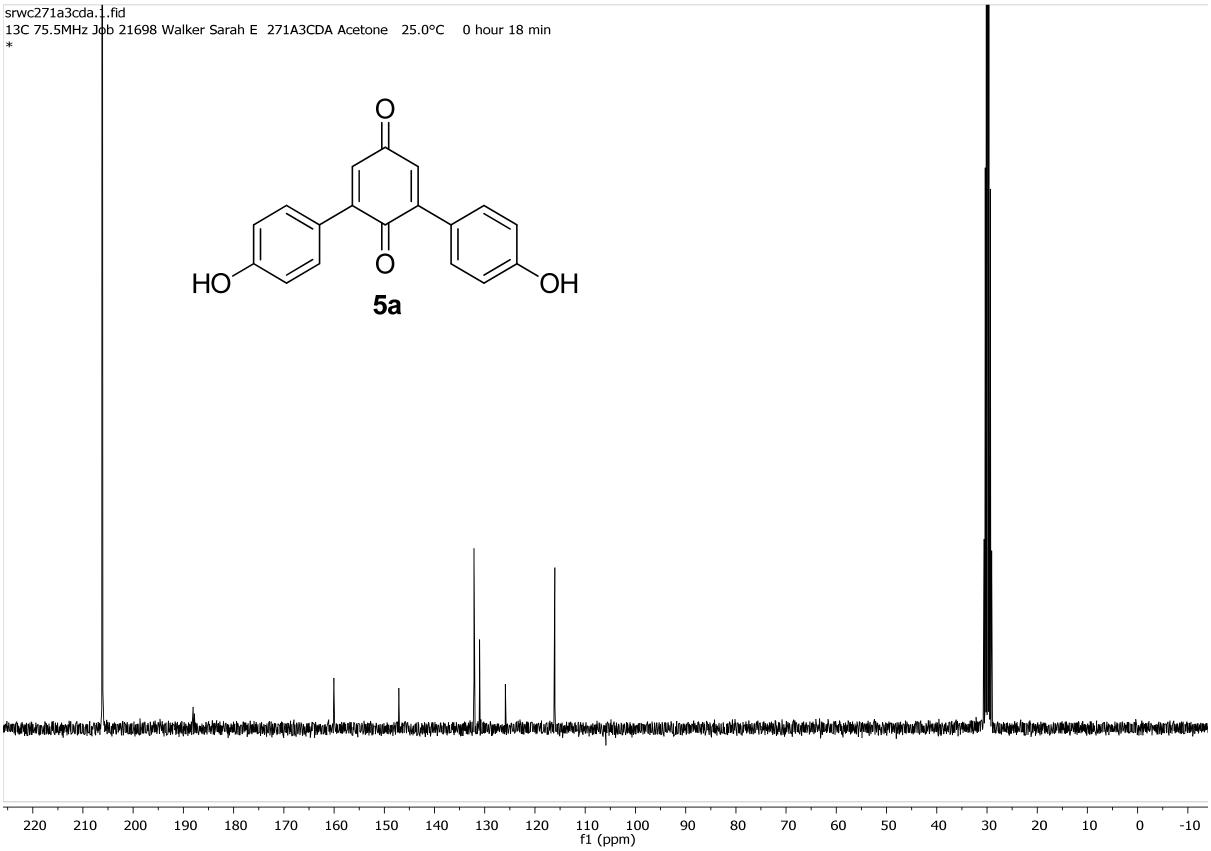
srwc271a3cda.1.fid
13C 75.5MHz Job 21698 Walker Sarah E 271A3CDA Acetone 25.0°C 0 hour 18 min
*
5a
f1 (ppm)

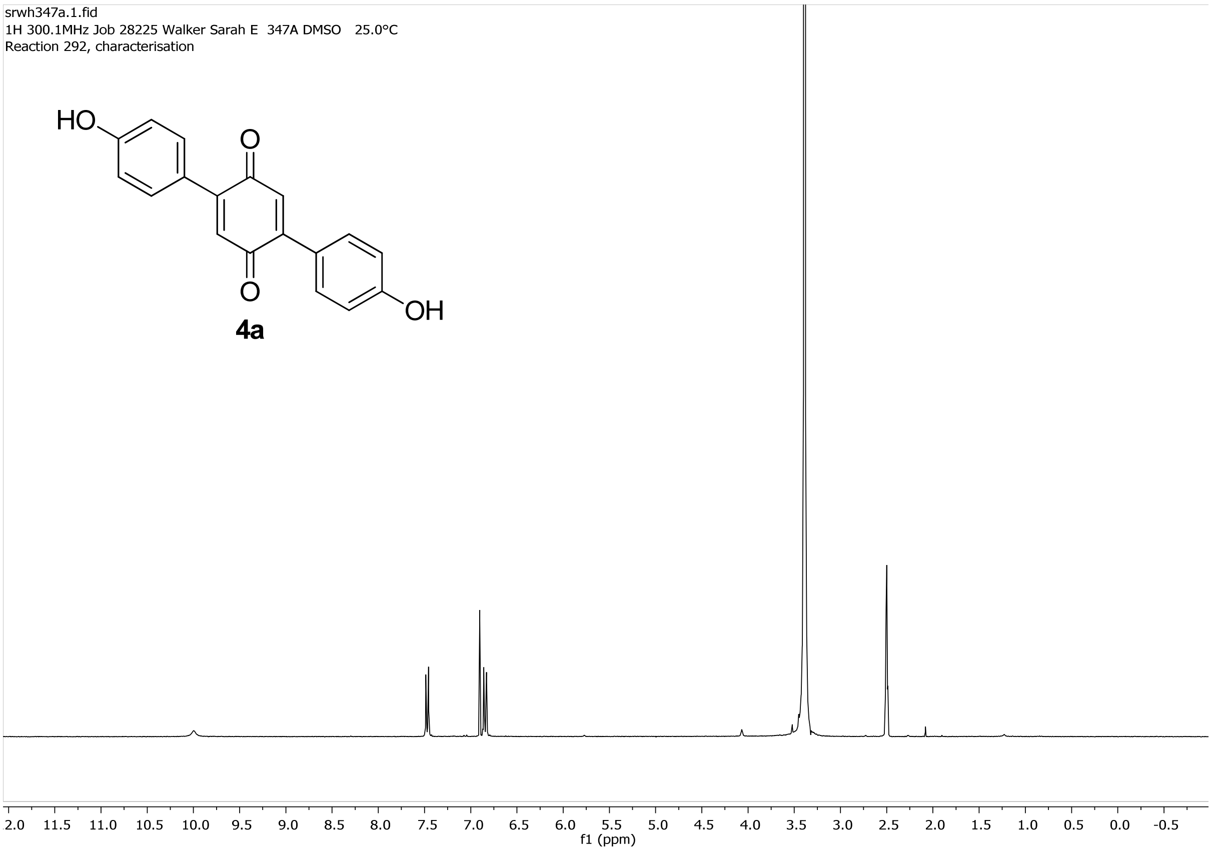
srwh347a.1.fid
1H 300.1MHz Job 28225 Walker Sarah E 347A DMSO 25.0°C
Reaction 292, characterisation
HO
O
O
OH
4a
2.0 11.5 11.0 10.5 10.0 9.5 9.0 8.5 8.0 7.5 7.0 6.5 6.0 5.5 5.0 4.5 4.0 3.5 3.0 2.5 2.0 1.5 1.0 0.5 0.0 -0.5
f1 (ppm)

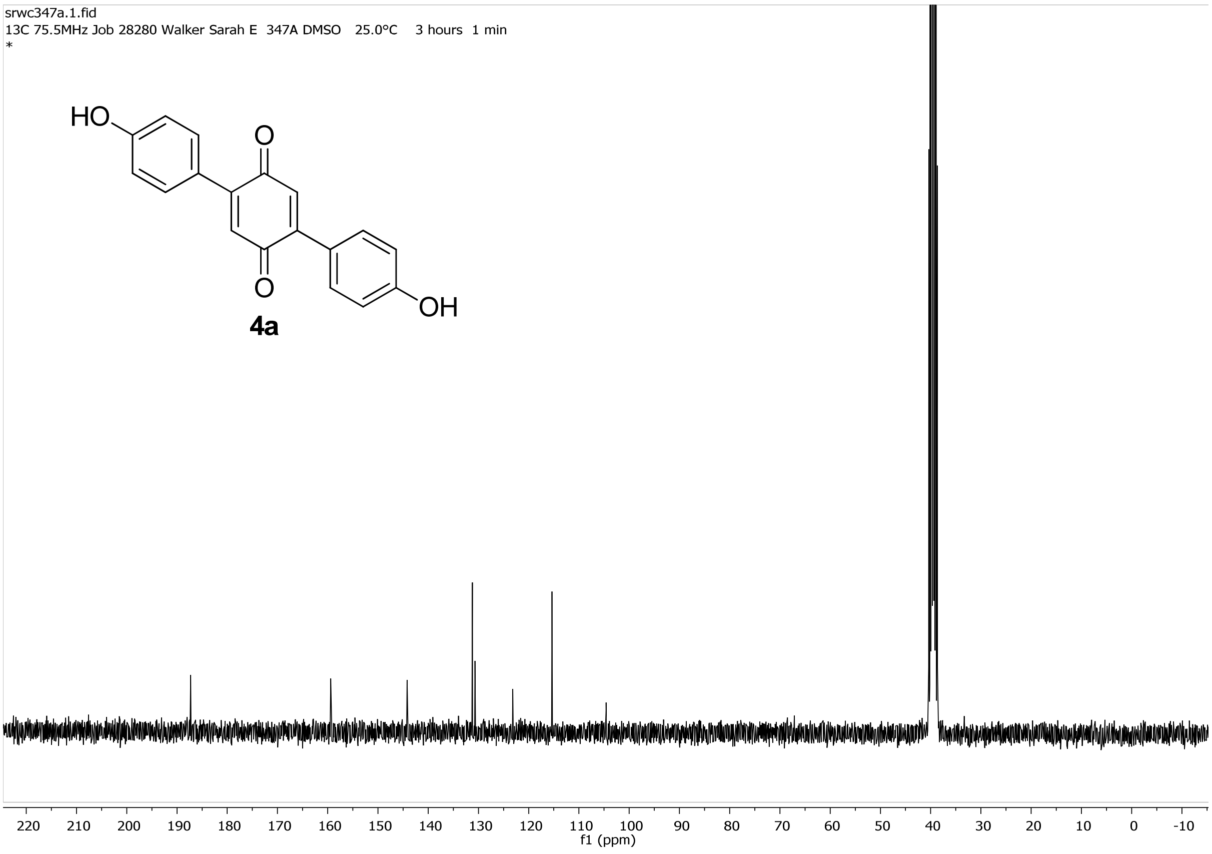
srwc347a.1.fid
13C 75.5MHz Job 28280 Walker Sarah E 347A DMSO 25.0°C 3 hours 1 min
*
HO
O
O
OH
4a
220 210 200 190 180 170 160 150 140 130 120 110 100 90 80 70 60 50 40 30 20 10 0 -10
f1 (ppm)

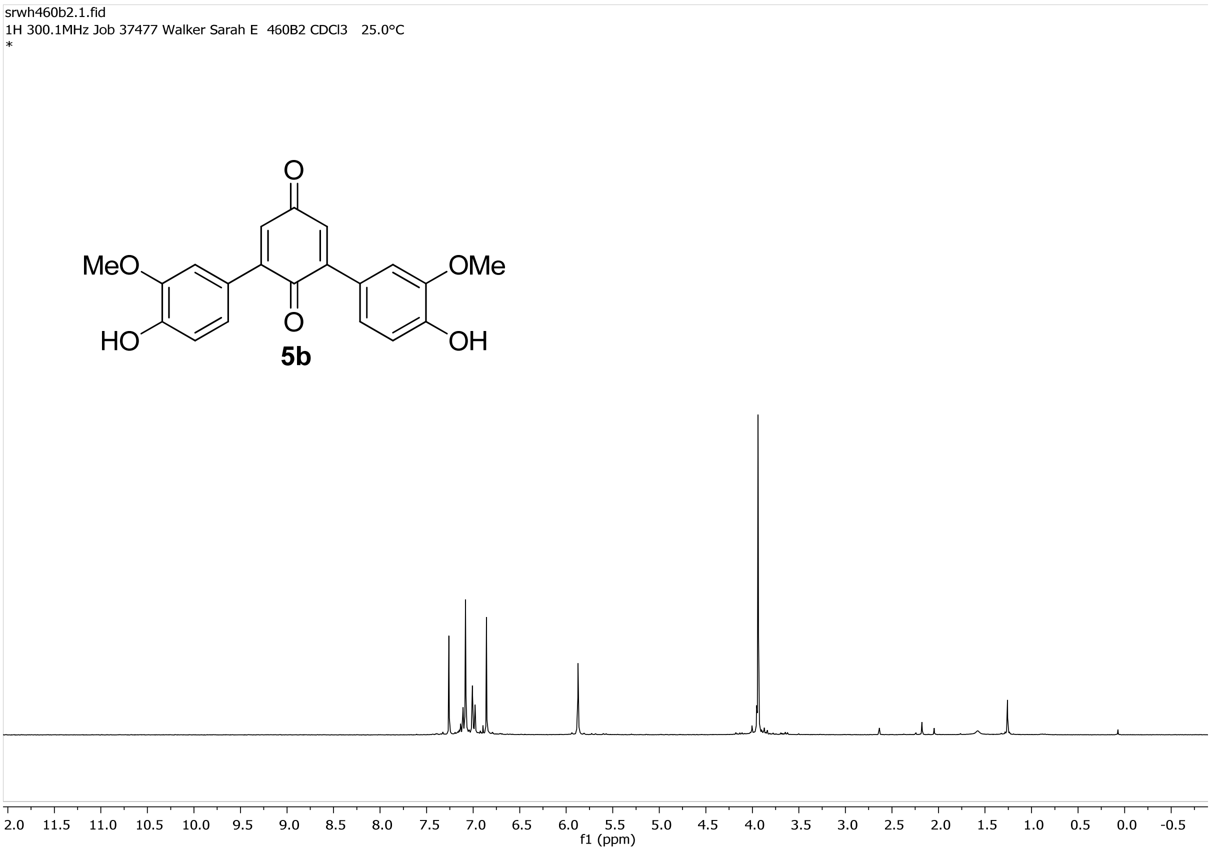
srwh460b2.1.fid
1H 300.1MHz Job 37477 Walker Sarah E 460B2 CDCl3 25.0°C
*
MeO
OMe
HO
OH
O
O
5b
2.0 11.5 11.0 10.5 10.0 9.5 9.0 8.5 8.0 7.5 7.0 6.5 6.0 5.5 5.0 4.5 4.0 3.5 3.0 2.5 2.0 1.5 1.0 0.5 0.0 -0.5
f1 (ppm)

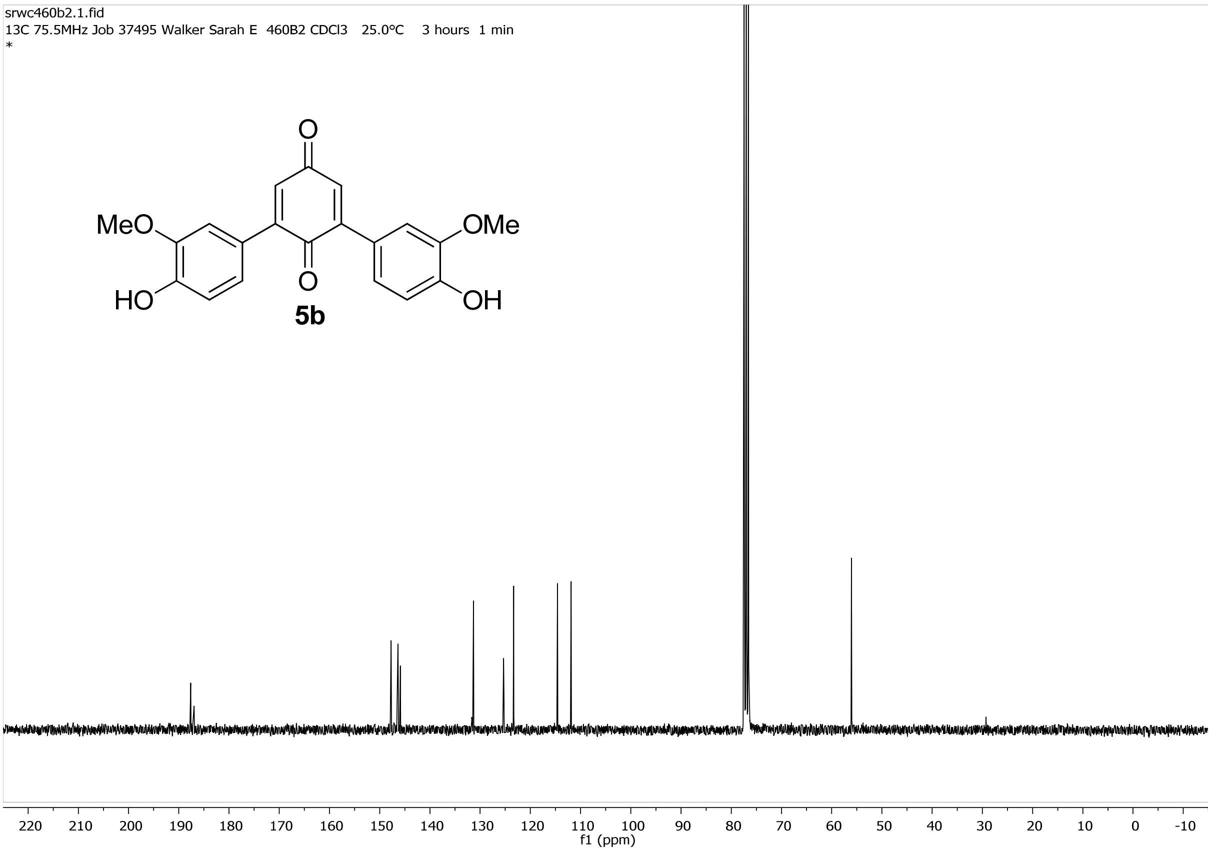
srwc460b2.1.fid
13C 75.5MHz Job 37495 Walker Sarah E 460B2 CDCl3 25.0°C 3 hours 1 min
*
MeO
OMe
HO
OH
O
O
5b
220 210 200 190 180 170 160 150 140 130 120 110 100 90 80 70 60 50 40 30 20 10 0 -10
f1 (ppm)

srwh294d2.1.fid
1H 300.1MHz Job 23925 Walker Sarah E 294D2 CDCl3 24.9°C
*

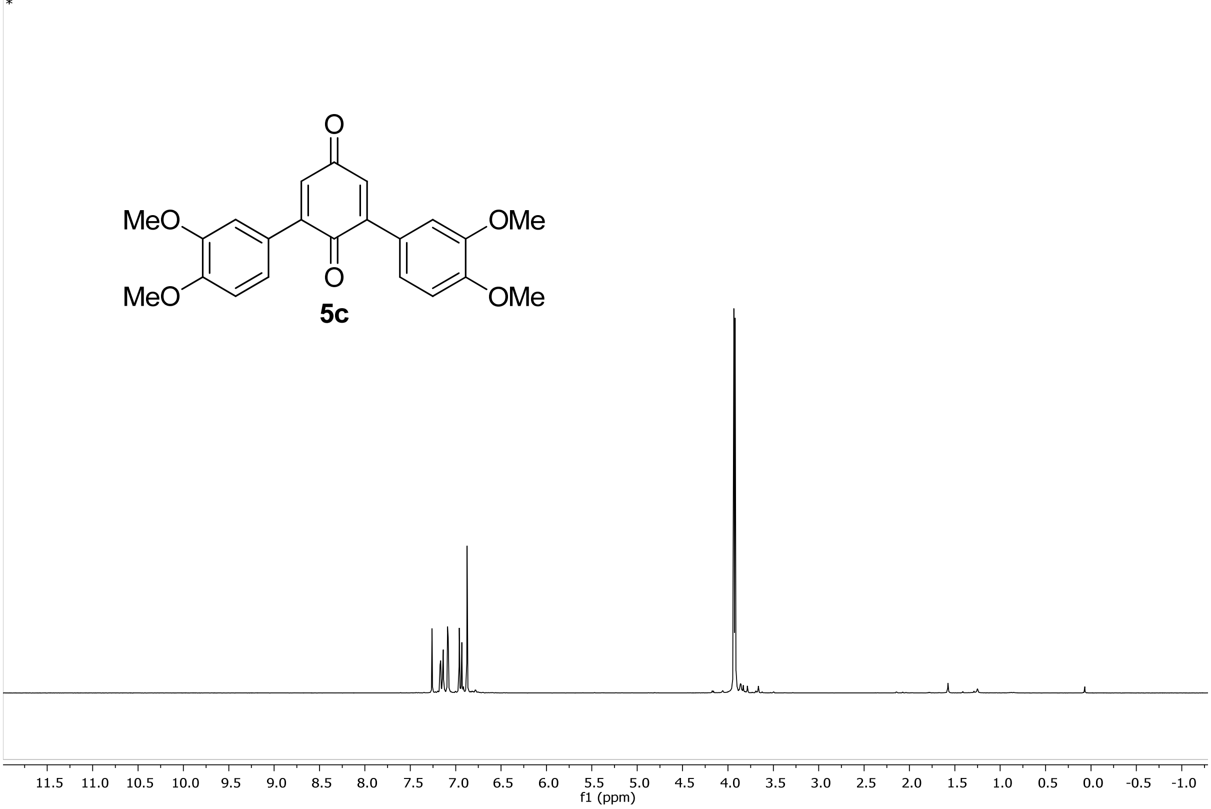

srwc292b.1.fid
13C 75.5MHz Job 23859 Walker Sarah E 292B CDCl3 25.0°C 2 hours 43 min
*

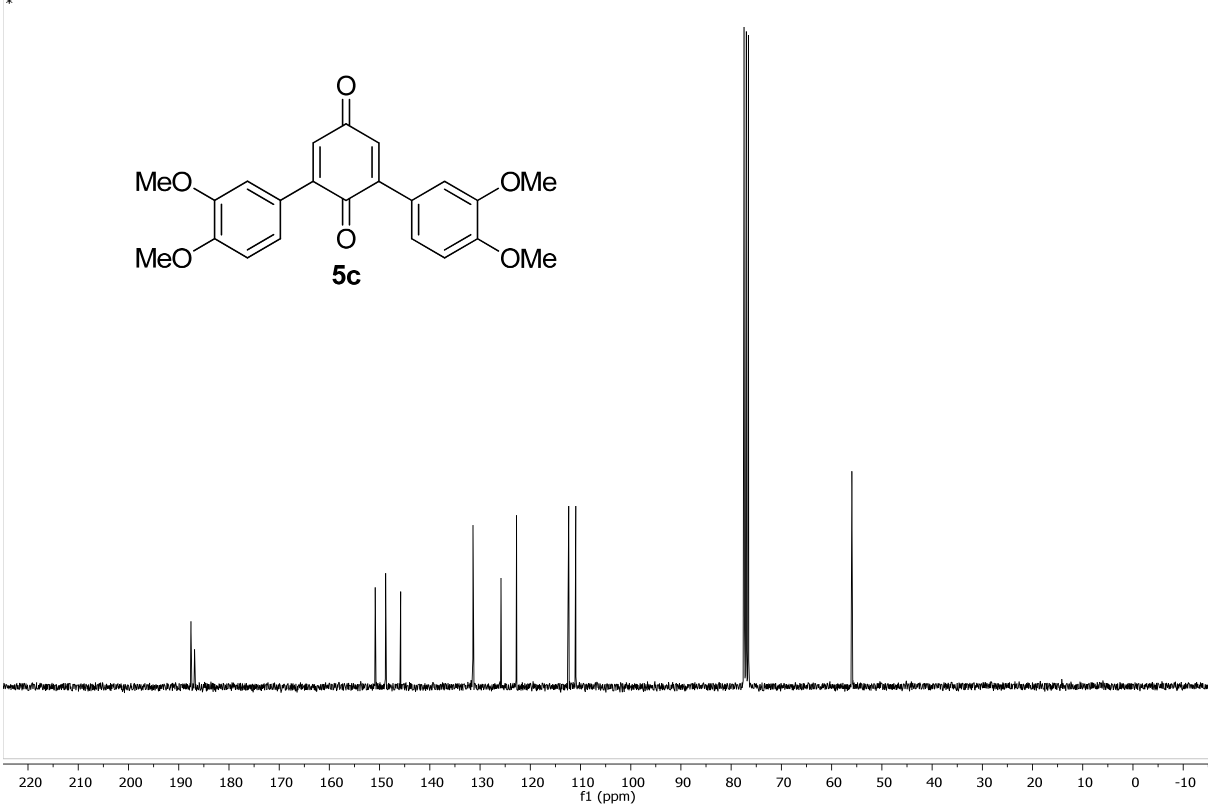

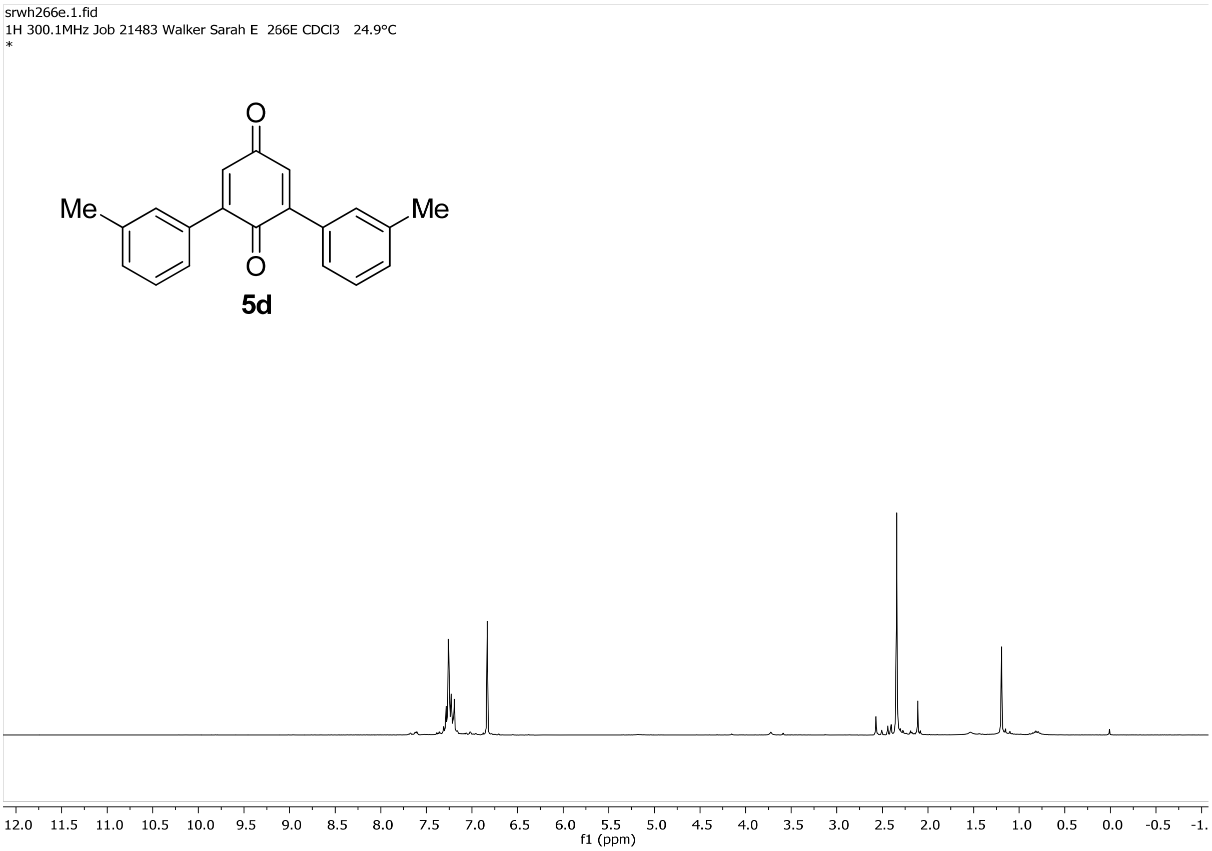
srwh266e.1.fid
1H 300.1MHz Job 21483 Walker Sarah E 266E CDCl3 24.9°C
*
Me
Me
O
O
5d
12.0 11.5 11.0 10.5 10.0 9.5 9.0 8.5 8.0 7.5 7.0 6.5 6.0 5.5 5.0 4.5 4.0 3.5 3.0 2.5 2.0 1.5 1.0 0.5 0.0 -0.5 -1.
f1 (ppm)

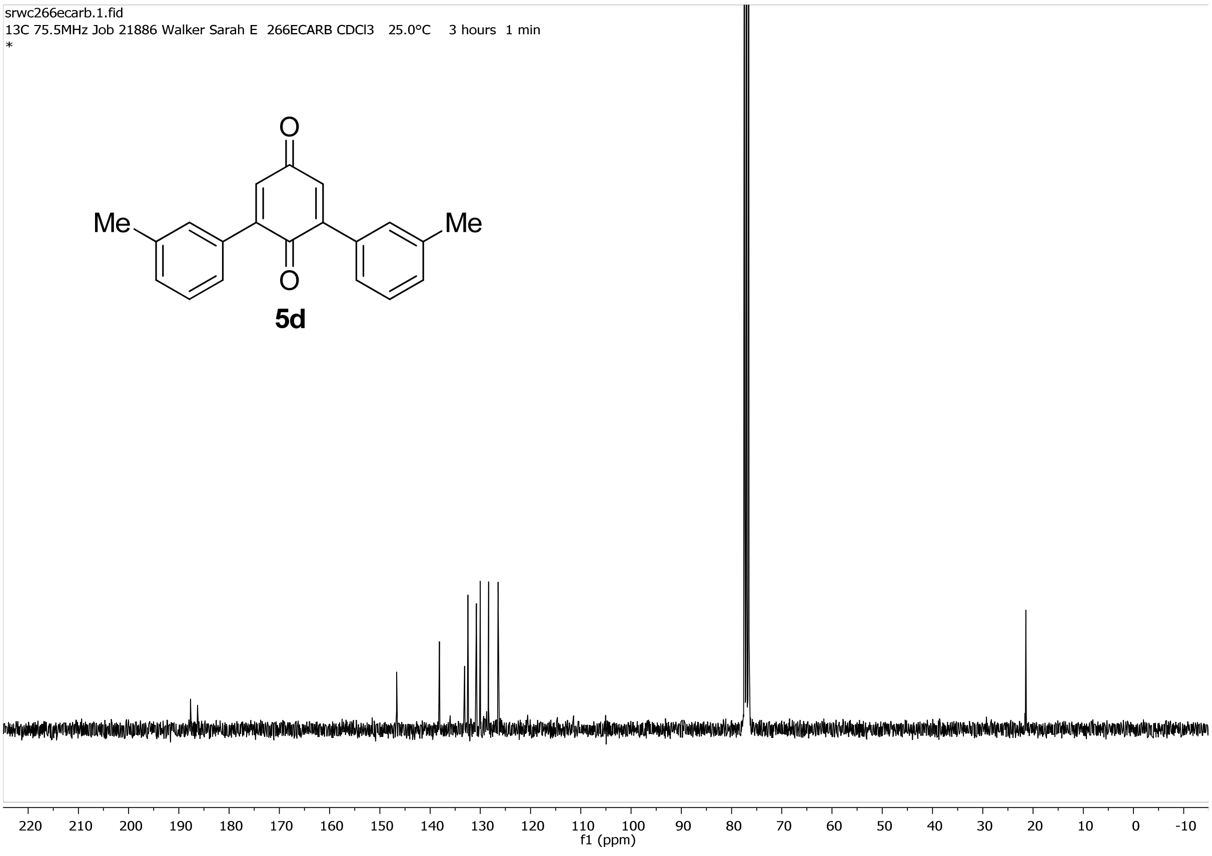
srwc266ecarb.1.fid
13C 75.5MHz Job 21886 Walker Sarah E 266ECARB CDCl3 25.0°C 3 hours 1 min
*
Me
Me
O
O
5d
220 210 200 190 180 170 160 150 140 130 120 110 100 90 80 70 60 50 40 30 20 10 0 -10
f1 (ppm)

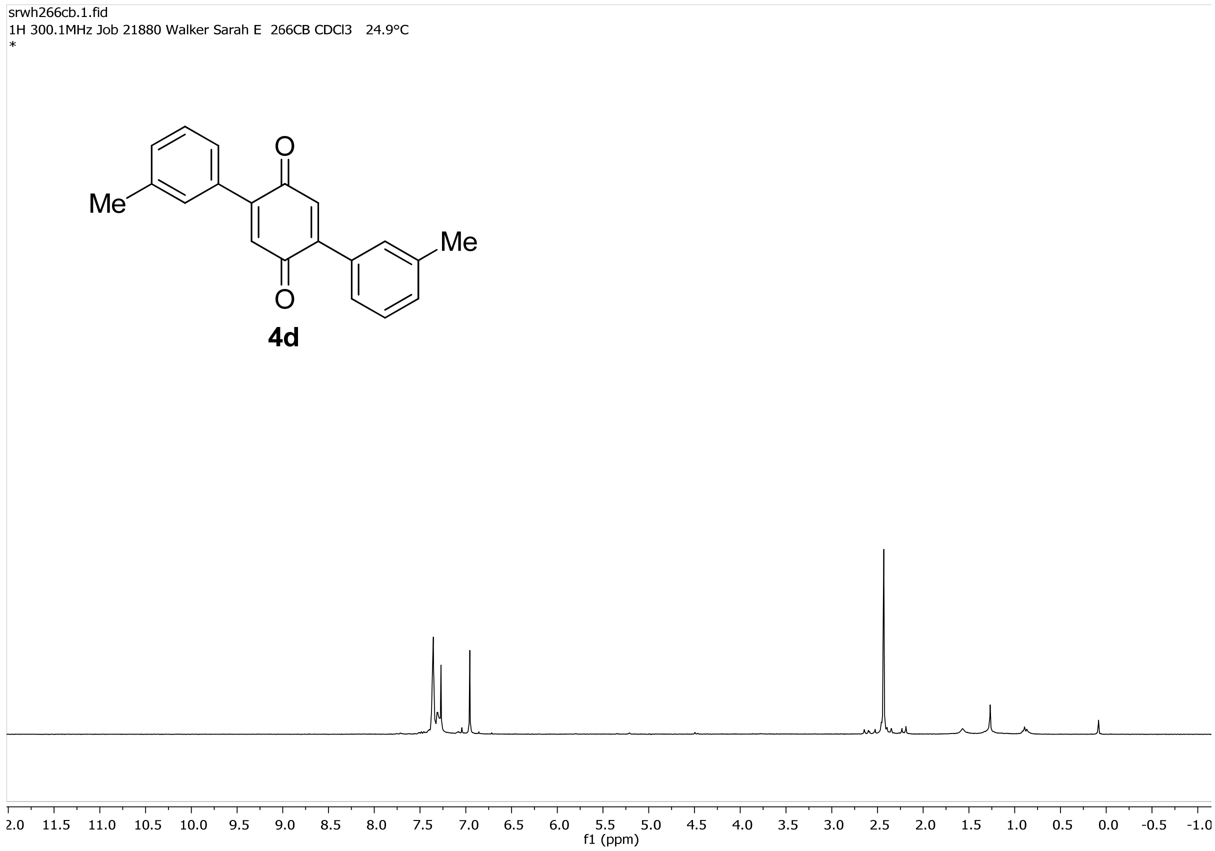
srwh266cb.1.fid
1H 300.1MHz Job 21880 Walker Sarah E 266CB CDCl3 24.9°C
*
O
Me
Me
O
4d
f1 (ppm)

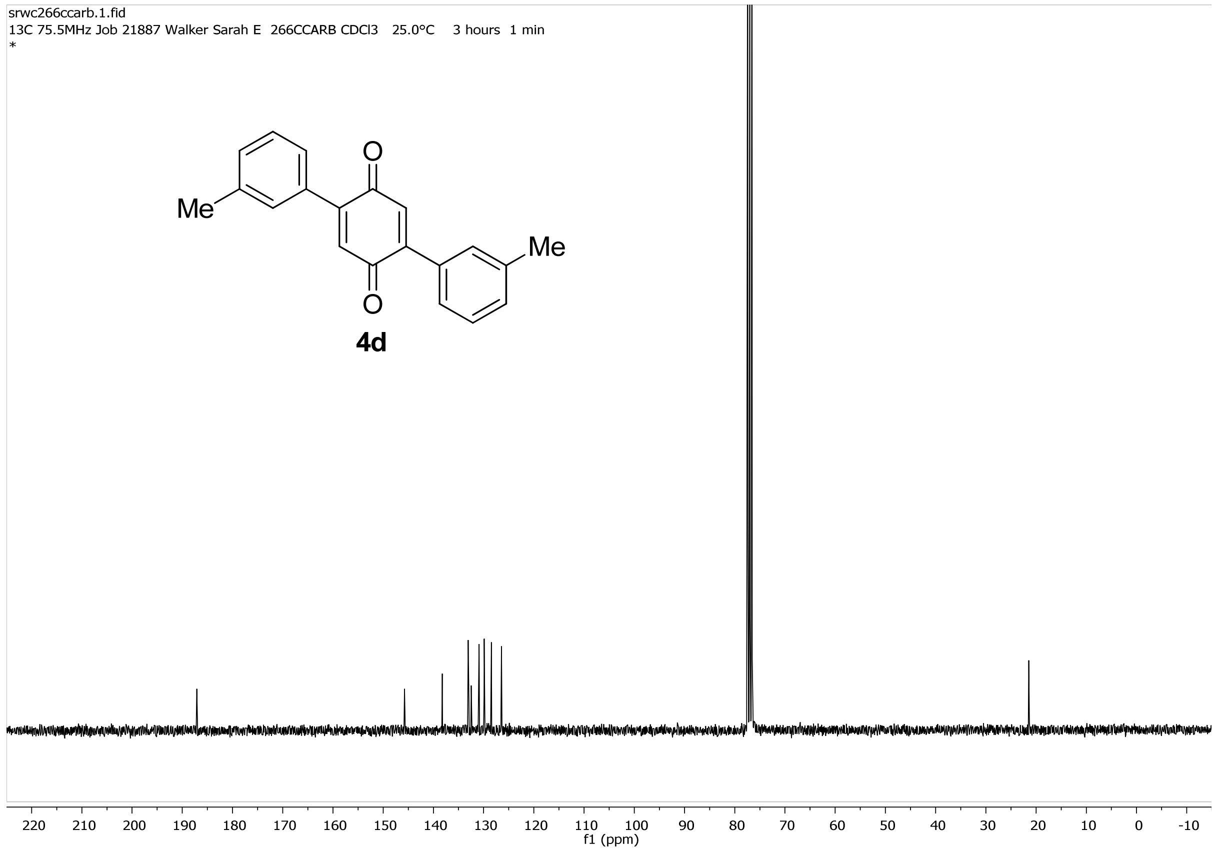
srwc266ccarb.1.fid
13C 75.5MHz Job 21887 Walker Sarah E 266CCARB CDCl3 25.0°C 3 hours 1 min
*
O
Me
Me
O
4d
f1 (ppm)

srwh286bc.1.fid
1H 300.1MHz Job 22941 Walker Sarah E 286BC CDCl3 24.9°C
B and C combined for mass and carbon

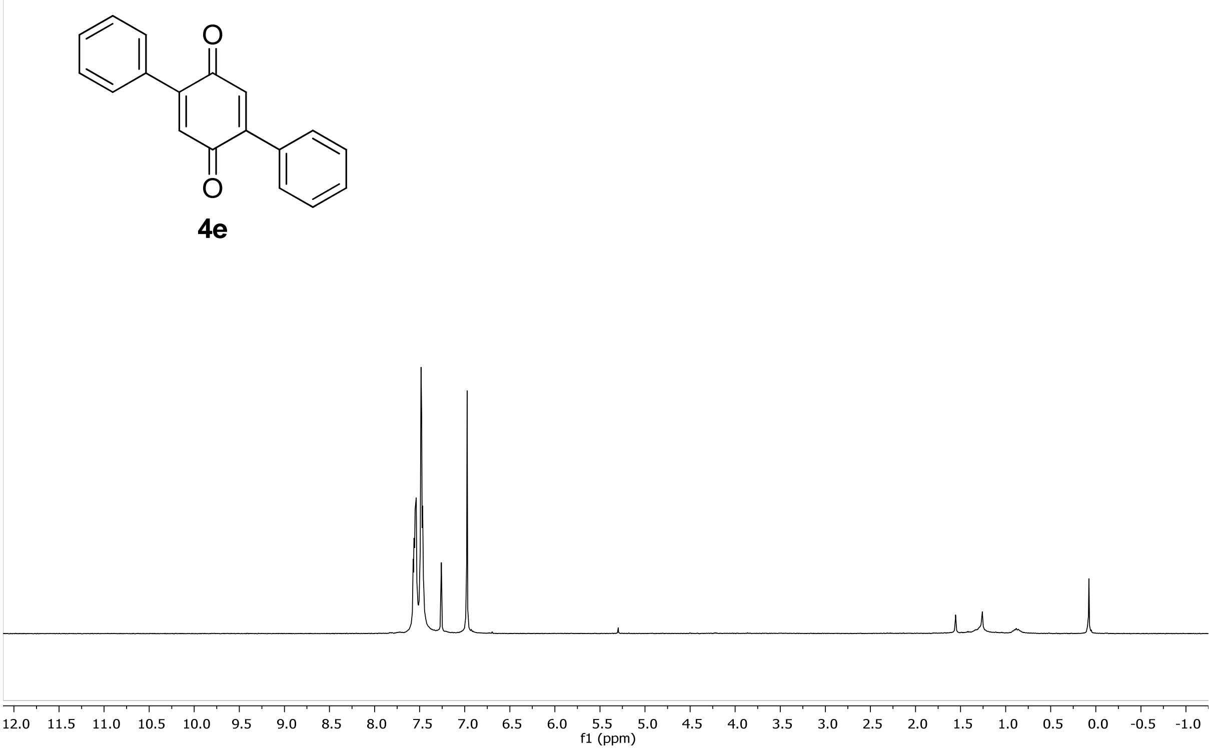

srwc286bc.1.fid
13C 75.5MHz Job 22960 Walker Sarah E 286BC CDCl3 24.9°C 3 hours 1 min
*

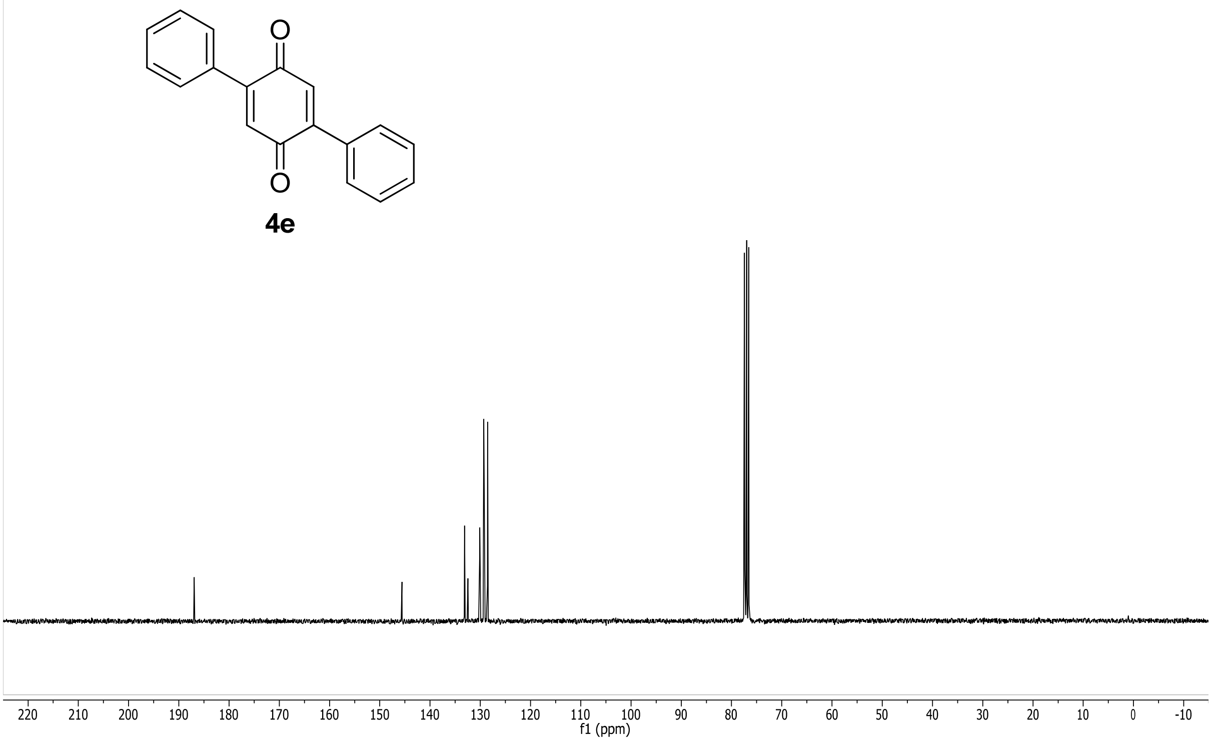

srwh286e.1.fid
1H 300.1MHz Job 22938 Walker Sarah E 286E CDCl3 24.9°C
*

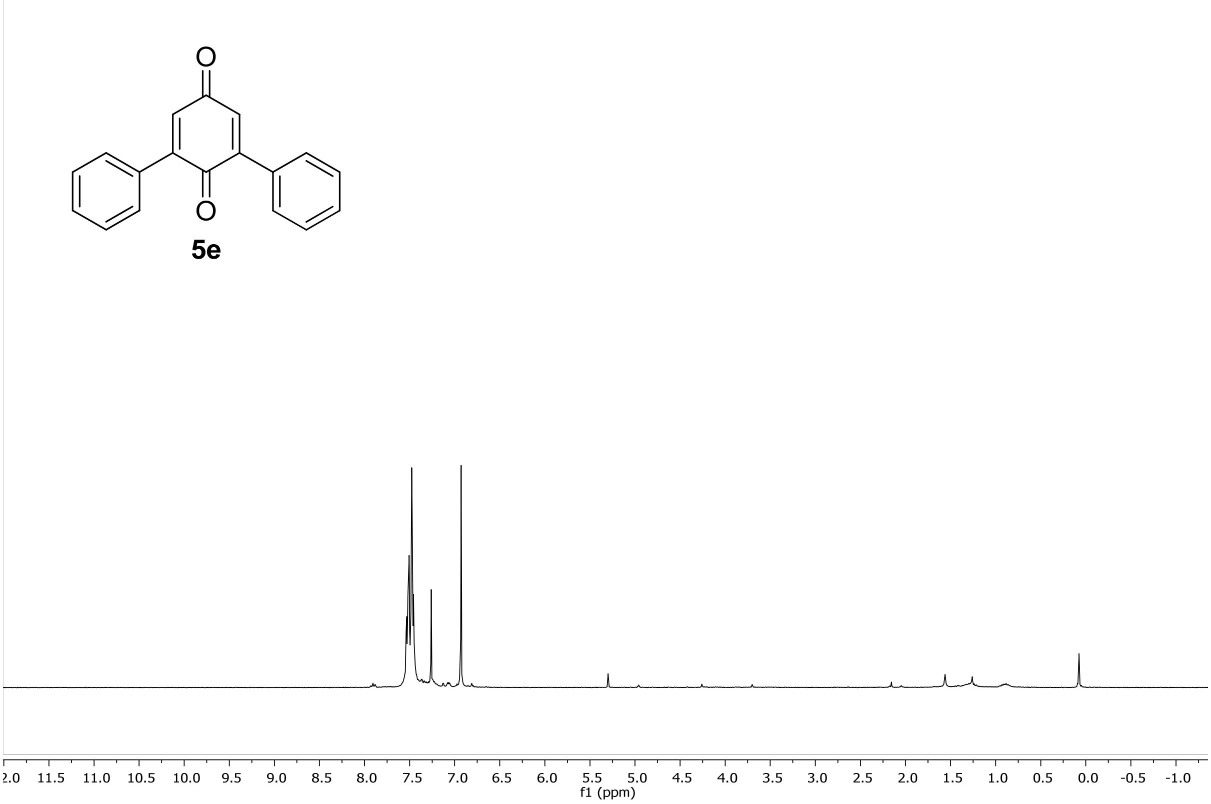

srwc286e.1.fid
13C 75.5MHz Job 22962 Walker Sarah E 286E CDCl3 24.9°C 3 hours 1 min
*

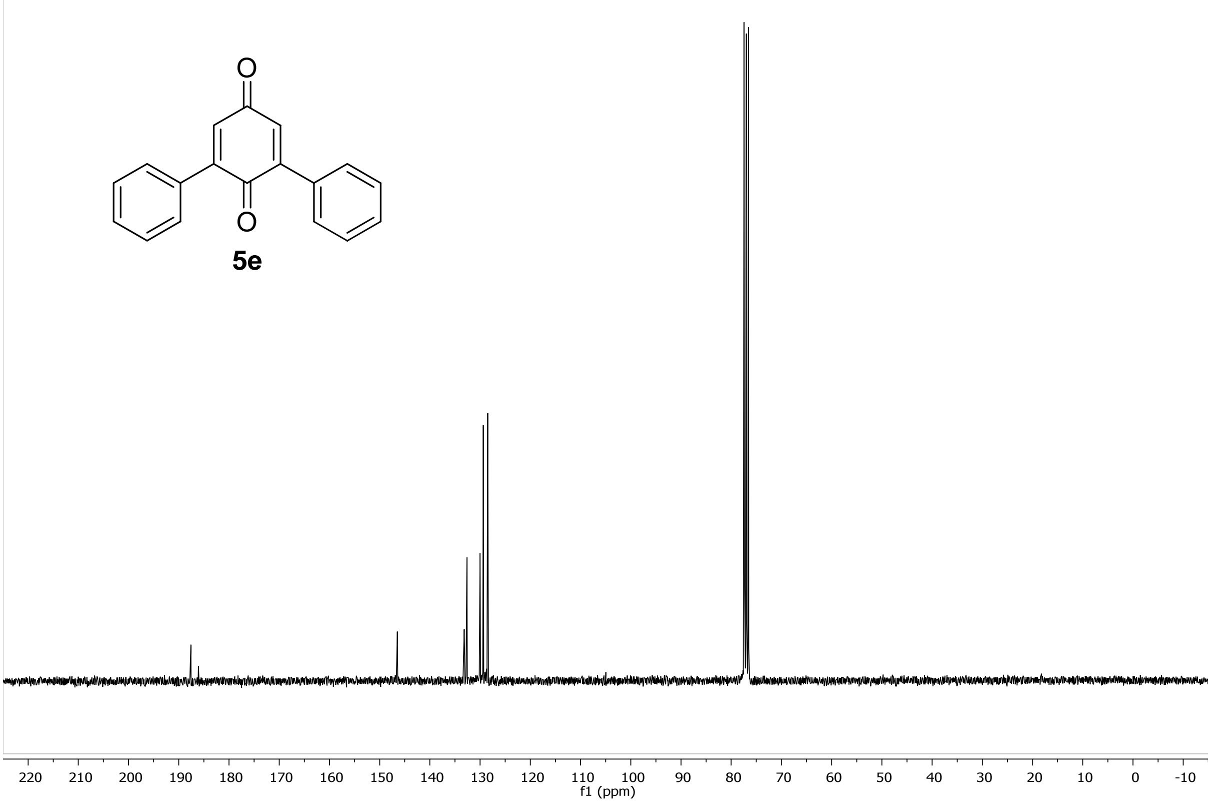

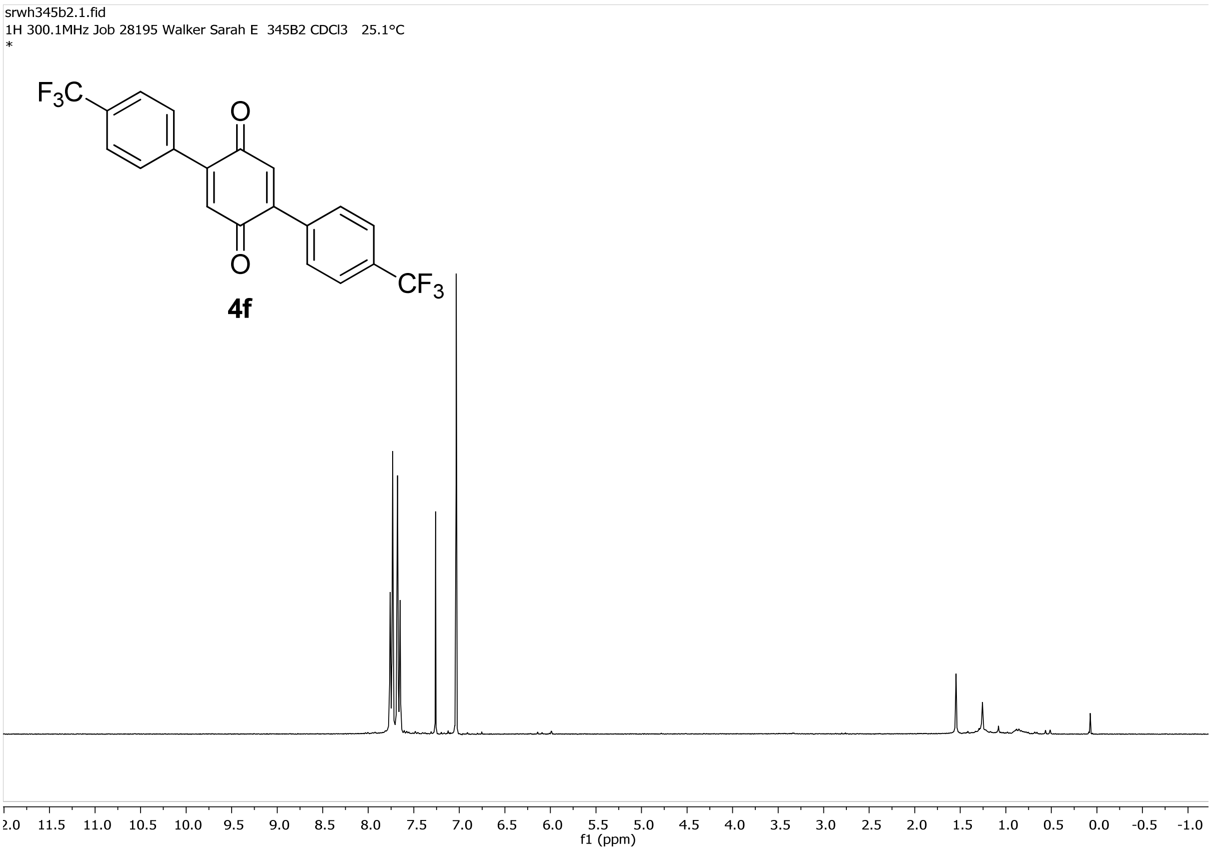
srwh345b2.1.fid
1H 300.1MHz Job 28195 Walker Sarah E 345B2 CDCl3 25.1°C
4f
f1 (ppm)

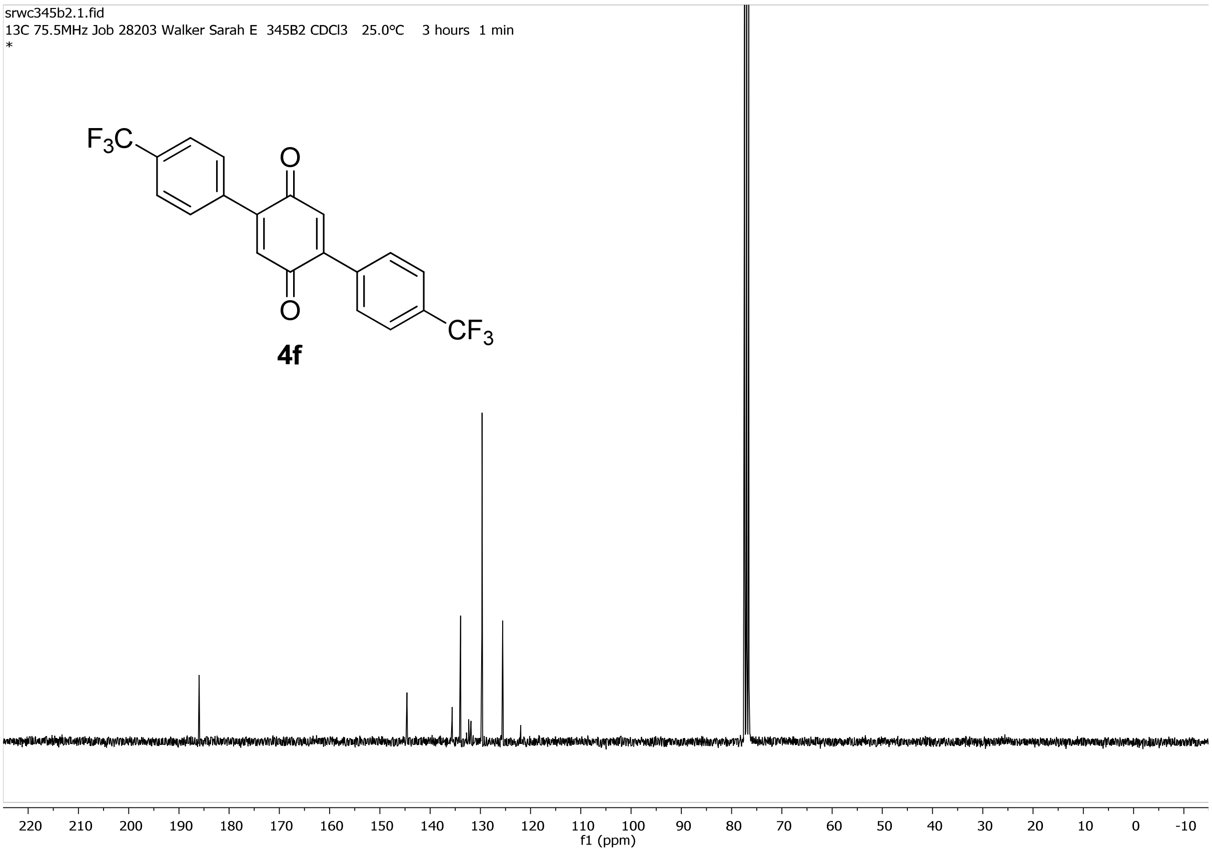
srwc345b2.1.fid
13C 75.5MHz Job 28203 Walker Sarah E 345B2 CDCl3 25.0°C 3 hours 1 min
4f
f1 (ppm)

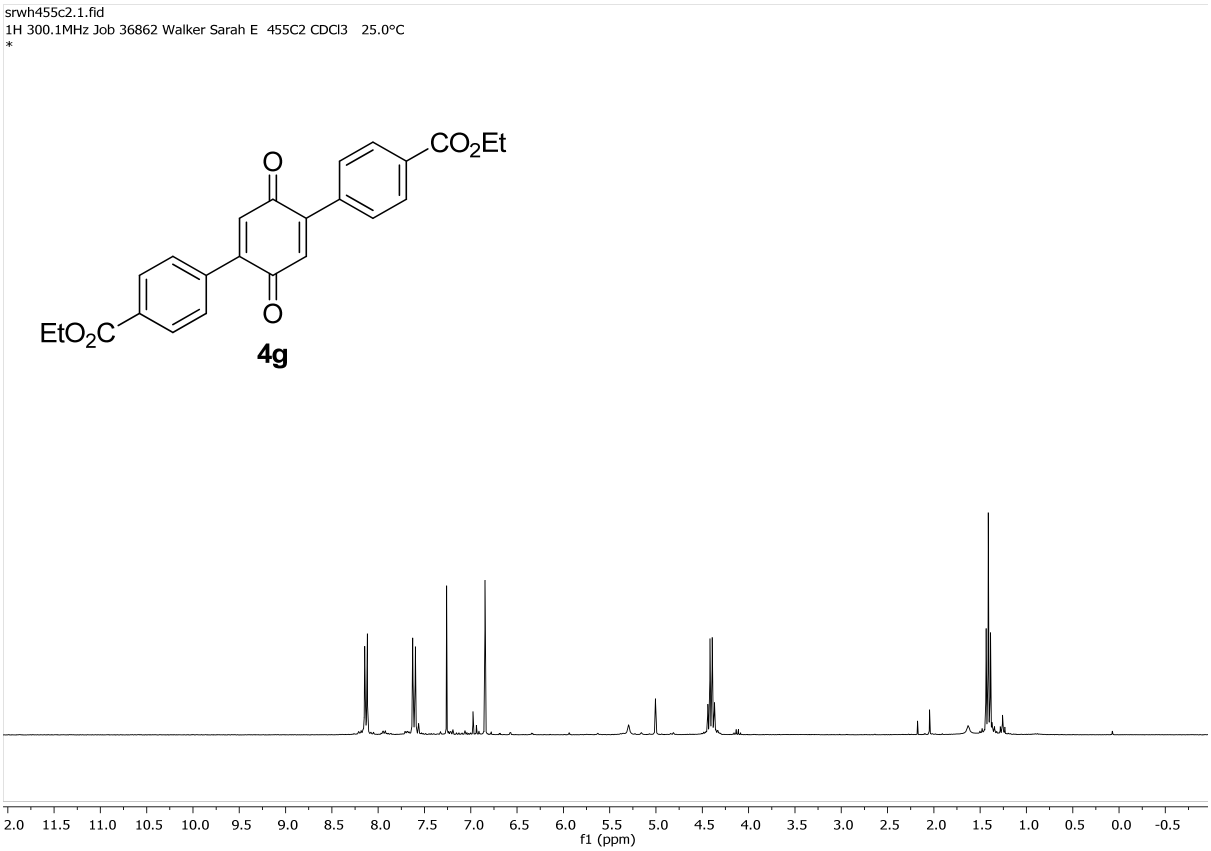
srwh455c2.1.fid
1H 300.1MHz Job 36862 Walker Sarah E 455C2 CDCl3 25.0°C
*
CO2Et
EtO2C
O
O
4g
2.0 11.5 11.0 10.5 10.0 9.5 9.0 8.5 8.0 7.5 7.0 6.5 6.0 5.5 5.0 4.5 4.0 3.5 3.0 2.5 2.0 1.5 1.0 0.5 0.0 -0.5
f1 (ppm)

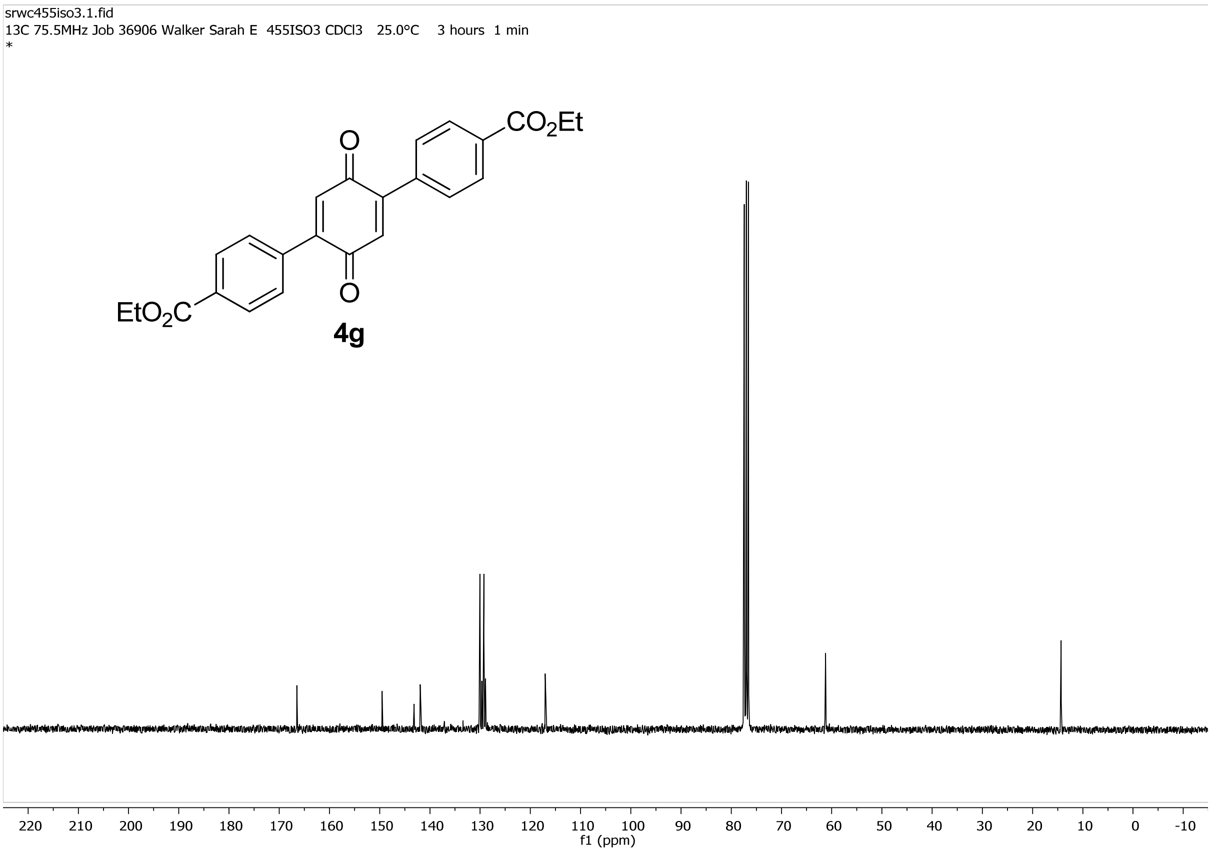
srwc455iso3.1.fid
13C 75.5MHz Job 36906 Walker Sarah E 455ISO3 CDCl3 25.0°C 3 hours 1 min
*
CO2Et
EtO2C
O
O
4g
220 210 200 190 180 170 160 150 140 130 120 110 100 90 80 70 60 50 40 30 20 10 0 -10
f1 (ppm)

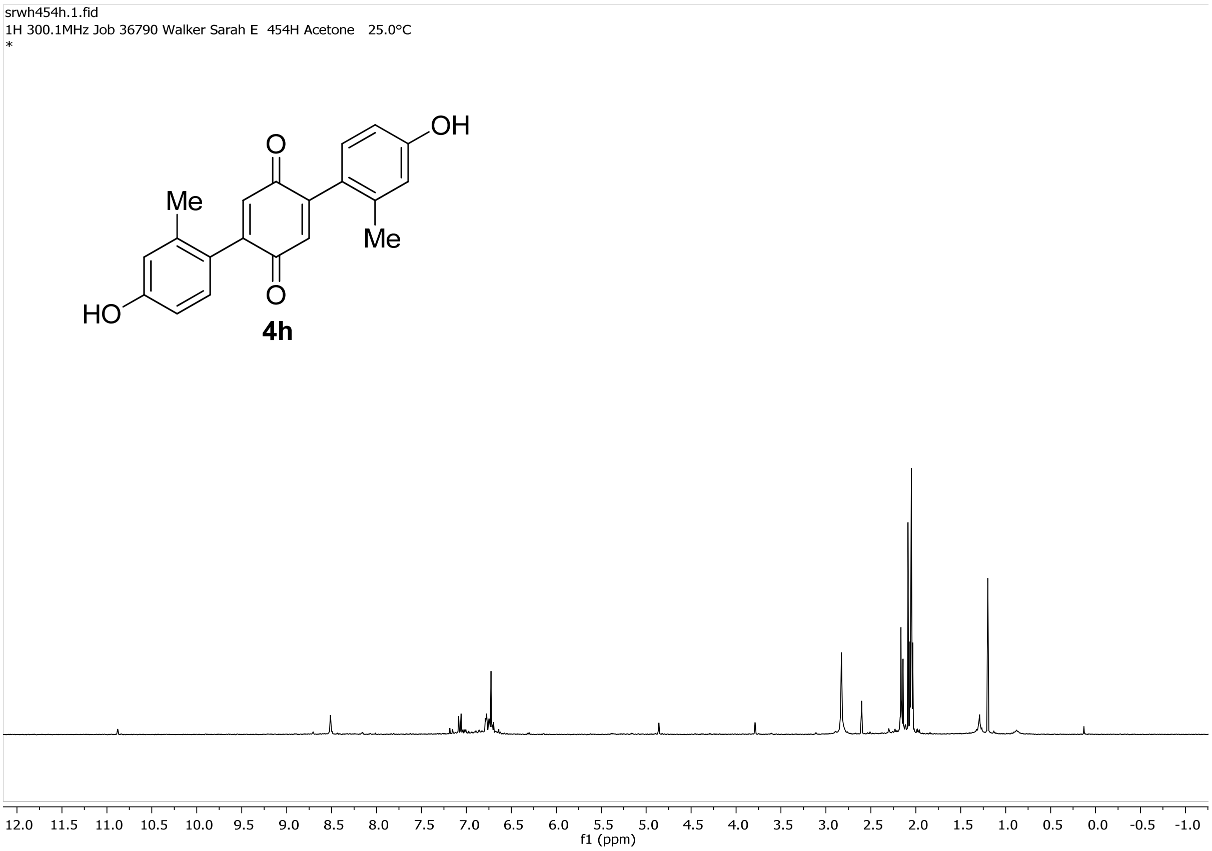
srwh454h.1.fid
1H 300.1MHz Job 36790 Walker Sarah E 454H Acetone 25.0°C
*
OH
O
Me
Me
O
HO
4h
12.0 11.5 11.0 10.5 10.0 9.5 9.0 8.5 8.0 7.5 7.0 6.5 6.0 5.5 5.0 4.5 4.0 3.5 3.0 2.5 2.0 1.5 1.0 0.5 0.0 -0.5 -1.0
f1 (ppm)

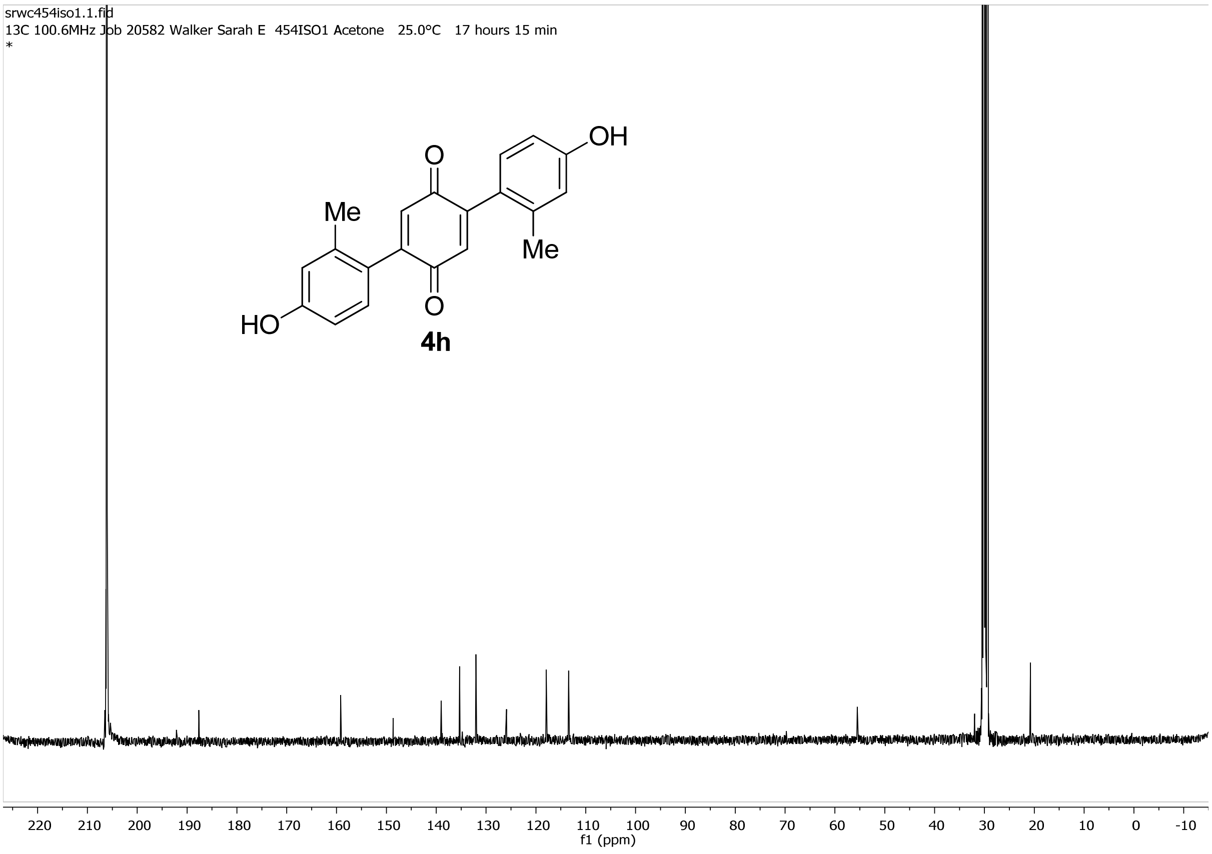
srwc454iso1.1.fid
13C 100.6MHz Job 20582 Walker Sarah E 454ISO1 Acetone 25.0°C 17 hours 15 min
*
OH
O
Me
Me
O
HO
4h
220 210 200 190 180 170 160 150 140 130 120 110 100 90 80 70 60 50 40 30 20 10 0 -10
f1 (ppm)

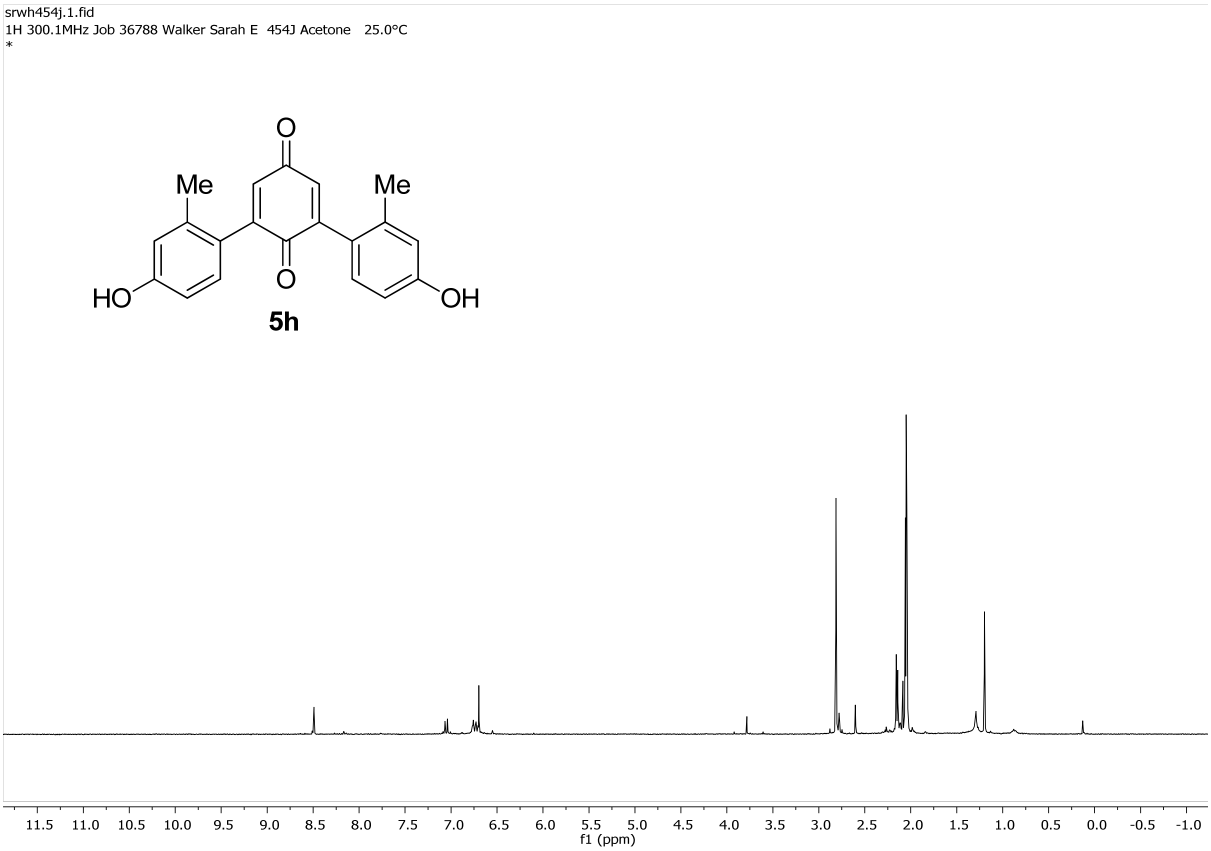
srwh454j.1.fid
1H 300.1MHz Job 36788 Walker Sarah E 454J Acetone 25.0°C
*
O
Me
Me
O
HO
OH
5h
11.5 11.0 10.5 10.0 9.5 9.0 8.5 8.0 7.5 7.0 6.5 6.0 5.5 5.0 4.5 4.0 3.5 3.0 2.5 2.0 1.5 1.0 0.5 0.0 -0.5 -1.0
f1 (ppm)

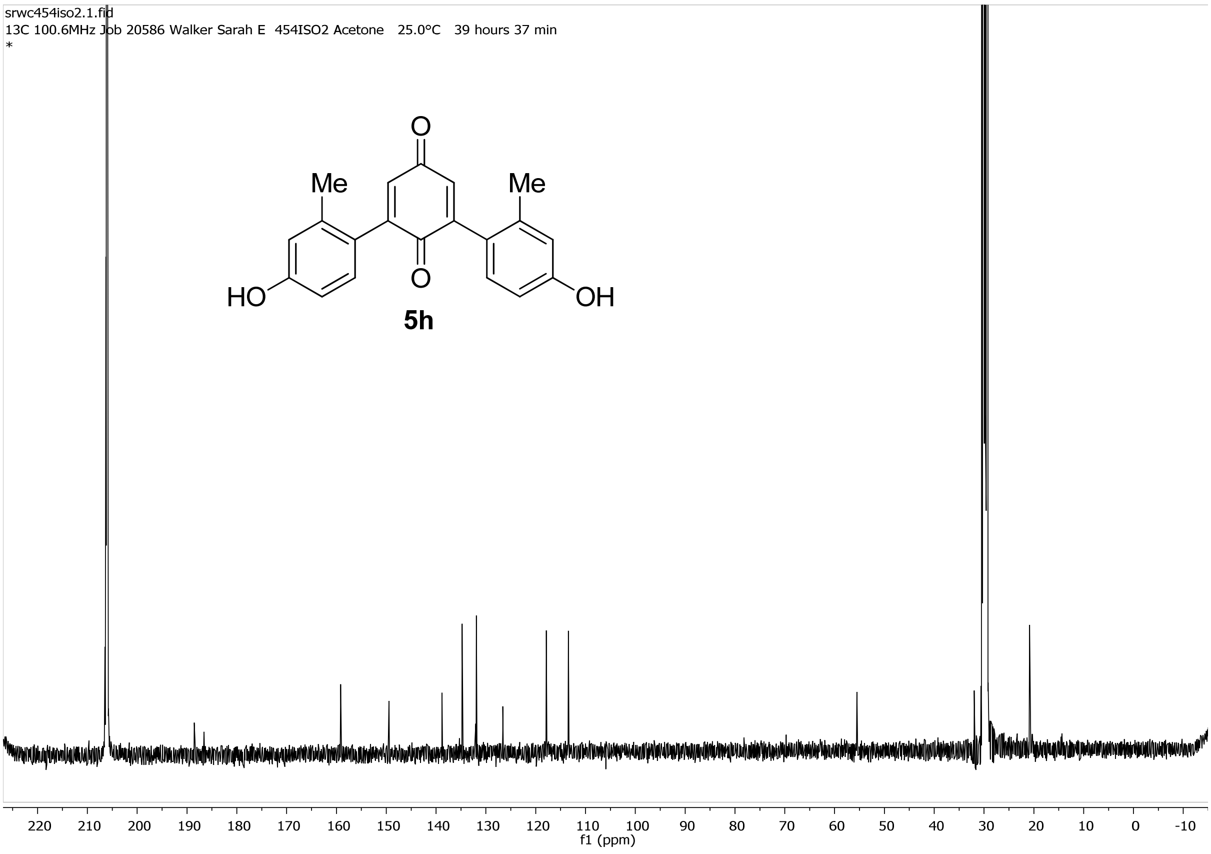
srwc454iso2.1.fid
13C 100.6MHz Job 20586 Walker Sarah E 454ISO2 Acetone 25.0°C 39 hours 37 min
*
O
Me
Me
O
HO
OH
5h
220 210 200 190 180 170 160 150 140 130 120 110 100 90 80 70 60 50 40 30 20 10 0 -10
f1 (ppm)

srwh441col2e.1.fid
1H 300.1MHz Job 35230 Walker Sarah E 441COL2E CDCl3 25.1°C
*

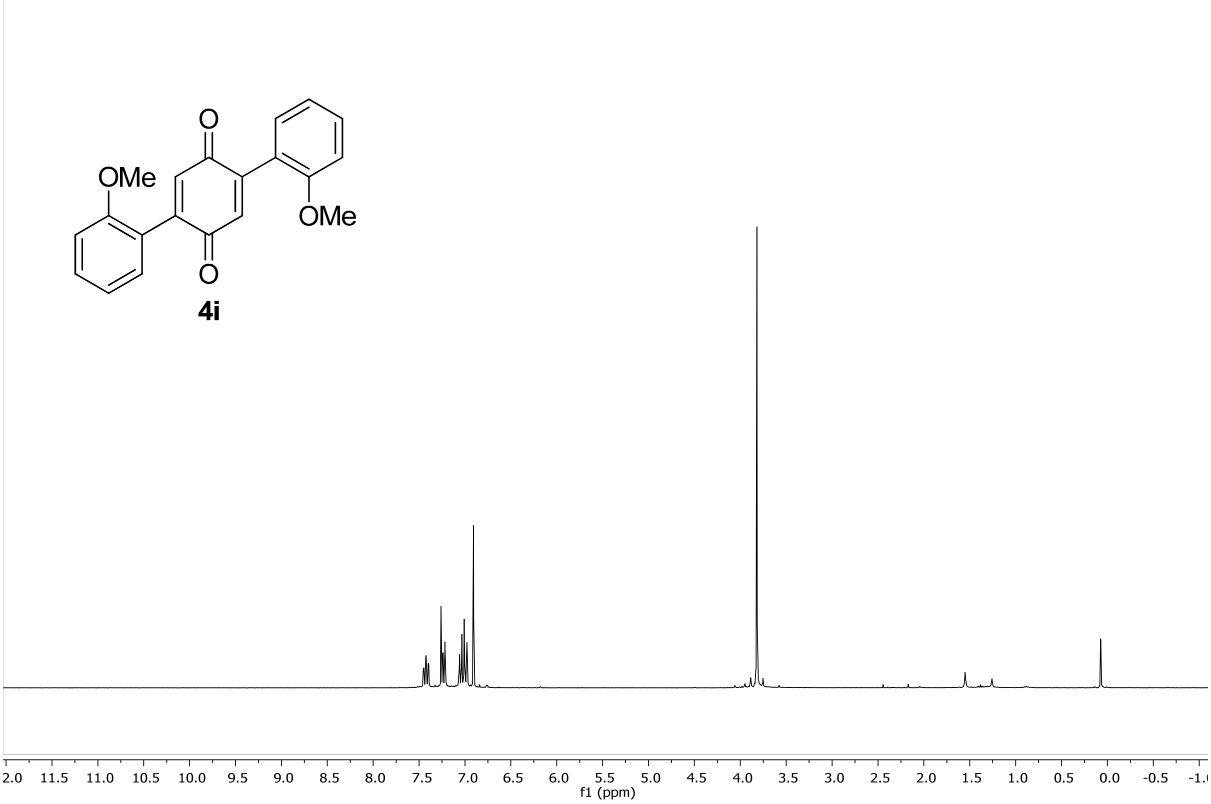

srwc441col2e.1.fid
13C 75.5MHz Job 35351 Walker Sarah E 441COL2E CDCl3 25.0°C 3 hours 1 min
*

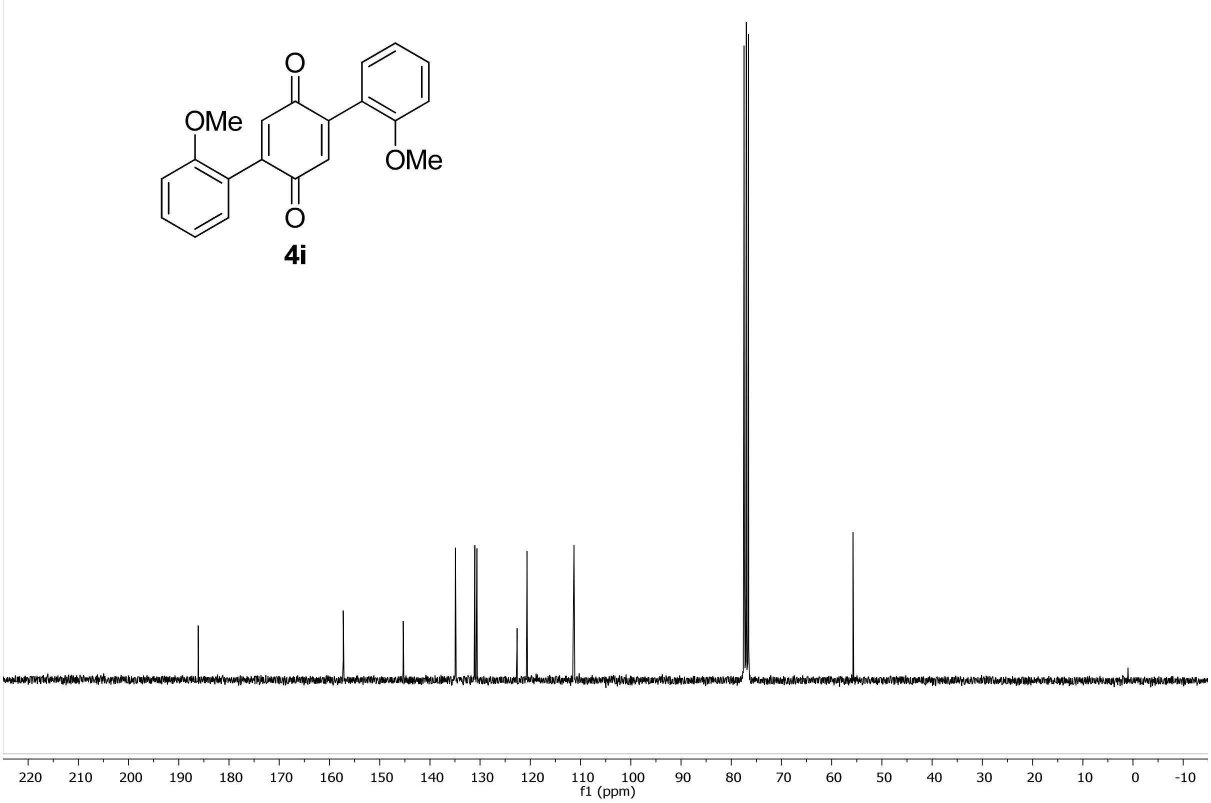

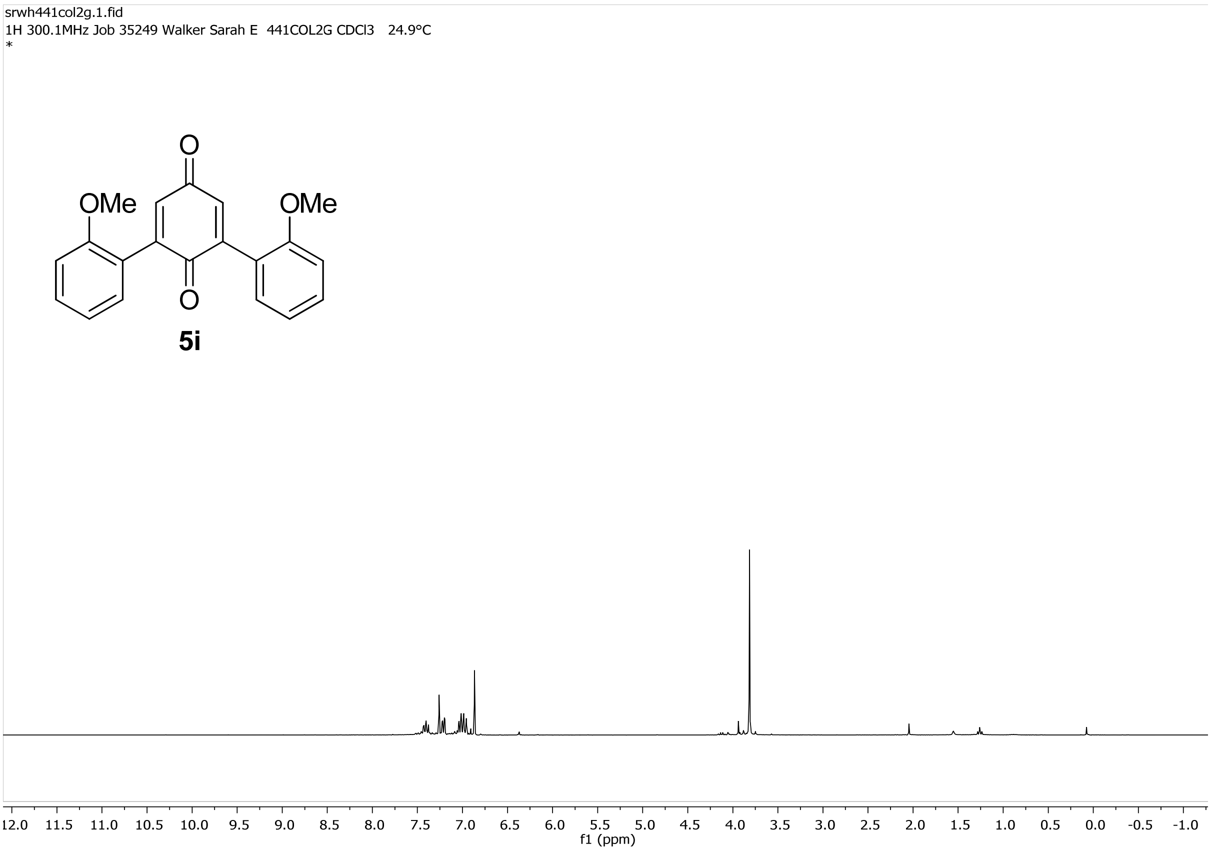
srwh441col2g.1.fid
1H 300.1MHz Job 35249 Walker Sarah E 441COL2G CDCl3 24.9°C
OMe
OMe
5i
f1 (ppm)

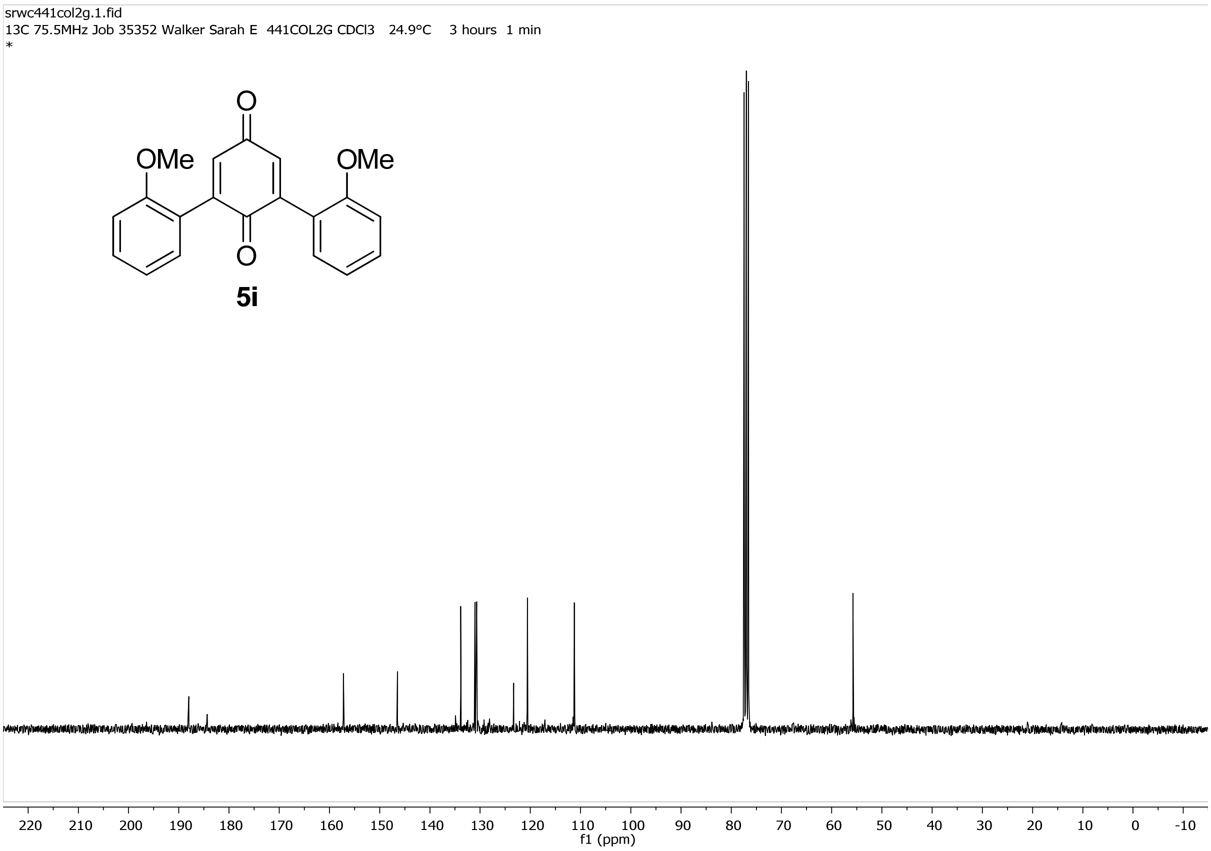
srwc441col2g.1.fid
13C 75.5MHz Job 35352 Walker Sarah E 441COL2G CDCl3 24.9°C 3 hours 1 min
OMe
OMe
5i
f1 (ppm)

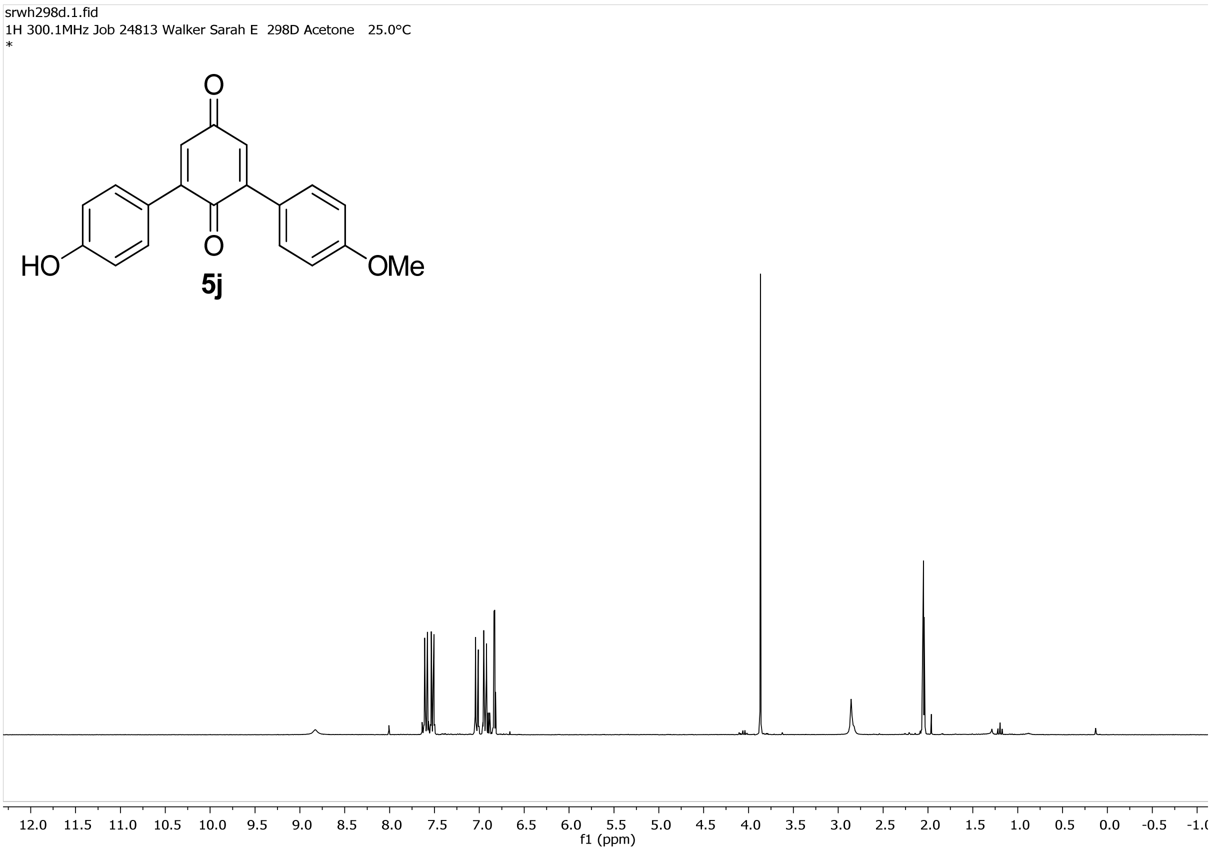
srwh298d.1.fid
1H 300.1MHz Job 24813 Walker Sarah E 298D Acetone 25.0°C
HO
OMe
5j
f1 (ppm)

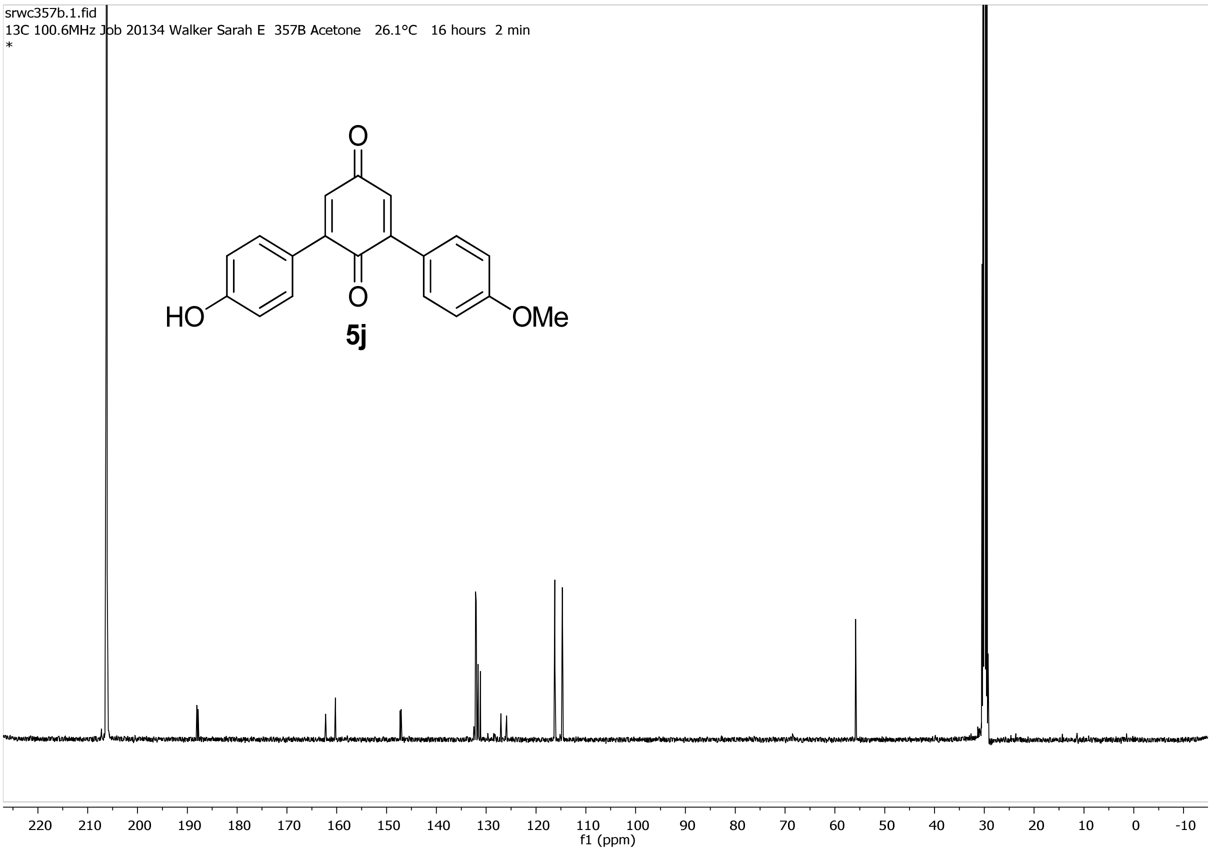
srwc357b.1.fid
13C 100.6MHz Job 20134 Walker Sarah E 357B Acetone 26.1°C 16 hours 2 min
HO
OMe
5j
f1 (ppm)

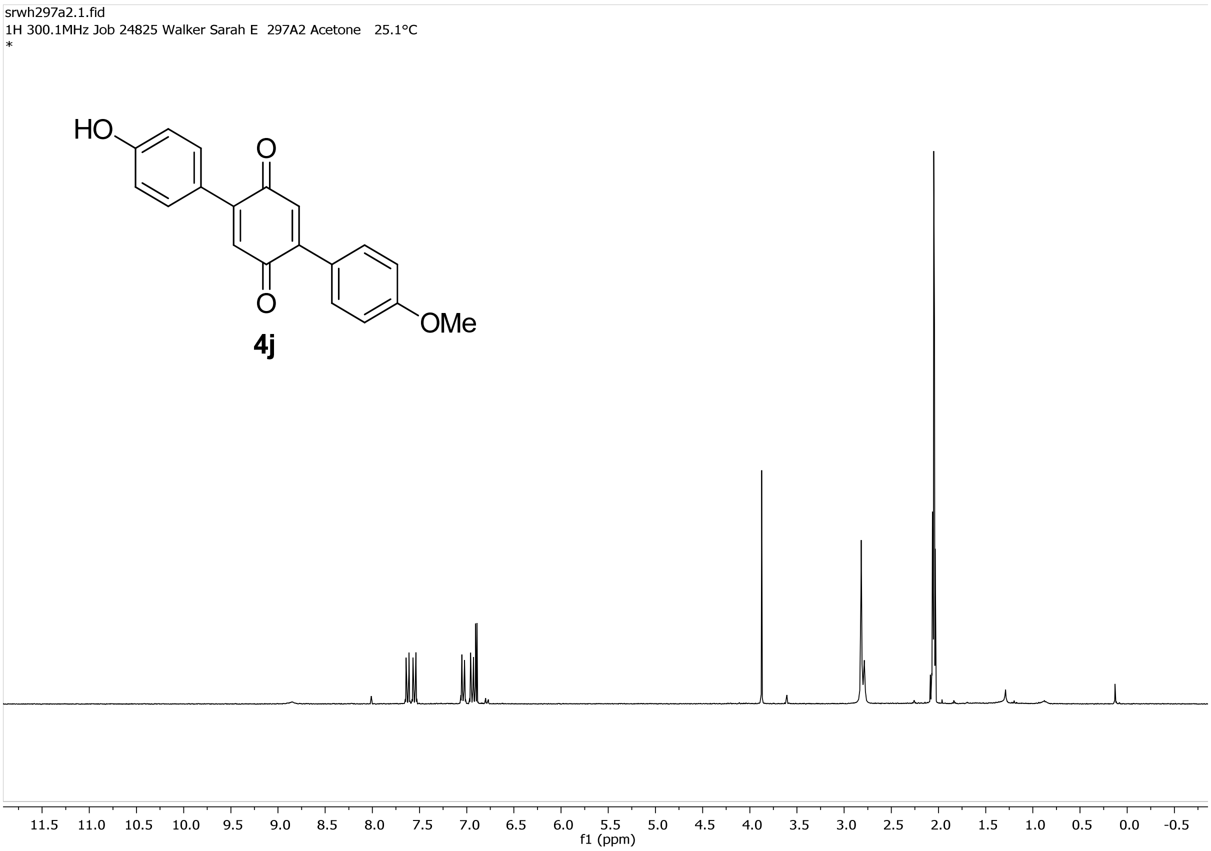
srwh297a2.1.fid
1H 300.1MHz Job 24825 Walker Sarah E 297A2 Acetone 25.1°C
*
HO
O
O
OMe
4j
11.5 11.0 10.5 10.0 9.5 9.0 8.5 8.0 7.5 7.0 6.5 6.0 5.5 5.0 4.5 4.0 3.5 3.0 2.5 2.0 1.5 1.0 0.5 0.0 -0.5
f1 (ppm)

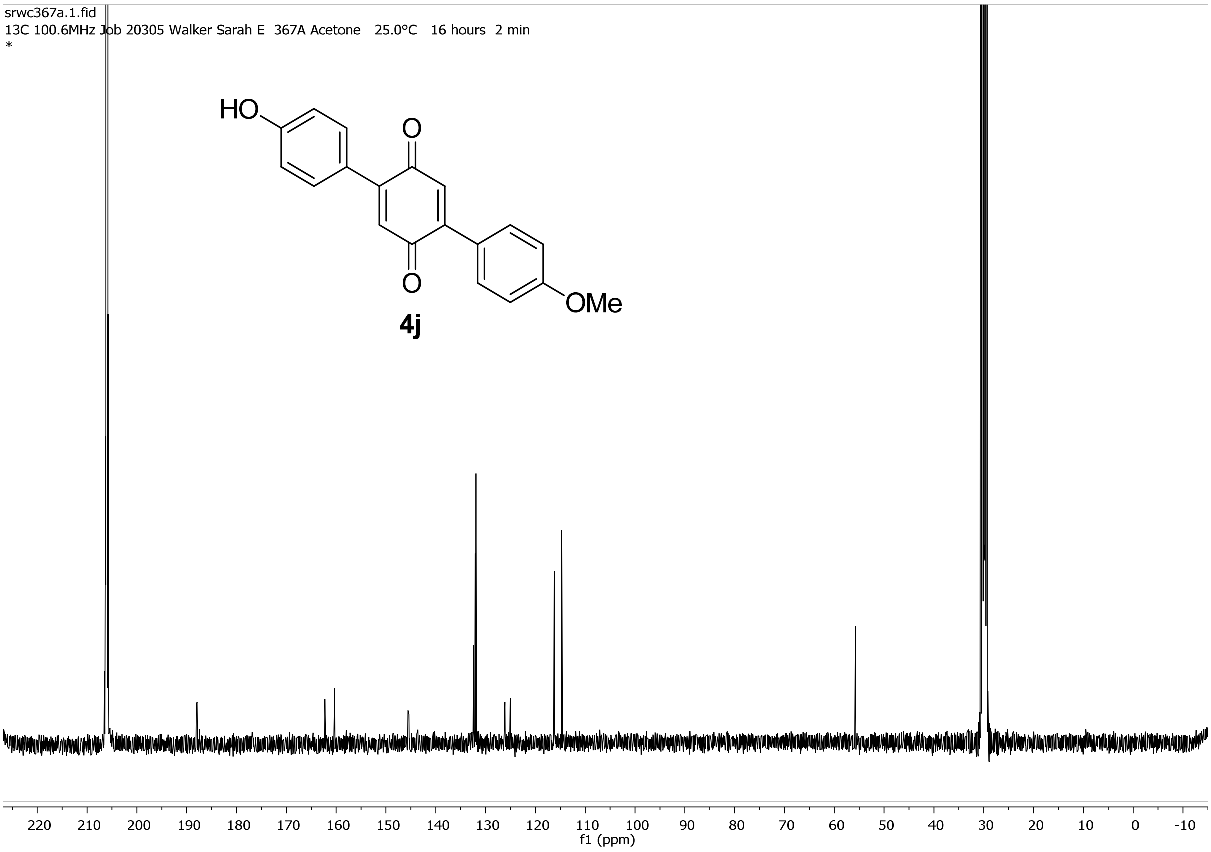
srwc367a.1.fid
13C 100.6MHz Job 20305 Walker Sarah E 367A Acetone 25.0°C 16 hours 2 min
*
HO
O
O
OMe
4j
220 210 200 190 180 170 160 150 140 130 120 110 100 90 80 70 60 50 40 30 20 10 0 -10
f1 (ppm)

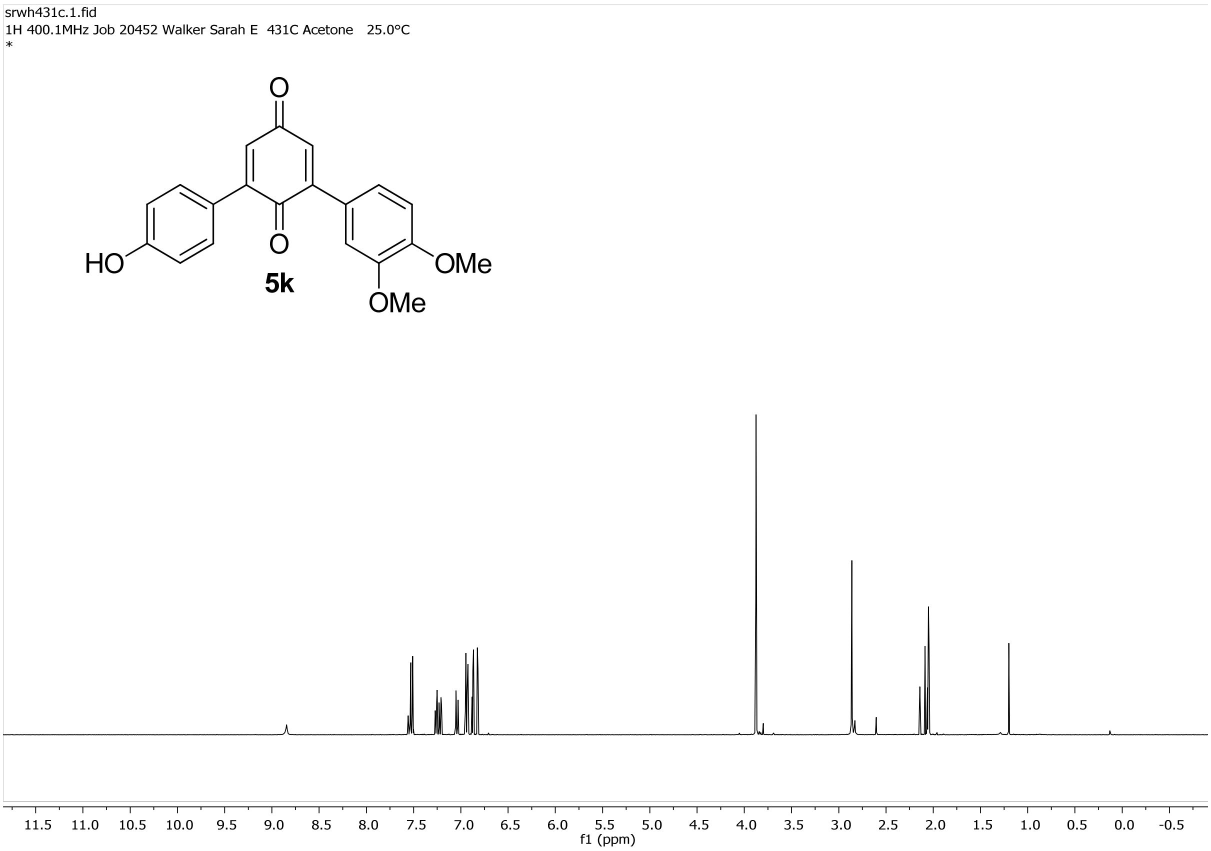
srwh431c.1.fid
1H 400.1MHz Job 20452 Walker Sarah E 431C Acetone 25.0°C
*
O
O
HO
OMe
OMe
5k
11.5 11.0 10.5 10.0 9.5 9.0 8.5 8.0 7.5 7.0 6.5 6.0 5.5 5.0 4.5 4.0 3.5 3.0 2.5 2.0 1.5 1.0 0.5 0.0 -0.5
f1 (ppm)

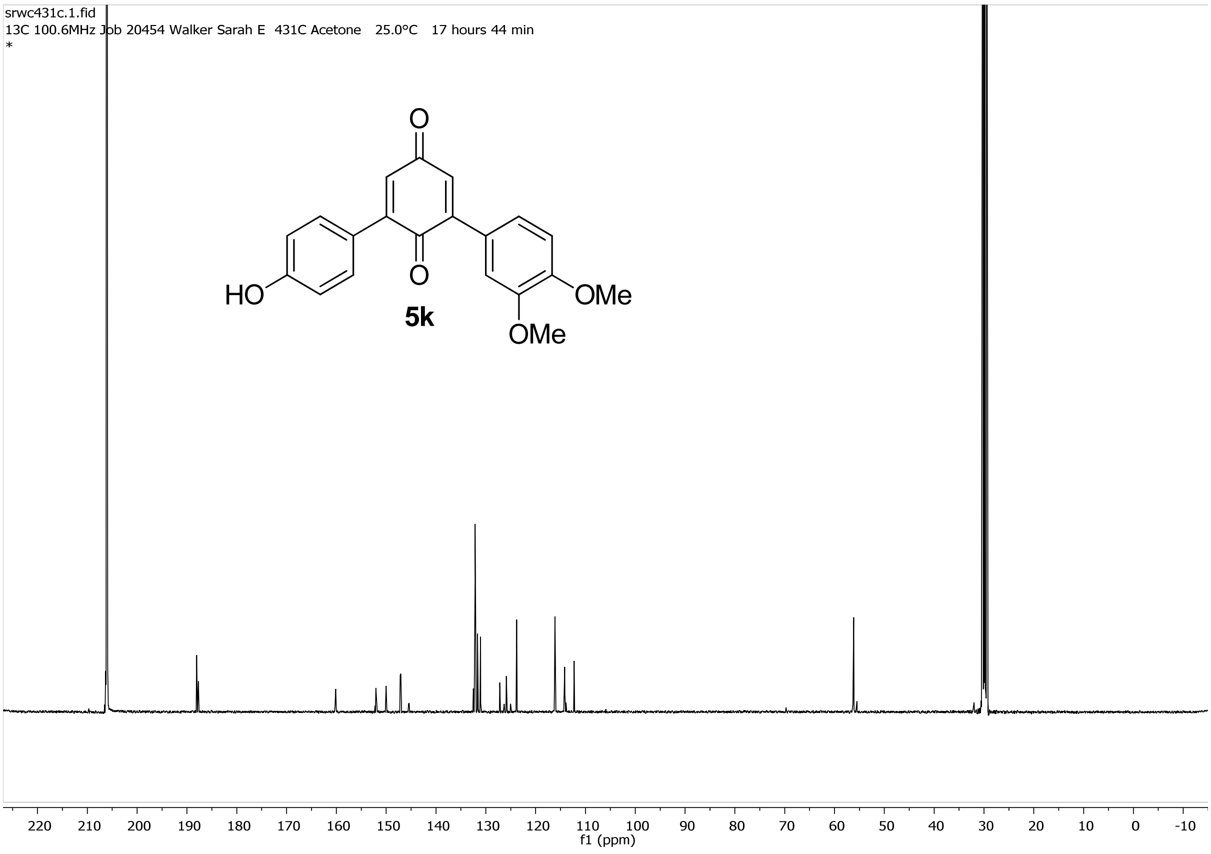
srwc431c.1.fid
13C 100.6MHz Job 20454 Walker Sarah E 431C Acetone 25.0°C 17 hours 44 min
*
O
O
HO
OMe
OMe
5k
220 210 200 190 180 170 160 150 140 130 120 110 100 90 80 70 60 50 40 30 20 10 0 -10
f1 (ppm)

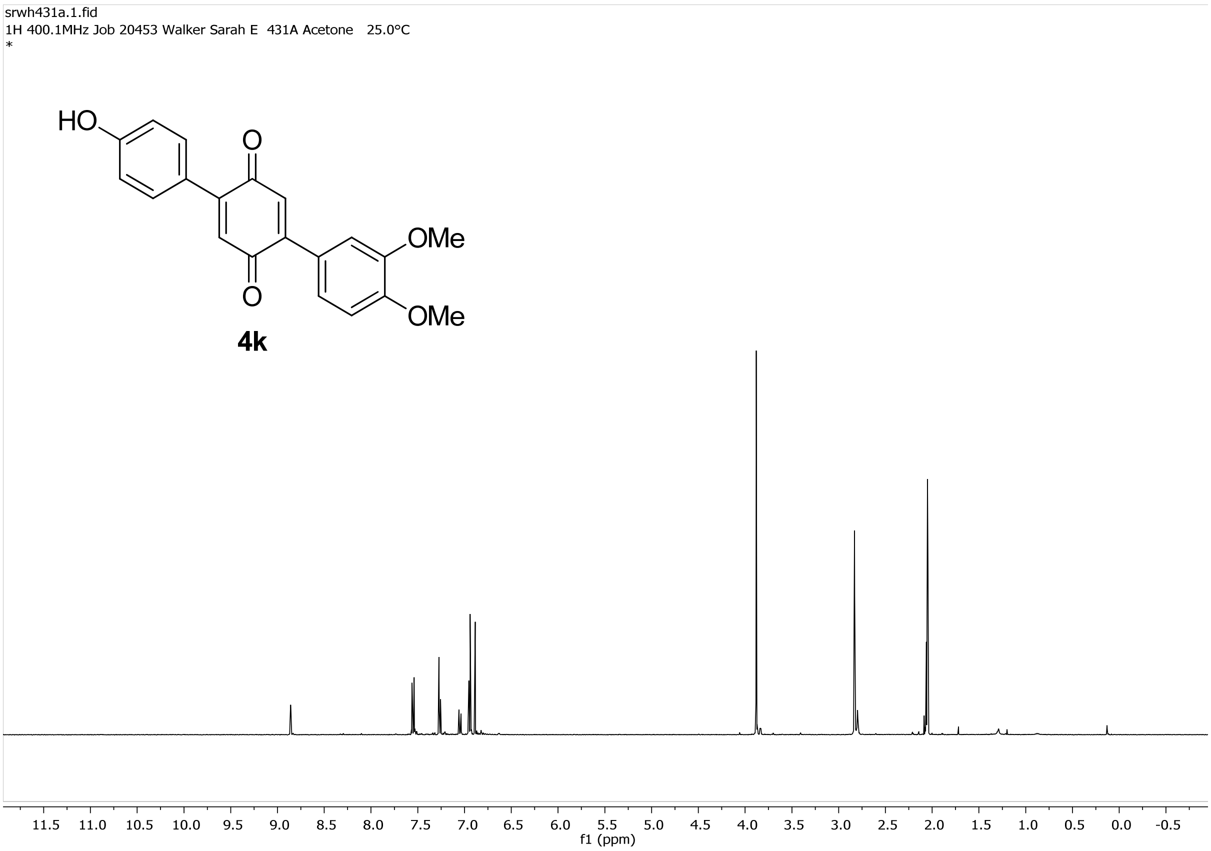
srwh431a.1.fid
1H 400.1MHz Job 20453 Walker Sarah E 431A Acetone 25.0°C
*
HO
O
O
OMe
OMe
4k
11.5 11.0 10.5 10.0 9.5 9.0 8.5 8.0 7.5 7.0 6.5 6.0 5.5 5.0 4.5 4.0 3.5 3.0 2.5 2.0 1.5 1.0 0.5 0.0 -0.5
f1 (ppm)

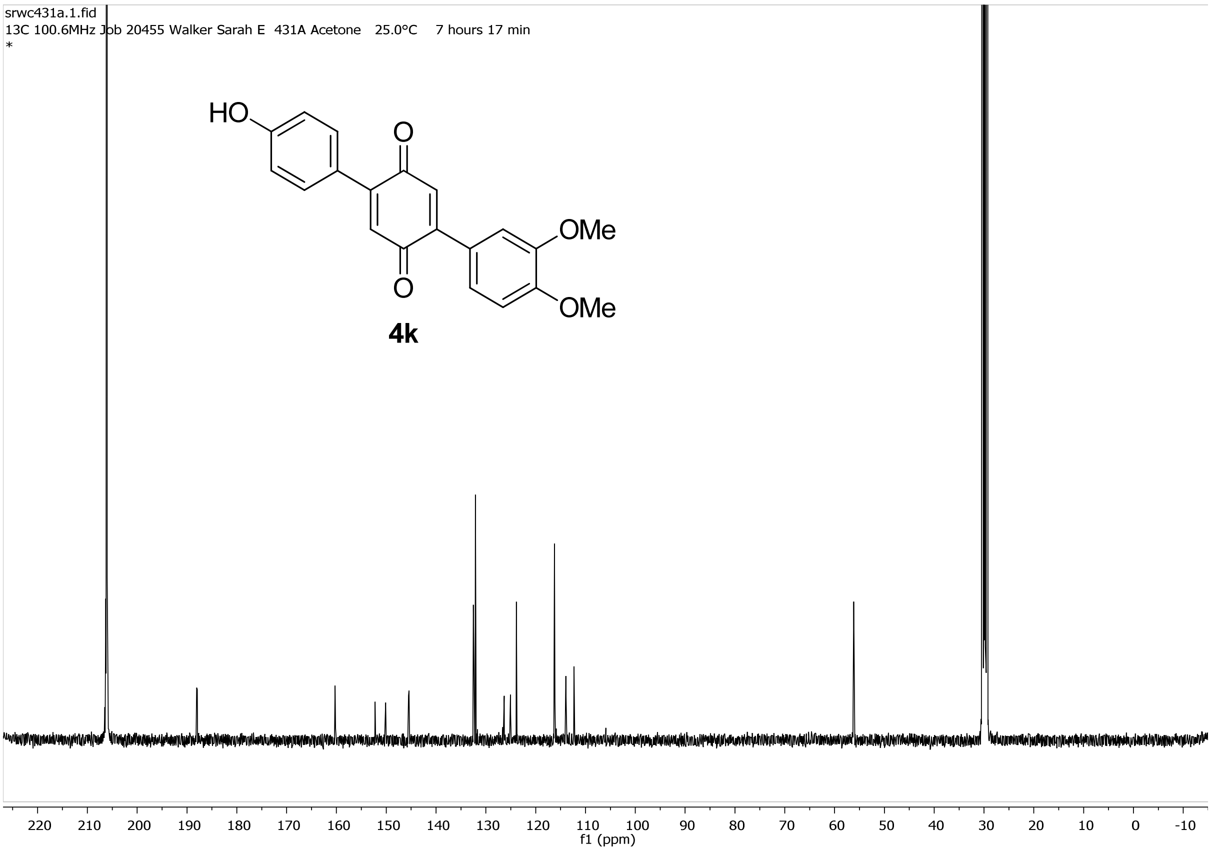
srwc431a.1.fid
13C 100.6MHz Job 20455 Walker Sarah E 431A Acetone 25.0°C 7 hours 17 min
*
HO
O
O
OMe
OMe
4k
220 210 200 190 180 170 160 150 140 130 120 110 100 90 80 70 60 50 40 30 20 10 0 -10
f1 (ppm)

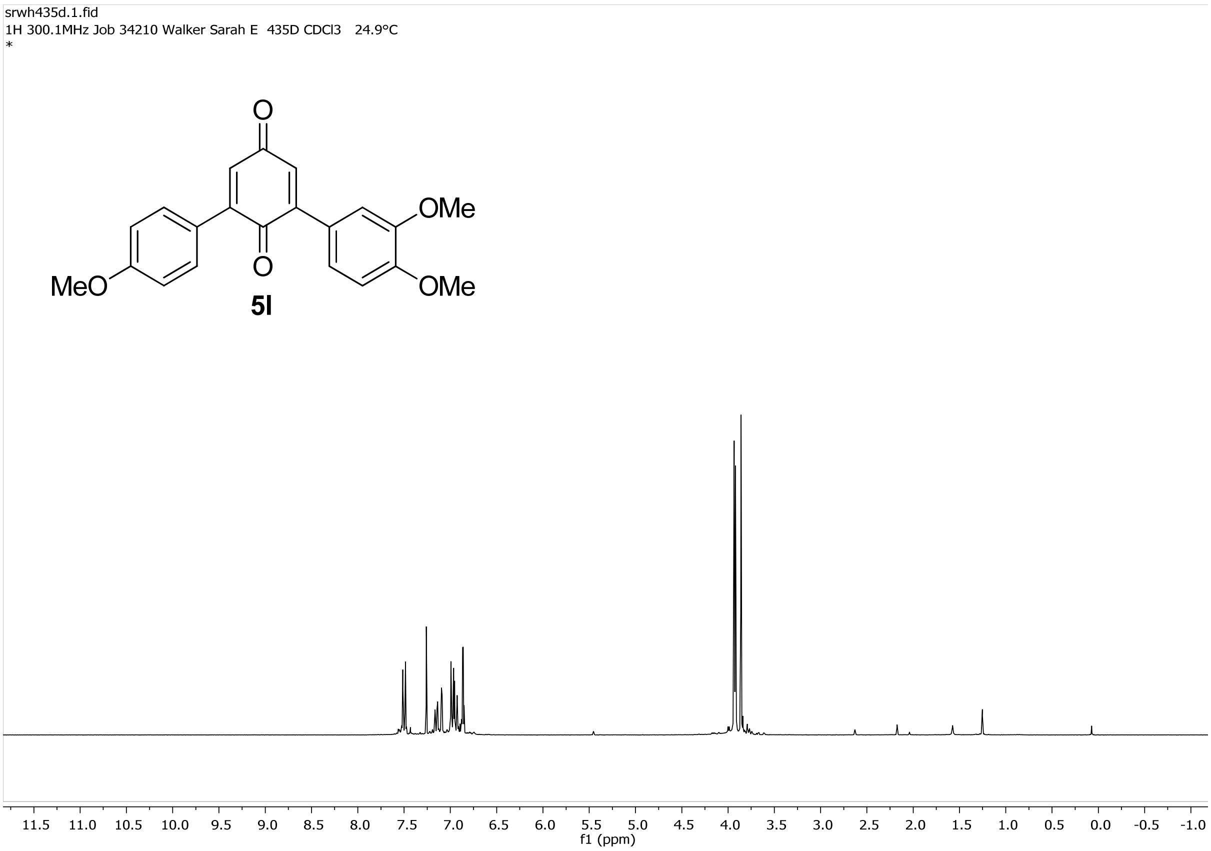
srwh435d.1.fid
1H 300.1MHz Job 34210 Walker Sarah E 435D CDCl3 24.9°C
*
O
O
OMe
OMe
MeO
5l
11.5 11.0 10.5 10.0 9.5 9.0 8.5 8.0 7.5 7.0 6.5 6.0 5.5 5.0 4.5 4.0 3.5 3.0 2.5 2.0 1.5 1.0 0.5 0.0 -0.5 -1.0
f1 (ppm)

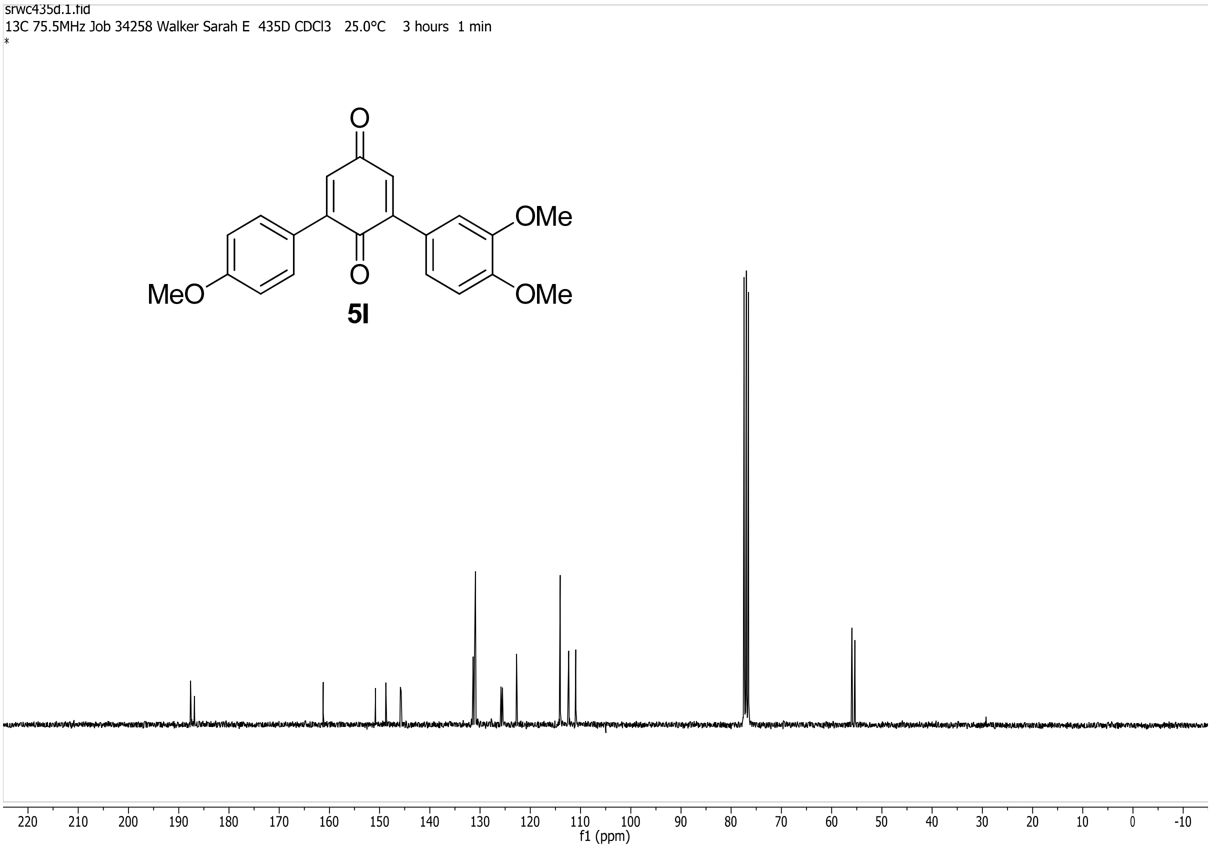
srwc435d.1.fid
13C 75.5MHz Job 34258 Walker Sarah E 435D CDCl3 25.0°C 3 hours 1 min
*
O
O
OMe
OMe
MeO
5l
220 210 200 190 180 170 160 150 140 130 120 110 100 90 80 70 60 50 40 30 20 10 0 -10
f1 (ppm)

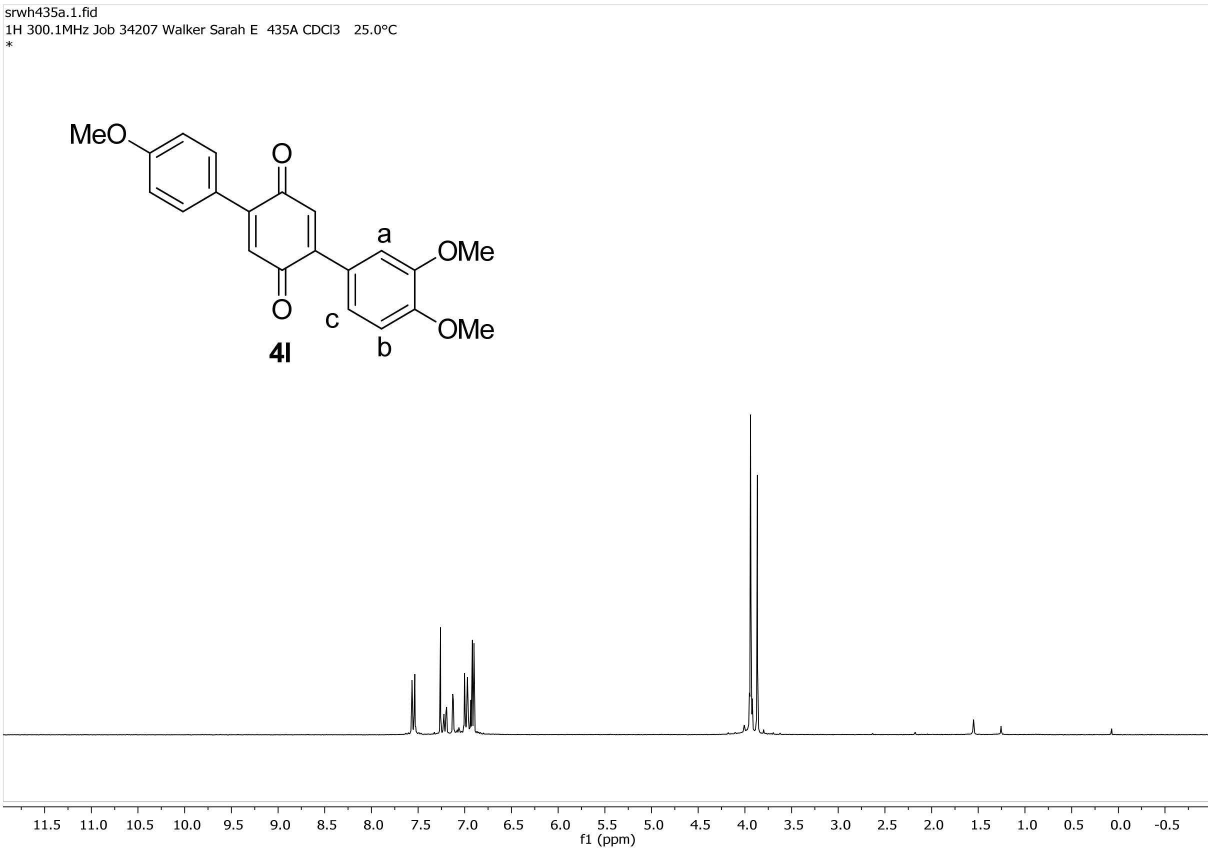
srwh435a.1.fid
1H 300.1MHz Job 34207 Walker Sarah E 435A CDCl3 25.0°C
*
MeO
O
O
a
OMe
c
OMe
b
4l
11.5 11.0 10.5 10.0 9.5 9.0 8.5 8.0 7.5 7.0 6.5 6.0 5.5 5.0 4.5 4.0 3.5 3.0 2.5 2.0 1.5 1.0 0.5 0.0 -0.5
f1 (ppm)

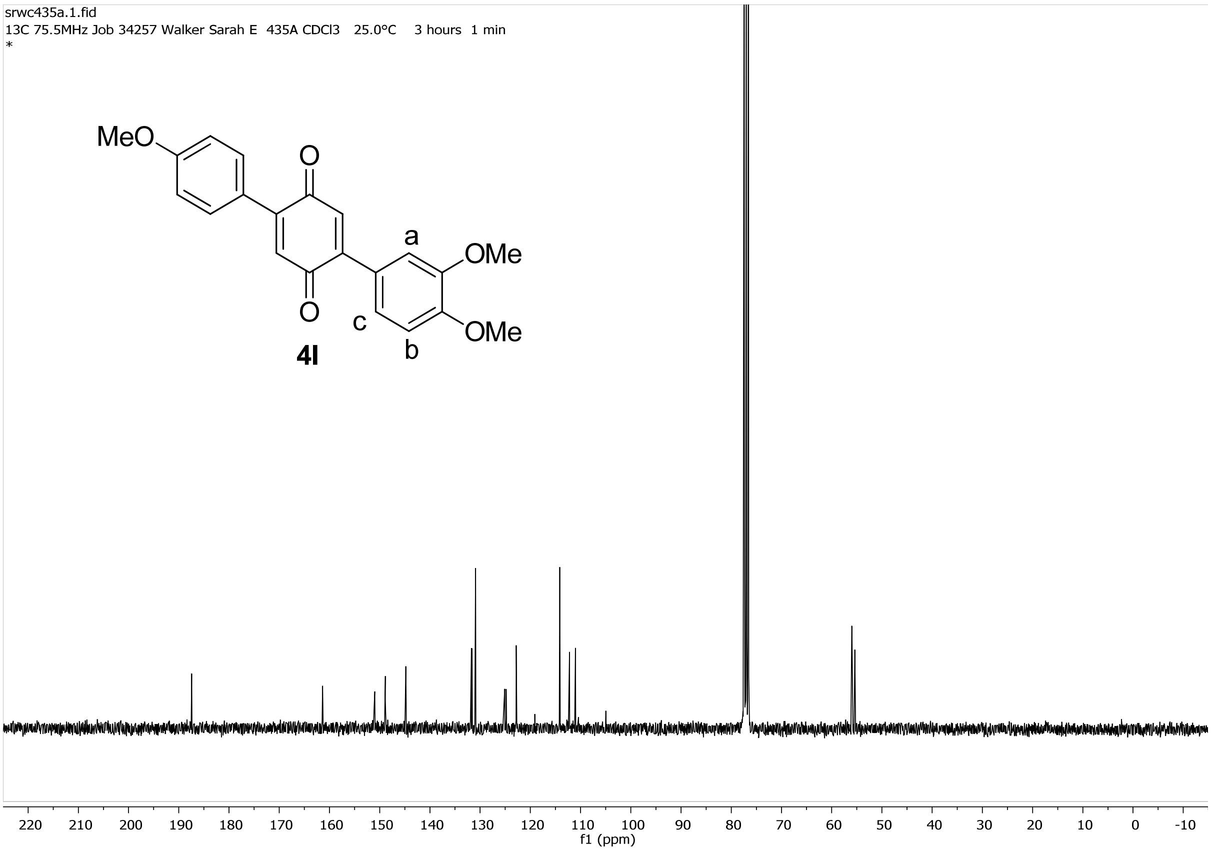
srwc435a.1.fid
13C 75.5MHz Job 34257 Walker Sarah E 435A CDCl3 25.0°C 3 hours 1 min
*
MeO
O
O
a
OMe
c
OMe
b
4l
220 210 200 190 180 170 160 150 140 130 120 110 100 90 80 70 60 50 40 30 20 10 0 -10
f1 (ppm)

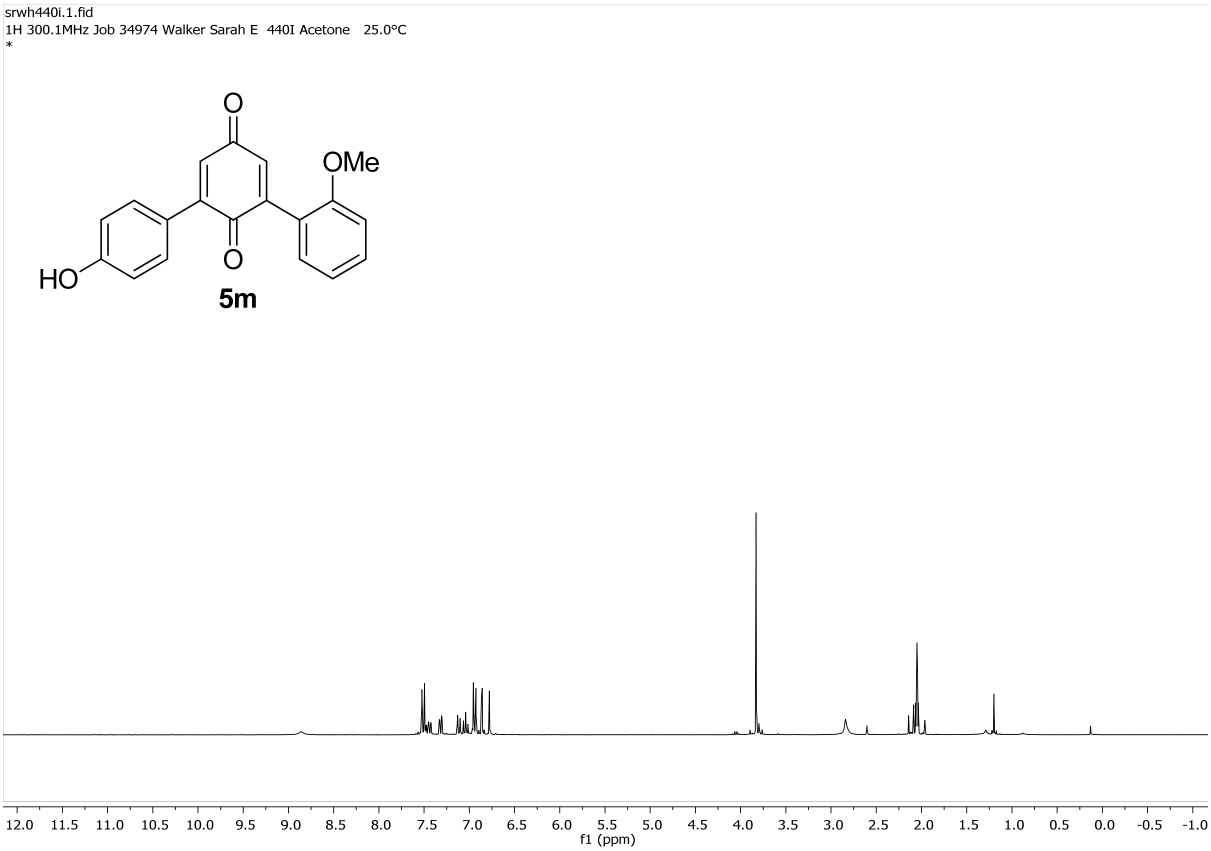
srwh440i.1.fid
1H 300.1MHz Job 34974 Walker Sarah E 440I Acetone 25.0°C
*
OMe
O
O
HO
5m
12.0 11.5 11.0 10.5 10.0 9.5 9.0 8.5 8.0 7.5 7.0 6.5 6.0 5.5 5.0 4.5 4.0 3.5 3.0 2.5 2.0 1.5 1.0 0.5 0.0 -0.5 -1.0
f1 (ppm)

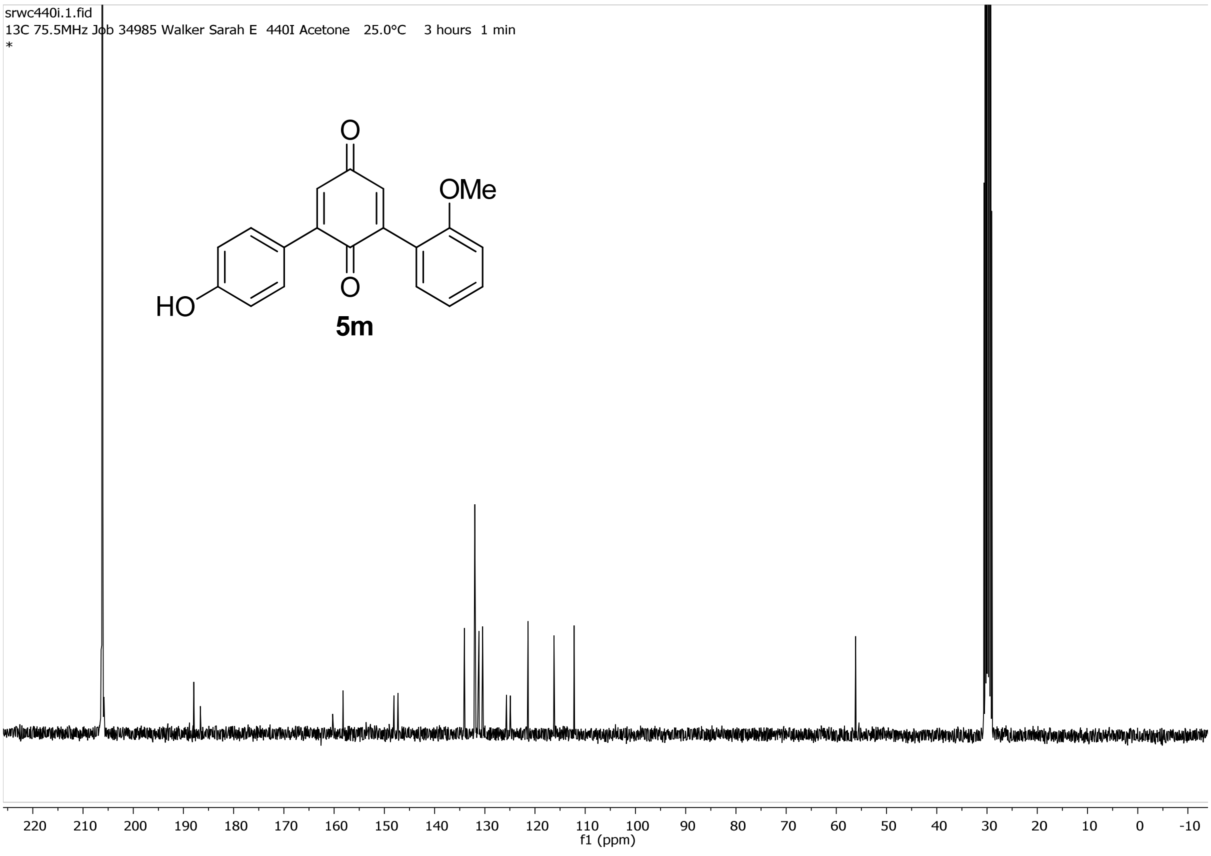
srwc440i.1.fid
13C 75.5MHz Job 34985 Walker Sarah E 440I Acetone 25.0°C 3 hours 1 min
*
OMe
O
O
HO
5m
220 210 200 190 180 170 160 150 140 130 120 110 100 90 80 70 60 50 40 30 20 10 0 -10
f1 (ppm)

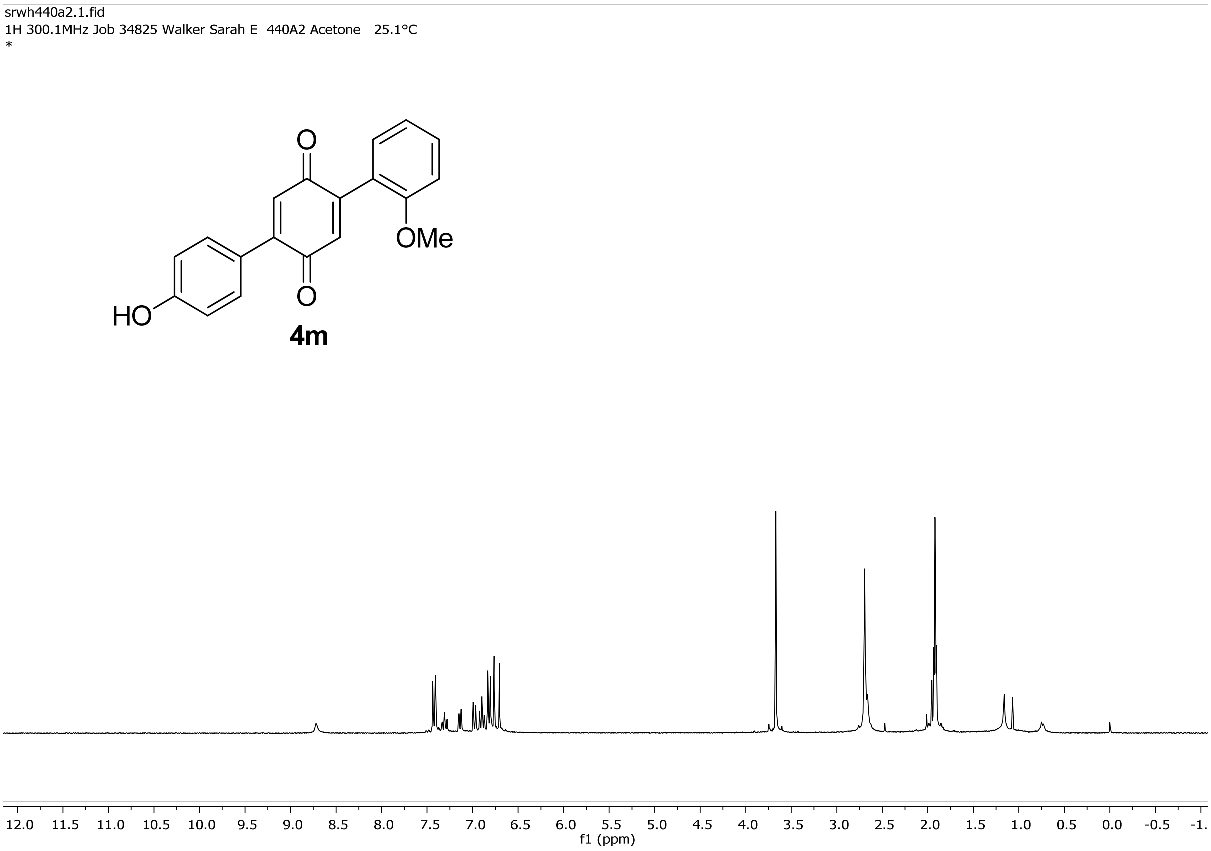
srwh440a2.1.fid
1H 300.1MHz Job 34825 Walker Sarah E 440A2 Acetone 25.1°C
*
O
OMe
O
HO
4m
12.0 11.5 11.0 10.5 10.0 9.5 9.0 8.5 8.0 7.5 7.0 6.5 6.0 5.5 5.0 4.5 4.0 3.5 3.0 2.5 2.0 1.5 1.0 0.5 0.0 -0.5 -1.
f1 (ppm)

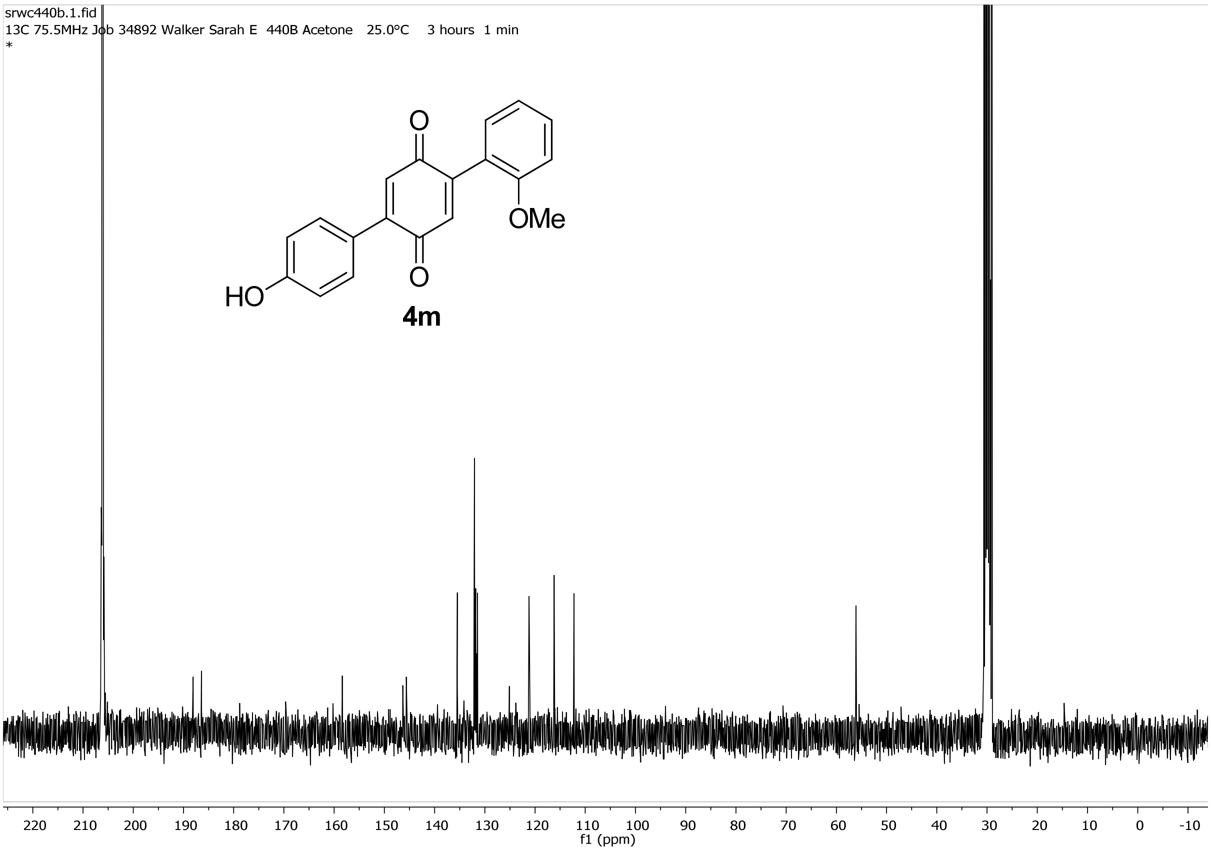
srwc440b.1.fid
13C 75.5MHz Job 34892 Walker Sarah E 440B Acetone 25.0°C 3 hours 1 min
*
O
OMe
O
HO
4m
220 210 200 190 180 170 160 150 140 130 120 110 100 90 80 70 60 50 40 30 20 10 0 -10
f1 (ppm)

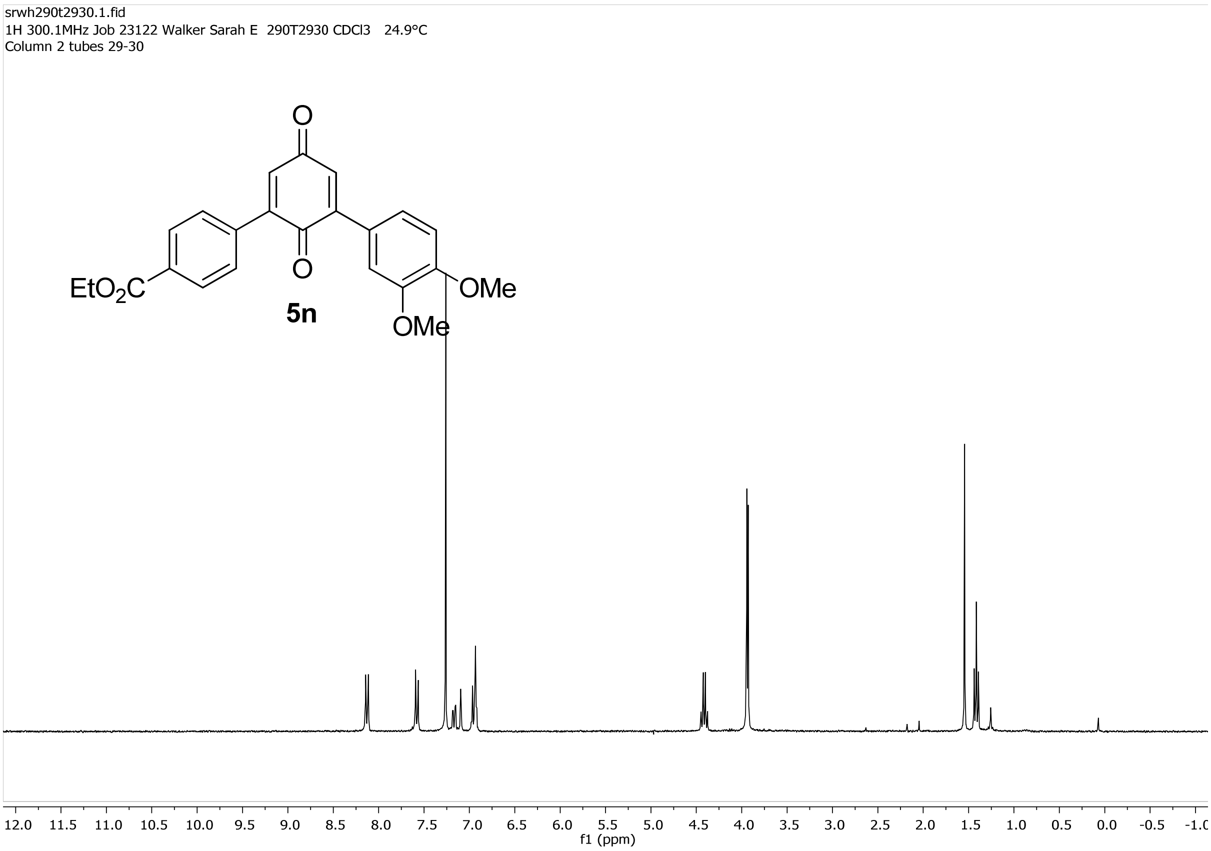
srwh290t2930.1.fid
1H 300.1MHz Job 23122 Walker Sarah E 290T2930 CDCl3 24.9°C
Column 2 tubes 29-30
O
O
EtO2C
OMe
OMe
5n
12.0 11.5 11.0 10.5 10.0 9.5 9.0 8.5 8.0 7.5 7.0 6.5 6.0 5.5 5.0 4.5 4.0 3.5 3.0 2.5 2.0 1.5 1.0 0.5 0.0 -0.5 -1.0
f1 (ppm)

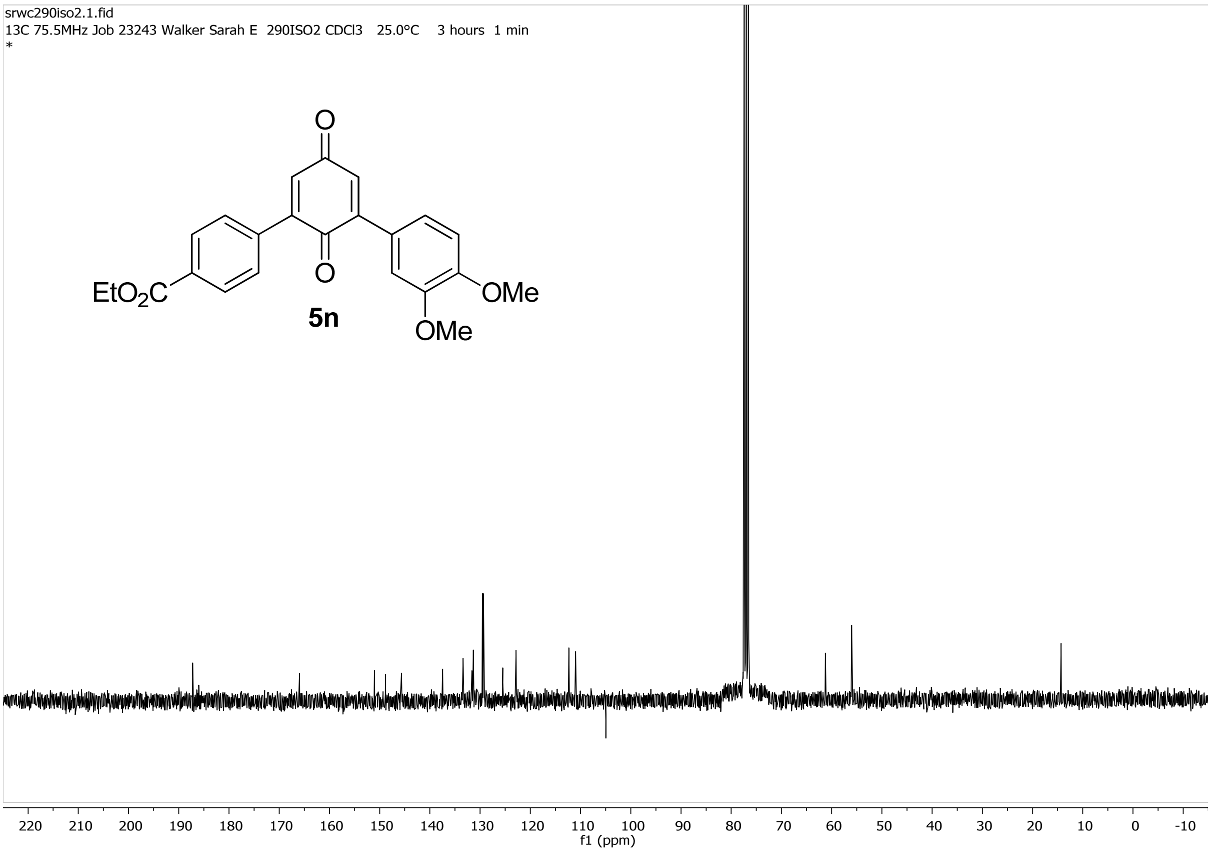
srwc290iso2.1.fid
13C 75.5MHz Job 23243 Walker Sarah E 290ISO2 CDCl3 25.0°C 3 hours 1 min
*
O
O
EtO2C
OMe
OMe
5n
220 210 200 190 180 170 160 150 140 130 120 110 100 90 80 70 60 50 40 30 20 10 0 -10
f1 (ppm)

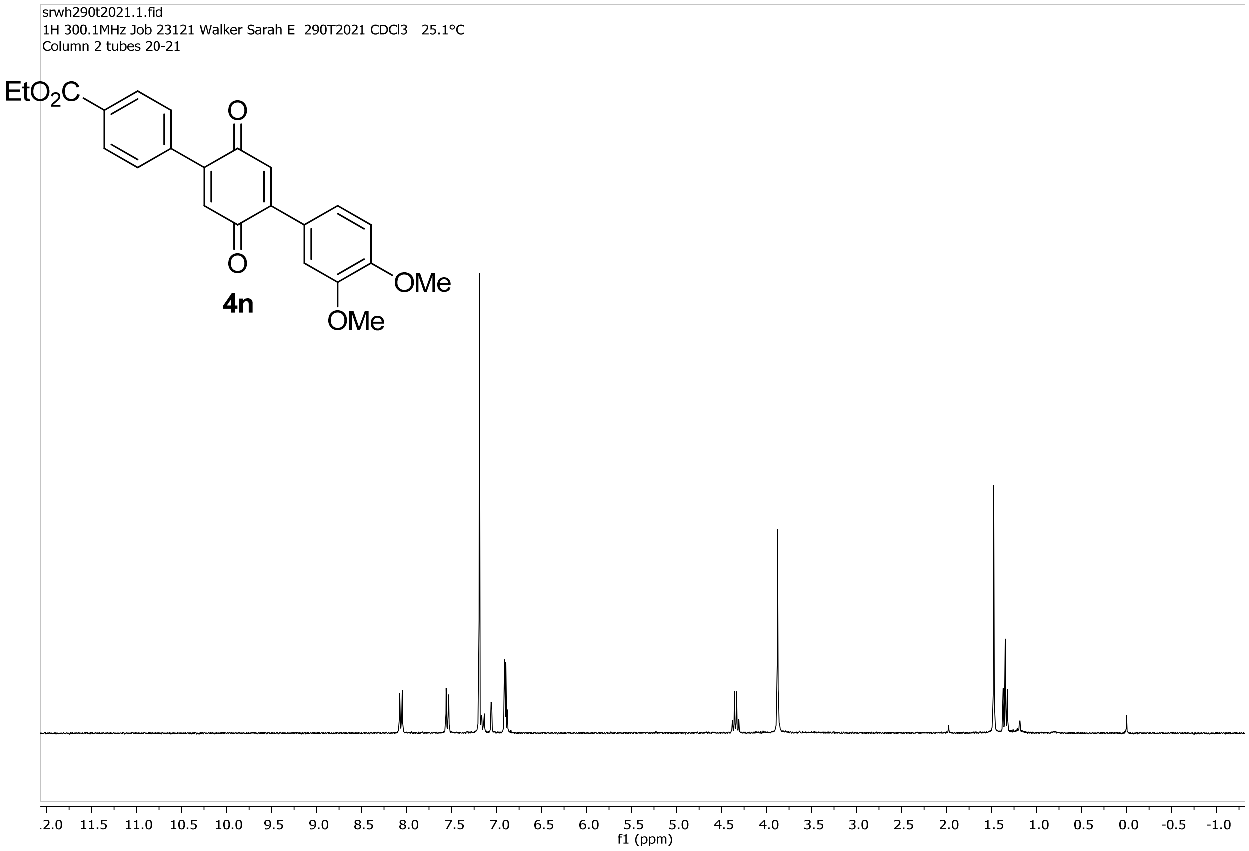
srwh290t2021.1.fid
1H 300.1MHz Job 23121 Walker Sarah E 290T2021 CDCl3 25.1°C
Column 2 tubes 20-21
EtO2C
O
O
OMe
OMe
4n
.2.0 11.5 11.0 10.5 10.0 9.5 9.0 8.5 8.0 7.5 7.0 6.5 6.0 5.5 5.0 4.5 4.0 3.5 3.0 2.5 2.0 1.5 1.0 0.5 0.0 -0.5 -1.0
f1 (ppm)

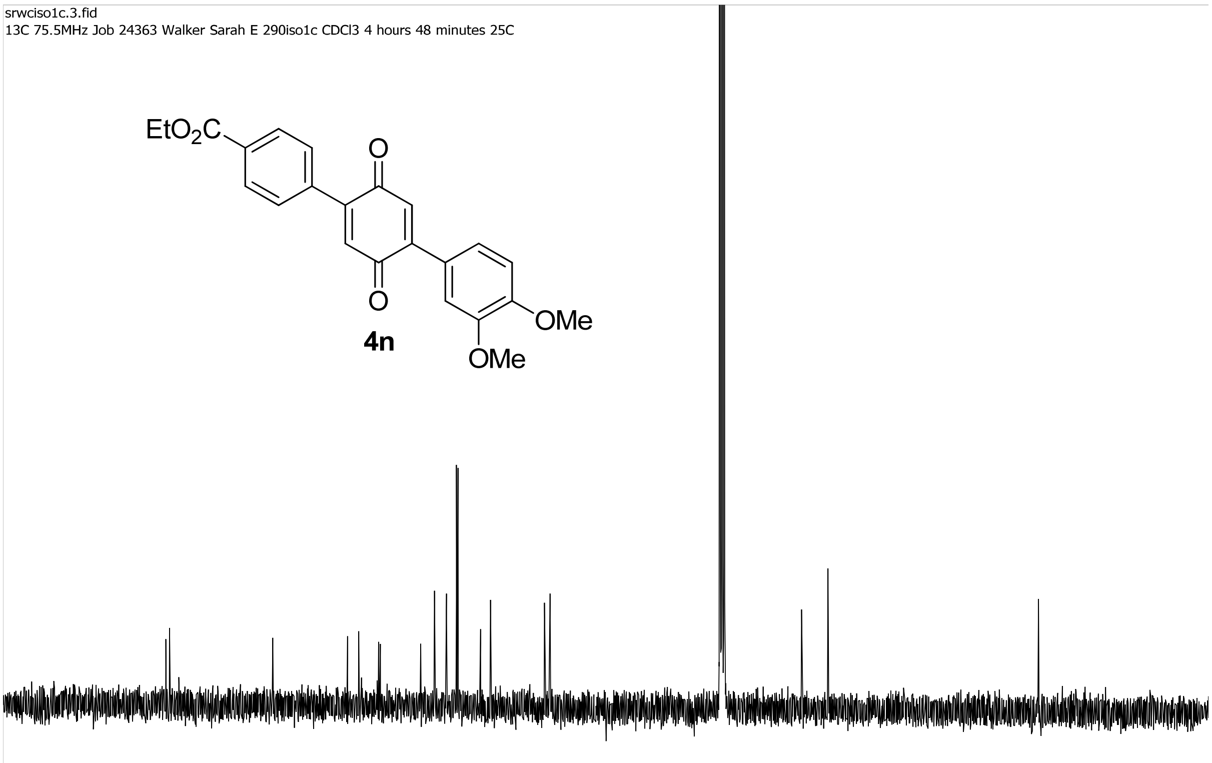
srwciso1c.3.fid
13C 75.5MHz Job 24363 Walker Sarah E 290iso1c CDCl3 4 hours 48 minutes 25C
EtO2C
O
O
OMe
OMe
4n
210 200 190 180 170 160 150 140 130 120 110 100 90 80 70 60 50 40 30 20 10 0 -10
f1 (ppm)

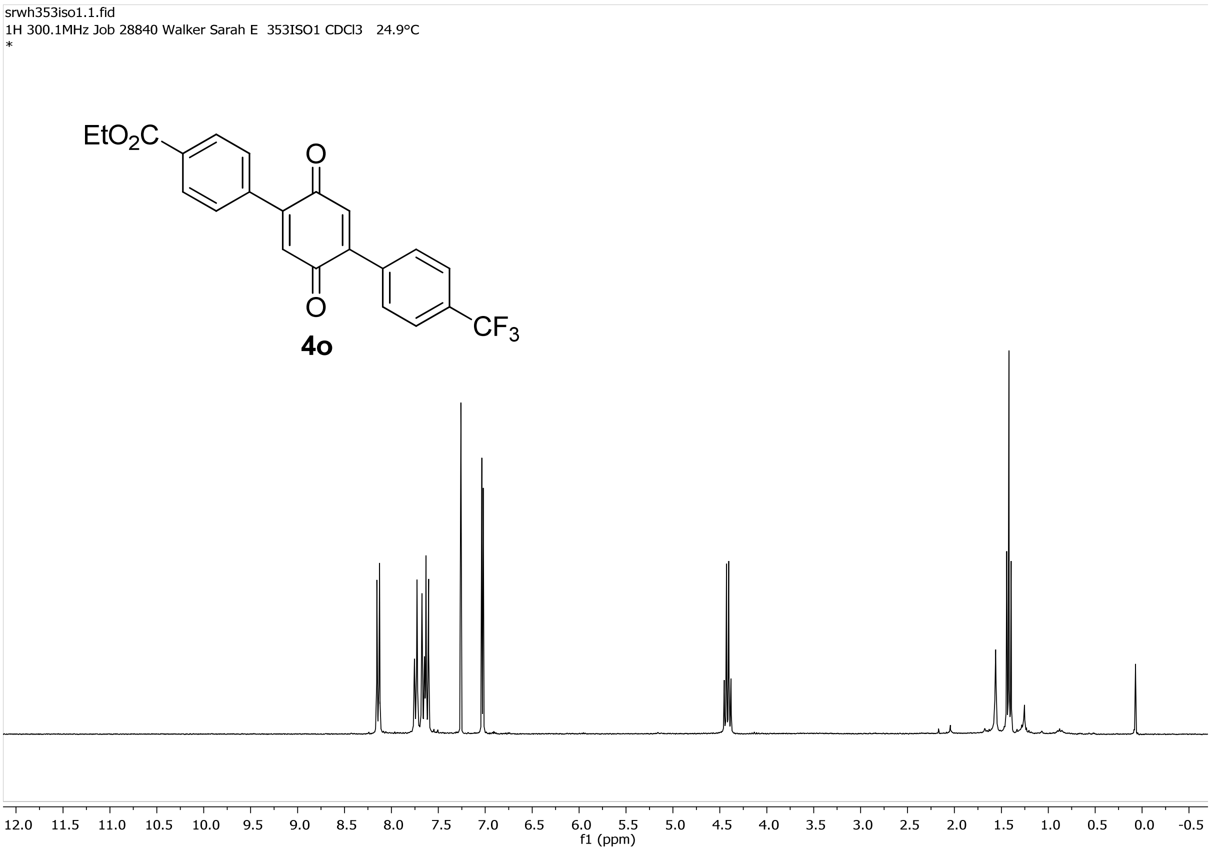
srwh353iso1.1.fid
1H 300.1MHz Job 28840 Walker Sarah E 353ISO1 CDCl3 24.9°C
*
EtO2C
O
O
CF3
4o
12.0 11.5 11.0 10.5 10.0 9.5 9.0 8.5 8.0 7.5 7.0 6.5 6.0 5.5 5.0 4.5 4.0 3.5 3.0 2.5 2.0 1.5 1.0 0.5 0.0 -0.5
f1 (ppm)

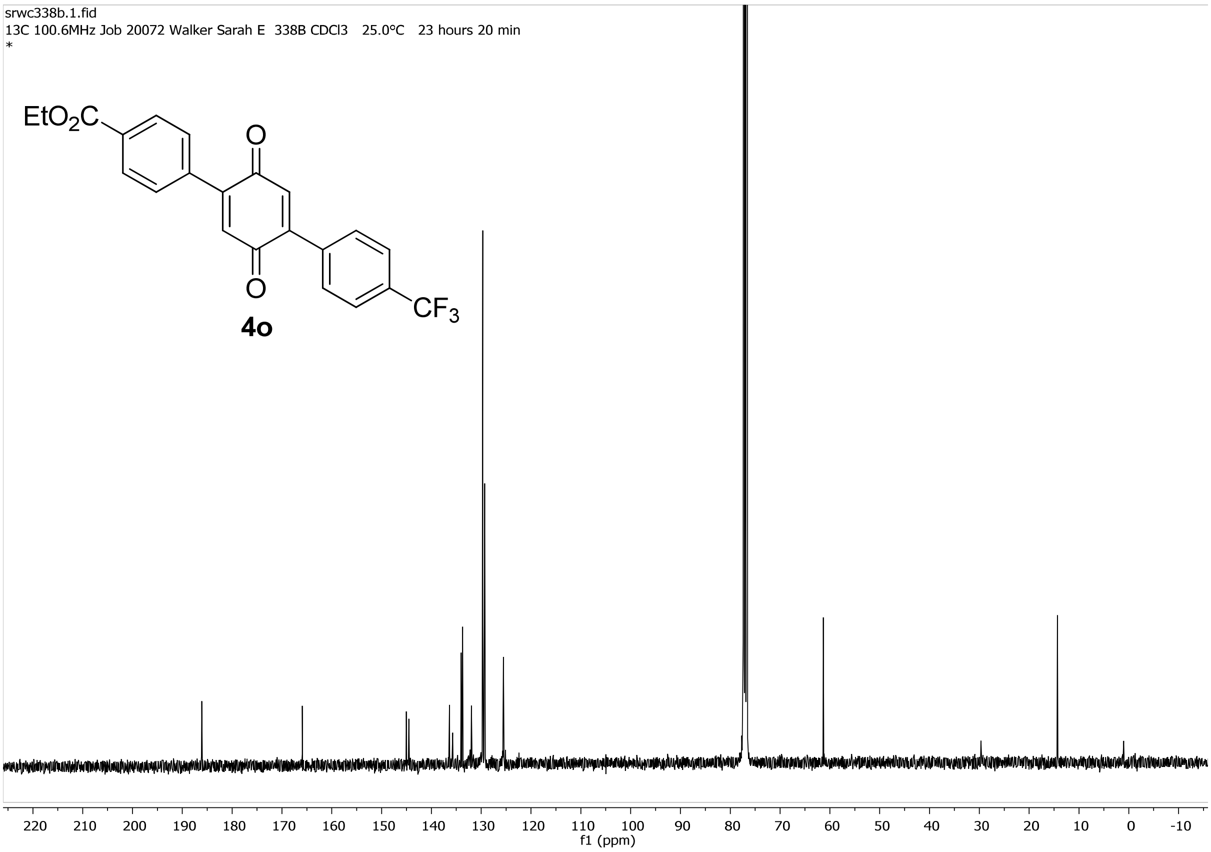
srwc338b.1.fid
13C 100.6MHz Job 20072 Walker Sarah E 338B CDCl3 25.0°C 23 hours 20 min
*
EtO2C
O
O
CF3
4o
220 210 200 190 180 170 160 150 140 130 120 110 100 90 80 70 60 50 40 30 20 10 0 -10
f1 (ppm)

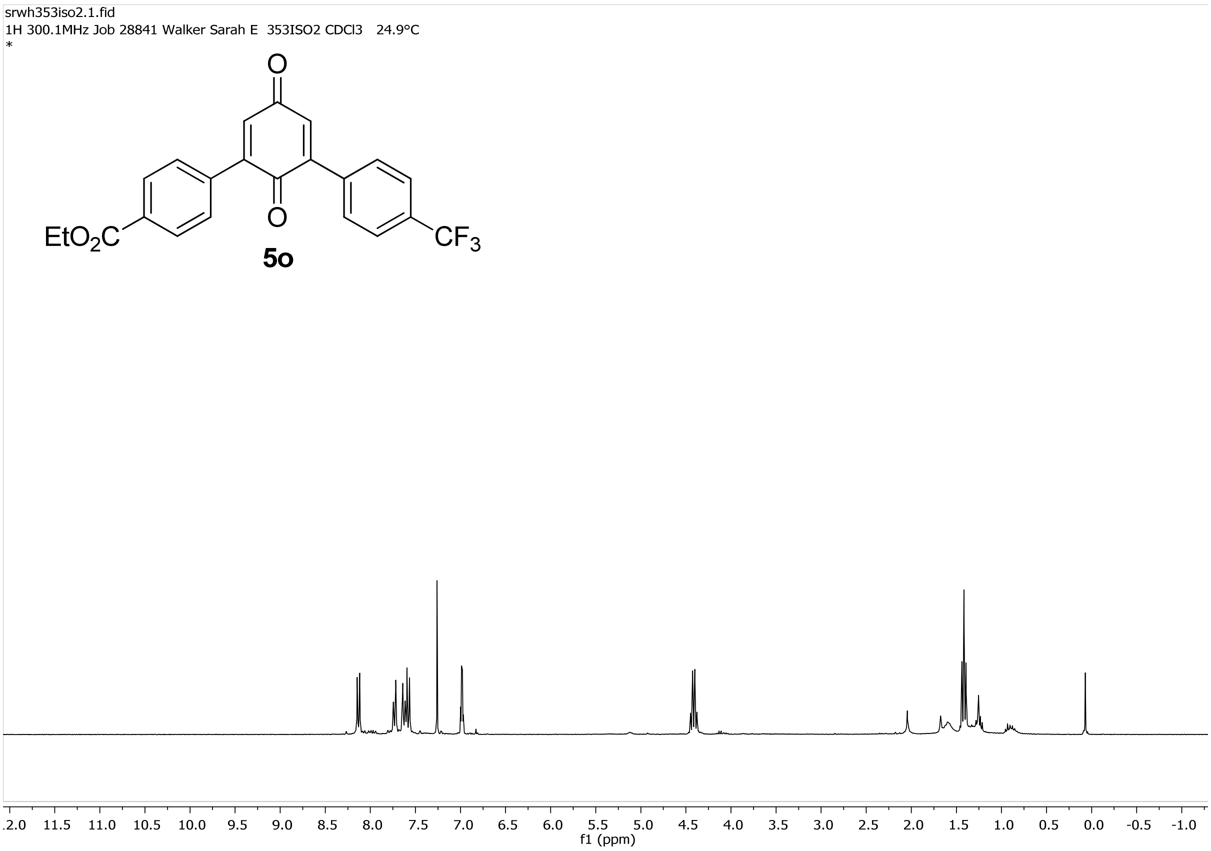
srwh353iso2.1.fid
1H 300.1MHz Job 28841 Walker Sarah E 353ISO2 CDCl3 24.9°C
EtO2C
CF3
5o
f1 (ppm)

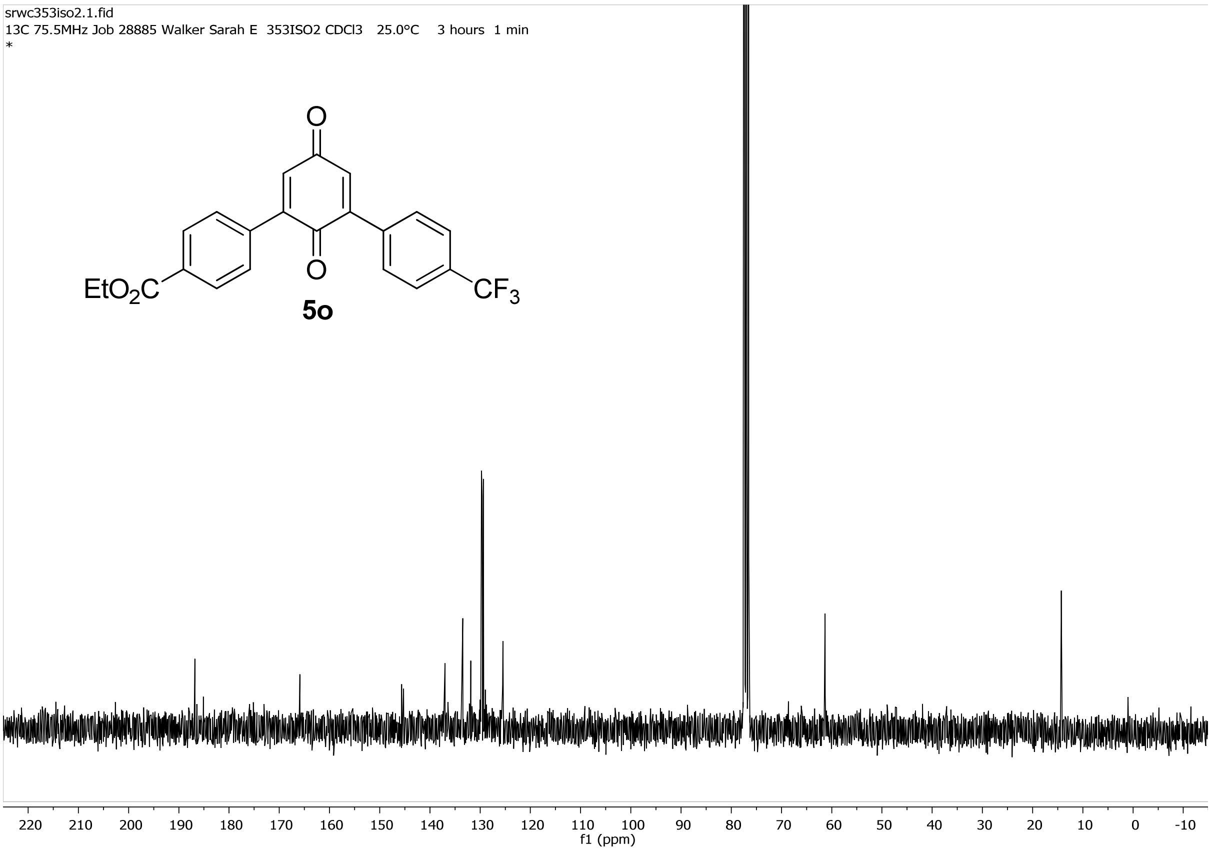
srwc353iso2.1.fid
13C 75.5MHz Job 28885 Walker Sarah E 353ISO2 CDCl3 25.0°C 3 hours 1 min
EtO2C
CF3
5o
f1 (ppm)

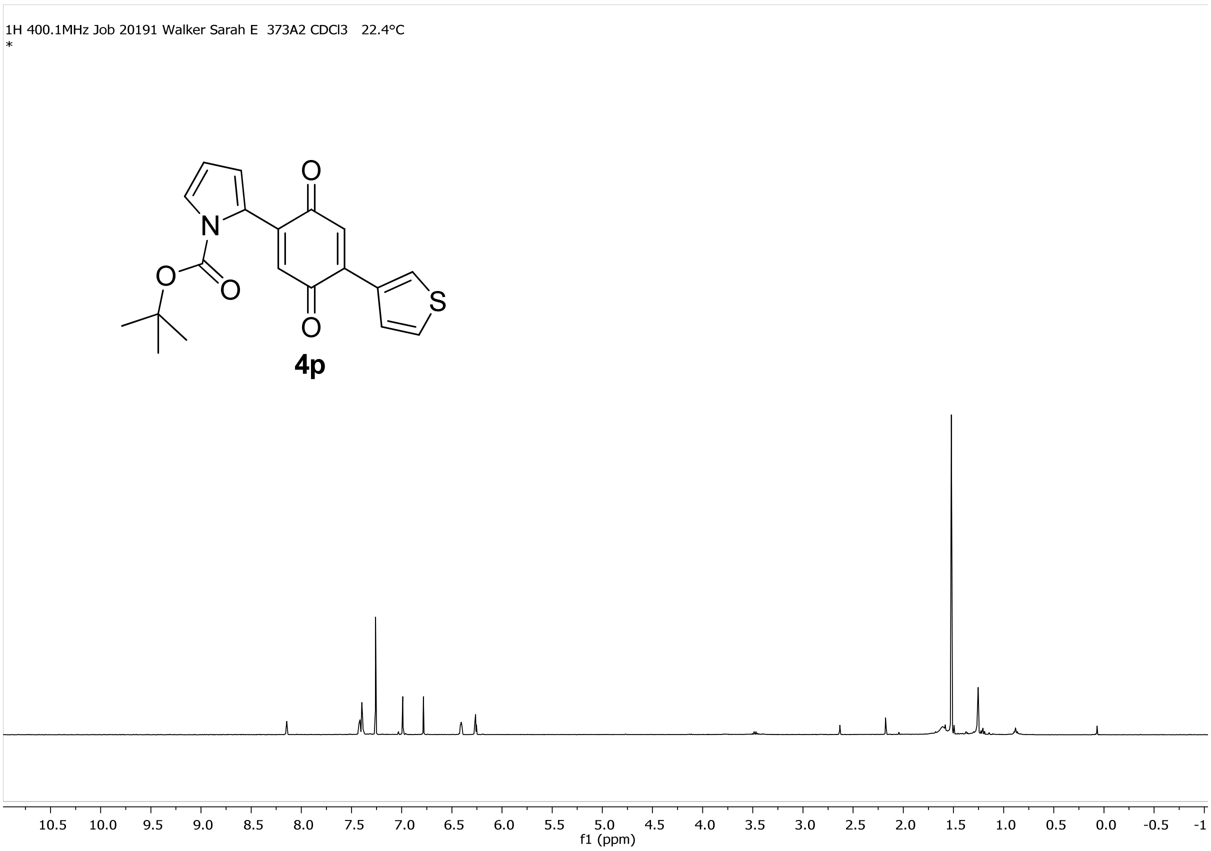
1H 400.1MHz Job 20191 Walker Sarah E 373A2 CDCl3 22.4°C
*
4p
10.5 10.0 9.5 9.0 8.5 8.0 7.5 7.0 6.5 6.0 5.5 5.0 4.5 4.0 3.5 3.0 2.5 2.0 1.5 1.0 0.5 0.0 -0.5 -1
f1 (ppm)

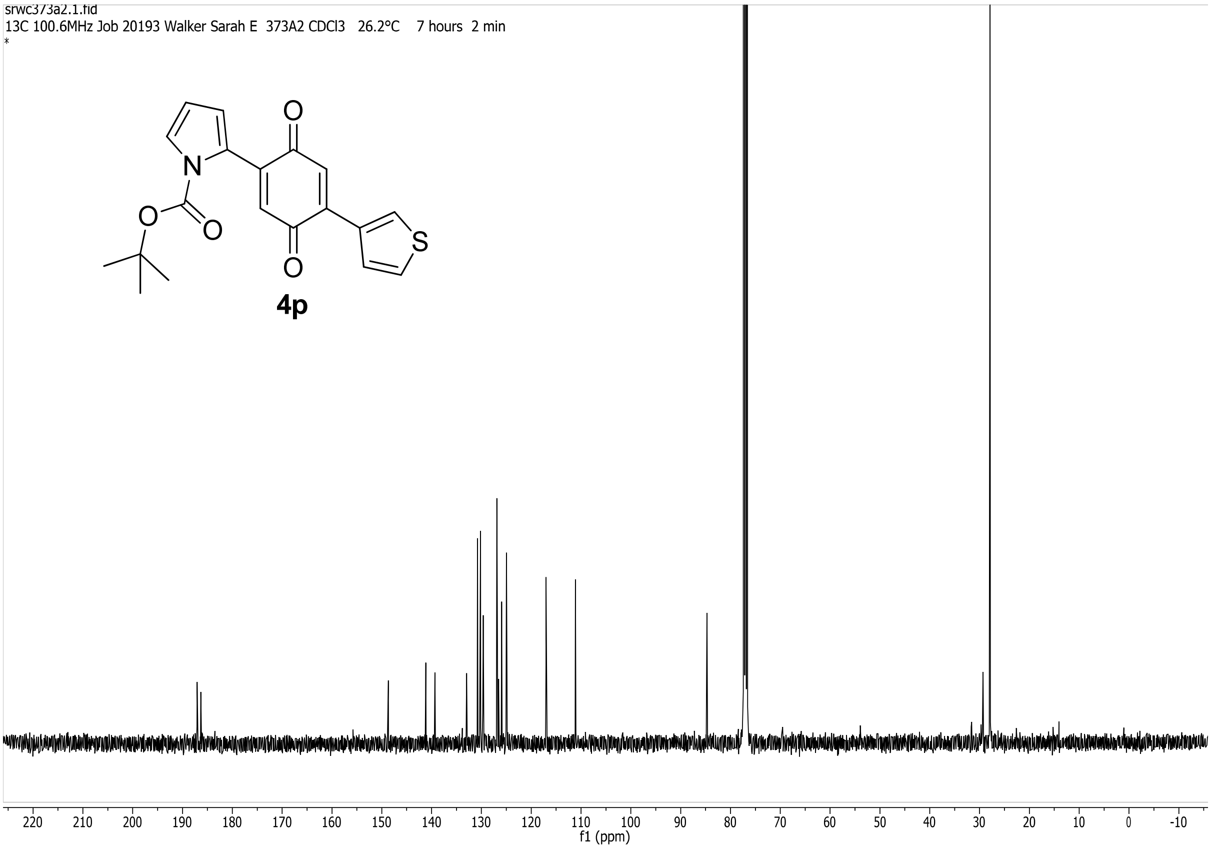
srwc373a2.1.fid
13C 100.6MHz Job 20193 Walker Sarah E 373A2 CDCl3 26.2°C 7 hours 2 min
*
4p
220 210 200 190 180 170 160 150 140 130 120 110 100 90 80 70 60 50 40 30 20 10 0 -10
f1 (ppm)

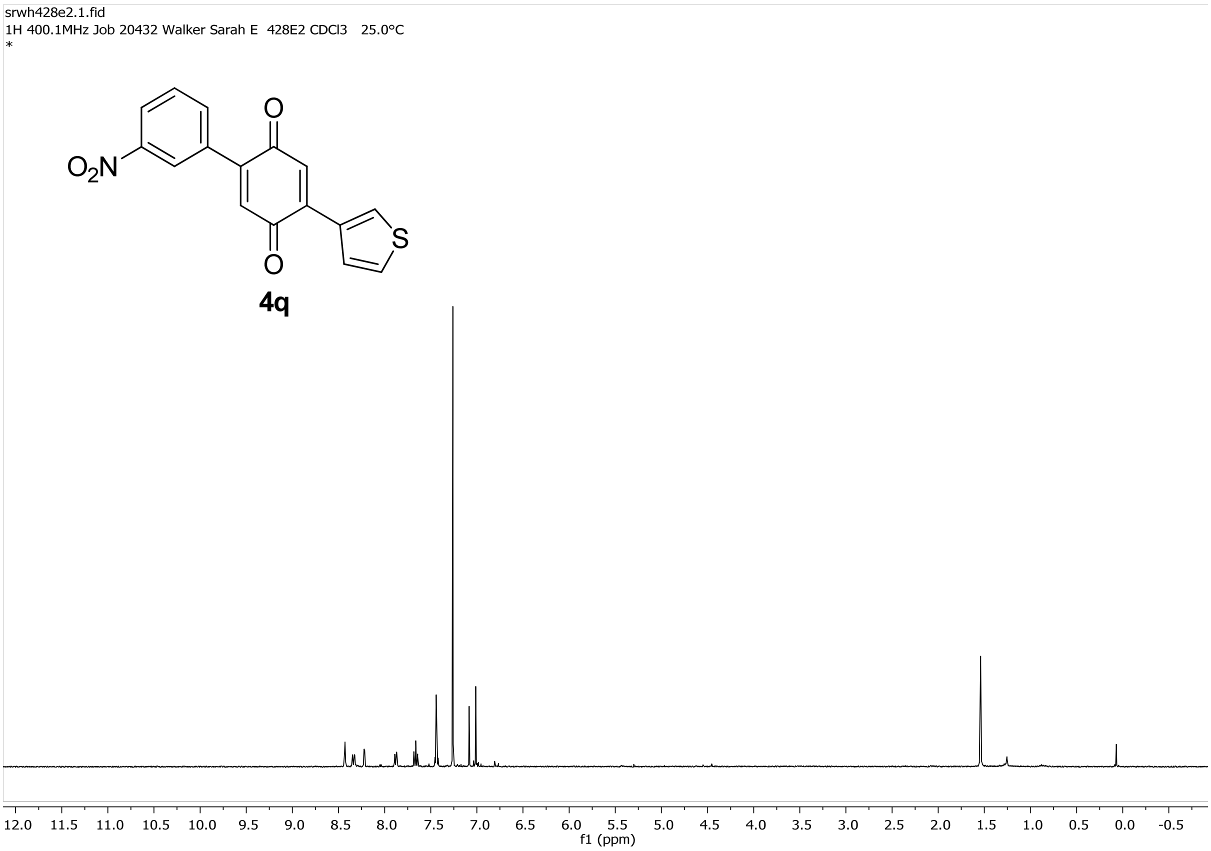
srwh428e2.1.fid
1H 400.1MHz Job 20432 Walker Sarah E 428E2 CDCl3 25.0°C
O2N
O
O
S
4q
12.0 11.5 11.0 10.5 10.0 9.5 9.0 8.5 8.0 7.5 7.0 6.5 6.0 5.5 5.0 4.5 4.0 3.5 3.0 2.5 2.0 1.5 1.0 0.5 0.0 -0.5
f1 (ppm)

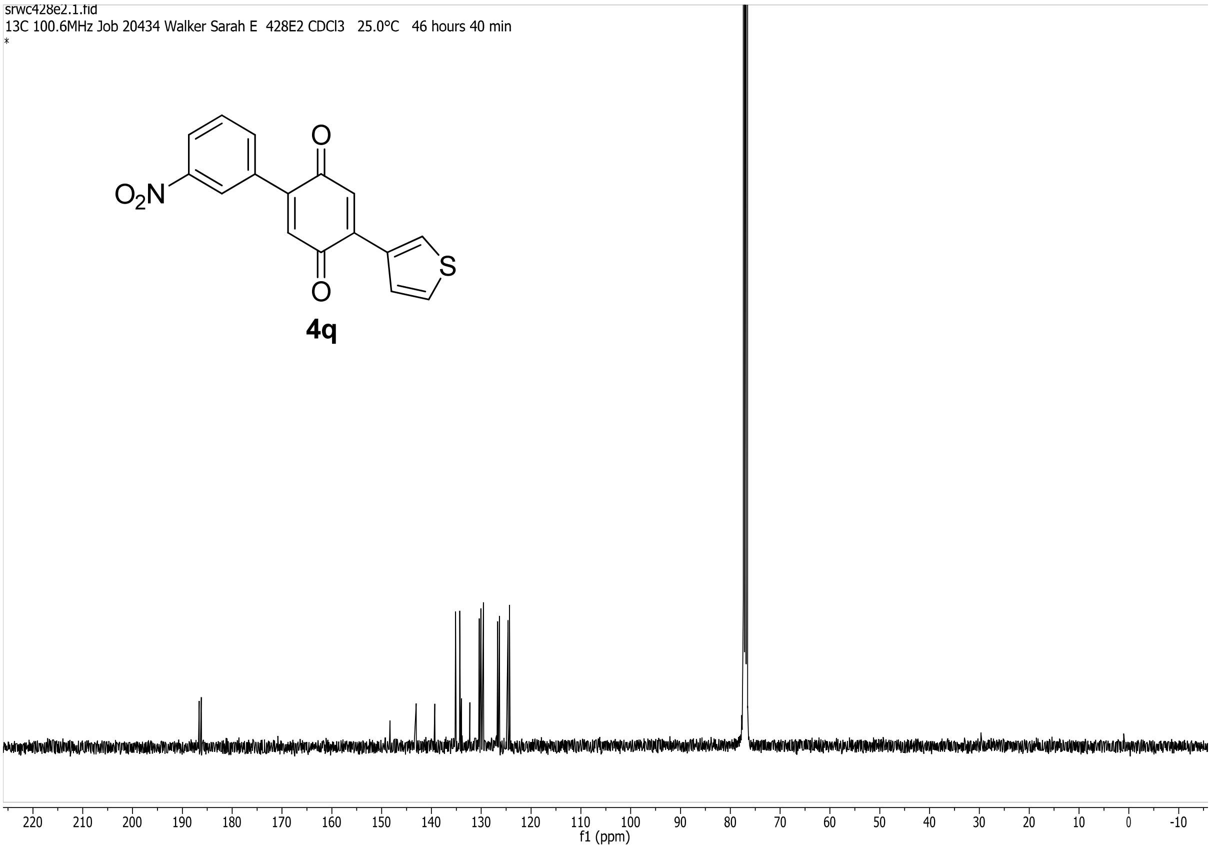
srwc428e2.1.fid
13C 100.6MHz Job 20434 Walker Sarah E 428E2 CDCl3 25.0°C 46 hours 40 min
O2N
O
O
S
4q
220 210 200 190 180 170 160 150 140 130 120 110 100 90 80 70 60 50 40 30 20 10 0 -10
f1 (ppm)

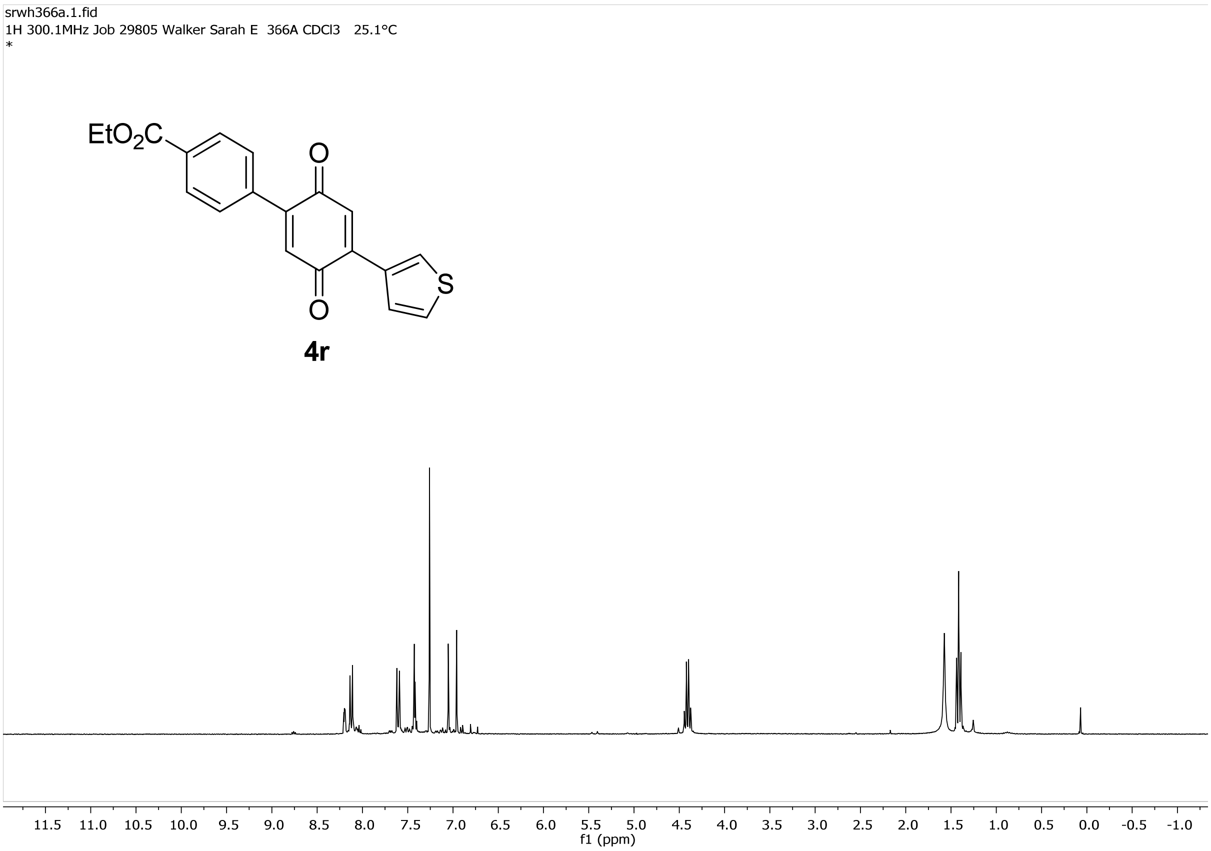
srwh366a.1.fid
1H 300.1MHz Job 29805 Walker Sarah E 366A CDCl3 25.1°C
*
EtO2C
O
S
O
4r
11.5 11.0 10.5 10.0 9.5 9.0 8.5 8.0 7.5 7.0 6.5 6.0 5.5 5.0 4.5 4.0 3.5 3.0 2.5 2.0 1.5 1.0 0.5 0.0 -0.5 -1.0
f1 (ppm)

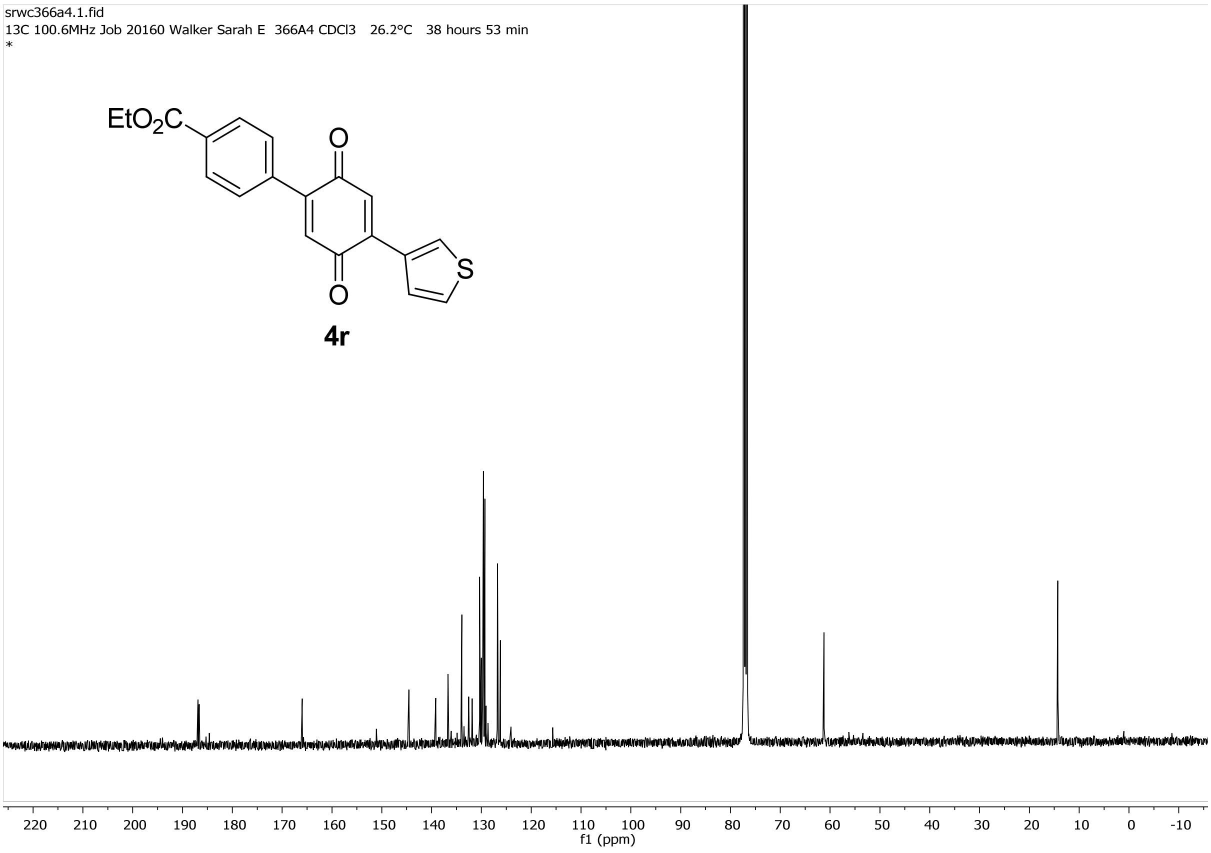
srwc366a4.1.fid
13C 100.6MHz Job 20160 Walker Sarah E 366A4 CDCl3 26.2°C 38 hours 53 min
*
EtO2C
O
S
O
4r
220 210 200 190 180 170 160 150 140 130 120 110 100 90 80 70 60 50 40 30 20 10 0 -10
f1 (ppm)

srwh309a.1.fid
1H 400.1MHz Job 20047 Walker Sarah E 309A CDCl3 25.0°C
*

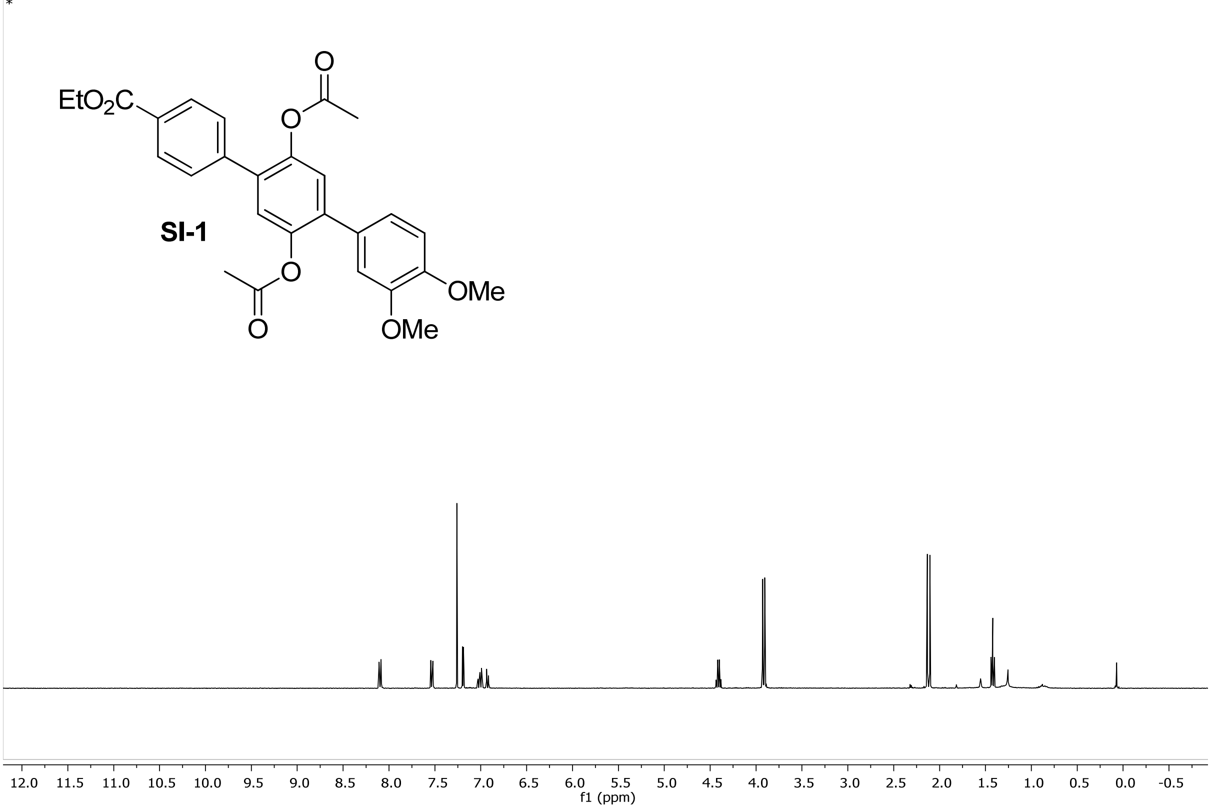

srwc309a.1.fid
13C 100.6MHz Job 20048 Walker Sarah E 309A CDCl3 25.0°C 52 hours 0 min
*

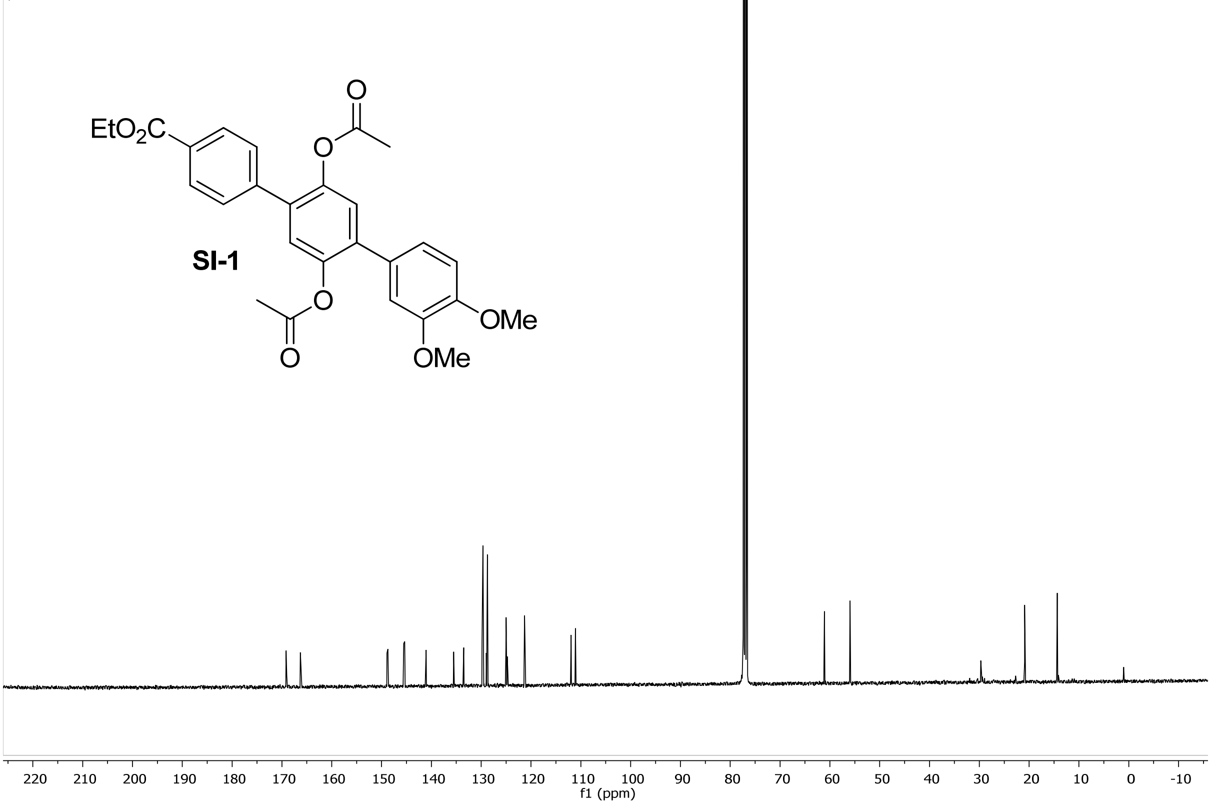

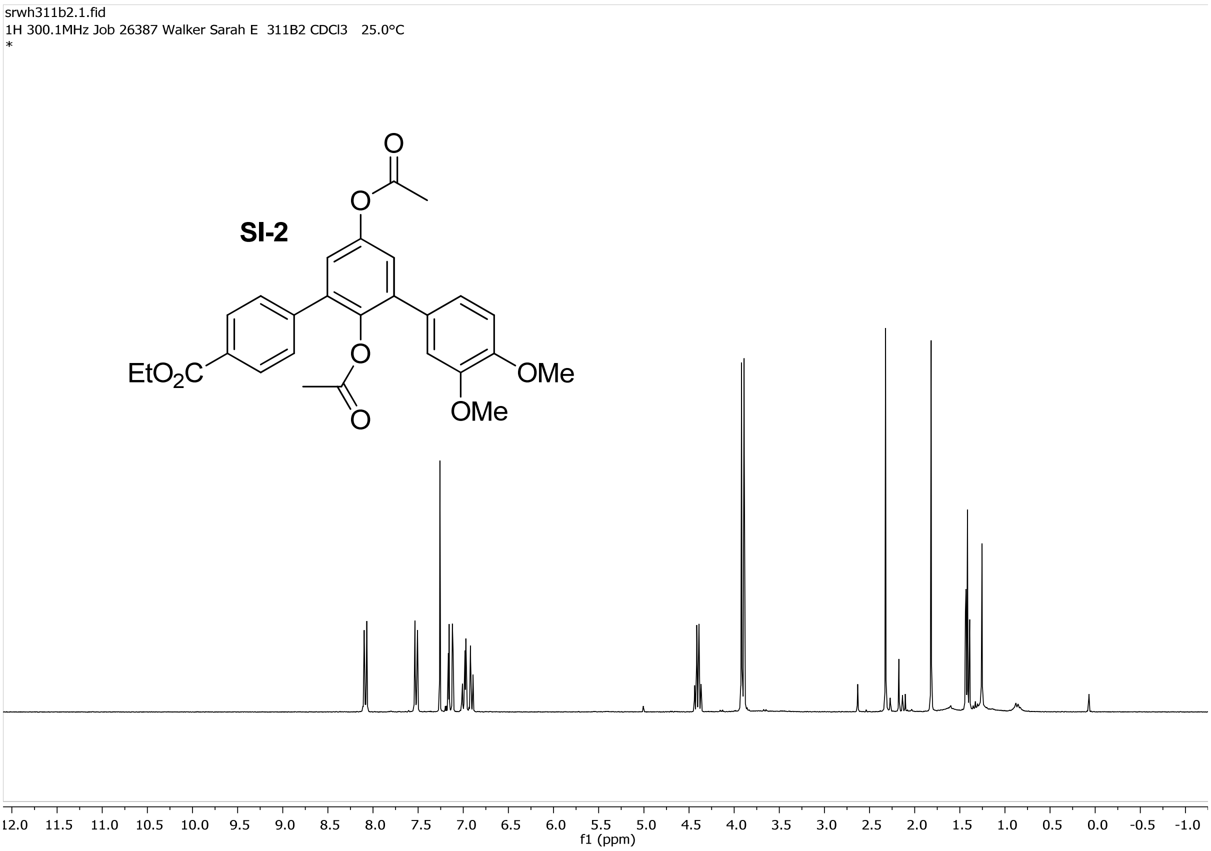
srwh311b2.1.fid
1H 300.1MHz Job 26387 Walker Sarah E 311B2 CDCl3 25.0°C
*
SI-2
EtO2C
OMe
OMe
O
12.0 11.5 11.0 10.5 10.0 9.5 9.0 8.5 8.0 7.5 7.0 6.5 6.0 5.5 5.0 4.5 4.0 3.5 3.0 2.5 2.0 1.5 1.0 0.5 0.0 -0.5 -1.0
f1 (ppm)

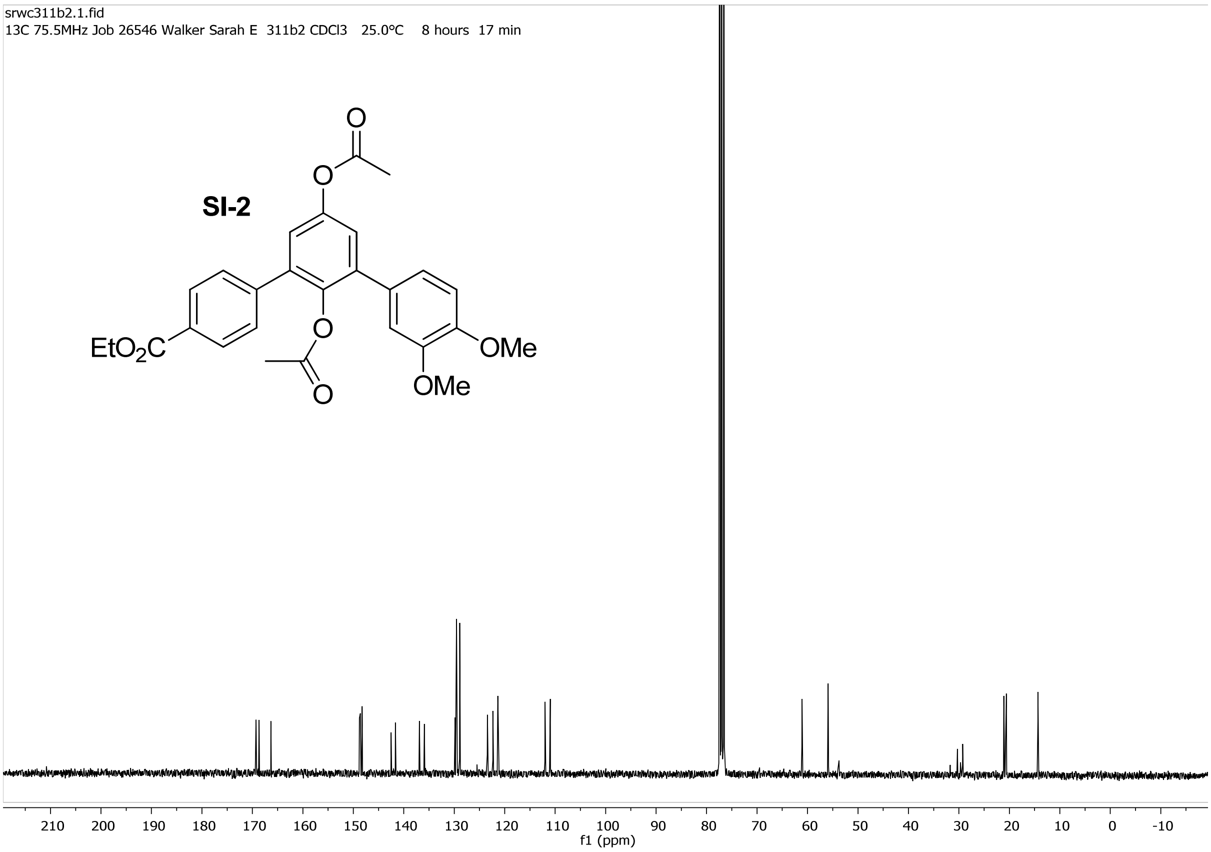
srwc311b2.1.fid
13C 75.5MHz Job 26546 Walker Sarah E 311b2 CDCl3 25.0°C 8 hours 17 min
SI-2
EtO2C
OMe
OMe
O
210 200 190 180 170 160 150 140 130 120 110 100 90 80 70 60 50 40 30 20 10 0 -10
f1 (ppm)

13. References

[1] D. P. Bancroft, F. A.Cotton, M. Verbruggen, *Acta Cryst.* **1989**, *C45*, 1289.

[2] Y. Fujiwara, V. Domingo, I. B. Seiple, R. Gianatassio, M. D. Bel, P. S. Baran, *J. Am. Chem. Soc* **2011**, *133*, 3292-3295.

[3] P. Brassard, P. L'Ecuyer, *Can. J.Chem.* **1958**, *36*, 700-708.

[4] M. Lamblin, N. G., J. Dessolin, F.-X. Felpin, *Synlett* **2012**, *23*, 1621-1624.

[5] J. Wang, S. Wang, G. Wang, J. Zhang, X.-Q. Yu, *Chem. Commun.* **2012**, *48*, 11769-11771.

[6] K. Komeyama, T. Kashihara, K. Takaki, *Tet. Lett.* **2013**, *54*, 1084-1086.

[7] R. Buchan, O. C. Musgrave, *J. Chem. Soc., Perkin Trans. 1* **1975**, *6*, 568-572.

[8] M. L. N. Rao, S. Giri, *RSC Adv.* **2012**, *2*, 12739–12750.

[9] K. Maruyama, T. Shio, Y. Yamamoto, *Bull. Chem. Soc. Jpn.* **1979**, *52*, 1877.

[10] T. W. Lyons, K. L. Hull, M. S. Sanford, *J. Am. Chem. Soc* **2011**, *133*, 4455-4464.

[11] A. Ortega, A. Rincón, K. L. Jiménez-Aliaga, P. Bermejo-Bescós, A. Martín-Aragón, M. T. Molina, A. G. Csákÿ, *Bioorg.Med.Chem. Lett.* **2011**, *21*, 2183-2187.

Computational Supporting Information

DFT calculations probing the migratory insertion step suggest the factors controlling selectivity are subtle. Representative results with a $[Pd(Ar)(\mathbf{3})(acetone)_2]^+$ model system showed that electron donating substituents, R (the BQ substituent, Table 3 in the main text) = Ar = *p*-C_6H_4-OMe, kinetically favor migratory insertion to the 2,6-product, but only by 0.5 kcal/mol; when R = Ar = *p*-C_6H_4-CF_3 the two transition states have the same energy. Similar trends were obtained with other model systems featuring $CF_3CO_2^-$ and acetone co-ligands. The small energy differences are consistent with the similar charge distribution and LUMO coefficients of these two BQ substrates. Full details are given below and future work will consider the overall mechanism of this direct C-H functionalization of BQ, and the factors controlling selectivity.

1. Computational Details and References.

Calculations were run with Gaussian 03 Revision D.01[1] with PCM solvent corrections run with Gaussian 09, Revision A.02.[2] Geometry optimisations were performed using the BP86 functional[3] with Pd described with the Stuttgart RECPs and associated basis set[4] and 6-31G** basis sets for all other atoms.[5] All stationary points were fully characterized via analytical frequency calculations as either minima (all positive eigenvalues) or transition states (one negative eigenvalue) and IRC calculations and subsequent geometry optimizations were used to confirm the minima linked by each transition state. Frequency calculations also provided a free energy in the gas-phase, computed at 298.15 K and 1 atm. Correction for dispersion effects using Grimme's D3 parameter set[6] (i.e. BP86-D3) as well as solvation in acetone (PCM approach) were applied. NBO charge distributions were calculated within Gaussian 09 using NBO 5.9.[7]

1. Gaussian 03, Revision C.02, M. J. Frisch, G. W. Trucks, H. B. Schlegel, G. E. Scuseria, M. A. Robb, J. R. Cheeseman, J. A. Montgomery, Jr., T. Vreven, K. N. Kudin, J. C. Burant, J. M. Millam, S. S. Iyengar, J. Tomasi, V. Barone, B. Mennucci, M. Cossi, G. Scalmani, N. Rega, G. A. Petersson, H. Nakatsuji, M. Hada, M. Ehara, K. Toyota, R. Fukuda, J. Hasegawa, M. Ishida, T. Nakajima, Y. Honda, O. Kitao, H. Nakai, M. Klene, X. Li, J. E. Knox, H. P. Hratchian, J. B. Cross, C. Adamo, J. Jaramillo, R. Gomperts, R. E. Stratmann, O. Yazyev, A. J. Austin, R. Cammi, C. Pomelli, J. W. Ochterski, P. Y. Ayala, K. Morokuma, G. A. Voth, P. Salvador, J. J. Dannenberg, V. G. Zakrzewski, S. Dapprich, A. D. Daniels, M. C. Strain, O. Farkas, D. K. Malick, A. D. Rabuck, K. Raghavachari, J. B. Foresman, J. V. Ortiz, Q. Cui, A. G. Baboul, S. Clifford, J. Cioslowski, B. B. Stefanov, G. Liu, A. Liashenko, P. Piskorz, I. Komaromi, R. L. Martin, D. J. Fox, T. Keith, M. A. Al-Laham, C. Y. Peng, A. Nanayakkara, M. Challacombe, P. M. W. Gill, B. Johnson, W. Chen, M. W. Wong, C. Gonzalez, and J. A. Pople, Gaussian, Inc., Wallingford CT, 2004.
2. Gaussian 09, Revision A.02, M. J. Frisch, G. W. Trucks, H. B. Schlegel, G. E. Scuseria, M. A. Robb, J. R. Cheeseman, G. Scalmani, V. Barone, B. Mennucci, G. A. Petersson, H. Nakatsuji, M. Caricato, X. Li, H. P. Hratchian, A. F. Izmaylov, J. Bloino, G. Zheng, J. L. Sonnenberg, M. Hada, M. Ehara, K. Toyota, R. Fukuda, J. Hasegawa, M. Ishida, T. Nakajima, Y. Honda, O. Kitao, H. Nakai, T. Vreven, J. A. Montgomery, Jr., J. E. Peralta, F. Ogliaro, M. Bearpark, J. J. Heyd, E. Brothers, K. N. Kudin, V. N. Staroverov, R. Kobayashi, J. Normand, K. Raghavachari, A. Rendell, J. C. Burant, S. S. Iyengar, J. Tomasi, M. Cossi, N. Rega, J. M. Millam, M. Klene, J. E. Knox, J. B. Cross, V. Bakken, C. Adamo, J. Jaramillo, R. Gomperts, R. E. Stratmann, O. Yazyev, A. J. Austin, R. Cammi, C. Pomelli, J. W. Ochterski, R. L. Martin, K. Morokuma, V. G. Zakrzewski, G. A. Voth, P. Salvador, J. J. Dannenberg, S. Dapprich, A. D. Daniels, O. Farkas, J. B. Foresman, J. V. Ortiz, J. Cioslowski, and D. J. Fox, Gaussian, Inc., Wallingford CT, 2009.
3. (a) A. D. Becke, *Phys. Rev. A* **1988**, *38*, 3098. (b) J. P. Perdew, *Physical Review B* **1986**, *33*, 8822.
4. D. Andrae, U. Häußermann, M. Dolg, H. Stoll and H. Preuß, *Theor. Chim. Acta* **1990**, *77*, 123.
5. (a) W. J. Hehre, R. Ditchfield and J. A. Pople, *J. Chem. Phys.* **1972**, *56*, 2257. (b) P. C. Hariharan and J. A. Pople, *Theor. Chim. Acta.* **1973**, *28*, 213.
6. S. Grimme, J. Antony, S. Ehrlich and H. Krieg, *J. Chem. Phys.* **2010**, *132*, 154104.
7. NBO 5.9. E. D. Glendening, J. K. Badenhoop, A. E. Reed, J. E. Carpenter, J. A. Bohmann, C. M. Morales, and F. Weinhold (Theoretical Chemistry Institute, University of Wisconsin, Madison, WI, 2009); http://www.chem.wisc.edu/~nbo5

2. Computed Charge Distributions in 3 R = *p*-$C_6H_4CF_3$ and *p*-C_6H_4OMe

NBO atomic charges were computed for substrates **3** with R = *p*-$C_6H_4CF_3$ and *p*-C_6H_4OMe with a focus on the C6 and C5 positions. In both cases C5 bears a slightly lower negative and no significant change in charge distribution is seen when comparing the two substrates. Thus the change in regioselectivity in moving from combinations of electron donating to electron withdrawing substituents cannot be simply rationalised by changes in the charge distribution.

Table S1. Selected Computed NBO Charges on the C5 and C6 positions of substrates 3 (R = *p*-$C_6H_4CF_3$ and *p*-C_6H_4OMe)

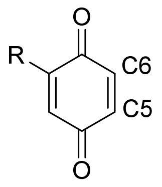

R	*p*-$C_6H_4CF_3$	*p*-C_6H_4OMe
C6	-0.262	-0.268
C5	-0.259	-0.247

3. Orbital Distributions in the LUMOs of 3 (R = *p*-$C_6H_4CF_3$ and *p*-C_6H_4OMe)

Orbital distributions were computed for the LUMO of substrates **3** with R = *p*-$C_6H_4CF_3$ and *p*-C_6H_4OMe. As shown in Figure S1 only a minor asymmetry in $2p_z$ contribution is computed with both substituents, with the larger contribution being at C5 in both cases. The LUMO+1 was also considered in each case, although this is significantly higher in energy. A larger asymmetry is seen here, although the greater contribution is associated with C5, suggesting that changes in regioselectivity as a function of the electronic character of the substituent cannot be attributed to orbital overlap arguments.

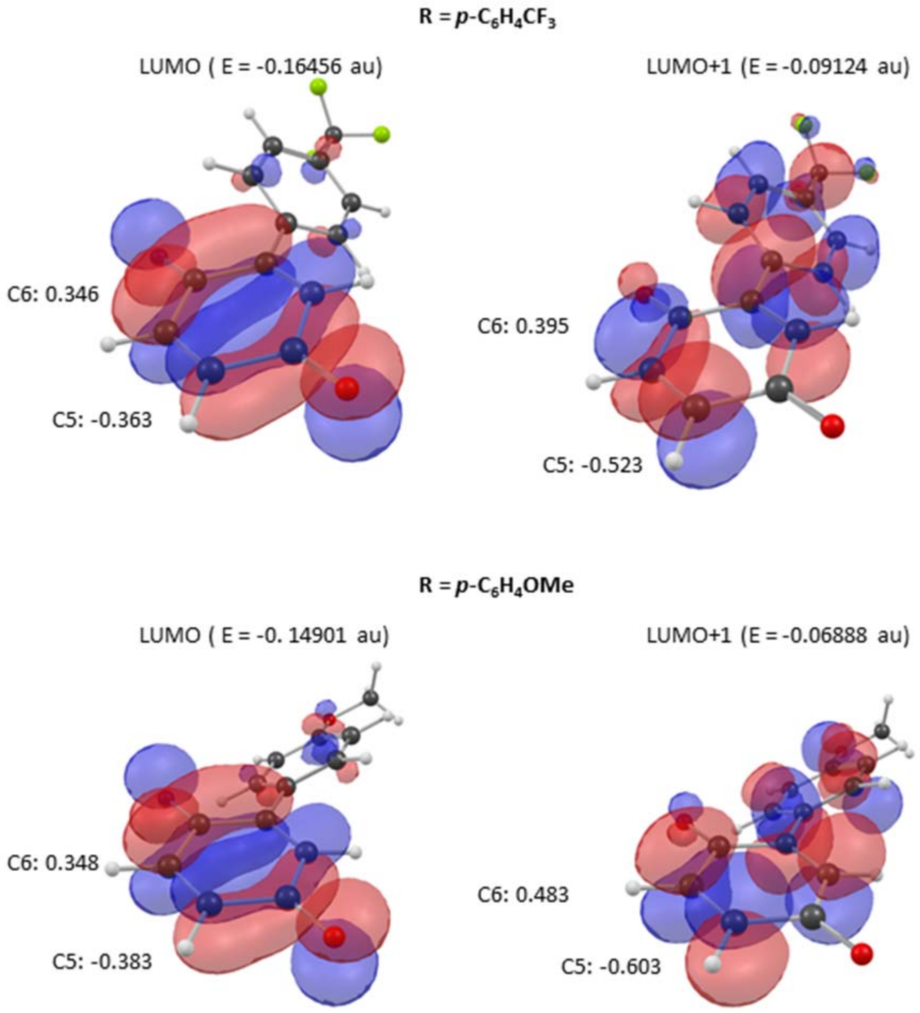

Figure S1. Orbital Distributions in the LUMOs and LUMOs+1 of **3** (R = *p*-$C_6H_4CF_3$ and *p*-C_6H_4OMe). The total $2p_z$ orbital contributions at the C6 and C5 positions are indicated in each case and the orbital energy is given in atomic units**.**

4. Mechanistic Studies.

4.1 Product Stability

Computed energies are reported as ΔE (uncorrected SCF), ΔH_{298} (enthalpy at 298.15 K), ΔG (free energy at 298.15 K), ΔG-D3 (free energy corrected for dispersion using Grimme's D3 parameter set) and ΔG-D3 (acetone) (as for ΔG-D3 now corrected for acetone solvent via the PCM approach).

Table S2. Computed Product Energies

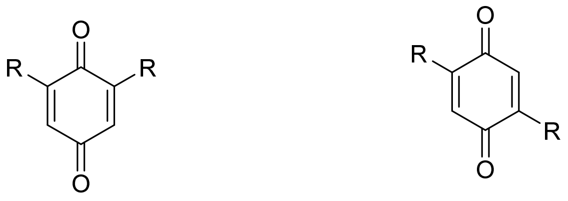

2,6-disubstituted product (5)　　**2,5-disubstituted product (4)**

R = *p*-$C_6H_4CF_3$	ΔE	ΔH_{298}	ΔG	ΔG-D3	ΔG-D3(acetone)
**5 (2,6)**	+0.0	+0.0	+0.0	+0.0	+0.0
**4 (2,5)**	-1.2	-1.2	-1.1	-0.9	-0.5
**R = *p*-C_6H_4OMe**					
**5 (2,6)**	+0.0	+0.0	+0.0	+0.0	+0.0
**4 (2.5)**	-1.8	-1.7	-1.8	-1.5	-1.0

The similar energies of the alternative 2,6- and 2,5-disubstituted benzoquinoline products suggests that any change in regioselectivity in moving from combinations of electron donating to electron withdrawing substituents cannot be simply rationalised by product stability.

4.2 Computed reaction profiles for migratory insertion.

Reaction profiles were computed for the insertion of substrates **3** (with R = *p*-$C_6H_4CF_3$ and *p*-C_6H_4OMe) into the Pd-R' metal-aryl bond (where R'= *p*-$C_6H_4CF_3$ and *p*-C_6H_4OMe). Only symmetrical combinations of R and R' were considered, modelling the homo-difunctionalisation reactions. As the nature of the active species is unknown several model systems were assessed, including anionic $[PdR'(CF_3CO_2)_2(\mathbf{3})]^-$, neutral $[PdR'(CF_3CO_2)(\mathbf{3})]$ and $[PdR'(CF_3CO_2)(acetone)(\mathbf{3})]$ as well as a cationic model, $[PdR'(acetone)_2(\mathbf{3})]^+$. For each system several different orientations of both the BQ moiety and the supporting ligands where considered, and the lowest lying stationary points are reported below. Energies are quoted relative to the lowest energy form of the precursor complex and are reported as ΔE (uncorrected SCF), ΔH_{298} (enthalpy at 298.15 K), ΔG (free energy at 298.15 K), ΔG-D3 (free energy corrected for dispersion using Grimme's D3 parameter set) and ΔG-D3 (acetone) (as for ΔG-D3 now corrected for acetone solvent via the PCM approach).

Table S3 summarises the computed barriers for migratory insertion at the four model systems considered. Based on the results at the ΔG-D3(acetone) level, a small kinetic preference for the formation of the 2,6-regioisomer is computed for all four model systems when R = *p*-C_6H_4OMe (between 0.3 kcal/mol and 1.4 kcal/mol). When R = *p*-$C_6H_4CF_3$ the preference for the 2,6-regioisomer is either reduced ($[PdR'(CF_3CO_2)_2(\mathbf{3})]^-$, $\Delta\Delta G^\ddagger$ = +0.9 kcal/mol; $[PdR'(CF_3CO_2)(acetone)(\mathbf{3})]$ with $CF_3CO_2^-$ trans to **3**: $\Delta\Delta G^\ddagger$ = +0.1 kcal/mol; $[PdR'(acetone)_2(\mathbf{3})]^+$, $\Delta\Delta G^\ddagger$ = +0.0 kcal/mol) or the 2,5-regioisomer becomes preferred ($[PdR'(CF_3CO_2)(\mathbf{3})]$, $\Delta\Delta G^\ddagger$ = -0.7 kcal/mol, $[PdR'(CF_3CO_2)(acetone)(\mathbf{3})]$ with $CF_3CO_2^-$ trans to **3**: $\Delta\Delta G^\ddagger$ = -0.1 kcal/mol). Thus the trend os for the 2,5-regioisomer to become *relatively* more accessible upon moving from the electron donating *p*-C_6H_4OMe substituent to the electron withdrawing *p*-$C_6H_4CF_3$.

Table S3. Summary of computed activation barriers for migratory insertion at the four model systems considered.

**Model System**	**R = R' = *p*-C_6H_4OMe**		**R = R' = *p*-$C_6H_4CF_3$**	
	**$\Delta G^\ddagger$(2,6)**	**$\Delta G^\ddagger$(2,5)**	**$\Delta G^\ddagger$(2,6)**	**$\Delta G^\ddagger$(2,5)**
$[PdR'(CF_3CO_2)_2(\mathbf{3})]^-$	11.5	12.9	15.1	16.0
$[PdR'(CF_3CO_2)(\mathbf{3})]$	13.9	14.6	19.3	18.6
$[PdR'(CF_3CO_2)(acetone)(\mathbf{3})]$[a]	13.0	13.7	19.3	19.4
$[PdR'(CF_3CO_2)(acetone)(\mathbf{3})]$[b]	14.2	14.5	18.4	18.3
$[PdR'(acetone)_2(\mathbf{3})]^+$.	9.6	10.1	14.5	14.5

[a] $CF_3CO_2^-$ trans to **3;** [b] acetone trans to **3**

4.2.1 [PdR’(CF_3CO_2)(3)]

Table S4. Computed energetics for insertion at [PdR’(CF_3CO_2)(**3**)] (R = R' = *p*-$C_6H_4CF_3$; R = R' = *p*-C_6H_4OMe).

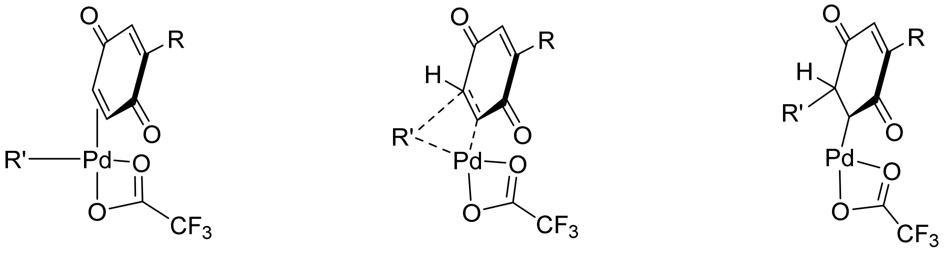

Int 1 **TS(1-2)** **Int 2**

R = R' = *p*-$C_6H_4CF_3$	ΔE	$ΔH_{298}$	ΔG	ΔG-D3	ΔG-D3(acetone)
**Int 1**	+0.0	+0.0	+0.0	+0.0	+0.0
**TS_{1-2} (2,6)**	+14.5	+13.8	+15.0	+17.3	+15.1
**TS_{1-2} (2,5)**	+14.2	+13.5	+14.3	+17.9	+16.0
**Int 2 (2,6)**	-7.3	-6.7	-6.0	-2.7	-5.2
**Int 2 (2.5)**	-8.1	-7.5	-6.8	-3.8	-6.0
**R = R' = *p*-C_6H_4OMe**					
**Int 1**	+0.0	+0.0	+0.0	+0.0	+0.0
**TS_{1-2} (2,6)**	+14.6	+13.8	+16.3	+13.2	+11.5
**TS_{1-2} (2,5)**	+14.1	+13.3	+15.5	+13.7	+12.9
**Int 2 (2,6)**	-8.8	-8.2	-6.0	-7.9	-9.7
**Int 2 (2.5)**	-9.0	-8.4	-6.4	-8.6	-10.3

4.2.2 [PdR′(CF_3CO_2)$_2$(3)]$^-$

Table S5. Computed energetics for insertion at [PdR′(CF_3CO_2)$_2$(**3**)]$^-$ (R = R' = *p*-$C_6H_4CF_3$; R = R' = *p*-C_6H_4OMe).

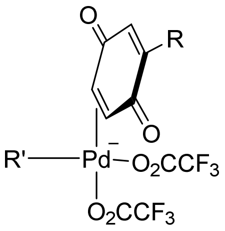

Int 1

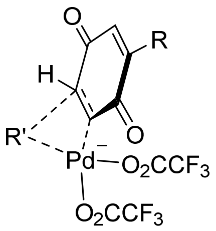

TS(1-2)

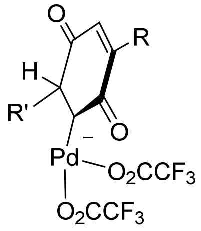

Int 2

R = R' = *p*-$C_6H_4CF_3$	ΔE	$ΔH_{298}$	ΔG	ΔG-D3	ΔG-D3(acetone)
**Int 1**[a]	+0.0	+0.0	+0.0	+0.0	+0.0
**TS_{1-2} (2,6)**	+23.0	+21.9	+20.3	+22.9	+19.3
**TS_{1-2} (2,5)**	+23.6	+22.6	+20.8	+24.0	+18.6
**Int 2 (2,6)**	+1.4	+1.8	+2.2	+1.6	-1.7
**Int 2 (2,5)**	+0.5	+0.9	+0.3	+2.2	-2.9
**R = R' = *p*-C_6H_4OMe**					
**Int 1**[a]	+0.0	+0.0	+0.0	+0.0	+0.0
**TS_{1-2} (2,6)**	+16.3	+15.7	+17.1	+18.2	+13.9
**TS_{1-2} (2.5)**	+16.3	+15.5	+17.2	+18.7	+14.6
**Int 2 (2.6)**	-8.0	-7.2	-4.2	-4.2	-6.9
**Int 2 (2.5)**	-6.6	-5.9	-2.7	-5.1	-8.8

[a] With R = R' = *p*-$C_6H_4CF_3$ the lowest energy form of **Int 1** at the G-D3(acetone) level features one κ^2-$CF_3CO_2^-$ ligand and one κ^1-bound form with the κ^2-bound ligand being *trans* to the benzoquinone moiety, whereas with R = R' = *p*-C_6H_4OMe the reverse is true, with the κ^1-$CF_3CO_2^-$ being *trans* to the benzoquinone moiety; all energies are quoted relative to these most stable forms in each case.

4.2.3 [PdR'(CF_3CO_2)(acetone)(3)]

Table S5a. Computed energetics for insertion at [PdR'(CF_3CO_2)(acetone)(**3**)] (R = R' = *p*-$C_6H_4CF_3$; R = R' = *p*-C_6H_4OMe), $CF_3CO_2^-$ trans to **3**.

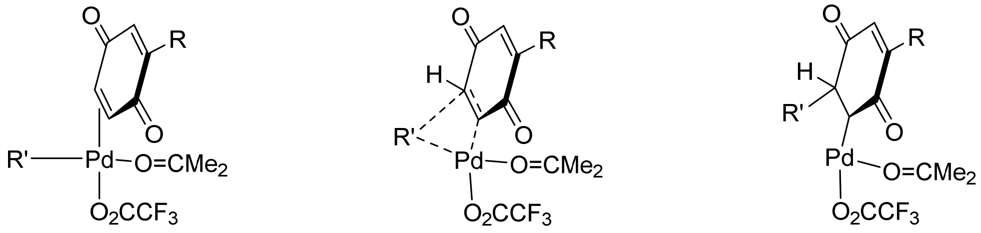

Int 1 **TS(1-2)** **Int 2**

R = R' = *p*-$C_6H_4CF_3$	ΔE	$ΔH_{298}$	ΔG	$ΔG_{disp}$	$ΔG_{Acetone+disp}$
**Int 1**	+0.0	+0.0	+0.0	+0.0	+0.0
**TS_{1-2} (2,6)**	+20.0	+19.2	+18.7	+21.8	+19.3
**TS_{1-2} (2,5)**	+19.9	+19.1	+18.5	+21.9	+19.4
**Int 2 (2,6)**	-1.5	-0.9	-1.4	+0.5	-1.9
**Int 2 (2,5)**	-1.9	-1.3	-1.5	-0.4	-2.7
**R = R' = *p*-C_6H_4OMe**					
**Int 1**	+0.0	+0.0	+0.0	+0.0	+0.0
**TS_{1-2} (2,6)**	+15.0	+14.3	+14.3	+15.0	+13.0
**TS_{1-2} (2,5)**	+14.8	+14.1	+14.0	+15.4	+13.7
**Int 2 (2,6)**	-7.2	-6.5	-6.1	-6.3	-7.4
**Int 2 (2,5)**	-7.3	-6.6	-5.6	-6.8	-8.4

Table S5b. Computed energetics for insertion at [PdR'(CF_3CO_2)(acetone)(**3**)] (R = R' = *p*-$C_6H_4CF_3$; R = R' = *p*-C_6H_4OMe), acetone trans to **3**.

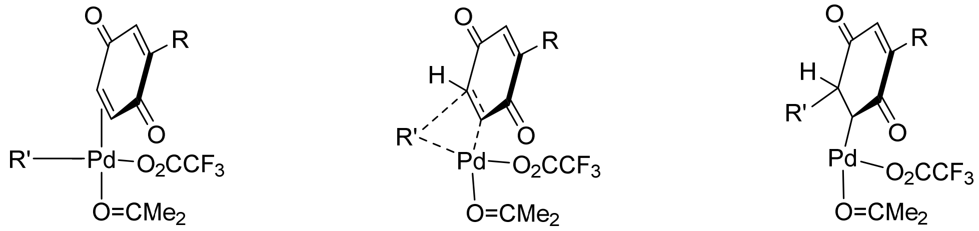

R = R' = *p*-$C_6H_4CF_3$	ΔE	ΔH_{298}	ΔG	ΔG_{disp}	$\Delta G_{Acetone+disp}$
**Int 1[a]**	+6.2	+6.2	+4.7	+5.3	+3.4
**TS_{1-2} (2,6)**	+19.8	+19.0	+17.8	+21.4	+18.4
**TS_{1-2} (2,5)**	+20.0	+19.1	+17.7	+21.1	+18.3
**Int 2 (2,6)**	-2.9	-2.3	-3.2	-0.4	-3.3
**Int 2 (2,5)**	-3.8	-3.1	3.6	-1.2	-3.8
**R = R' = *p*-C_6H_4OMe**					
**Int 1[a]**	+5.5	+5.5	+3.5	+4.6	+3.0
**TS_{1-2} (2,6)**	+15.9	+15.3	+15.6	+17.2	+14.2
**TS_{1-2} (2,5)**	+15.6	+14.9	+15.0	+17.1	+14.5
**Int 2 (2,6)**	-6.5	-5.8	-5.7	-4.0	-7.5
**Int 2 (2,5)**	-7.0	-6.3	-6.1	-4.9	-8.0

[a] Energies are quoted relative to the more stable isomers of [PdR'(CF_3CO_2)(acetone)(**3**)] with $CF_3CO_2^-$ trans to **3** set to 0.0 kcal/mol.

4.2.4 $[PdR'(CF_3CO_2)(acetone)_2(3)]^+$

Table S6. Computed energetics for insertion at $[PdR'(acetone)_2(\mathbf{3})]^+$ (R = R' = *p*-$C_6H_4CF_3$; R = R' = *p*-C_6H_4OMe).

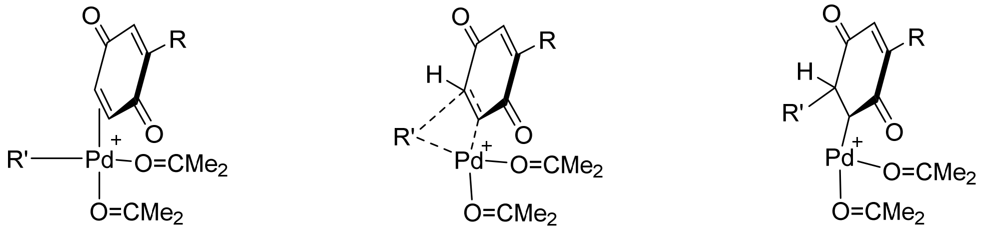

Int 1 TS(1-2) Int 2

R = R' = *p*-$C_6H_4CF_3$	ΔE	ΔH_{298}	ΔG	ΔG_{disp}	$\Delta G_{Acetone+disp}$
**Int 1**	+0.0	+0.0	+0.0	+0.0	+0.0
**TS_{1-2} (2,6)**	+9.5	+8.8	+9.7	+11.5	+14.5
**TS_{1-2} (2,5)**	+10.1	+9.3	+10.2	+12.3	+14.5
**Int 2 (2,6)**	-14.5	-13.6	-11.6	-11.2	-6.7
**Int 2 (2,5)**	-13.8	-13.0	-10.6	-11.0	-7.5
**R = R' = *p*-C_6H_4OMe**					
**Int 1**	+0.0	+0.0	+0.0	+0.0	+0.0
**TS_{1-2} (2,6)**	+5.7	+5.2	+7.2	+6.7	+9.6
**TS_{1-2} (2,5)**	+6.7	+6.0	+8.1	+7.9	+10.1
**Int 2 (2,6)**	-15.7	-14.7	-13.4	-13.9	-11.6
**Int 2 (2,5)**	-16.1	-15.2	-13.3	-13.8	-11.7

5. Computed Cartesian Coordinates (Å) and energies (au) for all species

5.1 Organic Intermediates and Products

3 (R=*p*-CF_3-C_6H_4)

25

C	1.856717	0.046320	-0.018135
C	4.778388	0.165398	0.056972
C	4.081317	1.311739	0.234197
C	2.593422	1.346737	0.214272
C	2.592119	-1.091221	-0.212133
C	4.071652	-1.122159	-0.179744
H	5.872639	0.125098	0.074355
H	4.563480	2.279499	0.408529
O	2.001480	2.419797	0.394637
H	2.112898	-2.053099	-0.424623
O	4.699219	-2.179473	-0.349868
C	0.375602	0.028144	-0.013378
C	-2.448845	-0.099277	-0.047606
C	-1.785858	1.019145	-0.581431
C	-0.388417	1.087439	-0.560943
C	-0.308938	-1.088419	0.525385
C	-1.704991	-1.154400	0.509891
H	-2.366564	1.833628	-1.022861
H	0.117773	1.959726	-0.976567
H	0.263797	-1.899968	0.985334
H	-2.221043	-2.021614	0.930569
C	-3.959245	-0.138274	-0.011523
F	-4.507692	0.541829	-1.057338
F	-4.444458	0.428150	1.131651
F	-4.432973	-1.415857	-0.055183

SCF(BP86) = -949.540604781
H 0K = -949.375198
H 298K = -949.360247
G 298K = -949.419669
Solvent Correction(Acetone) = -0.00770629
BP86-D3 Correction = -0.02553241
Lowest frequencies = 14.6594 cm-1, 42.4685 cm-1

5 (R=*p*-CF_3-C_6H_4)

38

C	0.008307	3.460537	-0.270758
C	-1.301954	1.339188	-0.139704
C	-0.005637	0.564624	-0.067503
C	-1.259169	2.700952	-0.238730
H	-2.171925	3.300683	-0.322160
C	-2.589113	0.605990	-0.070507
C	1.268562	2.689972	-0.221154
C	1.298211	1.327863	-0.121925
O	-0.012028	-0.666669	0.055660
C	2.578622	0.584562	-0.036372
H	2.187677	3.281626	-0.291601
O	0.014229	4.701050	-0.350636
C	-5.097871	-0.694356	0.024211
C	-4.914260	0.526917	0.695923
C	-3.672640	1.167723	0.646719
C	-2.787487	-0.629295	-0.732988
C	-4.031478	-1.268067	-0.689538
C	-6.419267	-1.421141	0.124714
H	-5.742227	0.970666	1.255257
H	-3.527456	2.105366	1.192592
H	-1.966913	-1.082400	-1.291191
H	-4.180387	-2.212523	-1.219869
C	5.075034	-0.736473	0.093579
C	4.891212	0.484938	0.764987
C	3.655328	1.135392	0.699382
C	2.775699	-0.653083	-0.694802
C	4.014056	-1.301436	-0.634992
C	6.426426	-1.413109	0.110902
H	5.708985	0.912415	1.351307
H	3.507173	2.069137	1.251082
H	1.956914	-1.103310	-1.257885
H	4.157429	-2.255558	-1.149312
F	6.316641	-2.766677	0.000887
F	7.111908	-1.145022	1.257947
F	7.205497	-0.990067	-0.927585
F	-6.669665	-2.169994	-0.986022
F	-7.460741	-0.556184	0.283865
F	-6.439459	-2.271094	1.192344

SCF(BP86) = -1517.62767378
H 0K = -1517.379421
H 298K = -1517.355810
G 298K = -1517.437611
Solvent Correction(Acetone) = -0.00927612
BP86-D3 Correction = -0.04336731
Lowest frequencies = 12.0451 cm-1, 12.6008 cm-1

4 (R=*p*-CF_3-C_6H_4)

38

C	0.697115	-1.256282	-0.081445
C	0.777898	1.203764	-0.095760
C	-0.697709	1.289389	-0.074893
C	1.483517	0.033291	-0.077678
C	2.962670	0.008100	-0.044374
C	-0.778626	-1.170673	-0.084303
C	-1.484012	-0.000161	-0.059617
O	-1.249444	2.401043	-0.067966
C	-2.962767	0.023728	-0.009568
H	-1.282100	-2.142800	-0.119903
O	1.249146	-2.367781	-0.079810
H	1.280694	2.175909	-0.139250
C	5.789430	0.055861	-0.009235
C	5.109013	-0.954351	-0.710462
C	3.710145	-0.983999	-0.725311
C	3.664817	1.015093	0.662646
C	5.061848	1.041357	0.681716
C	7.299372	0.046121	0.059356
H	5.677612	-1.713747	-1.253986
H	3.189027	-1.772611	-1.269489
H	3.104484	1.768018	1.225860
H	5.591495	1.823263	1.232585
C	-5.788933	-0.025226	0.060885
C	-5.052305	-1.008085	0.745974
C	-3.655560	-0.980544	0.710468
C	-3.719168	1.015723	-0.680729
C	-5.117730	0.984717	-0.649524
C	-7.299117	-0.086927	0.046121
H	-5.574507	-1.778371	1.319782
H	-3.087883	-1.726711	1.275295
H	-3.205036	1.808508	-1.225460
H	-5.693204	1.753087	-1.172842
F	-7.855850	1.149714	-0.082314
F	-7.793473	-0.647304	1.186034
F	-7.753449	-0.840231	-0.998101
F	7.806532	1.304243	0.194923
F	7.852826	-0.504687	-1.057248
F	7.743681	-0.680182	1.126041

SCF(BP86) = -1517.62957198
H 0K = -1517.381270
H 298K = -1517.357648
G 298K = -1517.439406
Solvent Correction(Acetone) = -0.00861415
BP86-D3 Correction = -0.04302233
Lowest frequencies = 12.6505 cm-1, 14.0474 cm-1

3 (R=*p*-OMe-C_6H_4)

26

C	-1.215337	0.060201	-0.029029
C	-4.139777	0.038622	0.089192
C	-3.496674	1.216793	0.257935
C	-2.012043	1.321669	0.225576
C	-1.906646	-1.110894	-0.218430
C	-3.375185	-1.214597	-0.160987
H	-5.230705	-0.054407	0.121088
H	-4.021571	2.160871	0.439326
O	-1.475891	2.422132	0.420975
H	-1.386370	-2.046383	-0.450380
O	-3.962748	-2.298046	-0.328421
C	0.258625	0.110723	-0.040156
C	3.099892	0.106244	-0.111858
C	2.373553	1.230731	-0.563888
C	0.981508	1.236513	-0.521435
C	1.006009	-1.002166	0.414394
C	2.404664	-1.016557	0.386437
O	4.460209	0.208323	-0.191139
H	2.930334	2.090261	-0.948112
H	0.437216	2.115262	-0.869629
H	0.479258	-1.865534	0.834009
H	2.940596	-1.889747	0.765892
C	5.238477	-0.910955	0.245218
H	5.011548	-1.818392	-0.345116
H	5.077074	-1.124228	1.318570
H	6.287669	-0.624934	0.085216

SCF(BP86) = -727.033391893
H 0K = -726.840661
H 298K = -726.826854
G 298K = -726.881844
Solvent Correction(Acetone) = -0.00928796
BP86-D3 Correction = -0.02505687
Lowest frequencies = 47.9643 cm-1, 55.3300 cm-1

5 (R=*p*-OMe-C_6H_4)

40

C	0.000002	3.056798	-0.273498
C	-1.302516	0.927465	-0.147920
C	-0.000001	0.162373	-0.062092
C	-1.260093	2.292417	-0.244216
H	-2.174604	2.888689	-0.333252
C	-2.581115	0.189456	-0.079609
C	1.260095	2.292414	-0.244212
C	1.302516	0.927462	-0.147917
O	-0.000003	-1.064618	0.107477
C	2.581114	0.189451	-0.079602
H	2.174607	2.888685	-0.333246
O	0.000003	4.301254	-0.351108
C	-5.099264	-1.126161	0.003948
C	-4.938500	0.130116	0.624999
C	-3.693234	0.768200	0.574630
C	-2.763338	-1.083349	-0.683821
C	-3.999561	-1.724768	-0.650167
H	-5.764644	0.606633	1.158058
H	-3.572031	1.728754	1.086269
H	-1.925454	-1.563487	-1.191517
H	-4.144211	-2.698150	-1.127782
C	5.099260	-1.126170	0.003962
C	4.938497	0.130108	0.625012
C	3.693232	0.768194	0.574639
C	2.763335	-1.083355	-0.683811
C	3.999558	-1.724777	-0.650154
H	5.764640	0.606622	1.158074
H	3.572029	1.728748	1.086278
H	1.925451	-1.563493	-1.191509
H	4.144207	-2.698159	-1.127768
O	-6.263566	-1.844089	-0.015427
O	6.263559	-1.844102	-0.015404
C	-7.406422	-1.271669	0.627010
C	7.406435	-1.271647	0.626969
H	7.233113	-1.122971	1.709527
H	7.690045	-0.305740	0.168147
H	8.222028	-1.995115	0.485826
H	-7.690087	-0.305770	0.168206
H	-7.233045	-1.122990	1.709558
H	-8.222003	-1.995160	0.485908

SCF(BP86) = -1072.61238288
H 0K = -1072.309467
H 298K = -1072.288176
G 298K = -1072.360912
Solvent Correction(Acetone) = -0.01253515
BP86-D3 Correction = -0.04245545
Lowest frequencies = 20.7153 cm-1, 34.2949 cm-1

4 (R=*p*-OMe-C_6H_4)

40

C	-0.702080	1.375677	-0.064089
C	-0.771227	-1.083923	-0.126120
C	0.697649	-1.168854	-0.074036
C	-1.488323	0.083648	-0.096999
C	-2.962017	0.100287	-0.081440
C	0.767530	1.291548	-0.049190
C	1.484158	0.123069	-0.028409
O	1.249738	-2.284145	-0.068173
C	2.955528	0.106505	0.051337
H	1.266137	2.266530	-0.068662
O	-1.256877	2.489294	-0.049380
H	-1.268355	-2.056915	-0.202779
C	-5.803702	0.027658	-0.087105
C	-5.113159	1.097148	-0.699512
C	-3.720868	1.137542	-0.690220
C	-3.674361	-0.957036	0.534141
C	-5.072652	-1.004022	0.539925
H	-5.697532	1.887063	-1.180274
H	-3.201183	1.974156	-1.158660
H	-3.119726	-1.745670	1.053079
H	-5.580214	-1.829957	1.043871
C	5.795637	0.172360	0.166312
C	5.033598	1.195979	0.775632
C	3.644190	1.158900	0.714621
C	3.737096	-0.916485	-0.539066
C	5.136588	-0.885947	-0.492050
H	5.559490	1.997586	1.302193
H	3.069881	1.941759	1.220332
H	3.236907	-1.744344	-1.043092
H	5.702329	-1.687675	-0.972747
O	-7.168310	0.089332	-0.148911
O	7.152101	0.299907	0.279984
C	-7.909720	-0.977405	0.450878
C	7.965852	-0.724805	-0.299988
H	-7.721039	-1.042445	1.539015
H	-7.673488	-1.950983	-0.018262
H	-8.968823	-0.737912	0.279510
H	9.005195	-0.434952	-0.089778
H	7.818131	-0.790383	-1.394292
H	7.760766	-1.712028	0.154654

SCF(BP86) = -1072.61520904
H 0K = -1072.312203
H 298K = -1072.290899
G 298K = -1072.363725
Solvent Correction(Acetone) = -0.01182318
BP86-D3 Correction = -0.04202658
Lowest frequencies = 17.4301 cm-1, 36.7105 cm-1

5.2 Stationary Points for [PdR'(CF_3CO_2)(3)] (R = R' = p-$C_6H_4CF_3$; R = R' = p-C_6H_4OMe)

R=P-C6H4CF3/Int1/

47

C	0.683303	-1.141763	2.620051
Pd	2.563538	-0.500301	0.170395
C	1.347565	-2.275821	0.470869
C	-0.077704	-2.216442	-0.007295
C	1.721729	-1.762383	1.728387
H	2.641720	-2.081679	2.235855
C	1.101397	0.841585	0.129767
C	-0.707097	-1.106967	2.123077
C	-1.101889	-1.581918	0.899812
O	-0.369684	-2.709282	-1.101868
C	-2.501846	-1.458458	0.433404
H	-1.433215	-0.668165	2.815952
O	0.987772	-0.716223	3.742560
H	1.961801	-3.015842	-0.059314
O	4.062475	0.684129	-0.762181
C	4.919720	-0.276133	-0.658250
C	6.361953	0.009328	-1.138081
O	4.627595	-1.400116	-0.149303
F	7.038860	-1.139691	-1.340944
F	7.009621	0.732673	-0.187605
F	6.352191	0.720684	-2.287861
C	-0.883631	2.806468	0.021292
C	-0.033261	2.729788	1.138761
C	0.968551	1.746361	1.196325
C	0.281335	0.930670	-1.006660
C	-0.717535	1.918901	-1.055346
C	-2.039300	3.775965	0.031875
H	-0.150276	3.436860	1.965453
H	1.619211	1.690281	2.074509
H	0.397225	0.240008	-1.847933
H	-1.369479	1.992528	-1.930299
C	-5.190401	-1.188916	-0.384156
C	-4.549684	-0.128392	0.280528
C	-3.219876	-0.265591	0.689615
C	-3.153860	-2.511391	-0.252541
C	-4.489505	-2.378782	-0.647575
C	-6.609931	-1.022834	-0.878418
H	-5.086286	0.804725	0.470326
H	-2.719347	0.578200	1.173062
H	-2.615789	-3.436990	-0.464171
H	-4.994191	-3.203750	-1.157450
F	-7.270708	-2.213678	-0.923898
F	-6.637101	-0.501308	-2.139048
F	-7.326243	-0.181609	-0.081713
F	-2.454632	4.098979	-1.222031
F	-1.733043	4.931518	0.682160
F	-3.123342	3.235758	0.683107

SCF(BP86) = -2172.45681861
H 0K = -2172.171194
H 298K = -2172.137618
G 298K = -2172.244865
Solvent Correction(Acetone) = -0.01549639
BP86-D3 Correction = -0.06945141
Lowest frequencies = 9.4107 cm-1, 11.1379 cm-1

R=P-C6H4CF3/TS(1-2) (2,5)

47

C	-0.188419	-1.917243	2.169351
Pd	-1.330313	1.136319	0.852477
C	-0.119720	0.599951	2.442964
C	1.305623	0.590461	1.988746
C	-0.929465	-0.597465	2.370659
H	-1.754099	-0.688920	3.086531
C	-2.093077	-0.775943	0.708092
C	1.119218	-1.840732	1.490806
C	1.841297	-0.681925	1.361191
O	2.032543	1.571387	2.179573
H	1.512204	-2.797841	1.130163
O	-0.656692	-2.971753	2.600551
H	-0.335214	1.358385	3.209153
O	-2.489434	2.157356	-0.744321
C	-1.819870	3.200715	-0.459363
C	-2.143538	4.534123	-1.168683
O	-0.908740	3.196262	0.444427
F	-2.649518	4.314772	-2.401521
F	-1.038426	5.303943	-1.281191
F	-3.068065	5.211952	-0.437360
C	-3.706903	-2.565896	-0.732849
C	-4.233454	-1.886396	0.381576
C	-3.428779	-1.004110	1.111116
C	-1.554554	-1.486106	-0.392408
C	-2.370079	-2.362744	-1.119004
H	-5.270409	-2.057808	0.683444
H	-3.844261	-0.481793	1.978508
H	-0.513148	-1.338621	-0.693767
H	-1.964810	-2.899357	-1.981256
C	3.175085	-0.654247	0.722129
C	5.681603	-0.652141	-0.580945
C	5.272758	-1.779957	0.153448
C	4.032145	-1.778961	0.796516
C	3.610894	0.482552	-0.002121
C	4.848030	0.476792	-0.655497
H	5.935124	-2.645938	0.234264
H	3.738099	-2.646557	1.395535
H	2.976553	1.369383	-0.054503
H	5.174616	1.356669	-1.216032
C	6.995199	-0.676553	-1.330139
C	-4.598275	-3.474922	-1.549135
F	-5.241681	-2.781421	-2.531259
F	-5.556230	-4.062825	-0.779601
F	-3.886927	-4.465112	-2.156546
F	7.919722	-1.455165	-0.701068
F	7.524705	0.570280	-1.464763
F	6.834644	-1.183069	-2.587416

SCF(BP86) = -2172.43413695
H 0K = -2172.148939
H 298K = -2172.116135
G 298K = -2172.222066
Solvent Correction(Acetone) = -0.01846996
BP86-D3 Correction = -0.06377113
Lowest frequencies = -231.4645 cm-1, 7.1076 cm-1

R=P-C6H4CF3/TS(1-2) (2,6)

47

C	-0.656634	-3.046924	0.569737
Pd	-2.452732	-0.710401	0.477352
C	-0.653914	-0.910311	1.939835
C	0.862499	-0.841415	1.757630
C	-1.340446	-2.114437	1.519602
H	-2.081736	-2.581850	2.183255
C	-1.003248	0.720171	0.797696
C	0.699507	-2.666024	0.099842
C	1.462039	-1.669779	0.653487
O	1.535200	-0.188552	2.552386
H	1.102303	-3.309625	-0.690381
O	-1.171402	-4.115093	0.212935
H	-0.948173	-0.474531	2.901638
O	-3.979559	0.463186	-0.623320
C	-4.642235	-0.620176	-0.711446
C	-6.046577	-0.602828	-1.354422
O	-4.201311	-1.725674	-0.233902
F	-6.969144	-0.325551	-0.394045
F	-6.346047	-1.800045	-1.902871
F	-6.126363	0.348004	-2.311050
C	-0.041852	3.286266	0.171793
C	0.262263	2.201149	-0.669612
C	-0.194931	0.915241	-0.351167
C	-1.288628	1.811692	1.651479
C	-0.817683	3.088990	1.330045
H	0.850044	2.363219	-1.577481
H	0.043346	0.076716	-1.011807
H	-1.897990	1.668127	2.548981
H	-1.058707	3.939373	1.973794
C	2.872089	-1.432184	0.267638
C	5.540139	-0.987805	-0.550008
C	5.001393	-2.286426	-0.584803
C	3.681699	-2.504090	-0.179526
C	3.433683	-0.133215	0.309819
C	4.752635	0.086317	-0.101200
H	5.617727	-3.122337	-0.926279
H	3.281132	-3.522564	-0.181435
H	2.829605	0.708694	0.654773
H	5.172122	1.095671	-0.081211
C	6.986347	-0.760718	-0.930299
C	0.525497	4.654477	-0.133120
F	7.187085	0.493432	-1.422065
F	7.402787	-1.649046	-1.875876
F	7.809667	-0.906706	0.147271
F	-0.263507	5.648735	0.357492
F	0.664752	4.853514	-1.472770
F	1.761964	4.808640	0.425598

SCF(BP86) = -2172.433652
H 0K = -2172.148410
H 298K = -2172.115622
G 298K = -2172.220953
Solvent Correction(Acetone) = -0.01897745
BP86-D3 Correction = -0.06578101
Lowest frequencies = -230.8918 cm-1, 9.3465 cm-1

R=P-C6H4CF3/Int2 (2,5)

47

C	-0.438800	-2.937267	-1.750212
Pd	1.398609	0.372561	-0.768218
C	0.168183	-0.445207	-2.216147
C	-1.237294	-0.089998	-1.862271
C	0.637254	-1.898694	-2.116081
H	1.124146	-2.238390	-3.045565
C	1.702210	-1.820648	-0.998007
C	-1.692047	-2.441914	-1.156026
C	-2.083076	-1.125500	-1.142583
O	-1.730077	0.994984	-2.199417
H	-2.326405	-3.222200	-0.718701
O	-0.216211	-4.143841	-1.886759
H	0.494222	0.100028	-3.116651
O	2.720187	1.722582	0.456384
C	2.210162	2.672737	-0.213777
C	2.706415	4.120702	-0.005343
O	1.310751	2.468877	-1.111003
F	3.246106	4.273444	1.222814
F	1.693807	5.004638	-0.150181
F	3.659500	4.401605	-0.933549
C	3.587644	-1.872411	1.120114
C	4.007250	-2.104530	-0.219415
C	3.087397	-2.074816	-1.257776
C	1.290120	-1.582258	0.359753
C	2.251600	-1.625926	1.410667
H	5.060289	-2.316971	-0.424383
H	3.407004	-2.271543	-2.285955
H	0.225882	-1.584357	0.619695
H	1.933423	-1.471242	2.444188
C	-3.367143	-0.699293	-0.542949
C	-5.787883	0.067453	0.697798
C	-5.679343	-1.196357	0.090221
C	-4.480844	-1.573538	-0.521609
C	-3.501033	0.579639	0.051954
C	-4.696316	0.952532	0.675733
H	-6.538794	-1.872004	0.082894
H	-4.417941	-2.545093	-1.021517
H	-2.665259	1.281289	0.023283
H	-4.788854	1.940010	1.135545
C	-7.061635	0.451406	1.416641
C	4.632705	-1.877592	2.215279
F	5.452511	-0.796863	2.107138
F	5.420045	-2.988040	2.130448
F	4.076940	-1.855480	3.454872
F	-8.155472	-0.133618	0.852587
F	-7.261297	1.798113	1.409814
F	-7.027039	0.057393	2.723366

SCF(BP86) = -2172.46977007
H 0K = -2172.182484
H 298K = -2172.149597
G 298K = -2172.255640
Solvent Correction(Acetone) = -0.01891378
BP86-D3 Correction = -0.06475277
Lowest frequencies = 9.3658 cm-1, 10.3320 cm-1

R=P-C6H4CF3/Int2 (2,6)

47

C	0.275868	-2.525832	0.301457
Pd	-2.042127	-0.770601	0.295908
C	0.134747	-0.522515	1.933622
C	1.648648	-0.313231	1.728750
C	-0.522967	-1.749212	1.295706
H	-1.084831	-2.405686	1.979480
C	-0.650481	0.644101	1.286460
C	1.608991	-1.992003	-0.093059
C	2.313992	-1.030616	0.587948
O	2.251706	0.465688	2.467711
H	2.068346	-2.522162	-0.936089
O	-0.097230	-3.614915	-0.160211
H	-0.037671	-0.488879	3.021932
O	-3.998728	-0.162507	-0.632093
C	-4.327174	-1.383682	-0.513141
C	-5.749145	-1.841577	-0.906574
O	-3.525621	-2.262208	-0.023451
F	-6.545479	-1.809360	0.195933
F	-5.737394	-3.103654	-1.389059
F	-6.278405	-1.022182	-1.840452
C	-1.899947	2.888298	0.083851
C	-1.181309	2.007422	-0.714304
C	-0.553912	0.866969	-0.132589
C	-1.393364	1.578559	2.077678
C	-2.000493	2.677508	1.487584
H	-1.089475	2.188004	-1.787805
H	0.167065	0.301612	-0.732918
H	-1.458495	1.424122	3.159204
H	-2.555047	3.393381	2.100811
C	3.724370	-0.715667	0.251593
C	6.392173	-0.144018	-0.487786
C	5.905916	-1.460347	-0.575253
C	4.586099	-1.740163	-0.209205
C	4.234210	0.601696	0.348800
C	5.552594	0.883749	-0.024895
H	6.561715	-2.260900	-0.927819
H	4.223515	-2.771976	-0.254207
H	3.593156	1.405756	0.714174
H	5.931750	1.907401	0.035823
C	7.836018	0.151923	-0.825547
C	-2.610073	4.081275	-0.519396
F	7.995822	1.424764	-1.284545
F	8.314311	-0.695705	-1.779341
F	8.638932	0.016900	0.269190
F	-3.959952	3.922788	-0.460899
F	-2.269973	4.276908	-1.820279
F	-2.310279	5.222061	0.166100

SCF(BP86) = -2172.46848869
H 0K = -2172.181219
H 298K = -2172.148321
G 298K = -2172.254349
Solvent Correction(Acetone) = -0.01944981
BP86-D3 Correction = -0.06426756
Lowest frequencies = 10.5375 cm-1, 11.7088 cm-1

R=P-C6H4OMe/Int1

49

C	-0.271731	-1.302340	2.430616
Pd	1.185816	0.169653	0.300247
C	0.444923	-1.840830	0.073813
C	-0.983925	-1.764979	-0.388426
C	0.793615	-1.633736	1.423935
H	1.747280	-1.990491	1.829737
C	3.043171	-0.463746	-0.042697
C	-1.645401	-1.159678	1.927870
C	-2.032914	-1.368637	0.622613
O	-1.252818	-2.072531	-1.555963
H	-2.373084	-0.839830	2.680853
O	0.015051	-1.164571	3.630898
H	1.115836	-2.367405	-0.614829
O	1.531677	2.247348	-0.078689
C	0.292603	2.528491	0.136222
C	-0.140720	3.999259	-0.065767
O	-0.565497	1.653887	0.455984
F	0.791428	4.850827	0.420582
F	-1.317609	4.249733	0.546861
F	-0.289338	4.241161	-1.396647
C	5.728284	-1.087387	-0.582184
C	4.769238	-1.157768	-1.611549
C	3.420824	-0.851412	-1.335070
C	3.994378	-0.402418	0.993332
C	5.333885	-0.708696	0.719590
O	7.062587	-1.366886	-0.736203
H	5.049565	-1.444652	-2.628178
H	2.688534	-0.900895	-2.148167
H	3.709123	-0.100190	2.006613
H	6.094081	-0.657227	1.504970
C	7.509001	-1.743334	-2.040233
H	7.021157	-2.674638	-2.386841
H	8.591326	-1.913831	-1.948886
H	7.326974	-0.941863	-2.781583
C	-3.438549	-1.254404	0.201443
C	-6.166916	-0.961798	-0.533233
C	-5.822048	-1.420164	0.760170
C	-4.484743	-1.556973	1.115517
C	-3.804818	-0.813123	-1.093797
C	-5.146750	-0.657548	-1.458845
O	-7.504309	-0.857642	-0.782947
H	-6.625626	-1.672428	1.457779
H	-4.237070	-1.940680	2.110441
H	-3.027115	-0.573652	-1.820276
H	-5.386981	-0.295680	-2.461200
C	-7.907520	-0.410611	-2.083131
H	-7.537821	0.610262	-2.292528
H	-9.006441	-0.404758	-2.067639
H	-7.554437	-1.097412	-2.874554

SCF(BP86) = -1727.44466601
H 0K = -1727.104406
H 298K = -1727.072953
G 298K = -1727.173883
Solvent Correction(Acetone) = -0.01933412
BP86-D3 Correction = -0.06026314
Lowest frequencies = 9.4732 cm-1, 11.9125 cm-1

R=P-C6H4OMe/TS(1-2) (2,5)

49

C	-0.159299	-2.421017	-1.653466
Pd	1.238501	0.662602	-0.717939
C	-0.037057	0.038552	-2.242669
C	-1.456238	0.198085	-1.796598
C	0.660324	-1.200811	-2.040526
H	1.502349	-1.438802	-2.699508
C	1.805159	-1.247742	-0.248543
C	-1.452802	-2.158694	-1.007142
C	-2.087295	-0.937150	-1.013995
O	-2.100341	1.201871	-2.125813
H	-1.914231	-3.031653	-0.532809
O	0.245446	-3.559095	-1.916324
H	0.244588	0.701904	-3.073004
O	2.482265	1.818272	0.713571
C	1.923116	2.864113	0.250200
C	2.384389	4.250373	0.752559
O	1.022846	2.815005	-0.658894
F	2.839814	4.181074	2.023852
F	1.374907	5.148897	0.703317
F	3.395686	4.699524	-0.039865
C	3.135535	-3.125226	1.379555
C	3.766995	-2.624412	0.219417
C	3.096169	-1.699617	-0.592810
C	1.163917	-1.777013	0.900811
C	1.831629	-2.691703	1.714748
O	3.690213	-4.032283	2.235544
H	4.772169	-2.948988	-0.060024
H	3.596386	-1.317287	-1.488581
H	0.152705	-1.455591	1.168407
H	1.363201	-3.092122	2.618596
C	5.008857	-4.508056	1.942229
H	5.042553	-5.034221	0.970054
H	5.255790	-5.214094	2.747479
H	5.745573	-3.683198	1.939110
C	-3.401748	-0.725374	-0.385403
C	-5.895037	-0.353453	0.925788
C	-5.538525	-1.624849	0.417250
C	-4.315270	-1.801722	-0.220303
C	-3.785572	0.543678	0.114086
C	-5.007476	0.731508	0.770495
O	-7.113922	-0.284813	1.535950
H	-6.249055	-2.449304	0.524911
H	-4.073765	-2.785679	-0.634826
H	-3.114203	1.395715	-0.006668
H	-5.258414	1.723136	1.153970
C	-7.533265	0.986885	2.045663
H	-6.847367	1.354450	2.831386
H	-8.528917	0.819306	2.479885
H	-7.605167	1.741517	1.240718

SCF(BP86) = -1727.42218041
H 0K = -1727.082370
H 298K = -1727.051796
G 298K = -1727.149224
Solvent Correction(Acetone) = -0.02067957
BP86-D3 Correction = -0.06308880
Lowest frequencies = -209.3217 cm-1, 8.5642 cm-1

R=P-C6H4OMe/TS(1-2) (2,6)

49

C	-0.288346	-2.828118	0.299274
Pd	-1.986382	-0.407870	0.454337
C	-0.155453	-0.831521	1.846421
C	1.344867	-0.771427	1.605774
C	-0.905426	-1.952472	1.345713
H	-1.671237	-2.431242	1.972336
C	-0.471015	0.934488	0.756351
C	1.065030	-2.465726	-0.171619
C	1.885092	-1.535404	0.424325
O	2.061758	-0.191429	2.423350
H	1.419721	-3.064021	-1.018250
O	-0.871089	-3.833491	-0.136033
H	-0.416806	-0.428925	2.831580
O	-3.521551	0.880474	-0.498572
C	-4.244849	-0.164568	-0.587083
C	-5.682613	-0.037695	-1.137918
O	-3.842924	-1.311901	-0.187216
F	-6.528594	0.257264	-0.112912
F	-6.092963	-1.191402	-1.709325
F	-5.765581	0.953338	-2.054818
C	0.707231	3.455531	0.292852
C	0.907538	2.406918	-0.629717
C	0.337838	1.147260	-0.384406
C	-0.640800	1.985342	1.693337
C	-0.067310	3.232508	1.457895
O	1.219120	4.714214	0.164783
H	1.498792	2.556337	-1.536215
H	0.499222	0.340495	-1.105456
H	-1.245129	1.832371	2.592924
H	-0.205910	4.060743	2.158987
C	1.981074	5.007918	-1.011678
H	2.888735	4.378784	-1.074532
H	2.274545	6.063036	-0.920076
H	1.377652	4.872181	-1.928311
C	3.285833	-1.325754	0.014079
C	5.962261	-0.921900	-0.851968
C	5.331916	-2.173907	-1.041066
C	4.021574	-2.365855	-0.615335
C	3.938711	-0.084517	0.205626
C	5.254682	0.124509	-0.224570
O	7.248856	-0.836642	-1.301215
H	5.902295	-2.981596	-1.508419
H	3.565511	-3.353106	-0.739255
H	3.404604	0.733712	0.692657
H	5.720061	1.099959	-0.064295
C	7.943634	0.399544	-1.102446
H	7.449406	1.233106	-1.635823
H	8.949032	0.246307	-1.519199
H	8.025138	0.650337	-0.028488

SCF(BP86) = -1727.42144901
H 0K = -1727.081586
H 298K = -1727.051040
G 298K = -1727.147884
Solvent Correction(Acetone) = -0.02198563
BP86-D3 Correction = -0.06527054
Lowest frequencies = -211.6658 cm-1, 10.3998 cm-1

R=P-C6H4OMe/Int2 (2,5)

49

C	-0.750014	-3.198608	-1.085749
Pd	1.219582	0.112568	-0.602916
C	-0.042377	-0.840148	-1.927482
C	-1.430487	-0.379341	-1.640984
C	0.370182	-2.272303	-1.590798
H	0.857370	-2.772184	-2.444986
C	1.430209	-2.054541	-0.478382
C	-1.971325	-2.566397	-0.575329
C	-2.307709	-1.240596	-0.746927
O	-1.885691	0.643297	-2.173905
H	-2.630738	-3.245462	-0.021964
O	-0.571629	-4.422318	-1.035588
H	0.305459	-0.453882	-2.899601
O	2.622303	1.530990	0.481502
C	2.076831	2.444695	-0.210385
C	2.593410	3.896789	-0.101142
O	1.132099	2.208894	-1.046936
F	3.166910	4.120355	1.103327
F	1.591433	4.789372	-0.273465
F	3.529526	4.114361	-1.065381
C	3.321859	-1.946385	1.651965
C	3.747254	-2.263817	0.329084
C	2.820355	-2.313862	-0.705653
C	1.025769	-1.720753	0.864320
C	1.965727	-1.686391	1.912024
O	4.154015	-1.885640	2.724516
H	4.798832	-2.468543	0.116833
H	3.149767	-2.579012	-1.715852
H	-0.039355	-1.643243	1.105394
H	1.658594	-1.453417	2.934653
C	5.556096	-2.109610	2.515484
H	5.751028	-3.129963	2.137393
H	6.021677	-1.992984	3.503750
H	5.982342	-1.365234	1.819032
C	-3.545378	-0.663861	-0.197957
C	-5.892271	0.430871	0.973342
C	-5.822538	-0.949539	0.670845
C	-4.670069	-1.480185	0.101341
C	-3.643660	0.719392	0.094625
C	-4.791628	1.263237	0.681911
O	-7.064178	0.852240	1.532326
H	-6.693987	-1.575881	0.881788
H	-4.650697	-2.544845	-0.152000
H	-2.806099	1.379266	-0.138721
H	-4.820446	2.332640	0.903222
C	-7.194375	2.246613	1.834198
H	-6.440113	2.573199	2.574096
H	-8.199958	2.365612	2.261632
H	-7.105789	2.867725	0.923715

SCF(BP86) = -1727.45893144
H 0K = -1727.116987
H 298K = -1727.086296
G 298K = -1727.184055
Solvent Correction(Acetone) = -0.02207144
BP86-D3 Correction = -0.06384067
Lowest frequencies = 9.5364 cm-1, 11.0177 cm-1

R=P-C6H4OMe/Int2 (2,6)

49

C	0.420047	-2.345754	0.265726
Pd	-1.748748	-0.411596	0.352507
C	0.492837	-0.331022	1.881386
C	2.007724	-0.238017	1.621006
C	-0.277976	-1.511605	1.288752
H	-0.857031	-2.120507	2.001937
C	-0.231839	0.885180	1.245325
C	1.760547	-1.901507	-0.193524
C	2.570158	-0.988443	0.444716
O	2.695882	0.479609	2.350215
H	2.137335	-2.461828	-1.057604
O	-0.052672	-3.407251	-0.173958
H	0.362626	-0.267327	2.974279
O	-3.671463	0.403875	-0.530203
C	-4.091885	-0.793402	-0.474628
C	-5.549328	-1.105173	-0.882590
O	-3.369336	-1.761863	-0.043073
F	-6.353498	-0.995013	0.210549
F	-5.668372	-2.359077	-1.372865
F	-5.987638	-0.232133	-1.819460
C	-1.304405	3.253222	0.080818
C	-0.664913	2.317673	-0.740637
C	-0.137363	1.128685	-0.169967
C	-0.879853	1.879603	2.055319
C	-1.396747	3.029906	1.489877
O	-1.861846	4.417337	-0.344099
H	-0.566835	2.475541	-1.816242
H	0.505782	0.495462	-0.790381
H	-0.948099	1.720084	3.136196
H	-1.893754	3.790234	2.098918
C	-1.862884	4.679313	-1.755398
H	-0.833016	4.775519	-2.146168
H	-2.391782	5.634266	-1.878626
H	-2.397101	3.884215	-2.306016
C	3.974290	-0.771686	0.039702
C	6.646391	-0.370146	-0.843623
C	6.028680	-1.633542	-0.990519
C	4.720027	-1.823651	-0.557458
C	4.617197	0.479810	0.194511
C	5.930158	0.687642	-0.246332
O	7.931181	-0.285267	-1.300003
H	6.605807	-2.450436	-1.433025
H	4.271557	-2.818003	-0.650131
H	4.080804	1.304814	0.666252
H	6.385144	1.672735	-0.118621
C	8.609637	0.966341	-1.148262
H	8.100422	1.774482	-1.706074
H	9.614599	0.812926	-1.566196
H	8.694582	1.255155	-0.084066

SCF(BP86) = -1727.4587155
H 0K = -1727.116687
H 298K = -1727.086068
G 298K = -1727.183410
Solvent Correction(Acetone) = -0.02217793
BP86-D3 Correction = -0.06337404
Lowest frequencies = 11.1954 cm-1, 11.6105 cm-1

5.3 Stationary Points for [PdR'(CF_3CO_2)$_2$(3)] (R = R' = p-$C_6H_4CF_3$; R = R' = p-C_6H_4OMe)

R=CF3/Int1

54

C	0.090953	-1.324992	2.542108
Pd	1.924511	-0.188956	0.269836
C	0.855192	-2.064270	0.246435
C	-0.554647	-2.072065	-0.233369
C	1.173588	-1.703205	1.593143
H	2.101392	-2.051231	2.062833
C	0.312460	0.993716	0.288032
C	-1.297612	-1.311987	2.017982
C	-1.636082	-1.646965	0.737215
O	-0.840738	-2.455992	-1.378084
C	-3.044901	-1.589206	0.275499
H	-2.063832	-1.030493	2.749986
O	0.311801	-1.070499	3.738859
H	1.553154	-2.666096	-0.357186
O	3.156391	1.708041	0.693357
O	3.818956	-1.182717	0.328849
C	4.163034	-2.213421	-0.373026
C	3.278000	1.851482	-0.560678
C	5.698771	-2.482704	-0.232110
O	3.491351	-2.978150	-1.081783
C	4.185249	2.992237	-1.082955
O	2.733102	1.096519	-1.426058
F	6.434662	-1.406932	-0.629923
F	6.099766	-3.546265	-0.975102
F	6.032233	-2.747979	1.067163
F	4.319544	3.988735	-0.171860
F	3.688667	3.538712	-2.223680
F	5.427357	2.509128	-1.358918
C	-1.910098	2.729247	0.318838
C	-1.093184	2.607366	1.459809
C	0.017053	1.749854	1.440931
C	-0.486558	1.144058	-0.861567
C	-1.598657	2.002658	-0.844429
C	-3.157995	3.560816	0.389437
H	-1.321999	3.188865	2.358899
H	0.648869	1.666811	2.330500
H	-0.251914	0.585121	-1.772679
H	-2.225892	2.106380	-1.734843
F	-2.986959	4.698352	1.128195
F	-4.196026	2.877716	0.991378
F	-3.612762	3.939712	-0.839420
C	-5.751036	-1.458216	-0.534888
C	-4.947994	-2.545425	-0.924373
C	-3.605410	-2.606208	-0.533854
C	-3.862414	-0.497331	0.649659
C	-5.200720	-0.428138	0.248441
C	-7.208915	-1.423971	-0.913355
H	-5.373531	-3.337981	-1.546555
H	-2.979107	-3.439155	-0.857270
H	-3.430458	0.329118	1.220595
H	-5.805944	0.440937	0.519180
F	-7.448615	-2.055024	-2.101145
F	-7.683345	-0.149282	-1.027060
F	-7.991590	-2.048862	0.022083

SCF(BP86) = -2698.79058074
H 0K = -2698.478115
H 298K = -2698.436983
G 298K = -2698.563190
Solvent Correction(Acetone) = -0.06003011

```
BP86-D3 Correction = -0.08622203
Lowest frequencies = 6.5531 cm-1, 10.4447 cm-1
```

R=P-C6H4CF3/TS(1-2) (2,5)

54

C	-0.119400	-1.406217	-2.813954
Pd	1.031842	1.067558	-0.566415
C	-0.215172	1.052224	-2.238338
C	-1.622386	0.874673	-1.824448
C	0.622409	-0.105378	-2.541533
H	1.417536	0.065942	-3.276520
C	1.761613	-0.817949	-1.115540
C	-1.398514	-1.585514	-2.093383
C	-2.098446	-0.545199	-1.540221
O	-2.416023	1.828326	-1.779883
C	-3.351958	-0.798072	-0.790111
H	-1.802372	-2.604705	-2.096727
O	0.303908	-2.237981	-3.622257
H	0.009182	2.013942	-2.717468
O	2.449290	1.090011	0.994899
O	0.403876	2.937965	0.099868
C	0.691266	3.906757	-0.710975
C	1.974716	0.561221	2.078706
C	0.318036	5.290609	-0.089486
O	1.222367	3.886149	-1.831978
C	3.024788	0.615966	3.233767
O	0.874636	0.030383	2.290278
F	0.208968	6.251507	-1.045123
F	1.291462	5.691717	0.781044
F	-0.856821	5.263517	0.594133
F	4.091263	-0.195986	2.957005
F	2.500895	0.200297	4.415831
F	3.512026	1.873662	3.424925
C	3.394485	-3.017866	-0.424413
C	3.900670	-1.997370	-1.253885
C	3.090048	-0.914844	-1.602305
C	1.258708	-1.861121	-0.293331
C	2.076396	-2.940407	0.061023
C	4.243032	-4.222154	-0.115441
H	4.932736	-2.050674	-1.612565
H	3.492434	-0.117668	-2.236142
H	0.248551	-1.790185	0.116271
H	1.697804	-3.717966	0.730645
F	3.914674	-4.794486	1.079314
F	5.573859	-3.917709	-0.070130
F	4.103342	-5.201220	-1.066139
C	-5.720548	-1.398128	0.640866
C	-5.646599	-0.241407	-0.154503
C	-4.476366	0.061098	-0.860198
C	-3.437818	-1.949730	0.031809
C	-4.606175	-2.250847	0.737092
C	-7.006172	-1.753264	1.342846
H	-6.507886	0.430016	-0.215211
H	-4.405351	0.967173	-1.462843
H	-2.561290	-2.595585	0.142198
H	-4.649567	-3.133943	1.380452
F	-6.789846	-2.480519	2.477519
F	-7.824723	-2.509550	0.545274
F	-7.722944	-0.647380	1.696494

SCF(BP86) = -2698.75297052
H 0K = -2698.441782
H 298K = -2698.400964
G 298K = -2698.530036
Solvent Correction(Acetone) = -0.06855836
BP86-D3 Correction = -0.08114884
Lowest frequencies = -253.7414 cm-1, 2.8859 cm-1

R=P-C6H4CF3/**TS(1-2) (2,6)**

54

C	0.319128	2.185886	-2.401578
Pd	-1.199780	0.856354	-0.379296
C	-0.559952	-0.203318	-2.338662
C	0.859906	-0.673918	-2.089096
C	-0.822467	1.239041	-2.404673
H	-1.705615	1.593449	-2.950914
C	-1.767232	-1.042539	-1.064602
C	1.615949	1.671631	-1.881892
C	1.888578	0.346580	-1.684077
O	1.149914	-1.864348	-2.266072
C	3.200824	-0.091492	-1.147524
H	2.383054	2.435491	-1.711202
O	0.228613	3.355169	-2.808616
H	-1.025250	-0.825513	-3.113810
O	0.709844	-0.413516	1.574425
O	-1.047259	2.834635	0.308282
C	-2.119709	3.485942	-0.015959
C	-0.384369	-0.203112	2.128042
C	-2.117937	4.932749	0.567179
O	-3.110402	3.106378	-0.658394
C	-0.525720	-0.619930	3.624858
O	-1.478860	0.274389	1.636068
F	-2.551106	4.912662	1.863223
F	-2.952199	5.750310	-0.126441
F	-0.885297	5.507407	0.558127
F	0.635990	-0.422716	4.304619
F	-0.820911	-1.955242	3.706356
F	-1.502814	0.050908	4.287620
C	-3.497102	-3.198274	-0.538930
C	-2.144851	-3.266065	-0.152868
C	-1.278064	-2.199954	-0.420254
C	-3.122900	-0.988112	-1.476542
C	-3.985772	-2.052966	-1.197200
C	-4.404779	-4.377875	-0.318060
H	-1.774988	-4.149214	0.375979
H	-0.237634	-2.243305	-0.090263
H	-3.502701	-0.098455	-1.990114
H	-5.040405	-1.995908	-1.482351
F	-5.706071	-4.001905	-0.137017
F	-4.040032	-5.117895	0.769089
F	-4.398274	-5.235577	-1.390013
C	5.739335	-0.793757	-0.122746
C	5.183685	-1.518265	-1.192137
C	3.923819	-1.176385	-1.696949
C	3.762876	0.615343	-0.058533
C	5.020496	0.272124	0.447687
C	7.122935	-1.124341	0.372049
H	5.737399	-2.356896	-1.624110
H	3.485599	-1.750137	-2.514806
H	3.179490	1.409472	0.416062
H	5.435584	0.814053	1.301906
F	7.420463	-2.450075	0.226435
F	7.290468	-0.812369	1.689975
F	8.087615	-0.434499	-0.316363

SCF(BP86) = -2698.75400307
H 0K = -2698.443095
H 298K = -2698.402127
G 298K = -2698.530819
Solvent Correction(Acetone) = -0.06577236
BP86-D3 Correction = -0.08213379
Lowest frequencies = -278.0564 cm-1, 5.9501 cm-1

R=P-C6H4CF3/Int2 (2,5)

54

C	-0.833148	-1.290658	-3.387828
Pd	1.064110	0.599320	-0.513905
C	-0.185441	0.920552	-2.169441
C	-1.577137	0.993886	-1.697851
C	0.263644	-0.291338	-2.995676
H	0.769688	0.021942	-3.924811
C	1.307099	-0.985718	-2.090509
C	-2.061458	-1.288110	-2.572736
C	-2.391455	-0.297707	-1.679990
O	-2.130157	2.066453	-1.392387
C	-3.594313	-0.439901	-0.824641
H	-2.738412	-2.132956	-2.753032
O	-0.666632	-2.108525	-4.302502
H	0.179443	1.890714	-2.534999
O	2.528357	0.376615	1.002808
O	0.784086	2.521635	0.187040
C	1.285527	3.455104	-0.561303
C	2.084981	-0.183607	2.081562
C	1.132872	4.867138	0.087409
O	1.866598	3.382816	-1.653018
C	3.132483	-0.092966	3.237746
O	0.996205	-0.735755	2.305041
F	0.832776	5.800143	-0.858127
F	2.314383	5.238273	0.664210
F	0.172733	4.934635	1.043335
F	4.388384	-0.427947	2.824016
F	2.816232	-0.918113	4.270542
F	3.191303	1.176691	3.734207
C	3.140284	-2.549559	-0.593685
C	3.564840	-1.881541	-1.776188
C	2.670504	-1.115798	-2.506170
C	0.895323	-1.640624	-0.881217
C	1.828166	-2.436037	-0.154507
C	4.123581	-3.431130	0.132120
H	4.605787	-1.970579	-2.100320
H	2.994183	-0.605804	-3.419025
H	-0.164579	-1.719661	-0.620921
H	1.504102	-2.926200	0.765448
F	3.714182	-3.750198	1.388641
F	5.352767	-2.847184	0.230649
F	4.315782	-4.616067	-0.536855
C	-5.888423	-0.828535	0.792242
C	-5.564032	0.454115	0.317508
C	-4.430388	0.651304	-0.479426
C	-3.926677	-1.723448	-0.320196
C	-5.058975	-1.919440	0.474705
C	-7.148558	-1.046464	1.589404
H	-6.199730	1.304944	0.578869
H	-4.160186	1.644360	-0.840875
H	-3.265286	-2.569802	-0.530254
H	-5.290666	-2.912899	0.868699
F	-7.034016	-2.094063	2.457748
F	-8.217196	-1.328655	0.778767
F	-7.494758	0.052953	2.319202

SCF(BP86) = -2698.78974886
H 0K = -2698.476364
H 298K = -2698.435547
G 298K = -2698.562669
Solvent Correction(Acetone) = -0.06811230
BP86-D3 Correction = -0.08321470
Lowest frequencies = 8.0381 cm-1, 8.3308 cm-1

R=P-C6H4CF3/Int2 (2,6)

54

C	-0.872228	1.549667	2.547648
Pd	1.380754	0.521136	0.968717
C	-0.241890	-0.945638	2.622179
C	-1.692954	-1.231761	2.197118
C	0.186449	0.533605	2.696968
H	0.870170	0.776013	3.526643
C	0.763536	-1.535630	1.599547
C	-2.068563	1.156323	1.752115
C	-2.478823	-0.137249	1.563041
O	-2.147170	-2.372952	2.367416
C	-3.680625	-0.431402	0.742029
H	-2.683444	1.992737	1.405339
O	-0.814208	2.695001	3.029430
H	-0.117271	-1.474151	3.587948
O	4.426307	0.701755	0.513094
O	1.144610	2.527366	0.489923
C	0.100006	2.822825	-0.211238
C	3.818000	0.579348	-0.561365
C	0.104205	4.307876	-0.690198
O	-0.855363	2.111181	-0.564585
C	4.616661	0.703245	-1.898264
O	2.559542	0.371161	-0.783193
F	0.926106	5.113203	0.026272
F	-1.149419	4.836396	-0.621396
F	0.501074	4.368405	-1.994976
F	5.958431	0.644752	-1.691458
F	4.349695	1.894669	-2.507650
F	4.300276	-0.286972	-2.781285
C	2.733211	-2.846553	0.027060
C	1.354080	-2.969045	-0.295900
C	0.389770	-2.331658	0.469337
C	2.162739	-1.398774	1.901171
C	3.134149	-2.081016	1.114132
C	3.744462	-3.623486	-0.776452
H	1.056622	-3.568565	-1.160841
H	-0.667671	-2.458162	0.225459
H	2.483914	-0.965370	2.854201
H	4.192513	-1.969028	1.357322
F	5.002611	-3.120365	-0.658234
F	3.430115	-3.651789	-2.102793
F	3.802727	-4.934448	-0.367379
C	-5.951416	-0.879106	-0.882000
C	-5.721206	-1.682442	0.249269
C	-4.594592	-1.466649	1.051807
C	-3.911995	0.358013	-0.412723
C	-5.038548	0.139508	-1.212740
C	-7.198040	-1.074616	-1.703305
H	-6.424319	-2.483334	0.496436
H	-4.402117	-2.102142	1.917612
H	-3.174320	1.118305	-0.693319
H	-5.200036	0.745286	-2.108966
F	-7.623147	-2.374291	-1.695728
F	-7.020528	-0.714474	-3.008441
F	-8.244251	-0.324451	-1.232587

SCF(BP86) = -2698.78829941
H 0K = -2698.474747
H 298K = -2698.434059
G 298K = -2698.559686
Solvent Correction(Acetone) = -0.06539725
BP86-D3 Correction = -0.08713983
Lowest frequencies = 9.7755 cm-1, 10.0606 cm-1

R=P-C6H4OMe/Int1

56

C	-0.410847	-2.879809	0.567298
Pd	1.439163	-0.430426	-0.087095
C	0.286022	-1.587976	-1.485157
C	-1.137975	-1.244260	-1.769118
C	0.640156	-2.415134	-0.373890
H	1.552347	-3.021803	-0.396541
C	-0.110472	0.422024	0.836453
C	-1.795774	-2.421017	0.316238
C	-2.175580	-1.638942	-0.741515
O	-1.457389	-0.718479	-2.845934
C	-3.580817	-1.200234	-0.900967
H	-2.535962	-2.784104	1.038931
O	-0.166181	-3.661124	1.505920
H	0.929243	-1.526643	-2.371602
O	3.322098	-0.875728	1.460951
O	2.321104	1.485529	-0.184835
C	2.039442	2.209029	-1.223278
C	3.899305	-1.249588	0.407099
C	2.826805	3.559380	-1.173069
O	1.276291	1.992883	-2.172698
C	5.378801	-1.712373	0.482821
O	3.373716	-1.301780	-0.761352
F	2.424516	4.322985	-0.113824
F	2.635002	4.298732	-2.296859
F	4.169733	3.359920	-1.044633
F	5.895187	-1.588558	1.730289
F	6.164097	-0.991815	-0.363783
F	5.486979	-3.026686	0.122639
C	-2.105240	1.698319	2.362680
C	-1.284628	0.721735	2.957355
C	-0.285277	0.088894	2.187343
C	-0.929744	1.401837	0.244878
C	-1.923638	2.034932	1.006442
H	-1.396555	0.448515	4.010591
H	0.355814	-0.662062	2.662396
H	-0.775539	1.684957	-0.801235
H	-2.567922	2.801640	0.563871
C	-6.305823	-0.428200	-1.109510
C	-5.571401	-0.773689	-2.262875
C	-4.229815	-1.145191	-2.161814
C	-4.330906	-0.838941	0.240188
C	-5.676567	-0.456096	0.150329
H	-6.076112	-0.739476	-3.233323
H	-3.663976	-1.391988	-3.061219
H	-3.833209	-0.814404	1.214773
H	-6.210685	-0.161957	1.057284
O	-7.619305	-0.071249	-1.321293
O	-3.113255	2.384722	3.022678
C	-3.275236	2.107783	4.407107
C	-8.382459	0.305686	-0.177781
H	-4.092993	2.758010	4.755608
H	-2.358281	2.335795	4.986698
H	-3.551372	1.049607	4.592494
H	-7.954350	1.192429	0.328777
H	-8.461108	-0.520296	0.556134
H	-9.387099	0.552972	-0.552738

SCF(BP86) = -2253.75368579
H 0K = -2253.387381
H 298K = -2253.348213
G 298K = -2253.470664
Solvent Correction(Acetone) = -0.07426802
BP86-D3 Correction = -0.08308749
Lowest frequencies = 1.4270 cm-1, 6.5509 cm-1

R=**P-C6H4OMe/TS(1-2) (2,5)**

56

C	0.683435	-2.158241	2.217625
Pd	-0.987526	0.549688	0.641390
C	0.418380	0.358612	2.188836
C	1.843202	0.484180	1.814095
C	-0.225672	-0.944063	2.240685
H	-1.063646	-1.065099	2.937135
C	-1.154235	-1.507385	0.524618
C	1.987717	-1.976316	1.553172
C	2.534535	-0.750602	1.258407
O	2.476730	1.530064	2.036928
C	3.812736	-0.652317	0.520791
H	2.544613	-2.901643	1.365252
O	0.364689	-3.221951	2.765859
H	0.072141	1.155586	2.860804
O	-2.624045	0.721661	-0.686928
O	-0.773676	2.634503	0.543181
C	0.095878	2.949957	-0.364220
C	-3.762438	0.703208	-0.075718
C	0.204082	4.498324	-0.528486
O	0.785920	2.220199	-1.090496
C	-4.961125	0.832054	-1.069481
O	-4.028777	0.553342	1.128315
F	-0.903085	4.994268	-1.158219
F	1.283558	4.854515	-1.273023
F	0.310562	5.141325	0.669440
F	-4.676171	1.566122	-2.177320
F	-6.045459	1.401642	-0.475890
F	-5.347728	-0.413010	-1.497697
C	-1.941526	-3.841575	-0.866173
C	-2.737022	-3.345616	0.188874
C	-2.337776	-2.194451	0.879574
C	-0.350850	-2.033707	-0.525589
C	-0.751944	-3.173213	-1.225841
H	-3.671959	-3.837620	0.469620
H	-2.980469	-1.787853	1.665216
H	0.565052	-1.513137	-0.820975
H	-0.162023	-3.564621	-2.060708
C	6.255122	-0.575882	-0.933678
C	5.961893	0.415225	0.025824
C	4.761919	0.382935	0.735883
C	4.122110	-1.628203	-0.456440
C	5.321812	-1.602520	-1.179292
H	6.694526	1.210263	0.195354
H	4.524671	1.159717	1.464147
H	3.388889	-2.409218	-0.682680
H	5.506748	-2.366990	-1.938196
O	7.464501	-0.448068	-1.577294
O	-2.237565	-4.965490	-1.603764
C	-3.451384	-5.646962	-1.294193
C	7.778831	-1.418433	-2.573603
H	-3.508408	-6.494349	-1.994199
H	-4.335321	-4.995896	-1.436550
H	-3.454061	-6.031932	-0.255746
H	7.044724	-1.409036	-3.402419
H	7.828194	-2.441393	-2.151643
H	8.769134	-1.140315	-2.964372

SCF(BP86) = -2253.72770902
H 0K = -2253.362021
H 298K = -2253.323505
G 298K = -2253.443310
Solvent Correction(Acetone) = -0.08088742
BP86-D3 Correction = -0.08058099
Lowest frequencies = -234.2764 cm-1, 4.0131 cm-1

R=P-C6H4OMe/TS(1-2) (2,6)

56

C	0.137972	-2.878041	0.105501
Pd	1.469699	-0.261984	-0.381212
C	-0.131862	-1.241438	-1.791311
C	-1.635387	-1.400963	-1.673205
C	0.727177	-2.121626	-1.021607
H	1.622419	-2.548072	-1.492645
C	-0.189628	0.684430	-1.199333
C	-1.281516	-2.584340	0.449477
C	-2.155420	-1.887918	-0.345213
O	-2.358035	-1.210711	-2.658683
C	-3.569824	-1.679631	0.048537
H	-1.632604	-3.077463	1.363961
O	0.743460	-3.768565	0.728423
H	0.158962	-1.056987	-2.831859
O	1.085209	1.610145	2.055016
O	3.172717	-1.089402	0.507349
C	3.979957	-1.684899	-0.308135
C	1.859452	2.098839	1.218967
C	5.292448	-2.140632	0.406269
O	3.883225	-1.901589	-1.527543
C	2.445429	3.521113	1.497039
O	2.259097	1.643247	0.075611
F	6.230663	-1.146955	0.336248
F	5.827487	-3.239898	-0.193750
F	5.119862	-2.443042	1.719818
F	2.361701	3.856459	2.812751
F	1.742164	4.468793	0.797878
F	3.751690	3.641979	1.133305
C	-1.739276	3.029137	-1.553346
C	-1.916862	2.239229	-0.401320
C	-1.160499	1.068613	-0.239811
C	-0.037673	1.491153	-2.360051
C	-0.791278	2.648557	-2.532518
H	-2.627424	2.525235	0.377978
H	-1.284128	0.479814	0.671601
H	0.703121	1.213313	-3.117339
H	-0.663807	3.286513	-3.412683
C	-6.258664	-1.316728	0.900780
C	-5.953924	-1.449561	-0.469598
C	-4.634007	-1.623764	-0.889305
C	-3.895762	-1.528568	1.416077
C	-5.217304	-1.351452	1.848951
H	-6.774865	-1.415200	-1.192642
H	-4.400084	-1.713619	-1.951454
H	-3.092556	-1.520259	2.160412
H	-5.417755	-1.225692	2.916009
O	-7.590865	-1.148798	1.205699
O	-2.435099	4.182696	-1.825257
C	-3.373044	4.623785	-0.844442
C	-7.931004	-1.015576	2.584155
H	-3.804510	5.556280	-1.238439
H	-2.882593	4.828857	0.126271
H	-4.182167	3.884059	-0.688976
H	-7.460359	-0.122956	3.040428
H	-7.639494	-1.909937	3.168576
H	-9.025114	-0.903112	2.616001

SCF(BP86) = -2253.72765333
H 0K = -2253.361825
H 298K = -2253.323261
G 298K = -2253.443338
Solvent Correction(Acetone) = -0.08118333
BP86-D3 Correction = -0.08138742
Lowest frequencies = -236.1164 cm-1, 6.2227 cm-1

R=P-C6H4OMe/Int2 (2,5)

56

C	-0.731291	-3.459703	0.276678
Pd	1.002378	-0.338687	-0.655545
C	-0.228746	-1.741204	-1.605994
C	-1.654907	-1.356701	-1.568769
C	0.255411	-2.946661	-0.778448
H	0.477560	-3.828106	-1.416542
C	1.581418	-2.435496	-0.155168
C	-2.011966	-2.772222	0.444584
C	-2.463674	-1.741804	-0.342946
O	-2.201877	-0.771690	-2.520986
C	-3.763483	-1.101192	-0.057606
H	-2.638220	-3.170615	1.252379
O	-0.442590	-4.451394	0.971298
H	0.116642	-1.742144	-2.653243
O	3.772570	1.300946	-1.266870
O	-0.094950	1.363652	-1.124372
C	-0.989015	1.651848	-0.231539
C	3.295464	1.474616	-0.132653
C	-1.604708	3.062235	-0.494151
O	-1.366028	1.005694	0.755641
C	4.025670	2.523990	0.769476
O	2.282206	0.937630	0.455673
F	-0.721186	4.038787	-0.128879
F	-2.740498	3.256748	0.226253
F	-1.915920	3.264171	-1.803896
F	5.384760	2.321953	0.739737
F	3.655788	2.501524	2.075548
F	3.805999	3.787295	0.304515
C	4.202632	-1.786508	0.755375
C	3.906558	-1.669242	-0.602635
C	2.594804	-1.968617	-1.058439
C	1.938414	-2.575331	1.226396
C	3.212126	-2.258121	1.665030
H	4.634859	-1.289137	-1.318958
H	2.433072	-2.027617	-2.139985
H	1.198936	-2.960015	1.932021
H	3.483734	-2.346808	2.721330
C	-6.271710	0.055302	0.598334
C	-5.912038	-0.151918	-0.749075
C	-4.675404	-0.711451	-1.073398
C	-4.139755	-0.875711	1.286372
C	-5.373439	-0.304616	1.622421
H	-6.620221	0.142136	-1.529939
H	-4.386612	-0.848634	-2.116105
H	-3.424953	-1.105025	2.082024
H	-5.609568	-0.121227	2.673627
O	-7.511059	0.618921	0.807530
O	5.414644	-1.486641	1.319787
C	6.392281	-0.883711	0.461091
C	-7.883042	0.887887	2.157009
H	7.247958	-0.643401	1.109484
H	6.003253	0.040183	-0.000562
H	6.721199	-1.586071	-0.329985
H	-7.183113	1.594778	2.642984
H	-7.933607	-0.037580	2.763945
H	-8.883715	1.343643	2.109811

SCF(BP86) = -2253.76416362
H 0K = -2253.395998
H 298K = -2253.357647
G 298K = -2253.474889
Solvent Correction(Acetone) = -0.07803183
BP86-D3 Correction = -0.08699357
Lowest frequencies = 9.3326 cm-1, 11.1584 cm-1

R=P-C6H4OMe/Int2 (2,6)

56

C	0.763264	-2.462803	-0.300230
Pd	-1.202723	-0.371436	0.286006
C	0.885883	-1.117459	1.883361
C	2.411521	-1.102344	1.713905
C	0.056259	-1.927910	0.878732
H	-0.657463	-2.636678	1.322750
C	0.344206	0.329748	1.740476
C	2.131256	-1.931082	-0.584925
C	2.949005	-1.308039	0.325342
O	3.141136	-0.885411	2.689156
C	4.331272	-0.898929	-0.029764
H	2.522013	-2.218994	-1.569240
O	0.329587	-3.368459	-1.038295
H	0.693287	-1.445579	2.918863
O	-1.521882	1.858755	-1.983564
O	-2.578366	-1.639860	-0.605543
C	-3.196117	-2.445669	0.198574
C	-2.429677	1.886894	-1.135290
C	-4.286470	-3.283796	-0.541669
O	-3.076709	-2.615282	1.420827
C	-3.578628	2.915682	-1.392697
O	-2.598533	1.206586	-0.054002
F	-5.516887	-2.719258	-0.343103
F	-4.342256	-4.552475	-0.048290
F	-4.096555	-3.379766	-1.882233
F	-4.308191	2.560476	-2.487507
F	-3.053857	4.162062	-1.656975
F	-4.444716	3.070174	-0.358774
C	-0.291851	3.100801	1.608997
C	0.259139	2.474109	0.492539
C	0.566177	1.084810	0.542199
C	-0.217940	1.013341	2.868486
C	-0.524482	2.359777	2.805885
H	0.410803	3.003330	-0.448759
H	1.152519	0.661642	-0.278800
H	-0.395739	0.450960	3.791047
H	-0.961551	2.884465	3.660887
C	6.956964	-0.137592	-0.815006
C	6.700459	-0.567242	0.503149
C	5.411129	-0.936944	0.890784
C	4.608323	-0.451655	-1.341809
C	5.898218	-0.075519	-1.742113
H	7.534201	-0.605426	1.211187
H	5.216230	-1.253902	1.916241
H	3.788762	-0.370914	-2.063830
H	6.059732	0.275658	-2.764456
O	8.261925	0.204397	-1.091538
O	-0.637790	4.424874	1.679091
C	-0.507613	5.191269	0.475625
C	8.552925	0.639385	-2.417888
H	-0.852912	6.204380	0.730469
H	-1.134903	4.779513	-0.334134
H	0.546467	5.239365	0.139414
H	7.996311	1.559911	-2.681154
H	8.323056	-0.142672	-3.167600
H	9.632330	0.852306	-2.436283

SCF(BP86) = -2253.76639427
H 0K = -2253.398190
H 298K = -2253.359753
G 298K = -2253.477381
Solvent Correction(Acetone) = -0.07853417
BP86-D3 Correction = -0.08305070
Lowest frequencies = 8.1735 cm-1, 9.2726 cm-1

5.4a Stationary Points for [PdR’(CF_3CO_2)(acetone)(3)] (R = R' = p-$C_6H_4CF_3$; R = R' = p-C_6H_4OMe; BQ trans to acetone)

R=P-C6H4CF3/Int1

57

C	0.453636	-0.788577	2.519580
Pd	2.170401	0.156721	0.065248
C	1.183491	-1.779891	0.317615
C	-0.254004	-1.881933	-0.111711
C	1.527536	-1.260142	1.580958
H	2.499649	-1.472097	2.043368
C	0.484379	1.238917	-0.004696
C	-0.948797	-0.951121	2.085327
C	-1.321136	-1.449130	0.865033
O	-0.529141	-2.346864	-1.222626
C	-2.742946	-1.532860	0.459843
H	-1.700139	-0.639652	2.819425
O	0.742043	-0.316813	3.629199
H	1.894368	-2.374761	-0.279049
O	4.127815	-0.675656	0.205642
C	4.516457	-1.693466	-0.508546
C	4.096439	2.403017	-0.683725
C	6.066386	-1.863729	-0.422034
O	3.847874	-2.487073	-1.179333
O	3.094117	1.740188	-1.037228
F	6.698945	-0.673568	-0.672821
F	6.528281	-2.769217	-1.306886
F	6.433542	-2.262985	0.827483
C	-1.810981	2.858277	-0.143425
C	-0.992641	2.895725	1.000473
C	0.153665	2.087732	1.070217
C	-0.317673	1.225737	-1.160245
C	-1.468751	2.030705	-1.226017
C	-3.100419	3.639470	-0.152388
H	-1.251991	3.552629	1.836659
H	0.766364	2.110865	1.977950
H	-0.067933	0.579422	-2.008446
H	-2.102157	2.009985	-2.117400
F	-2.962026	4.858570	0.442250
F	-4.083501	2.978613	0.543939
F	-3.576001	3.843372	-1.410335
C	-5.472455	-1.669346	-0.247182
C	-4.608259	-2.731863	-0.564682
C	-3.252463	-2.661237	-0.226472
C	-3.623419	-0.468168	0.766450
C	-4.974627	-0.531769	0.412432
C	-6.945452	-1.768945	-0.571678
H	-4.997038	-3.609215	-1.088486
H	-2.584973	-3.484291	-0.487862
H	-3.235242	0.437856	1.240459
H	-5.636156	0.311886	0.624993
F	-7.172309	-2.547258	-1.666923
F	-7.494364	-0.545319	-0.812750
F	-7.643665	-2.322582	0.463071
C	4.746311	3.306628	-1.695908
H	5.819412	3.056710	-1.776678
H	4.693334	4.352364	-1.342525
H	4.259840	3.215695	-2.676258
C	4.686290	2.297219	0.692040
H	5.256573	1.348432	0.717539
H	3.889069	2.202306	1.447648
H	5.353412	3.138295	0.931272

SCF(BP86) = -2365.62487723
H 0K = -2365.256229
H 298K = -2365.215711
G 298K = -2365.339508
Solvent Correction(Acetone) = -0.02112290
BP86-D3 Correction = -0.08826754

Lowest frequencies = 11.1795 cm-1, 12.9217 cm-1

R=**P-C6H4CF3/TS(1-2) (2,5)**

57

C	-0.618748	-1.832361	2.351050
Pd	-1.326785	1.166056	0.687675
C	-0.224570	0.667549	2.365788
C	1.216862	0.404949	2.052808
C	-1.183978	-0.416937	2.391218
H	-2.031057	-0.324412	3.080149
C	-2.339466	-0.645435	0.706834
C	0.708188	-1.993492	1.728790
C	1.579611	-0.957296	1.495461
O	2.069803	1.252813	2.334870
C	2.876093	-1.191182	0.820538
H	1.002129	-3.030150	1.530660
O	-1.235301	-2.769645	2.863137
H	-0.365239	1.526755	3.036771
O	-2.530627	1.694078	-1.055488
O	-0.369075	3.006238	0.434021
C	0.643812	2.852620	-0.373746
C	-3.118890	2.779322	-1.248190
C	1.289873	4.225925	-0.724103
O	1.077830	1.808145	-0.872908
F	2.357000	4.088123	-1.536311
F	1.684709	4.891991	0.390427
F	0.368757	5.009985	-1.371115
C	-4.094440	-2.432633	-0.572205
C	-4.565139	-1.608573	0.466814
C	-3.690113	-0.727213	1.113445
C	-1.863192	-1.498268	-0.318075
C	-2.744393	-2.374038	-0.964279
C	-5.025768	-3.429401	-1.221616
H	-5.616035	-1.657870	0.765957
H	-4.062564	-0.093940	1.925380
H	-0.816031	-1.447799	-0.633217
H	-2.387262	-3.008627	-1.780333
F	-4.672174	-3.682949	-2.513357
F	-6.315287	-2.984805	-1.226201
F	-5.018126	-4.623874	-0.563055
C	5.315033	-1.748116	-0.500057
C	5.259775	-0.758657	0.495664
C	4.054436	-0.476064	1.148192
C	2.947724	-2.177200	-0.196367
C	4.150367	-2.455035	-0.849618
C	6.630866	-2.090638	-1.159773
H	6.163548	-0.201182	0.756028
H	4.009432	0.299826	1.912618
H	2.038850	-2.705353	-0.501037
H	4.186026	-3.204154	-1.645110
F	6.456675	-2.528450	-2.439009
F	7.278419	-3.085652	-0.483773
F	7.473689	-1.021783	-1.199111
C	-3.713894	3.048556	-2.606803
H	-3.312991	3.998119	-3.004775
H	-4.806632	3.184266	-2.513590
H	-3.494721	2.225826	-3.300642
C	-3.229901	3.836601	-0.183764
H	-2.249543	4.345006	-0.120982
H	-3.394841	3.366645	0.799868
H	-4.015868	4.574244	-0.404378

SCF(BP86) = -2365.60294659
H 0K = -2365.234935
H 298K = -2365.195108
G 298K = -2365.318725
Solvent Correction(Acetone) = -0.02250278
BP86-D3 Correction = -0.08391840
Lowest frequencies = -220.8957 cm-1, 5.4317 cm-1

R=**P-C6H4CF3/TS(1-2) (2,6)**

57

C	0.819370	-2.665571	-0.367083
Pd	2.111322	0.000563	-0.226655
C	0.532579	-0.614315	-1.823650
C	-0.974808	-0.838494	-1.783435
C	1.400812	-1.639987	-1.285300
H	2.299372	-1.937971	-1.843576
C	0.430384	1.111964	-0.728332
C	-0.631071	-2.561382	-0.057219
C	-1.510207	-1.711728	-0.678339
O	-1.670230	-0.373885	-2.686564
C	-2.950843	-1.689818	-0.321301
H	-0.989237	-3.301831	0.667345
O	1.488721	-3.599240	0.095978
H	0.831708	-0.153156	-2.772135
O	3.821627	-0.950915	0.479007
C	4.653027	-1.346139	-0.446585
C	3.339921	1.876815	1.883205
C	5.968837	-1.885921	0.194505
O	4.527332	-1.315825	-1.674931
O	2.951291	1.788982	0.698849
F	6.619616	-0.867391	0.836847
F	6.806173	-2.392254	-0.733587
F	5.715549	-2.854965	1.112403
C	-1.098113	3.448604	-0.362677
C	-1.278518	2.352968	0.499694
C	-0.534241	1.180487	0.307994
C	0.584680	2.209620	-1.608048
C	-0.166618	3.373725	-1.416851
H	-2.001154	2.417925	1.318056
H	-0.687429	0.328349	0.976653
H	1.312103	2.161549	-2.424750
H	-0.027338	4.231905	-2.080450
C	-5.675600	-1.711632	0.442317
C	-5.317182	-1.465209	-0.893362
C	-3.970082	-1.448843	-1.274601
C	-3.329971	-1.925760	1.024163
C	-4.674571	-1.939165	1.403781
H	-6.096088	-1.279278	-1.637806
H	-3.695644	-1.248509	-2.310779
H	-2.557450	-2.074558	1.785601
H	-4.950875	-2.107593	2.448166
C	3.078041	0.791600	2.892477
H	3.692346	-0.082685	2.606273
H	2.026266	0.466170	2.822279
H	3.316472	1.100749	3.920653
C	4.115582	3.097692	2.304624
H	4.261716	3.782629	1.458505
H	5.094454	2.786035	2.712165
H	3.584734	3.613362	3.125069
C	-1.953365	4.684196	-0.201681
C	-7.132437	-1.783558	0.839304
F	-7.914223	-1.003767	0.042112
F	-7.323685	-1.381417	2.128070
F	-7.609140	-3.058819	0.744736
F	-3.093395	4.602163	-0.945460
F	-1.294076	5.807709	-0.602714
F	-2.333301	4.868434	1.094767

SCF(BP86) = -2365.60324282
H 0K = -2365.235083
H 298K = -2365.195267
G 298K = -2365.318595
Solvent Correction(Acetone) = -0.02292236
BP86-D3 Correction = -0.08345340
Lowest frequencies = -226.0669 cm-1, 7.3916 cm-1

R=**P-C6H4CF3/Int2 (2,5)**

57

C	0.561164	-1.630955	3.061113
Pd	-1.327182	0.418673	0.276424
C	-0.155437	0.623622	1.968619
C	1.262466	0.801602	1.544067
C	-0.576750	-0.671184	2.671103
H	-1.158551	-0.459487	3.583518
C	-1.513908	-1.349293	1.645392
C	1.846735	-1.477868	2.357109
C	2.175158	-0.415962	1.548104
O	1.713354	1.922201	1.267153
C	3.446089	-0.421067	0.785018
H	2.569720	-2.280799	2.546003
O	0.372744	-2.535680	3.879894
H	-0.559874	1.546849	2.409123
O	-2.816601	0.166324	-1.326550
O	-1.187435	2.403633	-0.248744
C	-1.759844	3.249219	0.566040
C	-2.856554	0.653940	-2.475720
C	-1.711795	4.685257	-0.045171
O	-2.297172	3.050364	1.657795
F	-2.367056	5.576704	0.724887
F	-2.306828	4.688103	-1.282834
F	-0.434766	5.113860	-0.203960
C	-3.148609	-2.816597	-0.148371
C	-3.688709	-2.316828	1.067857
C	-2.890482	-1.597204	1.945361
C	-0.979250	-1.856320	0.413035
C	-1.811736	-2.605633	-0.465272
C	-4.062822	-3.562390	-1.091185
H	-4.737618	-2.508160	1.312072
H	-3.302621	-1.222270	2.887127
H	0.102422	-1.858913	0.243304
H	-1.394579	-3.019849	-1.386489
F	-3.395779	-4.086067	-2.154879
F	-5.035293	-2.736311	-1.587221
F	-4.704613	-4.583310	-0.458585
C	5.869360	-0.553059	-0.673064
C	5.422199	0.672885	-0.152346
C	4.222698	0.744364	0.565902
C	3.909490	-1.648587	0.244338
C	5.105164	-1.717122	-0.475088
C	7.192706	-0.634659	-1.398575
H	6.013967	1.578058	-0.313903
H	3.871087	1.696330	0.963758
H	3.305714	-2.553595	0.366857
H	5.440052	-2.667434	-0.899608
F	7.191748	-1.620971	-2.340632
F	8.215079	-0.908718	-0.536587
F	7.501104	0.534661	-2.025293
C	-4.102499	0.446131	-3.300998
H	-3.839709	0.019226	-4.284800
H	-4.574206	1.425974	-3.501248
H	-4.809285	-0.211299	-2.777690
C	-1.724990	1.458353	-3.056869
H	-0.756383	1.025219	-2.759595
H	-1.754155	2.466928	-2.605458
H	-1.796159	1.544092	-4.151340

SCF(BP86) = -2365.64082589
H 0K = -2365.270467
H 298K = -2365.230615
G 298K = -2365.352702
Solvent Correction(Acetone) = -0.02215227
BP86-D3 Correction = -0.08550978
Lowest frequencies = 9.9132 cm-1, 10.9651 cm-1

R=P-C6H4CF3/Int2 (2,6)

57

C	0.272822	-2.317051	-0.217954
Pd	-1.814529	-0.278969	0.012464
C	0.253388	-0.635998	1.747406
C	1.793532	-0.628970	1.692478
C	-0.501186	-1.630000	0.857833
H	-1.183144	-2.308835	1.391079
C	-0.288190	0.739173	1.286451
C	1.701512	-1.934445	-0.406434
C	2.452443	-1.173896	0.455004
O	2.426891	-0.140612	2.629292
C	3.891921	-0.916564	0.195497
H	2.178309	-2.426100	-1.263346
O	-0.195073	-3.229728	-0.916683
H	-0.018027	-0.767261	2.807666
O	-3.232834	-1.692857	-0.466848
C	-3.780792	-2.341875	0.524680
C	-4.036806	1.278700	-1.597195
C	-4.922933	-3.260925	-0.013898
O	-3.520492	-2.310252	1.729797
O	-3.255549	1.266342	-0.622950
F	-5.803455	-2.533192	-0.773534
F	-5.621447	-3.827376	0.990962
F	-4.424217	-4.249647	-0.798342
C	-1.048163	3.348917	0.479001
C	-0.423238	2.510298	-0.436235
C	-0.047617	1.190476	-0.055896
C	-0.917334	1.635681	2.207102
C	-1.287752	2.912383	1.810767
H	-0.211940	2.863723	-1.448496
H	0.608682	0.619115	-0.720353
H	-1.092801	1.302132	3.234342
H	-1.762908	3.591578	2.524491
C	6.624256	-0.472520	-0.387924
C	6.204290	-0.646826	0.941358
C	4.852131	-0.860771	1.234655
C	4.331762	-0.730398	-1.138309
C	5.680894	-0.511889	-1.430126
H	6.938235	-0.607805	1.750881
H	4.531420	-0.983492	2.269443
H	3.599996	-0.731002	-1.952753
H	6.003432	-0.353853	-2.462782
C	-4.026367	0.211276	-2.658205
H	-4.493010	-0.696946	-2.234674
H	-2.989527	-0.072556	-2.901725
H	-4.569753	0.518733	-3.563877
C	-5.045405	2.396545	-1.696534
H	-4.891378	3.130960	-0.894925
H	-6.065020	1.973634	-1.631584
H	-4.973061	2.886841	-2.683251
C	-1.500734	4.733957	0.081896
C	8.091400	-0.295776	-0.704666
F	8.761212	0.309857	0.315393
F	8.278678	0.456210	-1.826635
F	8.700019	-1.497693	-0.924784
F	-1.001919	5.680816	0.924206
F	-2.864208	4.839134	0.137891
F	-1.121076	5.061173	-1.182806

SCF(BP86) = -2365.63951221
H 0K = -2365.269268
H 298K = -2365.229332
G 298K = -2365.352162
Solvent Correction(Acetone) = -0.02272609
BP86-D3 Correction = -0.08472645
Lowest frequencies = 10.1549 cm-1, 11.2168 cm-1

R=P-C6H4OMe/Int1

59

C	-0.242568	-0.799876	2.388313
Pd	1.571507	0.211402	0.031241
C	0.553366	-1.709473	0.174786
C	-0.868554	-1.774951	-0.309421
C	0.858358	-1.242360	1.468211
H	1.811999	-1.482989	1.954664
C	-0.104144	1.305415	-0.133853
C	-1.623559	-0.989659	1.923162
C	-1.970300	-1.434046	0.669367
O	-1.100073	-2.143107	-1.466403
C	-3.377016	-1.542675	0.244970
H	-2.392989	-0.746657	2.664151
O	0.016654	-0.342826	3.514880
H	1.281379	-2.295468	-0.410281
O	2.581142	1.848259	-0.925111
O	3.543128	-0.606692	0.246007
C	3.955592	-1.652097	-0.410171
C	3.493719	2.526250	-0.403384
C	5.508497	-1.786010	-0.313085
O	3.311157	-2.485310	-1.057749
F	6.113668	-0.683419	-0.854734
F	5.969743	-2.871993	-0.967018
F	5.912711	-1.872923	0.986307
C	-2.248531	3.105211	-0.500015
C	-1.608280	3.017839	0.751574
C	-0.539615	2.113669	0.927939
C	-0.753101	1.392010	-1.382201
C	-1.818191	2.284628	-1.564041
H	-1.923765	3.639179	1.594125
H	-0.067661	2.048518	1.914861
H	-0.438446	0.762193	-2.221484
H	-2.332484	2.360056	-2.527145
C	-6.119956	-1.759098	-0.459219
C	-5.175426	-2.677842	-0.969629
C	-3.829053	-2.566186	-0.632499
C	-4.337820	-0.628644	0.739449
C	-5.691040	-0.722462	0.397517
H	-5.527947	-3.474369	-1.631116
H	-3.113194	-3.281546	-1.039797
H	-4.008250	0.201573	1.372217
H	-6.395560	0.017838	0.783781
O	-7.411717	-1.954499	-0.859299
O	-3.297445	3.947789	-0.786498
C	-3.748678	4.808431	0.258098
C	-8.406418	-1.052571	-0.363747
H	-4.571083	5.398846	-0.171651
H	-2.948963	5.493433	0.600740
H	-4.126899	4.237102	1.128275
H	-8.209330	-0.013151	-0.686865
H	-8.471008	-1.085559	0.740017
H	-9.358539	-1.392988	-0.794810
C	4.260563	3.488297	-1.270000
H	3.907492	3.449502	-2.309233
H	5.336589	3.240755	-1.225026
H	4.156307	4.512847	-0.869738
C	3.860456	2.381520	1.046584
H	2.946014	2.367237	1.664111
H	4.547041	3.167040	1.394397
H	4.332323	1.386305	1.158873

SCF(BP86) = -1920.60532124
H 0K = -1920.182191
H 298K = -1920.143870
G 298K = -1920.261553
Solvent Correction(Acetone) = -0.02473685
BP86-D3 Correction = -0.08363437
Lowest frequencies = 3.6710 cm-1, 12.7566 cm-1

R=P-C6H4OMe/TS(1-2) (2,5)

59

C	0.766834	1.710085	-2.331508
Pd	-1.377947	-0.147695	-0.218886
C	-0.175254	-0.593722	-1.871982
C	1.180730	-1.051651	-1.436871
C	-0.427276	0.793780	-2.148456
H	-1.270185	1.047008	-2.801351
C	-1.101377	1.882736	-0.441777
C	2.031231	1.264568	-1.726589
C	2.252259	0.002365	-1.221532
O	1.416257	-2.261494	-1.321625
C	3.523999	-0.335387	-0.552427
H	2.840298	2.002456	-1.760994
O	0.678309	2.738430	-3.013207
H	-0.792517	-1.366826	-2.350727
O	-2.788859	0.275981	1.387922
O	-1.711968	-2.153900	0.272040
C	-2.355787	-2.852216	-0.620400
C	-2.680663	-0.153829	2.555450
C	-2.706300	-4.266685	-0.063632
O	-2.714052	-2.531217	-1.759002
F	-1.606897	-4.904048	0.420035
F	-3.266462	-5.054521	-1.005972
F	-3.600280	-4.149721	0.967730
C	-1.302140	4.575871	0.395651
C	-2.284451	4.025750	-0.457265
C	-2.173038	2.692402	-0.875537
C	-0.102787	2.456233	0.388705
C	-0.214728	3.778886	0.821378
H	-3.130460	4.625368	-0.802139
H	-2.942135	2.277239	-1.535500
H	0.756533	1.857927	0.707878
H	0.535788	4.227867	1.479173
C	6.008578	-0.862939	0.729366
C	5.337130	-1.881890	0.017886
C	4.117686	-1.627414	-0.605417
C	4.210424	0.668844	0.174925
C	5.431739	0.422674	0.811083
H	5.799898	-2.871657	-0.035038
H	3.605440	-2.423286	-1.146292
H	3.761913	1.663395	0.270355
H	5.915563	1.224219	1.374326
O	7.195002	-1.222359	1.304402
O	-1.304899	5.862543	0.857909
C	-2.364290	6.722754	0.425193
C	7.918860	-0.217562	2.021602
H	-2.168344	7.696476	0.896097
H	-3.352034	6.349129	0.754897
H	-2.367077	6.840915	-0.674364
H	7.341368	0.160670	2.886114
H	8.192968	0.631014	1.367114
H	8.833179	-0.709321	2.382774
C	-3.841172	0.014838	3.503029
H	-4.155231	-0.976757	3.876467
H	-4.684698	0.512303	3.005426
H	-3.522477	0.596796	4.386483
C	-1.439905	-0.856705	3.036554
H	-0.547717	-0.292683	2.715388
H	-1.391491	-1.832850	2.518025
H	-1.430396	-1.005413	4.126442

SCF(BP86) = -1920.58924056
H 0K = -1920.166446
H 298K = -1920.128938
G 298K = -1920.243252
Solvent Correction(Acetone) = -0.02630217
BP86-D3 Correction = -0.08209469
Lowest frequencies = -203.7870 cm-1, 9.7802 cm-1

R=P-C6H4OMe/TS(1-2) (2,6)

59

C	0.329536	-2.477263	-0.119376
Pd	1.614813	0.218058	-0.235010
C	0.046445	-0.596929	-1.776739
C	-1.458249	-0.786167	-1.715449
C	0.915356	-1.541050	-1.127469
H	1.836035	-1.869429	-1.630139
C	-0.097935	1.245901	-0.743662
C	-1.123676	-2.370712	0.142341
C	-2.005086	-1.574496	-0.551275
O	-2.148777	-0.374481	-2.651496
C	-3.443788	-1.529588	-0.211102
H	-1.488209	-3.061580	0.911125
O	1.009740	-3.345079	0.450559
H	0.358472	-0.188112	-2.744760
O	3.385332	-0.648373	0.465301
C	4.194574	-1.115421	-0.442844
C	2.879253	2.237232	1.699707
C	5.547023	-1.553229	0.199864
O	4.031672	-1.215290	-1.664174
O	2.457750	2.077712	0.535211
F	6.204676	-0.454555	0.687112
F	6.359066	-2.155193	-0.694853
F	5.356783	-2.408582	1.239494
C	-1.842420	3.467873	-0.615493
C	-1.942201	2.441522	0.346564
C	-1.087340	1.330246	0.264423
C	-0.024792	2.276255	-1.716462
C	-0.874752	3.377430	-1.646160
H	-2.685604	2.487074	1.146108
H	-1.187365	0.529506	1.003414
H	0.716826	2.220188	-2.520077
H	-0.819511	4.185497	-2.381775
C	-6.191111	-1.521731	0.529206
C	-5.807934	-1.292040	-0.810349
C	-4.462392	-1.290881	-1.173690
C	-3.849281	-1.742974	1.128883
C	-5.196899	-1.741744	1.506262
H	-6.591618	-1.123662	-1.554843
H	-4.178222	-1.111165	-2.211149
H	-3.090095	-1.888646	1.904651
H	-5.460562	-1.899649	2.554814
O	-7.536037	-1.501082	0.775778
O	-2.632162	4.582231	-0.651770
C	-3.651765	4.707736	0.344909
C	-7.970556	-1.761870	2.113935
H	-4.167542	5.653366	0.126083
H	-3.221143	4.752744	1.363106
H	-4.375458	3.873200	0.291059
H	-7.595490	-0.997588	2.820741
H	-7.650115	-2.763237	2.457557
H	-9.068643	-1.720859	2.084565
C	2.652540	1.212611	2.778697
H	3.264920	0.326470	2.525773
H	1.601737	0.877221	2.754508
H	2.916728	1.583126	3.780213
C	3.662933	3.483389	2.025829
H	3.772723	4.121085	1.138189
H	4.659101	3.197637	2.409864
H	3.161878	4.041433	2.837105

SCF(BP86) = -1920.58872203
H 0K = -1920.165826
H 298K = -1920.128347
G 298K = -1920.242255
Solvent Correction(Acetone) = -0.02681997
BP86-D3 Correction = -0.08297720
Lowest frequencies = -200.3466 cm-1, 10.6778 cm-1

R=P-C6H4OMe/Int2 (2,5)

59

C	-1.031749	-2.114144	-2.623571
Pd	1.186864	0.052744	-0.224194
C	0.006577	0.160587	-1.910368
C	-1.365047	0.592064	-1.516795
C	0.235397	-1.275470	-2.383778
H	0.830846	-1.299909	-3.311698
C	1.085974	-1.910245	-1.252344
C	-2.258590	-1.684224	-1.941120
C	-2.419977	-0.480263	-1.286608
O	-1.661342	1.795011	-1.458170
C	-3.628774	-0.210828	-0.484224
H	-3.086798	-2.400676	-1.999984
O	-0.984790	-3.149018	-3.300531
H	0.516921	0.942481	-2.492771
O	2.721995	-0.110490	1.370094
O	1.204190	2.090349	0.141287
C	1.857031	2.800572	-0.737043
C	2.709654	0.468727	2.474693
C	1.917877	4.291051	-0.277334
O	2.394770	2.450158	-1.790684
F	0.680836	4.848608	-0.234478
F	2.688708	5.037123	-1.096757
F	2.452090	4.376122	0.984283
C	2.511119	-3.408076	0.709862
C	3.112453	-3.113438	-0.545999
C	2.412149	-2.381195	-1.498196
C	0.500255	-2.205823	0.026998
C	1.210751	-2.961281	0.985166
H	4.123609	-3.457858	-0.774411
H	2.874619	-2.172229	-2.468326
H	-0.561896	-2.004137	0.198118
H	0.754227	-3.220959	1.944400
C	-5.975085	0.185604	1.077125
C	-5.336533	1.278442	0.450344
C	-4.185449	1.087739	-0.310495
C	-4.279632	-1.291211	0.164527
C	-5.432674	-1.109794	0.935176
H	-5.771485	2.274925	0.571210
H	-3.697110	1.934098	-0.794666
H	-3.853943	-2.297475	0.091255
H	-5.889242	-1.970717	1.429408
O	-7.096101	0.485625	1.799442
O	3.117996	-4.129703	1.698288
C	4.424892	-4.658093	1.441407
C	-7.787368	-0.594157	2.434781
H	4.703894	-5.210907	2.349522
H	5.161995	-3.853349	1.262708
H	4.421195	-5.350601	0.579398
H	-7.151541	-1.098158	3.186986
H	-8.140801	-1.340103	1.698400
H	-8.653055	-0.139704	2.937207
C	3.965521	0.473767	3.313862
H	4.237633	1.513819	3.568490
H	4.794308	-0.011473	2.780572
H	3.777268	-0.044982	4.271282
C	1.497186	1.192306	2.996248
H	0.579425	0.653137	2.711389
H	1.449313	2.169846	2.480766
H	1.540686	1.350867	4.084536

SCF(BP86) = -1920.62533758
H 0K = -1920.200414
H 298K = -1920.162777
G 298K = -1920.276910
Solvent Correction(Acetone) = -0.02707871
BP86-D3 Correction = -0.08346715
Lowest frequencies = 11.9080 cm-1, 13.3433 cm-1

R=P-C6H4OMe/Int2 (2,6)

59

C	0.446825	-2.094625	-0.224010
Pd	-1.481296	0.096931	0.118784
C	0.582708	-0.453896	1.764377
C	2.116261	-0.572580	1.698397
C	-0.258270	-1.374942	0.879079
H	-0.981672	-2.013432	1.408782
C	0.157220	0.970916	1.315180
C	1.895569	-1.824808	-0.418302
C	2.720604	-1.130828	0.437920
O	2.789731	-0.165596	2.647685
C	4.162936	-0.963692	0.152601
H	2.321784	-2.345778	-1.284320
O	-0.105086	-2.948380	-0.938869
H	0.308129	-0.563313	2.826415
O	-3.003360	-1.168684	-0.483833
C	-3.594367	-1.877815	0.436497
C	-3.653364	1.843933	-1.336345
C	-4.779752	-2.677928	-0.191129
O	-3.346498	-1.980552	1.640937
O	-2.875724	1.761737	-0.364996
F	-5.593577	-1.845331	-0.918929
F	-5.543390	-3.266484	0.753880
F	-4.327689	-3.642800	-1.033067
C	-0.245986	3.690611	0.576176
C	0.247101	2.789949	-0.372995
C	0.438455	1.427017	-0.015247
C	-0.348658	1.925785	2.258307
C	-0.545045	3.246679	1.898756
H	0.506514	3.111999	-1.383844
H	0.995859	0.784914	-0.704889
H	-0.564563	1.596822	3.279659
H	-0.924835	3.980618	2.615315
C	6.920797	-0.703685	-0.492564
C	6.503549	-0.784183	0.853972
C	5.151951	-0.905118	1.171859
C	4.602091	-0.871369	-1.189944
C	5.955664	-0.741837	-1.521218
H	7.265633	-0.753768	1.638175
H	4.843668	-0.959311	2.216271
H	3.863351	-0.873289	-1.998565
H	6.245800	-0.659252	-2.571379
O	8.268549	-0.582993	-0.691606
O	-0.463424	5.020987	0.359370
C	-0.131741	5.543072	-0.931454
C	8.737127	-0.516170	-2.041640
H	-0.371454	6.614866	-0.891292
H	-0.728735	5.062033	-1.729030
H	0.943953	5.414309	-1.154297
H	8.339573	0.372604	-2.567300
H	8.468766	-1.425789	-2.611167
H	9.831602	-0.438489	-1.974333
C	-3.564881	0.929488	-2.528858
H	-4.068389	-0.017380	-2.258447
H	-2.515104	0.666735	-2.734387
H	-4.048470	1.359372	-3.419547
C	-4.752512	2.880698	-1.308098
H	-4.742458	3.434987	-0.359659
H	-5.730461	2.384414	-1.443820
H	-4.636170	3.578628	-2.156732

SCF(BP86) = -1920.62444831
H 0K = -1920.199536
H 298K = -1920.161893
G 298K = -1920.276141
Solvent Correction(Acetone) = -0.02773727
BP86-D3 Correction = -0.08276494
Lowest frequencies = 11.2767 cm-1, 14.1131 cm-1

5.4b Stationary Points for [PdR'(CF_3CO_2)(acetone)(3)] (R = R' = p-$C_6H_4CF_3$; R = R' = p-C_6H_4OMe; BQ trans to CF_3CO_2)

p-C6H4CF3/Int1

57

```
C       0.315560      -1.835634      2.243618
Pd      2.215574      -0.746920      -0.010008
C       0.900101      -2.442711      -0.141200
C       -0.520968     -2.217470      -0.557074
C       1.308368      -2.270942      1.214244
H       2.165716      -2.820513      1.621641
C       0.806417      0.634692       0.234979
C       -1.073939     -1.606988      1.790715
C       -1.502042     -1.765000      0.499173
O       -0.865762     -2.437715      -1.724840
C       -2.904870     -1.484752      0.111719
H       -1.772777     -1.310648      2.580901
O       0.636536      -1.712243      3.436183
H       1.450482      -3.102778      -0.823509
O       3.763970      0.889868       0.423058
C       3.786795      1.152375       -0.825361
C       4.957616      -2.359800      0.220801
C       4.813195      2.201836       -1.316875
O       3.062943      0.559783       -1.680278
O       3.886857      -2.196591      -0.401525
F       4.923228      3.228602       -0.443295
F       4.485138      2.698991       -2.526962
F       6.034795      1.599039       -1.419135
C       -1.129212     2.619152       0.589015
C       -0.279432     2.269695       1.654319
C       0.699231      1.277862       1.478655
C       -0.010742     0.998992       -0.845864
C       -0.987693     1.993242       -0.661014
C       -2.248806     3.600237       0.827692
H       -0.375783     2.774754       2.620177
H       1.357282      1.011433       2.310430
H       0.097263      0.517795       -1.821908
H       -1.638957     2.278388       -1.491893
F       -1.881383     4.600216       1.676638
F       -3.333018     2.980834       1.405314
F       -2.691445     4.173563       -0.323530
C       -5.595833     -0.935016      -0.548567
C       -4.948969     -2.055168      -1.099228
C       -3.612581     -2.323435      -0.782581
C       -3.567806     -0.357466      0.652682
C       -4.898401     -0.080915      0.323735
C       -7.052707     -0.676420      -0.855127
H       -5.490778     -2.710548      -1.786546
H       -3.112599     -3.186322      -1.225389
H       -3.021876     0.335715       1.298607
H       -5.385595     0.811884       0.723943
F       -7.401425     -1.137041      -2.089811
F       -7.352668     0.651981       -0.809908
F       -7.867605     -1.299892      0.046686
C       6.001433      -3.283495      -0.352464
H       6.249467      -4.071252      0.381616
H       6.934284      -2.715883      -0.524557
H       5.657508      -3.732555      -1.294024
C       5.244360      -1.656265      1.522747
H       4.464943      -1.914871      2.261339
H       5.160846      -0.565947      1.361418
H       6.232789      -1.911414      1.930941
```

```
SCF(BP86) = -2365.63482378
H 0K = -2365.265827
H 298K = -2365.225608
G 298K = -2365.347007
Solvent Correction(Acetone) = -0.01814559
```

BP86-D3 Correction = -0.08921984
Lowest frequencies = 13.2640 cm-1, 13.6284 cm-1

R=P-C6H4CF3/TS(1-2) (2,5)

57

C	-0.432975	1.422944	2.506228
Pd	1.212682	-1.191524	0.670384
C	-0.194963	-1.082843	2.222110
C	-1.586480	-1.156191	1.701388
C	0.457218	0.190141	2.430372
H	1.283304	0.226040	3.149795
C	1.464577	0.850428	0.792788
C	-1.745145	1.326403	1.834620
C	-2.299706	0.148788	1.400130
O	-2.152229	-2.252089	1.561481
C	-3.608553	0.142731	0.703772
H	-2.302189	2.267808	1.770884
O	-0.090282	2.415249	3.151299
H	0.107320	-1.951341	2.824104
O	2.706625	-1.298471	-0.810455
C	3.876190	-1.475065	-0.260751
C	0.690678	-3.886056	-0.618015
C	4.977835	-1.705195	-1.339109
O	4.168247	-1.500852	0.940885
O	0.982588	-3.303649	0.449701
F	4.747712	-2.885699	-1.991363
F	6.210506	-1.768364	-0.792521
F	4.979598	-0.714145	-2.270542
C	2.417325	3.256618	-0.310947
C	3.167473	2.588683	0.677633
C	2.696443	1.395562	1.232039
C	0.702632	1.540588	-0.186999
C	1.191919	2.725776	-0.749984
C	2.902074	4.580988	-0.855656
H	4.128959	2.998798	0.998851
H	3.298801	0.858994	1.969625
H	-0.253919	1.135100	-0.528699
H	0.625446	3.239737	-1.531522
F	4.261344	4.650677	-0.877041
F	2.458701	5.620910	-0.090696
F	2.454555	4.799148	-2.124403
C	-6.098899	0.239533	-0.635246
C	-5.812776	-0.812807	0.251176
C	-4.579823	-0.867692	0.911320
C	-3.912606	1.193942	-0.197255
C	-5.141848	1.244641	-0.860677
C	-7.452506	0.320514	-1.303765
H	-6.557260	-1.595581	0.420478
H	-4.358770	-1.691050	1.590726
H	-3.161791	1.964981	-0.398203
H	-5.355754	2.052182	-1.565905
F	-7.983818	-0.915227	-1.520537
F	-7.384610	0.958749	-2.506161
F	-8.344146	1.010101	-0.534604
C	0.699564	-5.390134	-0.644402
H	1.397934	-5.739865	-1.426177
H	-0.302053	-5.760119	-0.928953
H	0.990619	-5.799330	0.332366
C	0.346593	-3.121866	-1.867266
H	1.229457	-2.518272	-2.149221
H	-0.466688	-2.410187	-1.640478
H	0.049567	-3.777248	-2.698815

SCF(BP86) = -2365.60304356
H 0K = -2365.234959
H 298K = -2365.195198
G 298K = -2365.317595
Solvent Correction(Acetone) = -0.02212807
BP86-D3 Correction = -0.08369856
Lowest frequencies = -245.0589 cm-1, 8.4847 cm-1

R=P-C6H4CF3/TS(1-2) (2,6)

57

C	-0.031085	-3.214907	0.549989
Pd	-2.092841	-1.070537	0.362341
C	-0.314826	-1.106919	1.917056
C	1.191158	-0.882374	1.844887
C	-0.855428	-2.363842	1.449698
H	-1.600265	-2.894323	2.059279
C	-0.737709	0.449330	0.693087
C	1.336589	-2.737538	0.224728
C	1.946180	-1.642860	0.784010
O	1.730489	-0.166188	2.687728
C	3.325471	-1.249209	0.404576
H	1.887655	-3.388112	-0.464262
O	-0.438322	-4.299877	0.099554
H	-0.725799	-0.693157	2.845048
O	-3.374650	0.256630	-0.656609
C	-4.200892	0.820294	0.180003
C	-3.919605	-2.955104	-1.154180
C	-5.255544	1.691536	-0.567078
O	-4.242036	0.717798	1.412209
O	-3.533662	-2.599936	-0.018853
F	-4.663598	2.580409	-1.409439
F	-6.034773	2.383658	0.291144
F	-6.070277	0.889208	-1.318416
C	0.214685	3.022703	0.069496
C	0.589158	1.929595	-0.730001
C	0.135211	0.643342	-0.410300
C	-1.102415	1.557821	1.498653
C	-0.632911	2.834151	1.180585
C	0.772216	4.397734	-0.221417
H	1.234202	2.085913	-1.598799
H	0.434044	-0.204244	-1.033160
H	-1.789879	1.419747	2.337144
H	-0.935434	3.694252	1.784484
F	-0.111897	5.379080	0.108586
F	1.087792	4.547773	-1.538131
F	1.911966	4.628216	0.493558
C	5.955443	-0.577871	-0.395262
C	5.542291	-0.361963	0.929879
C	4.240520	-0.688034	1.328671
C	3.755518	-1.448651	-0.931448
C	5.054031	-1.119982	-1.328846
C	7.378792	-0.277715	-0.806962
H	6.240908	0.069549	1.651811
H	3.921656	-0.509889	2.356041
H	3.051883	-1.839903	-1.673297
H	5.366308	-1.264399	-2.366625
F	7.915315	0.736355	-0.073508
F	7.460119	0.072933	-2.121722
F	8.183241	-1.366920	-0.633976
C	-5.034069	-3.960350	-1.255427
H	-5.389619	-4.255627	-0.259115
H	-4.680347	-4.847475	-1.811344
H	-5.865032	-3.530640	-1.843619
C	-3.305116	-2.395390	-2.408214
H	-3.721499	-2.842750	-3.322432
H	-2.213636	-2.559573	-2.374407
H	-3.466001	-1.301273	-2.406622

SCF(BP86) = -2365.60293094
H 0K = -2365.234776
H 298K = -2365.195008
G 298K = -2365.317206
Solvent Correction(Acetone) = -0.02203938
BP86-D3 Correction = -0.08430828
Lowest frequencies = -243.1049 cm-1, 8.8738 cm-1

R=P-C6H4CF3/Int2 (2,5)

57

C	-0.896780	-2.709701	-2.246003
Pd	1.049217	0.443924	-0.863843
C	-0.228876	-0.198140	-2.389180
C	-1.589904	0.150406	-1.922186
C	0.208335	-1.664211	-2.462806
H	0.695827	-1.895292	-3.424350
C	1.266439	-1.766791	-1.342601
C	-2.127439	-2.261008	-1.566865
C	-2.448332	-0.948952	-1.317463
O	-2.048262	1.303854	-2.048295
C	-3.667565	-0.613241	-0.542818
H	-2.821241	-3.066001	-1.295521
O	-0.733695	-3.888710	-2.573120
H	0.085177	0.431481	-3.235708
O	2.309617	1.150815	0.680872
C	3.511950	1.382598	0.230704
C	0.280426	3.350744	-0.884179
C	4.355733	2.208509	1.248238
O	3.994351	1.082243	-0.866800
O	0.875589	2.447468	-1.513544
F	3.968332	3.521877	1.183478
F	5.675297	2.153870	0.967986
F	4.178212	1.794076	2.529685
C	3.120332	-2.199229	0.757461
C	3.525270	-2.333014	-0.599079
C	2.620695	-2.117607	-1.627697
C	0.867477	-1.614095	0.032717
C	1.814942	-1.850683	1.072574
C	4.150167	-2.410047	1.844676
H	4.557667	-2.611454	-0.827290
H	2.933017	-2.228861	-2.670212
H	-0.195010	-1.567776	0.295817
H	1.507672	-1.758747	2.116597
F	5.057957	-1.395700	1.859682
F	4.846625	-3.565694	1.641414
F	3.586371	-2.478878	3.080318
C	-5.991350	-0.080781	0.982278
C	-5.669144	0.726492	-0.122019
C	-4.517851	0.470443	-0.876059
C	-4.007254	-1.415615	0.575892
C	-5.153571	-1.154509	1.332016
C	-7.263890	0.170171	1.758729
H	-6.321694	1.562143	-0.389805
H	-4.265877	1.100689	-1.728944
H	-3.345127	-2.236505	0.869838
H	-5.392503	-1.770901	2.202804
F	-7.606246	1.489259	1.755144
F	-7.149027	-0.223893	3.058367
F	-8.314751	-0.516898	1.223919
C	0.254090	4.732947	-1.474974
H	0.739237	5.438240	-0.776215
H	-0.793828	5.065012	-1.585198
H	0.764858	4.755793	-2.446932
C	-0.413362	3.082681	0.421923
H	0.251431	2.481878	1.066207
H	-1.313932	2.480922	0.199129
H	-0.718638	4.007128	0.934007

SCF(BP86) = -2365.63779164
H 0K = -2365.267460
H 298K = -2365.227701
G 298K = -2365.349449
Solvent Correction(Acetone) = -0.02187557
BP86-D3 Correction = -0.08739240
Lowest frequencies = 9.6450 cm-1, 11.6010 cm-1

R=P-C6H4CF3/Int2 (2,6)

57

C	0.748627	-2.619636	0.428609
Pd	-1.616650	-0.987945	0.339238
C	0.499267	-0.655100	2.088221
C	2.005227	-0.386747	1.921222
C	-0.087862	-1.902338	1.420944
H	-0.637942	-2.582492	2.089272
C	-0.314743	0.493624	1.446176
C	2.073086	-2.038806	0.078438
C	2.700811	-1.013041	0.741282
O	2.582071	0.353601	2.718881
C	4.051652	-0.552074	0.334035
H	2.598421	-2.579744	-0.718346
O	0.407942	-3.697905	-0.102596
H	0.303654	-0.645408	3.172945
O	-3.172046	-0.120286	-0.805292
C	-4.192111	0.184130	-0.051982
C	-2.897911	-3.502989	-0.697875
C	-5.459992	0.500196	-0.901759
O	-4.264321	0.190779	1.182083
O	-2.717735	-2.795016	0.318568
F	-5.176996	1.236177	-2.008088
F	-6.393417	1.162510	-0.184856
F	-6.016592	-0.679161	-1.324332
C	-1.587737	2.739955	0.277925
C	-0.845935	1.890242	-0.530012
C	-0.207277	0.745685	0.032295
C	-1.091010	1.387716	2.244764
C	-1.711043	2.487593	1.671794
C	-2.308569	3.933617	-0.307399
H	-0.746835	2.095575	-1.598125
H	0.546807	0.223109	-0.566037
H	-1.182751	1.198909	3.318355
H	-2.298402	3.169657	2.292442
F	-3.656275	3.742921	-0.296384
F	-1.935869	4.180673	-1.591668
F	-2.059283	5.060531	0.420832
C	6.620250	0.266695	-0.523324
C	6.301037	0.257672	0.844773
C	5.029150	-0.141086	1.272805
C	4.387881	-0.528233	-1.042321
C	5.655985	-0.124981	-1.469100
C	8.011749	0.645028	-0.976453
H	7.049375	0.571283	1.577678
H	4.784127	-0.134595	2.335082
H	3.633264	-0.801783	-1.786958
H	5.895480	-0.094847	-2.535438
F	8.597916	1.533099	-0.126196
F	8.003263	1.204921	-2.219318
F	8.826985	-0.448445	-1.037390
C	-3.699392	-4.767030	-0.553650
H	-4.014525	-4.917444	0.487640
H	-3.095004	-5.625241	-0.898384
H	-4.584648	-4.716281	-1.212937
C	-2.319320	-3.133598	-2.033897
H	-2.727113	-3.743711	-2.853389
H	-1.227276	-3.298533	-1.967911
H	-2.496529	-2.060376	-2.220426

SCF(BP86) = -2365.63719454
H 0K = -2365.266926
H 298K = -2365.227113
G 298K = -2365.349278
Solvent Correction(Acetone) = -0.02183828
BP86-D3 Correction = -0.08622688
Lowest frequencies = 8.9926 cm-1, 12.0797 cm-1

R=P-C6H4OMe/Int1

59

C	-0.405024	-1.788532	2.097536
Pd	1.624358	-0.665474	-0.024586
C	0.258164	-2.296316	-0.289375
C	-1.135747	-1.999525	-0.751166
C	0.616616	-2.202799	1.087481
H	1.437059	-2.802759	1.500517
C	0.252987	0.762580	0.210007
C	-1.767755	-1.523978	1.606069
C	-2.155156	-1.594909	0.289555
O	-1.424503	-2.130111	-1.948097
C	-3.532199	-1.267835	-0.127540
H	-2.493222	-1.273803	2.387951
O	-0.121989	-1.725021	3.306967
H	0.811447	-2.944078	-0.981350
O	3.279314	-2.158666	-0.395431
O	3.225675	0.886030	0.553068
C	3.311984	1.201891	-0.679486
C	4.299818	-2.384439	0.286576
C	4.394168	2.236180	-1.074059
O	2.609797	0.678208	-1.594509
F	4.488000	3.227577	-0.156714
F	4.150128	2.791181	-2.279919
F	5.604104	1.602975	-1.135047
C	-1.515250	2.919806	0.514023
C	-0.769391	2.460626	1.617311
C	0.121104	1.378329	1.459355
C	-0.484433	1.215710	-0.897337
C	-1.369288	2.291833	-0.741077
H	-0.856878	2.933780	2.598756
H	0.699976	1.034807	2.321714
H	-0.374459	0.746026	-1.878948
H	-1.954020	2.663763	-1.587800
C	-6.217203	-0.646504	-0.815147
C	-5.518134	-1.661475	-1.505495
C	-4.198153	-1.960133	-1.174881
C	-4.247375	-0.250078	0.546427
C	-5.569445	0.067558	0.215179
H	-6.037536	-2.203289	-2.301224
H	-3.670065	-2.741410	-1.723178
H	-3.739336	0.335991	1.318859
H	-6.076095	0.877152	0.745747
O	-7.503977	-0.429032	-1.224730
O	-2.406519	3.966882	0.553552
C	-2.542688	4.661857	1.792529
C	-8.250863	0.590002	-0.553580
H	-3.273066	5.463619	1.609626
H	-1.584290	5.109338	2.119362
H	-2.921767	4.001045	2.596590
H	-7.784028	1.585215	-0.678065
H	-8.359141	0.371366	0.525514
H	-9.243567	0.592562	-1.025874
C	5.357076	-3.310659	-0.260959
H	5.068888	-3.697382	-1.247899
H	5.526917	-4.144807	0.443556
H	6.316359	-2.766485	-0.335065
C	4.518326	-1.751201	1.637428
H	4.504969	-0.652465	1.514391
H	5.457188	-2.075570	2.109043
H	3.668906	-1.990657	2.301052

SCF(BP86) = -1920.61411118
H 0K = -1920.190787
H 298K = -1920.152704
G 298K = -1920.267113
Solvent Correction(Acetone) = -0.02214888
BP86-D3 Correction = -0.08547969
Lowest frequencies = 13.9436 cm-1, 15.7862 cm-1

R=P-C6H4OMe/TS(1-2) (2,5)

59

C	0.922746	1.850344	-2.170013
Pd	-0.999685	-0.780777	-0.675227
C	0.463656	-0.639312	-2.194083
C	1.814704	-0.900388	-1.629486
C	-0.050259	0.696880	-2.298907
H	-0.864319	0.891914	-3.005578
C	-1.077149	1.254205	-0.530388
C	2.191958	1.555363	-1.485427
C	2.626887	0.290221	-1.159718
O	2.262437	-2.059782	-1.586937
C	3.884595	0.091157	-0.411358
H	2.828606	2.428138	-1.303373
O	0.678051	2.951334	-2.678006
H	0.092880	-1.422653	-2.869888
O	-0.967642	-2.929200	-0.769118
O	-2.486034	-0.953784	0.804264
C	-3.682923	-0.934447	0.289585
C	-0.683663	-3.677807	0.189599
C	-4.765410	-1.184154	1.384299
O	-4.023634	-0.771507	-0.887851
F	-4.635986	-2.446674	1.895038
F	-6.018360	-1.067125	0.891099
F	-4.641045	-0.308734	2.419906
C	-1.763957	3.718152	0.669518
C	-2.569601	3.184934	-0.363663
C	-2.223578	1.966653	-0.956520
C	-0.264750	1.809892	0.497940
C	-0.615790	3.015194	1.103244
H	-3.467014	3.707412	-0.703538
H	-2.869891	1.543189	-1.730125
H	0.628628	1.278816	0.839339
H	-0.016532	3.442222	1.912856
C	6.316709	-0.168773	1.040701
C	5.946657	-1.137111	0.080918
C	4.752607	-1.014754	-0.626480
C	4.272539	1.047045	0.559299
C	5.464839	0.929919	1.282492
H	6.622355	-1.979125	-0.095690
H	4.475903	-1.771616	-1.360808
H	3.608459	1.890549	0.775809
H	5.713379	1.683041	2.033979
O	7.504441	-0.389724	1.679897
O	-2.005206	4.898408	1.308982
C	-3.155698	5.654035	0.909331
C	7.928337	0.575253	2.648302
H	-3.154720	6.552289	1.542649
H	-4.089834	5.087091	1.077578
H	-3.093352	5.953265	-0.153185
H	7.214968	0.646458	3.490846
H	8.057994	1.575884	2.195074
H	8.897621	0.216346	3.022370
C	-0.804297	-5.167589	0.008452
H	0.172689	-5.644355	0.206441
H	-1.146310	-5.413205	-1.006029
H	-1.510625	-5.573430	0.755194
C	-0.229885	-3.125649	1.515141
H	-1.004997	-2.428133	1.881741
H	0.689451	-2.534712	1.354727
H	-0.036253	-3.912411	2.259114

SCF(BP86) = -1920.59054262
H 0K = -1920.167883
H 298K = -1920.130304
G 298K = -1920.244797
Solvent Correction(Acetone) = -0.02490461
BP86-D3 Correction = -0.08328543
Lowest frequencies = -217.0588 cm-1, 9.2107 cm-1

R=P-C6H4OMe/TS(1-2) (2,6)

59

C	-0.407779	-2.911285	-0.530860
Pd	1.648872	-0.796741	-0.420772
C	-0.167669	-0.869220	-1.983488
C	-1.650151	-0.588908	-1.839806
C	0.384646	-2.100143	-1.493436
H	1.132020	-2.641507	-2.089750
C	0.328806	0.734957	-0.716944
C	-1.761419	-2.425839	-0.192686
C	-2.381077	-1.327317	-0.744777
O	-2.203665	0.149329	-2.659599
C	-3.733823	-0.908616	-0.320788
H	-2.298682	-3.062524	0.519602
O	0.028600	-3.970979	-0.036488
H	0.234420	-0.449018	-2.911913
O	2.905540	0.464727	0.696528
C	3.766757	1.081949	-0.061395
C	3.350956	-2.890765	0.971075
C	4.773432	1.909072	0.795821
O	3.876141	1.064266	-1.293023
O	3.090950	-2.364036	-0.130696
F	4.130504	2.747614	1.655186
F	5.596793	2.656077	0.026989
F	5.552082	1.070428	1.545375
C	-0.735031	3.311042	-0.251761
C	-1.093881	2.243122	0.597717
C	-0.578527	0.963631	0.349978
C	0.676132	1.821998	-1.566431
C	0.157294	3.089902	-1.332235
H	-1.773178	2.394420	1.439781
H	-0.864887	0.140507	1.010652
H	1.388490	1.668529	-2.381443
H	0.429077	3.939835	-1.965087
C	-6.338848	-0.190353	0.570399
C	-5.936540	0.072494	-0.757520
C	-4.659061	-0.273516	-1.193912
C	-4.153313	-1.150725	1.010145
C	-5.431241	-0.802183	1.461267
H	-6.652651	0.547526	-1.434469
H	-4.358584	-0.061517	-2.220514
H	-3.451976	-1.597221	1.722928
H	-5.704482	-0.995873	2.501310
O	-7.611044	0.191936	0.893712
O	-1.189355	4.589931	-0.126455
C	-2.088514	4.878391	0.950883
C	-8.068617	-0.076217	2.222861
H	-2.320098	5.949312	0.865468
H	-1.618541	4.680983	1.932158
H	-3.021902	4.291737	0.865328
H	-7.464153	0.461092	2.977904
H	-8.052696	-1.159169	2.447528
H	-9.104808	0.288639	2.261022
C	2.606248	-2.507880	2.222648
H	3.054283	-2.938979	3.130480
H	1.569793	-2.879028	2.120571
H	2.567788	-1.406960	2.293104
C	4.424276	-3.944275	1.039045
H	4.844933	-4.140208	0.043462
H	4.005094	-4.873216	1.465718
H	5.222554	-3.611646	1.727064

SCF(BP86) = -1920.59027394
H 0K = -1920.167541
H 298K = -1920.129957
G 298K = -1920.244255
Solvent Correction(Acetone) = -0.02528681
BP86-D3 Correction = -0.08443885
Lowest frequencies = -215.9888 cm-1, 10.6277 cm-1

R=P-C6H4OMe/Int2 (2,5)

59

C	-1.289066	-2.615179	-1.990672
Pd	0.906860	0.394095	-0.712542
C	-0.484696	-0.152380	-2.157942
C	-1.817125	0.280227	-1.682414
C	-0.119641	-1.636956	-2.168136
H	0.412802	-1.916348	-3.092131
C	0.880857	-1.766786	-0.981585
C	-2.501481	-2.092417	-1.341503
C	-2.744026	-0.762088	-1.075885
O	-2.201066	1.461853	-1.813739
C	-3.930386	-0.358959	-0.293558
H	-3.252232	-2.852635	-1.094188
O	-1.187263	-3.803011	-2.319925
H	-0.154787	0.434064	-3.030224
O	0.714228	2.474403	-1.104716
O	2.433089	0.818032	0.706522
C	3.560626	1.024626	0.094853
C	0.040843	3.272774	-0.419982
C	4.693645	1.404845	1.097652
O	3.818248	0.944526	-1.113654
F	4.347769	2.481338	1.856431
F	5.853094	1.696857	0.467669
F	4.946062	0.365993	1.959386
C	2.602304	-2.436738	1.188728
C	3.094018	-2.451574	-0.146890
C	2.249342	-2.128866	-1.201544
C	0.411820	-1.749054	0.382715
C	1.260277	-2.097263	1.442128
H	4.137007	-2.698917	-0.354310
H	2.631973	-2.149241	-2.226683
H	-0.649610	-1.580575	0.585736
H	0.905224	-2.091756	2.475765
C	-6.226992	0.292509	1.255664
C	-5.806887	1.137060	0.204058
C	-4.679212	0.822960	-0.552083
C	-4.368731	-1.190103	0.767393
C	-5.493859	-0.879446	1.539266
H	-6.393941	2.035894	-0.006759
H	-4.363460	1.478815	-1.363620
H	-3.794256	-2.088983	1.015282
H	-5.781970	-1.541626	2.359163
O	-7.341255	0.698910	1.934767
O	3.347653	-2.752413	2.277836
C	4.753951	-2.987367	2.094823
C	-7.817746	-0.138576	2.993378
H	5.149107	-3.184667	3.100965
H	5.246897	-2.095377	1.673151
H	4.937035	-3.867973	1.452284
H	-7.070367	-0.235415	3.803236
H	-8.087804	-1.145658	2.624292
H	-8.716037	0.358813	3.385679
C	-0.072308	4.701092	-0.883744
H	-1.138423	4.970756	-0.987885
H	0.447996	4.841755	-1.840695
H	0.355270	5.374457	-0.119253
C	-0.678808	2.845489	0.829733
H	-0.100136	2.060994	1.344371
H	-1.645798	2.410860	0.513178
H	-0.881765	3.691323	1.504747

SCF(BP86) = -1920.62570753
H 0K = -1920.200658
H 298K = -1920.163144
G 298K = -1920.276012
Solvent Correction(Acetone) = -0.02470999
BP86-D3 Correction = -0.08746411
Lowest frequencies = 10.9865 cm-1, 15.8094 cm-1

R=P-C6H4OMe/Int2 (2,6)

59

C	-1.039199	2.412197	0.438579
Pd	1.309997	0.766181	0.413456
C	-0.822999	0.454733	2.104051
C	-2.322595	0.183230	1.904147
C	-0.222758	1.702820	1.454228
H	0.310087	2.386560	2.133458
C	0.011124	-0.695297	1.478592
C	-2.343117	1.820672	0.053566
C	-2.986014	0.782586	0.690815
O	-2.917925	-0.539470	2.706425
C	-4.302848	0.294195	0.228707
H	-2.849760	2.361290	-0.755268
O	-0.689341	3.495179	-0.083541
H	-0.650360	0.440718	3.192701
O	2.827470	-0.192095	-0.733374
C	3.879557	-0.491725	-0.030166
C	2.496078	3.260778	-0.797834
C	5.029709	-1.030320	-0.937322
O	4.067065	-0.377391	1.186331
O	2.402624	2.581371	0.247000
F	4.583920	-1.963960	-1.834082
F	6.021870	-1.599697	-0.218635
F	5.573677	-0.006373	-1.661643
C	1.274472	-2.999429	0.386095
C	0.507483	-2.181954	-0.449307
C	-0.116492	-1.023066	0.083407
C	0.788517	-1.572213	2.305530
C	1.395305	-2.693941	1.775943
H	0.398012	-2.399358	-1.513070
H	-0.852075	-0.498822	-0.535492
H	0.894369	-1.342119	3.370092
H	2.001116	-3.358922	2.397476
C	-6.829651	-0.572447	-0.748152
C	-6.533683	-0.635195	0.631256
C	-5.293769	-0.217338	1.110708
C	-4.616979	0.339569	-1.151307
C	-5.855232	-0.085223	-1.645294
H	-7.301068	-1.015562	1.311734
H	-5.075900	-0.278215	2.177226
H	-3.861104	0.687454	-1.863488
H	-6.045790	-0.047962	-2.720497
O	-8.072577	-1.011388	-1.110250
O	1.946302	-4.114225	-0.008363
C	2.015166	-4.380596	-1.417780
C	-8.420328	-0.954021	-2.497159
H	2.646068	-5.274667	-1.518009
H	2.481460	-3.533077	-1.949294
H	1.014282	-4.590913	-1.838749
H	-7.754894	-1.590704	-3.110186
H	-8.388502	0.082647	-2.881881
H	-9.449195	-1.335362	-2.562740
C	1.854231	2.827304	-2.085816
H	2.231106	3.387849	-2.954605
H	0.769431	3.016488	-1.975925
H	2.005580	1.743042	-2.221259
C	3.251689	4.561480	-0.743828
H	3.623422	4.756791	0.271098
H	2.588366	5.382503	-1.070351
H	4.095312	4.529623	-1.456461

SCF(BP86) = -1920.62564045
H 0K = -1920.200576
H 298K = -1920.163063
G 298K = -1920.276824
Solvent Correction(Acetone) = -0.02392992
BP86-D3 Correction = -0.08581086
Lowest frequencies = 8.0890 cm-1, 14.0745 cm-1

5.5 Stationary Points for [PdR'(acetone)$_2$(3)] (R = R' = p-$C_6H_4CF_3$; R = R' = p-C_6H_4OMe)

R=P-C6H4CF3/Int1

60

```
C       0.836372      -1.354627     2.305739
Pd      2.676236      -0.378082     -0.075857
C       1.419651      -2.173014     -0.008093
C       -0.022720     -2.013982     -0.425202
C       1.833409      -1.866737     1.298285
H       2.751697      -2.282412     1.731269
C       1.173433      0.927174      0.085706
C       -0.575289     -1.311424     1.892695
C       -1.023001     -1.611888     0.628466
O       -0.328099     -2.235191     -1.600726
C       -2.448491     -1.498969     0.257049
H       -1.278185     -1.017752     2.679594
O       1.214661      -1.040550     3.442601
H       2.004129      -2.828495     -0.667447
O       4.420611      -1.744270     -0.363390
C       5.500968      -1.811415     0.266793
C       3.977860      1.733799      -1.918382
O       3.872566      1.249189      -0.764825
C       -0.847828     2.830712      0.384968
C       0.118451      2.671620      1.393340
C       1.140925      1.718236      1.245560
C       0.231294      1.094715      -0.940945
C       -0.785236     2.055512      -0.784154
C       -2.015776     3.766992      0.610375
H       0.074978      3.287156      2.296590
H       1.876495      1.592482      2.046542
H       0.243570      0.471162      -1.840139
H       -1.535002     2.187793      -1.569303
F       -1.658688     4.847345      1.351553
F       -3.016041     3.124141      1.289266
F       -2.542279     4.210128      -0.560328
C       -5.193920     -1.292775     -0.362070
C       -4.430877     -2.347543     -0.891929
C       -3.067358     -2.446108     -0.596309
C       -3.229636     -0.437256     0.778097
C       -4.587949     -0.332019     0.467976
C       -6.680759     -1.213055     -0.646694
H       -4.905542     -3.086381     -1.542764
H       -2.484108     -3.268825     -1.014009
H       -2.758863     0.339332      1.388193
H       -5.175626     0.505898      0.851777
F       -6.991755     -1.781805     -1.842161
F       -7.113305     0.076639      -0.669958
F       -7.391986     -1.864541     0.313947
C       4.871680      2.924368      -2.115364
H       4.328898      3.717279      -2.659852
H       5.723979      2.638470      -2.759210
H       5.243340      3.307349      -1.155678
C       3.232418      1.176433      -3.096332
H       2.926894      0.133118      -2.920053
H       3.822227      1.256268      -4.023522
H       2.319383      1.784822      -3.242726
C       5.783880      -0.957848     1.475150
H       5.049671      -0.145896     1.583440
H       6.805767      -0.544404     1.435057
H       5.746705      -1.595262     2.378873
C       6.552870      -2.787851     -0.185156
H       7.427327      -2.228475     -0.566613
H       6.167970      -3.445568     -0.976095
H       6.919768      -3.384815     0.667903
```

SCF(BP86) = -2032.36983062
H 0K = -2031.945449
H 298K = -2031.905514
G 298K = -2032.026519

```
Solvent Correction(Acetone) = -0.07105763
BP86-D3 Correction = -0.08871507
Lowest frequencies = 12.6809 cm-1, 13.8534 cm-1
```

R=P-C6H4CF3/TS(1-2) (2,5)

60

C	-0.246356	-1.166854	2.394448
Pd	-1.460167	1.510372	0.357807
C	-0.126925	1.330311	1.971721
C	1.273434	1.168830	1.451017
C	-0.929869	0.189224	2.281948
H	-1.776500	0.305082	2.967759
C	-2.099303	-0.427526	0.578284
C	1.092544	-1.286795	1.792580
C	1.831177	-0.227434	1.318807
O	1.930874	2.179300	1.162192
C	3.165660	-0.434972	0.714559
H	1.518150	-2.295661	1.818229
O	-0.774949	-2.071246	3.042260
H	-0.308607	2.284867	2.487099
O	-0.878541	3.557847	0.062279
C	0.004097	4.006611	-0.708674
C	-3.938695	2.266989	-1.354273
O	-2.816344	1.712082	-1.328354
C	-3.432779	-2.743274	-0.269539
C	-4.046448	-1.880470	0.658171
C	-3.380810	-0.726844	1.091639
C	-1.464154	-1.316750	-0.320263
C	-2.145582	-2.460402	-0.759414
C	-4.188812	-3.954497	-0.781863
H	-5.036319	-2.123728	1.054617
H	-3.858514	-0.071955	1.827071
H	-0.454071	-1.113923	-0.689463
H	-1.671318	-3.143882	-1.469093
F	-5.019045	-4.458923	0.166424
F	-3.342733	-4.940210	-1.176445
F	-4.953960	-3.613685	-1.857707
C	5.704262	-0.940076	-0.418685
C	5.500295	0.202579	0.373648
C	4.242966	0.460931	0.930259
C	3.389782	-1.583847	-0.086139
C	4.643161	-1.833602	-0.650621
C	7.080477	-1.238064	-0.981259
H	6.329159	0.892816	0.551634
H	4.097279	1.348448	1.547089
H	2.562708	-2.271579	-0.290590
H	4.799299	-2.711630	-1.282764
F	7.779184	-0.095298	-1.216892
F	6.999336	-1.929550	-2.150076
F	7.806217	-1.990057	-0.110334
C	-4.710059	2.276940	-2.646440
H	-5.103975	3.284986	-2.861252
H	-5.589833	1.614803	-2.541882
H	-4.088974	1.918815	-3.478478
C	-4.546277	2.924615	-0.145226
H	-3.951635	2.725381	0.759348
H	-5.589291	2.593058	-0.003702
H	-4.588371	4.016769	-0.312865
C	0.756236	3.131881	-1.670498
H	0.189697	2.214885	-1.896882
H	1.706456	2.841233	-1.183436
H	1.010046	3.670336	-2.597290
C	0.332922	5.471432	-0.648896
H	1.418385	5.598817	-0.487655
H	-0.227819	5.972336	0.151493
H	0.106759	5.944637	-1.621614

SCF(BP86) = -2032.35380449
H 0K = -2031.929769
H 298K = -2031.890664
G 298K = -2032.010259
Solvent Correction(Acetone) = -0.06756135
BP86-D3 Correction = -0.08539378
Lowest frequencies = -200.7708 cm-1, 9.3335 cm-1

R=P-C6H4CF3/TS(1-2) (2,6)

60

C	-0.824157	-2.907199	0.573153
Pd	-2.605567	-0.555418	0.278327
C	-0.882276	-0.774262	1.919665
C	0.637732	-0.687334	1.821348
C	-1.560800	-1.943187	1.456877
H	-2.384217	-2.370073	2.047817
C	-1.149600	0.865597	0.553176
C	0.595524	-2.632528	0.294843
C	1.321989	-1.589800	0.825145
O	1.225854	0.040417	2.617459
C	2.740425	-1.375655	0.466450
H	1.082324	-3.374875	-0.347017
O	-1.397176	-3.908224	0.107208
H	-1.258406	-0.258340	2.810194
O	-3.667645	0.843333	-1.005506
O	-4.190563	-1.975721	-0.015281
C	-4.327876	-2.802123	-0.950432
C	-4.671245	1.540533	-0.732691
C	0.146212	3.283937	-0.029352
C	0.397639	2.149986	-0.820729
C	-0.229789	0.932181	-0.520075
C	-1.383361	2.000867	1.362471
C	-0.742370	3.208461	1.061036
H	1.085446	2.218720	-1.667850
H	-0.019252	0.050402	-1.132696
H	-2.060853	1.947360	2.220479
H	-0.927675	4.095403	1.673675
C	5.446164	-1.057970	-0.289172
C	5.030246	-0.711744	1.007157
C	3.690766	-0.859666	1.384087
C	3.177520	-1.713685	-0.841476
C	4.512716	-1.555886	-1.216982
H	5.757525	-0.319720	1.722895
H	3.377677	-0.586895	2.391987
H	2.454440	-2.072143	-1.581215
H	4.833103	-1.800615	-2.233089
C	-5.254178	2.420612	-1.805588
H	-4.625896	2.411297	-2.706263
H	-5.371082	3.453197	-1.432698
H	-6.270501	2.067106	-2.059071
C	-5.315151	1.537222	0.627599
H	-6.409067	1.421356	0.539739
H	-5.146199	2.518077	1.109142
H	-4.900442	0.740800	1.263958
C	-5.440318	-3.807681	-0.862166
C	-3.405509	-2.839408	-2.133927
H	-6.131120	-3.677874	-1.714746
H	-5.988471	-3.713229	0.084718
H	-5.020648	-4.825571	-0.956107
H	-2.944621	-1.853861	-2.306271
H	-3.916057	-3.195603	-3.042119
H	-2.599189	-3.559259	-1.893617
C	0.884825	4.580557	-0.304337
C	6.907037	-0.942267	-0.678893
F	7.537407	0.030686	0.029797
F	7.044511	-0.656018	-2.002125
F	7.561715	-2.112104	-0.444257
F	1.245609	4.677594	-1.610911
F	2.016053	4.659531	0.444632
F	0.112764	5.657842	0.001858

SCF(BP86) = -2032.35475989
H 0K = -2031.930603
H 298K = -2031.891510
G 298K = -2032.011115
Solvent Correction(Acetone) = -0.06637432
BP86-D3 Correction = -0.08575564
Lowest frequencies = -195.7197 cm-1, 9.9574 cm-1

R=P-C6H4CF3/Int2 (2,5)

60

C	-0.325372	-2.036202	-2.505458
Pd	1.408477	0.905183	-0.480918
C	0.134372	0.493117	-2.064089
C	-1.275573	0.615990	-1.564531
C	0.683090	-0.868863	-2.482553
H	1.154232	-0.824868	-3.478665
C	1.777062	-1.142528	-1.432833
C	-1.627971	-1.833525	-1.851601
C	-2.084799	-0.639832	-1.340005
O	-1.767884	1.742076	-1.379529
C	-3.382576	-0.574919	-0.629774
H	-2.265081	-2.725181	-1.822591
O	-0.009180	-3.112732	-3.014120
H	0.372791	1.322126	-2.750186
O	1.060649	2.979265	-0.704314
C	0.243425	3.681894	-0.057574
C	3.907396	1.736192	1.280997
O	2.712832	1.364372	1.237584
C	3.707945	-1.903131	0.495125
C	4.081430	-1.734954	-0.867031
C	3.139448	-1.348573	-1.811128
C	1.403814	-1.330361	-0.056079
C	2.389642	-1.721267	0.896623
C	4.786737	-2.233663	1.504691
H	5.114166	-1.930951	-1.170283
H	3.421486	-1.242683	-2.863217
H	0.346112	-1.412580	0.220402
H	2.102377	-1.889487	1.937403
F	5.608136	-3.211220	1.058887
F	4.278587	-2.604186	2.702124
F	5.571739	-1.119459	1.719052
C	-5.862379	-0.575504	0.726527
C	-5.525335	0.487915	-0.128271
C	-4.295485	0.496960	-0.795659
C	-3.740578	-1.641674	0.233939
C	-4.964081	-1.641706	0.909039
C	-7.215289	-0.599228	1.410097
H	-6.229251	1.312531	-0.269258
H	-4.045112	1.322746	-1.461874
H	-3.036412	-2.463675	0.399453
H	-5.220053	-2.459213	1.588034
F	-7.662397	0.659129	1.670483
F	-7.165441	-1.278726	2.587712
F	-8.144387	-1.208726	0.625007
C	4.540661	1.990193	2.622537
H	5.193134	2.878181	2.600877
H	5.185201	1.122623	2.856952
H	3.777724	2.084045	3.407594
C	4.746928	1.908252	0.045075
H	4.188165	1.634404	-0.862125
H	5.657425	1.289754	0.130861
H	5.087553	2.956554	-0.030108
C	-0.530609	3.155225	1.113828
H	-0.013977	2.299831	1.577185
H	-1.505709	2.801370	0.728047
H	-0.729129	3.942059	1.858296
C	0.028250	5.101049	-0.499051
H	-1.047474	5.259781	-0.695385
H	0.611968	5.330903	-1.400256
H	0.301531	5.793023	0.317684

SCF(BP86) = -2032.3918354
H 0K = -2031.965073
H 298K = -2031.926227
G 298K = -2032.043443
Solvent Correction(Acetone) = -0.06552714
BP86-D3 Correction = -0.08925652
Lowest frequencies = 10.7951 cm-1, 11.6797 cm-1

R=**P-C6H4CF3/Int2 (2,6)**

60

C	0.398209	-2.486317	0.322882
Pd	-2.080879	-0.954558	0.217550
C	0.050057	-0.465283	1.908703
C	1.547435	-0.122603	1.722692
C	-0.491565	-1.743591	1.275419
H	-1.002289	-2.429715	1.970865
C	-0.815807	0.634413	1.259330
C	1.746643	-1.951084	0.055373
C	2.332023	-0.870480	0.678399
O	2.021703	0.783248	2.403722
C	3.707306	-0.444531	0.341101
H	2.329493	-2.555180	-0.649598
O	0.038911	-3.559574	-0.199499
H	-0.131877	-0.448070	2.996182
O	-3.063309	-2.828399	0.282065
C	-3.080627	-3.697555	-0.626394
C	-4.875428	0.284441	-0.638083
O	-3.755862	-0.155201	-0.983569
C	-2.189397	2.797207	0.053059
C	-1.434792	1.947224	-0.747338
C	-0.741864	0.847759	-0.161533
C	-1.588369	1.537457	2.052799
C	-2.251159	2.604229	1.460716
C	-3.004892	3.916989	-0.557854
H	-1.360566	2.124777	-1.822959
H	0.017009	0.328535	-0.758511
H	-1.617150	1.401351	3.138261
H	-2.811257	3.313208	2.077568
F	-4.343502	3.587867	-0.512085
F	-2.688669	4.140071	-1.853942
F	-2.862532	5.073252	0.129822
C	6.345535	0.282145	-0.363287
C	5.903789	0.429704	0.962202
C	4.595591	0.079950	1.314005
C	4.169190	-0.582484	-0.993415
C	5.471216	-0.220682	-1.344310
C	7.778005	0.616374	-0.730510
H	6.584962	0.826671	1.719360
H	4.262802	0.202657	2.344914
H	3.487017	-0.943688	-1.769824
H	5.809287	-0.310097	-2.379986
F	8.287999	1.583909	0.076147
F	7.872271	1.048108	-2.017415
F	8.574740	-0.481016	-0.609299
C	-5.829247	0.762604	-1.699917
H	-5.528468	0.399559	-2.692385
H	-5.797831	1.868074	-1.703551
H	-6.867742	0.471299	-1.473459
C	-5.294424	0.384659	0.803160
H	-6.193544	-0.234457	0.973139
H	-5.584753	1.425349	1.030423
H	-4.488567	0.060687	1.478328
C	-3.665141	-5.042949	-0.305292
C	-2.520253	-3.450303	-1.994054
H	-2.906124	-5.821088	-0.504982
H	-4.513804	-5.255026	-0.979850
H	-3.990553	-5.097238	0.742073
H	-2.548686	-2.377918	-2.244465
H	-3.033593	-4.046856	-2.763829
H	-1.459864	-3.767230	-1.962949

SCF(BP86) = -2032.39288727
H 0K = -2031.966128
H 298K = -2031.927259
G 298K = -2032.044955
Solvent Correction(Acetone) = -0.06395613
BP86-D3 Correction = -0.08810340
Lowest frequencies = 9.4262 cm-1, 12.0837 cm-1

R=**P-C6H4OMe/Int1**

62

C	0.080901	-1.346318	2.186096
Pd	2.074843	-0.333623	-0.053722
C	0.731976	-2.046810	-0.147504
C	-0.678650	-1.786607	-0.616217
C	1.099035	-1.840395	1.192631
H	1.976672	-2.325908	1.637630
C	0.652146	1.061030	0.115860
C	-1.306232	-1.273397	1.729092
C	-1.722865	-1.479708	0.428002
O	-0.917650	-1.851940	-1.827154
C	-3.128131	-1.354779	0.022608
H	-2.032182	-1.045416	2.516299
O	0.430520	-1.095650	3.352201
H	1.311013	-2.693069	-0.820886
O	3.414108	1.256060	-0.597655
O	3.776137	-1.771295	-0.344073
C	4.806996	-1.930712	0.346579
C	3.558837	1.820813	-1.707850
C	-1.093944	3.248516	0.350026
C	-0.216017	2.929638	1.407345
C	0.657391	1.830172	1.286994
C	-0.230325	1.358907	-0.938680
C	-1.096666	2.455017	-0.819568
H	-0.199703	3.518616	2.327530
H	1.320970	1.590713	2.124661
H	-0.275882	0.740767	-1.840844
H	-1.795344	2.705792	-1.623361
C	-5.884106	-1.162044	-0.644112
C	-5.031772	-2.035540	-1.362136
C	-3.682245	-2.123009	-1.042228
C	-3.998373	-0.481352	0.725117
C	-5.352213	-0.373900	0.404364
H	-5.463704	-2.640655	-2.163921
H	-3.046443	-2.807527	-1.605423
H	-3.594272	0.159422	1.515077
H	-5.982452	0.327620	0.955446
O	-7.180629	-1.149275	-1.041752
O	-1.979454	4.287238	0.357572
C	-2.017389	5.124814	1.520861
C	-8.103956	-0.302560	-0.336776
H	-2.781787	5.886191	1.313482
H	-1.044306	5.620667	1.694766
H	-2.304354	4.553108	2.423003
H	-7.824661	0.762109	-0.433692
H	-8.162836	-0.577257	0.731605
H	-9.078885	-0.470307	-0.813688
C	4.525370	2.966645	-1.812728
H	4.924451	3.242494	-0.827570
H	4.028292	3.834871	-2.280857
H	5.354299	2.685854	-2.488279
C	2.783843	1.405138	-2.925352
H	3.336237	1.609101	-3.855718
H	1.853328	2.004330	-2.951324
H	2.495291	0.343500	-2.866938
C	5.848270	-2.921260	-0.103499
C	5.043504	-1.174285	1.628592
H	6.769179	-2.377987	-0.385628
H	5.491471	-3.504310	-0.963255
H	6.129459	-3.592549	0.726513
H	4.922193	-1.868800	2.481260
H	4.334040	-0.341886	1.746178
H	6.080573	-0.802000	1.680913

SCF(BP86) = -1587.36335437
H 0K = -1586.884447
H 298K = -1586.846668
G 298K = -1586.960363

Solvent Correction(Acetone) = -0.06394840
BP86-D3 Correction = -0.08481510
Lowest frequencies = 14.3340 cm-1, 14.6895 cm-1

R=P-C6H4OMe/TS(1-2) (2,5)

62

C	0.074076	1.646934	-2.066795
Pd	-1.410224	-1.057763	-0.309812
C	-0.048106	-0.877064	-1.932913
C	1.356838	-0.918332	-1.402203
C	-0.712445	0.357209	-2.145743
H	-1.593896	0.404274	-2.794342
C	-1.822907	0.930547	-0.221178
C	1.415360	1.561820	-1.482624
C	2.053233	0.389015	-1.124204
O	1.893594	-2.024553	-1.225895
C	3.387556	0.399250	-0.504259
H	1.940388	2.519914	-1.411258
O	-0.382906	2.681822	-2.569695
H	-0.343392	-1.770377	-2.502112
O	-2.736488	-1.340902	1.383818
O	-1.053255	-3.197673	-0.326023
C	-0.222672	-3.840836	0.357794
C	-3.920544	-1.744878	1.359800
C	-2.696487	3.457748	0.668377
C	-3.463424	2.736349	-0.276536
C	-3.018994	1.483116	-0.721901
C	-1.043631	1.665664	0.707031
C	-1.489554	2.904984	1.164303
H	-4.390190	3.149685	-0.681347
H	-3.612272	0.949122	-1.471570
H	-0.097188	1.264490	1.083472
H	-0.915153	3.479452	1.896809
C	5.978100	0.553141	0.659977
C	5.625110	-0.540309	-0.167261
C	4.356330	-0.620566	-0.730666
C	3.761285	1.484856	0.330065
C	5.027627	1.569901	0.912216
H	6.378389	-1.309280	-0.360129
H	4.108797	-1.465660	-1.373569
H	3.028766	2.267333	0.554512
H	5.265473	2.413474	1.564054
O	7.238400	0.527162	1.161837
O	-3.018569	4.682022	1.155524
C	-4.197896	5.330160	0.648868
C	7.669312	1.626667	1.981213
H	-4.243145	6.299898	1.162127
H	-5.107309	4.747428	0.882982
H	-4.126912	5.491799	-0.441521
H	7.060781	1.704177	2.900386
H	7.627922	2.580379	1.425198
H	8.710488	1.402837	2.249499
C	-4.668723	-1.870567	2.660518
H	-3.995512	-1.726313	3.516266
H	-5.177190	-2.847668	2.727390
H	-5.463648	-1.102465	2.691661
C	-4.624621	-2.116096	0.082863
H	-5.632880	-1.669839	0.045040
H	-4.766551	-3.212651	0.059496
H	-4.033156	-1.816316	-0.795935
C	-0.032767	-5.305589	0.075660
C	0.608258	-3.197024	1.431737
H	1.033389	-5.501349	-0.137961
H	-0.650132	-5.628765	-0.773200
H	-0.283901	-5.897839	0.973910
H	0.821288	-3.894341	2.257294
H	0.124302	-2.282390	1.808952
H	1.574640	-2.911529	0.973899

SCF(BP86) = -1587.35274348
H 0K = -1586.873916
H 298K = -1586.837098
G 298K = -1586.947482

```
Solvent Correction(Acetone) = -0.06044688
BP86-D3 Correction = -0.08505657
Lowest frequencies = -141.9418 cm-1, 13.4581 cm-1
```

R=P-C6H4OMe/TS(1-2) (2,6)

62

C	-0.397580	-2.640254	0.556999
Pd	-2.145667	-0.243997	0.256139
C	-0.442763	-0.528967	1.922893
C	1.062591	-0.401775	1.783738
C	-1.126511	-1.675678	1.444583
H	-1.981628	-2.083896	2.002128
C	-0.689610	1.165772	0.430934
C	1.013818	-2.372371	0.284867
C	1.749314	-1.317829	0.798259
O	1.647117	0.364835	2.552029
C	3.159089	-1.112048	0.435903
H	1.499530	-3.129004	-0.340141
O	-0.986154	-3.639886	0.093892
H	-0.827039	0.019206	2.790154
O	-3.768441	-1.665326	0.057788
C	-3.940268	-2.514046	-0.849717
C	-4.190054	1.861315	-0.750042
O	-3.235674	1.110932	-1.051841
C	0.843477	3.482212	-0.046857
C	1.027720	2.337359	-0.854389
C	0.278140	1.179366	-0.597885
C	-0.854471	2.307274	1.253070
C	-0.104873	3.456090	1.008468
H	1.755414	2.333622	-1.668976
H	0.443002	0.292602	-1.217881
H	-1.564508	2.299166	2.086306
H	-0.217525	4.349761	1.629256
C	5.892108	-0.842005	-0.314854
C	5.434880	-0.345346	0.929252
C	4.099092	-0.469818	1.294395
C	3.637146	-1.588483	-0.814697
C	4.973214	-1.460243	-1.195906
H	6.159935	0.125908	1.598724
H	3.766822	-0.088284	2.259804
H	2.937899	-2.043158	-1.523879
H	5.291714	-1.827852	-2.173983
O	7.212796	-0.667445	-0.569564
O	1.523798	4.647697	-0.183248
C	2.526269	4.735697	-1.209952
C	7.746267	-1.185632	-1.799793
H	2.942364	5.748613	-1.127867
H	2.084264	4.598495	-2.213483
H	3.327676	3.992489	-1.049340
H	7.285114	-0.694052	-2.675531
H	7.603974	-2.278931	-1.868180
H	8.819815	-0.955603	-1.774383
C	-4.732477	1.960564	0.650569
H	-4.471132	2.950679	1.067607
H	-4.312968	1.172040	1.293849
H	-5.834486	1.905023	0.647622
C	-4.819542	2.703263	-1.828568
H	-4.256506	2.622123	-2.768004
H	-4.876094	3.757976	-1.507366
H	-5.861651	2.372581	-1.991788
C	-5.052270	-3.514689	-0.701379
C	-3.053640	-2.588068	-2.058668
H	-5.579435	-3.384298	0.253230
H	-4.632763	-4.535142	-0.763741
H	-5.761099	-3.418194	-1.543267
H	-2.220342	-3.270611	-1.798803
H	-2.623410	-1.600890	-2.291146
H	-3.578185	-3.002926	-2.933116

SCF(BP86) = -1587.35424569
H 0K = -1586.875226
H 298K = -1586.838454
G 298K = -1586.948925

```
Solvent Correction(Acetone) = -0.05932739
BP86-D3 Correction = -0.08565029
Lowest frequencies = -143.2193 cm-1, 12.1638 cm-1
```

R=P-C6H4OMe/Int2 (2,5)

62

C	-0.577425	-2.251535	-2.105569
Pd	1.374602	0.712365	-0.386514
C	0.021102	0.268588	-1.881152
C	-1.378140	0.521260	-1.405697
C	0.503051	-1.154106	-2.121112
H	1.049503	-1.239517	-3.075050
C	1.516538	-1.392360	-0.965127
C	-1.875852	-1.915065	-1.516496
C	-2.269707	-0.658549	-1.092161
O	-1.793899	1.691412	-1.320289
C	-3.558426	-0.466006	-0.406985
H	-2.574921	-2.756742	-1.454251
O	-0.314859	-3.381678	-2.529472
H	0.302389	1.006817	-2.650953
O	2.943949	1.079743	1.139699
O	1.017037	2.812428	-0.487624
C	0.139609	3.456967	0.137074
C	4.027510	1.682084	0.986834
C	3.239715	-2.403430	1.068410
C	3.728770	-2.213925	-0.257806
C	2.885389	-1.715574	-1.243422
C	1.048531	-1.588476	0.385798
C	1.904006	-2.077430	1.381764
H	4.757179	-2.479378	-0.513696
H	3.254912	-1.607439	-2.268668
H	-0.018374	-1.484829	0.608676
H	1.546465	-2.248675	2.400372
C	-6.074672	-0.242009	0.903947
C	-5.603863	0.797825	0.066934
C	-4.370765	0.693523	-0.568592
C	-4.049527	-1.496093	0.437707
C	-5.279311	-1.395927	1.091902
H	-6.240885	1.674930	-0.078266
H	-4.032326	1.496901	-1.222804
H	-3.434920	-2.385311	0.613783
H	-5.606728	-2.204864	1.748800
O	-7.286812	-0.032414	1.477879
O	3.977149	-2.901716	2.086582
C	5.310102	-3.376863	1.815262
C	-7.836083	-1.070523	2.306038
H	5.676576	-3.774826	2.770606
H	5.969507	-2.555579	1.482120
H	5.299300	-4.181161	1.059295
H	-7.198350	-1.259656	3.188621
H	-7.971072	-2.007651	1.736721
H	-8.814920	-0.696973	2.635565
C	4.904914	1.931247	2.187555
H	4.537826	1.378836	3.063180
H	4.904367	3.013476	2.415390
H	5.953090	1.662297	1.971290
C	4.501094	2.168565	-0.358740
H	5.338144	1.527620	-0.693111
H	4.902579	3.193721	-0.289559
H	3.695201	2.122578	-1.106261
C	-0.142862	4.871768	-0.285597
C	-0.642386	2.869625	1.275936
H	-1.201881	4.943001	-0.593576
H	0.503455	5.171173	-1.121453
H	-0.014554	5.562476	0.565981
H	-0.802474	3.610044	2.076458
H	-0.143966	1.969569	1.669338
H	-1.635213	2.576709	0.886674

SCF(BP86) = -1587.38896358
H 0K = -1586.907769
H 298K = -1586.870897
G 298K = -1586.981484

```
Solvent Correction(Acetone) = -0.06068470
BP86-D3 Correction = -0.08567626
Lowest frequencies = 13.0460 cm-1, 14.8928 cm-1
```

R=P-C6H4OMe/Int2 (2,6)

62

C	-0.411578	-2.310068	-0.391982
Pd	1.899051	-0.496777	-0.312453
C	-0.228450	-0.262831	-1.966809
C	-1.734844	-0.010915	-1.736313
C	0.409765	-1.488052	-1.333867
H	0.974999	-2.120700	-2.037188
C	0.603456	0.906112	-1.362237
C	-1.764142	-1.843882	-0.062566
C	-2.436840	-0.786093	-0.650975
O	-2.283422	0.849851	-2.423296
C	-3.805152	-0.428270	-0.247744
H	-2.289773	-2.491707	0.647957
O	0.011546	-3.396393	0.060350
H	-0.082511	-0.246712	-3.060106
O	2.853271	-2.143390	0.663758
C	3.147595	-3.317494	0.338932
C	4.063137	0.862499	1.576084
O	3.621774	0.697756	0.421014
C	1.624378	3.396919	-0.438692
C	0.811830	2.623705	0.414646
C	0.312973	1.388300	-0.038881
C	1.456918	1.713046	-2.193203
C	1.959270	2.919449	-1.741061
H	0.534520	2.980788	1.408703
H	-0.417231	0.855196	0.579404
H	1.678100	1.376522	-3.211187
H	2.594111	3.546199	-2.373259
C	-6.460432	0.150773	0.593935
C	-6.049166	0.429993	-0.731278
C	-4.749442	0.155886	-1.141638
C	-4.236948	-0.692591	1.079118
C	-5.534855	-0.408946	1.506290
H	-6.779943	0.858552	-1.422718
H	-4.453287	0.377847	-2.166745
H	-3.527039	-1.099484	1.806895
H	-5.817389	-0.610352	2.542019
O	-7.747484	0.462328	0.888668
O	2.133302	4.611485	-0.133336
C	1.755984	5.222771	1.114498
C	-8.233032	0.178985	2.211180
H	2.250072	6.203087	1.121611
H	2.107366	4.625617	1.975196
H	0.661762	5.356827	1.173941
H	-7.678553	0.755305	2.973833
H	-8.167710	-0.900086	2.439040
H	-9.285997	0.491131	2.210806
C	3.326314	0.386599	2.803022
H	4.020756	-0.062834	3.532407
H	2.532098	-0.326360	2.541120
H	2.876036	1.264893	3.302741
C	5.378654	1.575908	1.765563
H	5.708364	2.045543	0.828852
H	6.140114	0.840934	2.087147
H	5.310915	2.325011	2.572912
C	3.627924	-4.258510	1.409287
C	3.002955	-3.836787	-1.059944
H	3.629852	-3.775577	2.395509
H	4.639794	-4.626323	1.162087
H	2.968643	-5.145320	1.428659
H	3.649869	-4.705527	-1.251963
H	3.192138	-3.040803	-1.797111
H	1.944060	-4.152079	-1.147187

SCF(BP86) = -1587.38838114
H 0K = -1586.906984
H 298K = -1586.870148
G 298K = -1586.981767

```
Solvent Correction(Acetone) = -0.06025810
BP86-D3 Correction = -0.08563137
Lowest frequencies = 8.3358 cm-1, 10.4404 cm-1
```
